# Comparative Analysis of the Genomes of Two Field Isolates of the Rice Blast Fungus *Magnaporthe oryzae*

**DOI:** 10.1371/journal.pgen.1002869

**Published:** 2012-08-02

**Authors:** Minfeng Xue, Jun Yang, Zhigang Li, Songnian Hu, Nan Yao, Ralph A. Dean, Wensheng Zhao, Mi Shen, Haiwang Zhang, Chao Li, Liyuan Liu, Lei Cao, Xiaowen Xu, Yunfei Xing, Tom Hsiang, Ziding Zhang, Jin-Rong Xu, You-Liang Peng

**Affiliations:** 1 State Key Laboratory of Agrobiotechnology and Ministry of Agriculture Key Laboratory of Plant Pathology, China Agricultural University, Beijing, China; 2 Beijing Genomics Institute, Chinese Academy of Sciences, Beijing, China; 3 State Key Laboratory of Biocontrol, School of Life Sciences, Sun Yat-sen University, Guangzhou, China; 4 Fungal Genomics Laboratory, Center for Integrated Fungal Research, North Carolina State University, Raleigh, North Carolina, United States of America; 5 School of Environmental Sciences, University of Guelph, Guelph, Canada; 6 State Key Laboratory of Agrobiotechnology, College of Biological Sciences, China Agricultural University, Beijing, China; 7 Department of Botany and Plant Pathology, Purdue University, West Lafayette, Indiana, United States of America; Progentech, United States of America

## Abstract

Rice blast caused by *Magnaporthe oryzae* is one of the most destructive diseases of rice worldwide. The fungal pathogen is notorious for its ability to overcome host resistance. To better understand its genetic variation in nature, we sequenced the genomes of two field isolates, Y34 and P131. In comparison with the previously sequenced laboratory strain 70-15, both field isolates had a similar genome size but slightly more genes. Sequences from the field isolates were used to improve genome assembly and gene prediction of 70-15. Although the overall genome structure is similar, a number of gene families that are likely involved in plant-fungal interactions are expanded in the field isolates. Genome-wide analysis on asynonymous to synonymous nucleotide substitution rates revealed that many infection-related genes underwent diversifying selection. The field isolates also have hundreds of isolate-specific genes and a number of isolate-specific gene duplication events. Functional characterization of randomly selected isolate-specific genes revealed that they play diverse roles, some of which affect virulence. Furthermore, each genome contains thousands of loci of transposon-like elements, but less than 30% of them are conserved among different isolates, suggesting active transposition events in *M. oryzae*. A total of approximately 200 genes were disrupted in these three strains by transposable elements. Interestingly, transposon-like elements tend to be associated with isolate-specific or duplicated sequences. Overall, our results indicate that gain or loss of unique genes, DNA duplication, gene family expansion, and frequent translocation of transposon-like elements are important factors in genome variation of the rice blast fungus.

## Author Summary

*Magnaporthe oryzae* is the causal agent of rice blast that is mainly controlled with resistance cultivars. However, genetic variations in the pathogen often lead to overcoming R gene-mediated resistance in rice cultivars. In this study we sequenced two field isolates from China and Japan. In comparison with the laboratory strain that was previously sequenced, the field isolates have a similar genome size and overall genome structure. However, they have slightly more genes and contain a number of expanded gene families that are likely involved in plant-fungal interactions. Each of the isolates has specific genes, some of which affect virulence and some others are important for asexual development. The three strains differ noticeably in the distribution of transposon-like elements. Many of the transposable elements tend to be associated with isolate-specific or duplicated sequences. This study revealed genetic factors involved in genome variation of the rice blast fungus.

## Introduction

Rice blast caused by the heterothallic ascomycete *Magnaporthe oryzae* (also known as *Pyricularia oryzae*) is one of the most destructive diseases of rice, which is a staple for over half of the world's population. This pathogen also infects wheat and other small grains, and poses major threats to global food security [1], [2]. In the past two decades, rice blast has been developed as a model system to study fungal-plant interactions. *M. oryzae* was the first plant pathogenic fungus to have its genome sequenced and made available to the public [3].

In most parts of the world, rice blast is controlled mainly with resistant cultivars. However, *M. oryzae* is notorious for its ability to overcome resistance based on race-specific R genes [4]–[6]. New cultivars often lose their resistance within a few years of introduction. Genetic variations in populations of the pathogen have been well-documented in many parts of the world [7], [8]. *M. oryzae* isolates are also known to lose virulence and female fertility during laboratory manipulations [1] and large chunks of genomic DNA can be lost spontaneously during cultivation on artificial media, such as the deletion of over a 40 kb region containing the *BUF1* locus [9]. The laboratory strain 70-15 of *M. oryzae* was generated by backcrossing a progeny from a cross between a rice isolate and a weeping love grass (*Eragrostis curvula*) isolate with the rice isolate Guy11 from French Guyana [10], [11]. It has been used in many laboratories and was selected for genome sequencing [3]. Although most of the 70-15 genome should be from the rice pathogen after backcrossing with Guy11 several times, some weeping love grass pathogen sequences are likely retained. In comparison with Guy11, 70-15 is reduced in female fertility, conidiation, and virulence [12].

To determine the extent of genetic variation among isolates of *M. oryzae*, we sequenced two field isolates Y34 and P131. Y34 was isolated from Japonica rice in 1982 in Yunnan province, China, where both Indica and Japonica rice cultivars are cultivated [13], [14]. Due to rich genetic diversity in rice cultivars and centuries of rice cultivation, highly diverse rice blast pathogen populations exist in Yunnan [15], and hence Y34 was chosen as a representative from this region for sequencing. The other field isolate, P131, originated from Japan where Japonica rice cultivars are dominant [16], [17]. The isolates P131, Y34, and 70-15 differ in some cultural characteristics (Figure S1). These three isolates also carry different avirulence genes and vary in aggressiveness toward different rice cultivars (Table S1). In comparison with 70-15, both Y34 and P131 have slightly larger genomes. The two Asian field isolates share a higher degree of similarity and contain over 200 genes that are absent in 70-15. Many pathogenesis-related genes showed evidence of exposure to diversifying selection when comparing either field isolate (P131 or Y34) to the laboratory strain (70-15). Functional characterization of randomly selected genes specific to the field isolates revealed that they play diverse roles, some of which affect virulence and others important for conidiation and vegetative growth. Furthermore, thousands of loci with transposon-like elements were identified in each genome. Many of them tend to be associated with the distribution of unique sequences and translocation of duplicated genes.

## Results

### Genome sequencing and assembly

The genomes of P131 and Y34 were sequenced with the Sanger (2-fold) and 454 sequencing technologies (18-fold). The combined sequence reads for P131 and Y34 were 793.94 Mb and 843.92 Mb, representing about 20- and 21-fold genome sequence coverage, respectively (Table 1). The 454 sequence reads were assembled into contigs and placed into scaffolds by the Newbler assembler with paired-end information from the Sanger reads. The assembled P131 genome consisted of 1,823 scaffolds with a combined length of 37.95 Mb. The N50 and maximum lengths of P131 scaffolds were 65 kb and 459 kb, respectively (Table 1). The Y34 genome was assembled into 1,198 scaffolds with a combined length of 38.87 Mb. The N50 and maximum length of Y34 scaffolds were 106 kb and 708 kb, respectively (Table 1). Over 95% of the sequence reads were assembled into scaffolds >5 kb in both isolates. Approximately 33% and 51% of P131 and Y34 sequences, respectively, were assembled into scaffolds longer than 100 kb. In addition, the mitochondrial genomes of P131 and Y34 were also assembled (Table 1). While P131 has an almost identical mitochondrial genome with 70-15, Y34 lacks two short fragments with a combined length shorter than 350 bp (Figure S2).

**Table 1:** Sequencing and genome analysis statistics for the genomes from three *Magnaporthe oryzae* isolates.

Features	P131	Y34	70-15
Reads from Sanger sequencing (Mb)	79.66	79.47	-
Reads from GS FLX sequencing (Mb)	314.4	406.7	-
Reads from GS FLX Titanium sequencing (Mb)	399.88	357.75	-
Total reads (Mb)	793.94	843.92	-
Coverage (fold)	20	21	-
Raw reads repeats content (%)	10.34	10.83	-
Scaffolds	1823	1198	-
Average scaffolds length (kb)	20.8	32.4	-
N50 scaffold length (kb)	12.3	11.6	-
Maximum scaffold length(kb)	459	708	-
Assembly size (Mb)^a^	37.95	38.87	41.70
Assembly size with A/C/T/G only (Mb)	37.549	38.242	37.499
G+C composition (%)	51.48	51.33	51.64
Coding region of assembly (%)	45.28	44.89	45.04
Number of predicted genes	12722	12869	12440
Average gene length (amino acids)	444.8	443.9	451.6
Average G+C composition of genes (%)	57.62	57.59	57.70
Mitochondrion (kb)	34.87	34.52	34.87

Because repetitive sequences comprise approximately 10% of the genome of the laboratory strain 70-15 (version 6), repetitive sequences in the new assemblies were masked out with the RepeatMasker program for comparative analyses. The resulting ATCG bases after masking were 37.6 Mb, 38.2 Mb, and 37.5 Mb, respectively, for P131, Y34, and 70-15 (Table 1), indicating that the core genomes of these three isolates were not significantly different in size. However, because repetitive sequences and singletons smaller than 2 kb were not included in this analysis, it remains possible that the complete genomes of these three isolates vary in abundance of repetitive sequences and actually have greater size differences.

**Figure 1:**
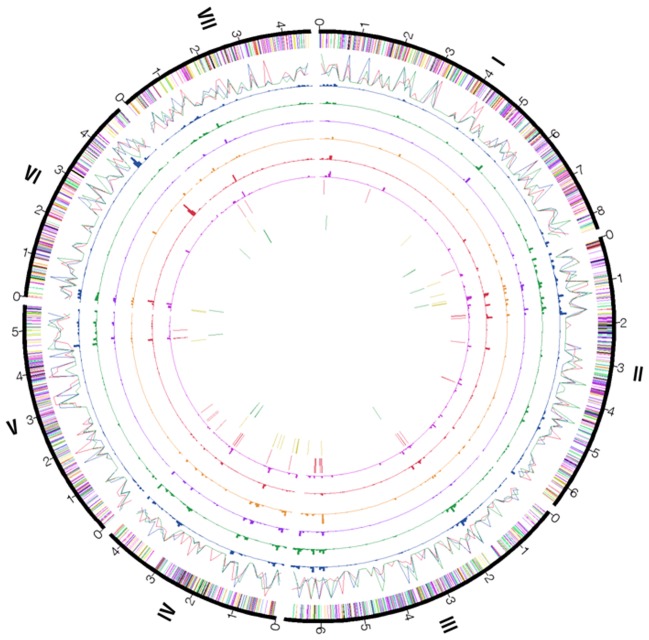
Genome organization and gene distribution in P131, Y34, and 70-15. The peripheral circle represents seven chromosomes (numbered I–VII) of 70-15 with their sizes marked in Mb. The second circle of color bands shows the distribution of predicted genes categorized by gene ontology along the chromosomes: red, DNA metabolism; blue, cellular component organization; yellow, carbohydrate metabolism; orange, amino acid and lipid metabolism; violet, transcription; black, signal transduction; green, transport. The third circle of color lines shows the distribution of repetitive DNA elements (percentages of repetitive sequences in 100-kb window) in 70-15 (red), Y34 (blue), and P131 (green). The fourth to ninth circles show the percentage of isolate-specific sequences from pair-wise comparisons in 50-kb windows: 4^th^, 70-15 sequences absent in P131; 5^th^, 70-15 sequences absent in Y34; 6^th^, P131 sequences absent in 70-15; 7^th^, P131 sequences absent in Y34; 8^th^, Y34 sequences absent in 70-15; 9^th^, Y34 sequences absent in P131. Genes unique to 70-15, P131, and Y34 are displayed on the tenth to twelfth circles, respectively, with the same color code as the second circle.

Scaffolds of P131 and Y34 were aligned with the assembled genome of 70-15 (Figure 1). Overall, most of the 70-15 genome (96%) is also conserved in two field isolates. Only 0.45 Mb of sequence in 70-15 are absent from the two field isolates. In contrast, P131 and Y34 have 1.69 Mb and 2.56 Mb isolate-specific sequences, respectively. In general, isolate-specific sequences were dispersed throughout the genomes. For individual chromosomes, there are regions enriched for isolate-specific sequences (Figure 1). Blocks of such sequences can be found at both ends of chromosome IV and at single ends of chromosomes I, II, III, V, and VI. In *M. oryzae*, genetic variation and avirulence genes are known to be enriched near the telomeres [18], [19]. Comparative analysis of the genomes of these three *M. oryzae* isolates revealed that genes responsible for variations in virulence and adaptation to the environment may be concentrated at the chromosomal ends.

**Figure 2:**
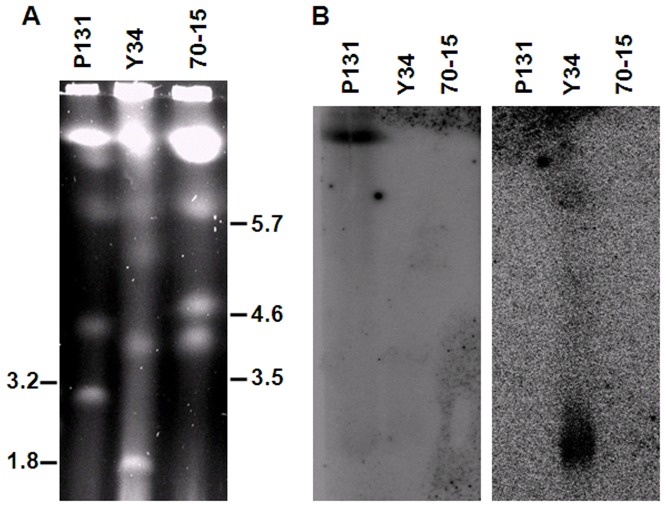
Electrokaryotypes of P131, Y34, and 70-15 and Southern blot analyses. (A) Chromosomes of P131, Y34, and 70-15 were separated by CHEF electrophoresis. Molecular weights of the markers (chromosomes of *Hansenula wingei* and *Schizosaccharomyces pombe*) are labeled on the sides (in Mb). (B) Southern blots of CHEF gels hybridized with the P131- and Y34-unique sequences as the probes. When hybridized with *P131_scaffold00006_11*, the largest chromosome band was detected only in P131 (left panel). When probed with *Y34_scaffold00824_1665*, only the smallest chromosome of Y34 was detected (right panel).

To locate and verify isolate-specific sequences in the field isolates, we used clamped homogenous electric fields (CHEF) gel electrophoresis to separate the chromosomes. Chromosome size polymorphisms were observed among these three isolates (Figure 2A). Whereas chromosome VII (the smallest chromosome) in 70-15 was estimated to be 4.3 Mb, the smallest chromosomes in Y34 and P131 were approximately 1.8 Mb and 2.5 Mb, respectively. When one P131-specific sequence, *P131_scaffold00006_11*, which was not mapped on the chromosome alignment was used as the probe, an aggregate band of chromosomes larger than 6.0 Mb was detected in P131 but not in Y34 nor in 70-15 (Figure 2B). When a similar blot was probed with an Y34-specific sequence, *Y34_scaffold00824_1665*, only the smallest chromosome of Y34 was hybridized (Figure 2B). These findings confirm that the field isolates contain isolate-specific DNA.

### Improvement of the 70-15 assembly

Because the assembly of P131 or Y34 relied on the alignment with the 70-15 genome, it was not possible to accurately map P131 and Y34 sequences that were absent from the 70-15 genome assembly. However, the P131 and Y34 sequences could be used to fill the sequence gaps (≥50 bp) in the 70-15 assembly. We identified the end sequences of the contigs or scaffolds flanking these gaps. After filtering out simple repeats, these sequences were used to search against the assembled P131 and Y34 sequences. If both upstream and downstream flanking sequences of one gap were mapped on the same contig in either P131 or Y34, the in-between sequences were used to fill the gaps of 70-15. A total of 55 gaps were filled with sequences from P131 or Y34 (Table 2). Among them, 35 gaps had the sequences present in both P131 and Y34 (Table 2). The total gap sequence filled in the 70-15 genome was 25.3 kb. We randomly selected 18 of these filled gaps of the 70-15 genome for verification. All of them were confirmed in 70-15 by PCR (Figure S3).

**Table 2:** Gaps in the genome of isolate 70-15 filled with sequences from the field isolates P131 and/or Y34.

70-15 chromosome	P131	Y34	P131+Y34
I	1	1	6
II	-	3	11
III	2	3	4
IV	-	3	8
V	-	-	1
VI	-	1	-
VII	1	-	1
unknown	4	1	4
Total	8	12	35

### Gene pool analysis and improvement of gene prediction in 70-15

**Figure 3:**
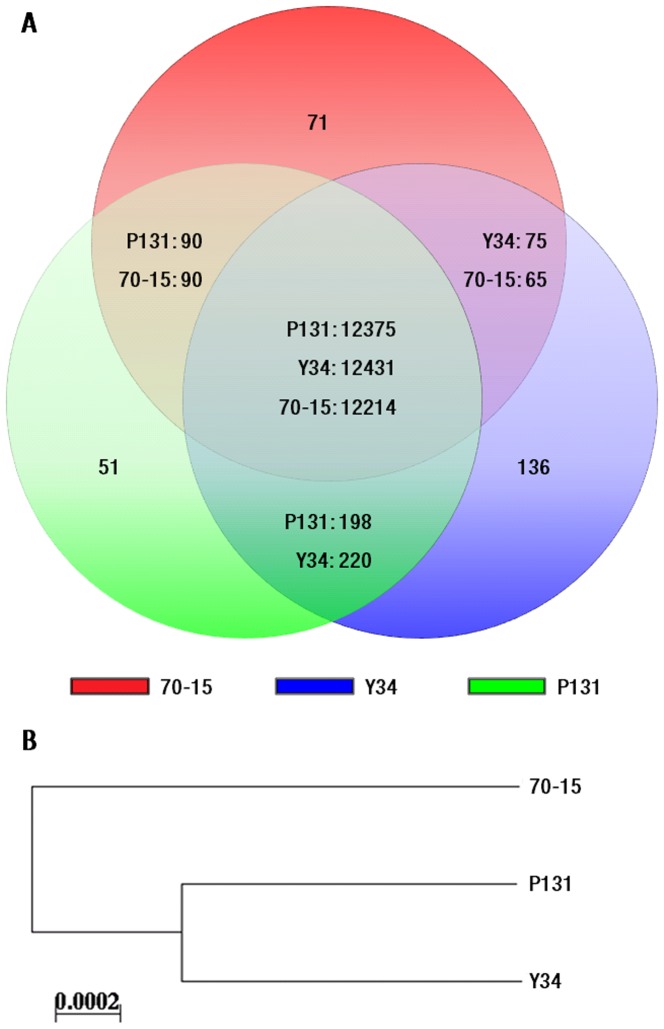
Gene pool analysis. (A) Numbers of the genes that are unique to each isolate, specific to two isolates, and common to all three isolates. Predicted genes of P131, Y34, and 70-15 are represented with circles colored in green, blue, and red, respectively. (B) Distance analysis with concatenated amino acid sequences of orthologous proteins that are conserved in P131, Y34, and 70-15. The tree was constructed with the NEIGHBOR program using the distance matrix calculated by PROTDIST from PHYLIP.

The number of predicted genes in the masked genomes of P131, Y34, and 70-15 was 12,714, 12,862, and 12,440 (Table 1), respectively. The average length of predicted proteins was over 400 amino acids. Y34 apparently has the largest genome size and gene content, which may contribute to its adaptation to the environment or to rice cultivars grown in Yunnan province, China. To identify the gene pool of these three strains, the predicted amino acid sequences of the total gene set from each isolate were used to search against the nucleotide sequences of other two isolates by TBLASTN. The large majority of *M. oryzae* genes (12,375 from P131, 12,431 from Y34, and 12,214 genes from 70-15) share sequence homology in pair-wise comparisons. Among these genes constituting the ‘core’ gene set of the *M. oryzae* genome (Figure 3A), 11.3% had no orthologous sequences in other organisms. Moreover, approximately 10.1% of these *M. oryzae*-specific genes were predicted to encode secreted proteins.

To improve gene annotation in 70-15, we identified the genes that were common to all three isolates and had similar sizes (difference less than 1%) between Y34 and P131 but were 50 amino acids or 20% longer or shorter in 70-15. A total of 340 genes meeting these criteria were then manually annotated. Among them, 135 genes in 70-15 had incorrect intron annotations. The number of genes with inaccurate start or stop codon predictions was 259 or 15, respectively (Table S2).

The number of genes shared only by the two field isolates (198 from P131 and 220 from Y34) was approximately twice that of those shared by either P131 or Y34 with 70-15 (Figure 3A), implying that the two Asian field isolates share a higher degree of similarity and with about 200 genes that are absent in 70-15. For isolate-specific genes, we found that 51, 136, and 71 genes were unique to P131, Y34, and 70-15, respectively (Figure 3A). All the genes randomly selected for verification were confirmed by PCR to be either shared by two isolates or unique to one specific isolate (Figure S4). As found in 70-15, isolates P131 and Y34 also had various copies of DNA helicase Q genes and LTR elements towards the chromosomal ends [3].

For the genes common in Y34 and P131 but absent in the automated annotation of 70-15, we used their amino acid sequences to search the 70-15 scaffolds. The resulting homologous sequences of 70-15 were then used to search against *M. oryzae* ESTs deposited in GenBank. A total of 81 candidate genes were identified in the 70-15 genome and ESTs (Table S3). Seventy-six of them encoded hypothetical proteins with no known homologs in GenBank. Some of these *M. oryzae* specific genes may be important for the virulence or fitness of the pathogen because all three isolates have these genes. The other five genes had orthologous sequences of unknown functions in Sordariomycetes but were absent in lower fungi, such as Zygomycetes and Saccharomycetales.

To further analyze genetic relatedness of these three isolates, the 10,074 clusters containing one protein from one isolate were selected and the resulting individual protein sequences from each isolate were combined for distance analysis with PHYLIP. As shown in Figure 3B, the two field isolates have a closer relationship to each other than with the laboratory strain 70-15.

### Isolate-specific genes

Based on analyses of gene content, 51, 136, and 71 genes, respectively, were unique to P131, Y34, and 70-15. Overall, 13% of these isolate-specific genes encoded secreted proteins and 46% of them had no significant homolog in GenBank (Table S4). RT-PCR analyses were performed with 10 and 14 randomly selected P131- and Y34-specific genes, respectively. All the selected genes were confirmed to be expressed in mycelia (Figure S5). While most of the isolate-specific genes were dispersed through the genome, some were located within clusters (Figure 1; Table S4). For example, scaffolds 00875 and 01112 of Y34 contained five and eight of the Y34-specific genes, respectively. In P131, there were three isolate-specific genes each on scaffolds P131_scaffold01777 and P131_scaffold01784. Moreover, many of the isolate-specific genes with known chromosomal positions in P131 and Y34 were located near the chromosomal ends (within 500 kb), which is consistent with the distribution tendency of isolate-specific sequences (Figure 1).

**Figure 4:**
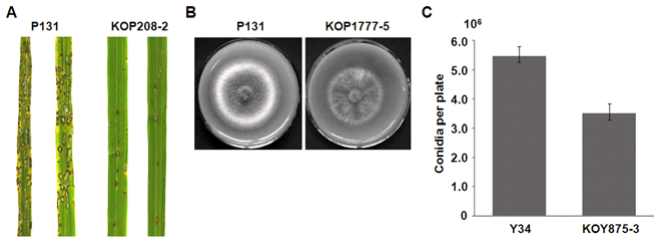
Functional analyses of isolate-specific genes. (A) Deletion of the P131-unique gene *P131_scaffold00208-2* resulted in reduced virulence. Rice seedlings were sprayed with conidia of the wild-type strain P131 and *P131_scaffold00208-2* deletion mutant KOP208-2. Representative leaves were photographed 7 days after inoculation (dai). (B) Deletion of the P131-unique gene *P131_scaffold01777-7* resulted in reduced colony growth. OTA plate cultures of P131 and the *P131_scaffold01777-7* deletion mutant KOP1777-5 were photographed after incubation for 5 days. (C) Deletion of the Y34-unique gene *Y34_scaffold00875-3* resulted in reduced conidiation. Conidiation was measured after incubation for 4 days.

To determine the biological function of these isolate-specific genes, nine Y34-specific genes and three P131-specific genes were selected for functional characterization. For majority of them, the resulting gene deletion mutants had no obvious changes in colony growth, conidiation, or virulence (Figure S6). Their functions in plant infection may be redundant or too minor to be detected under laboratory conditions. However, deletion of one P131 unique gene, *P131_scaffold00208-2*, resulted in a reduction in virulence in infection assays with seedlings of a susceptible rice cultivar (Figure 4A). Deletion of another P131 unique gene, *P131_scaffold01777-7*, resulted in approximately 10% growth reduction on oatmeal tomato agar plates (Figure 4B). Proteins encoded by these two P131-unique genes were predicted to be localized in the nucleus. Homologous sequences of these two genes were not found in other sequenced fungal species. Moreover, deletion of one Y34 unique gene, *Y34_scaffold00875-3*, resulted in approximately 36% reduction in conidiation (Figure 4C). Interestingly, deletion of one Y34-unique gene encoding a putative G protein-coupled receptor (GPCR)-like integral membrane protein with six transmembrane domains resulted in changes in pathogenicity on a rice cultivar carrying the *Pi-7* R gene, suggesting that this Y34-unique gene might be the potential *AVR Pi-7* gene (data not shown).

Among the genes shared by both field isolates P131 and Y34 but absent in 70-15, 19% had signal peptides for secretion and 12% had transmembrane domains (Figure 3; Table S5). About 70% of these genes had no functional annotation. Strain 70-15 may have lost these genes during the initial genetic cross or after generations of cultivation in the laboratory. For example, a gene encoding a CFEM-containing GPCR-like protein [20] and the avirulence gene *AVR Pi-a* [21] were present in the field isolates P131 and Y34 but not found in 70-15.

### Duplicated genomic sequences

Duplication is one of the major mechanisms for evolutionary innovation. The total duplicated genomic DNA fragments (longer than 500 bp and greater than 90% identity) were 289 kb, 385 kb, and 825 kb in P131, Y34, and 70-15, respectively. A total of 16, 20, and 155 predicted genes in P131, Y34, and 70-15, respectively, were located in these duplicated sequences (Table S6).

**Figure 5:**
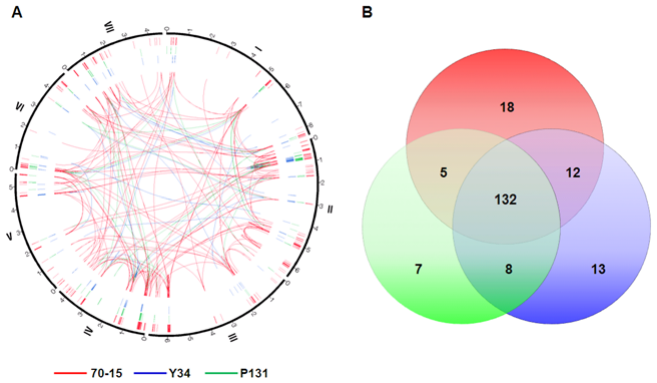
Genomic DNA duplication and gene family expansions. (A) Distribution of duplicated genomic sequences on chromosomes of P131, Y34, and 70-15. The green, blue, and red lines represent duplicated sequences in P131, Y34, and 70-15, respectively. Intra- and inter-chromosomal duplications are marked with lines in the center. (B) Venn diagram showing expanded gene families in P131 (green), Y34 (blue), and 70-15 (red). The numbers in the circles represent different sets of gene families that are isolate-specific, shared by any two isolates, and common to all three isolates, respectively.

Although duplicated DNA sequences were detected genome-wide in all three isolates, in general chromosomes II, IV, V, and VII had more duplicated DNA sequences than other chromosomes (Figure 5A). For individual chromosomes, the end regions tend to contain more duplicated DNA sequences than the central region. Comparative analysis indicated that P131, Y34, and 70-15 all contained isolate-specific duplicated regions (Figure 5A). However, the laboratory strain 70-15 had significantly more duplicated genes, including the *AVR* gene *PWL2* [22] (Table S6). Other duplicated genes with known functions include LPS glycosyltransferases, MFS transporters, sugar transporters, and carboxypeptidases. Both intra- and inter-chromosomal duplications were observed, but more inter-chromosomal duplications were apparent, and only a small portion of duplication events were conserved in all three isolates (Figure 5A).

### Gene families

To identify gene families, the entire set of the predicted proteins from all three isolates were clustered with the OrthoMCL program. A total of 38,016 proteins were grouped into 14,189 clusters with each cluster representing a group of putative orthologs. Among these clusters, 195 gene families were identified with more than one member in at least one isolate (Figure 5B), suggesting that 1.37% of the *M. oryzae* genes may have been evolved by gene family expansion. Among 45 clustered loci duplicated equally in each isolate, 38, 6, and 1 gene loci were duplicated between two, three or four times, respectively, per isolate (Table S7). These gene families might have existed before the divergence of the three isolates. The majority of these gene families were predicted to be involved in synthesis and transport of nutrition and secondary metabolites, suggesting that they may be related to plant infection (Table S7). There were 87 clustered loci duplicated at different frequencies in three isolates (Table S8). Most of these gene families (61 out of 87) contained duplicated genes in only one isolate, and 17 gene families contained gene loci duplicated at least three times in one or more isolates (Table S8), suggesting that they have been expanded or contracted in different strains, possibly during environmental adaptations. For example, one putative calcium P-type ATPase gene was duplicated three times in P131 and Y34, and twice in 70-15. Members of this gene family have been demonstrated to be required for disease development and induction of host resistance [23], [24].

For loci duplicated in two isolates but absent in the third one, there were eight in P131 and Y34, five in P131 and 70-15, and twelve in Y34 and 70-15 (Figure 5B; Table S9). Most of these expanded gene families had unknown functions. To confirm the duplication events that were unique to the two field isolates, three genes were selected by Southern blot analysis. All of them were confirmed to be specifically duplicated in P131 and Y34 but not in 70-15 (Figure S7). There were seven, thirteen, and eighteen gene families specifically expanded in P131, Y34, and 70-15, respectively (Figure 5B; Table S10). Most of these isolate-specific gene families contained two or three duplicated members that had unknown functions or no known homologs in GenBank.

### Asynonymous to synonymous nucleotide substitution rate (Ka/Ks) analysis

To analyze asynonymous and synonymous nucleotide substitutions, we first identified and removed orthologous genes with large deletions or insertions in any of the isolates from the list of common genes. In total, 9,184 highly conserved orthologs were used to identify nucleotide substitution events. Among them, 7,569 genes had neither synonymous nor asynonymous nucleotide substitution in pair-wise comparisons, indicating that most of the genes were well-conserved among different isolates. Only 428 genes had nucleotide substitutions between P131 and Y34, and 1,651 genes had nucleotide substitutions between 70-15 and P131 or Y34, further indicating that the field isolates had closer relationship with each other than with the laboratory strain. Genes with substitutions in the 70-15 versus P131/Y34 comparison could be categorized into four groups: 414 genes only with synonymous nucleotide substitutions, 697 genes only with asynonymous nucleotide substitutions, 124 genes with Ka/Ks<1, and 6 genes with Ka/Ks>1.

Overall, similar numbers of genes identical between Y34 and P131 but with nucleotide variations in 70-15 were thought to have undergone diversifying versus purifying selections. However, several functional categories of genes, such as those involved in cellular responses to stimuli and organophosphate metabolisms, had more members exhibiting diversifying selection in the two field isolates (Table S11). Several of the genes underwent diversifying selection in the 70-15 versus P131/Y34 comparison (Table S12), including *ATG4*, *HEX1*, *MCK1*, *MoSNF1*, *PTH2*, and *RGS1*, which are known virulence factors in *M. oryzae* [25]–[30]. Three of them encode putative CFEM-domain receptors that may be involved in recognizing different environmental and plant signals (Table S12).

### Repetitive sequences and transposable elements

Repetitive sequences were masked by Newbler for assembling 454 sequence data of P131 and Y34. To compare repetitive sequences of these two isolates, we assembled the Sanger reads of P131 and Y34 (approximately 2-fold genome coverage) and found that 10.8%, 10.3%, and 10.6% of the 70-15, P131, and Y34 genomes, respectively, were repetitive sequences, indicating that the abundance of repetitive sequences is similar among these three isolates. Transposable elements (TE) and their insertion sites (flanking sequences) were identified by RepeatMasker. Although the exact copy numbers vary, both field isolates contained all classes of transposable elements identified in 70-15 (Table 3). In general, 70-15 has more members of the LINE, Maggy, and RETRO5 LTR retrotransposons. The Pot2/Pot4 DNA transposons and the Pyret and Grasshopper LTR retrotransposons were more abundant in P131 and Y34. In addition, nine new clusters of repetitive sequences were identified by analysis with RepeatScout (Table 3). However, none of them was unique to the field isolates. While clusters 1, 4, 5, 6, and 7 were much more abundant in the field isolates, 70-15 had more copies of the cluster 2 repetitive elements (Table 3).

**Table 3:** Repetitive and transposable elements identified in isolates P131, Y34, and 70-15.

	70-15	Y34	P131	70-15/P131^a^	70-15/Y34	P131/Y34
**DNA transposon:**						
Pot2/Pot4	277	405	341	109	131	135
Occan	77	93	65	44	61	35
Pot3	68	85	79	29	25	27
**LTR retrotransposon:**						
Maggy	267	60	64	20	23	20
MGLR3	81	74	50	30	35	27
Pyret	232	471	425	160	188	128
RETRO5	329	103	86	21	21	37
RETRO6	107	108	85	47	40	42
RETRO7	119	103	102	51	42	47
Grasshopper	13	42	34	14	10	16
**LINE:**						
MGL	187	141	127	58	53	52
**SINE:**						
Mg-SINE/Mg-MINE/Ch-SINE	176	195	179	64	58	57
**New repetitive elements:**						
cluster1 (JQ929664)^b^	25	54	52	33	32	31
cluster2 (JQ929665)	36	23	24	22	21	16
cluster3 (JQ929666)	78	86	78	58	60	45
cluster4 (JQ929667)	17	34	27	7	13	8
cluster5 (JQ929668)	47	58	63	47	43	47
cluster6 (JQ929669)	5	25	13	4	4	8
cluster7 (JQ929670)	22	43	30	14	17	15
cluster8 (JQ929671)	118	101	116	98	88	79
cluster9 (JQ929672)	16	18	15	14	15	12

a The number of conserved repetitive and transposable elements in the 70-15 and P131 comparison.

b GenBank accession number of new repetitive elements. The copy number of repetitive and transposable elements was calculated by RepeatMasker. LTR, long terminal repeat; LINE, long interspersed repeat element; SINE, short interspersed repeat element. Novel repetitive elements were identified by RepeatScout.

**Figure 6:**
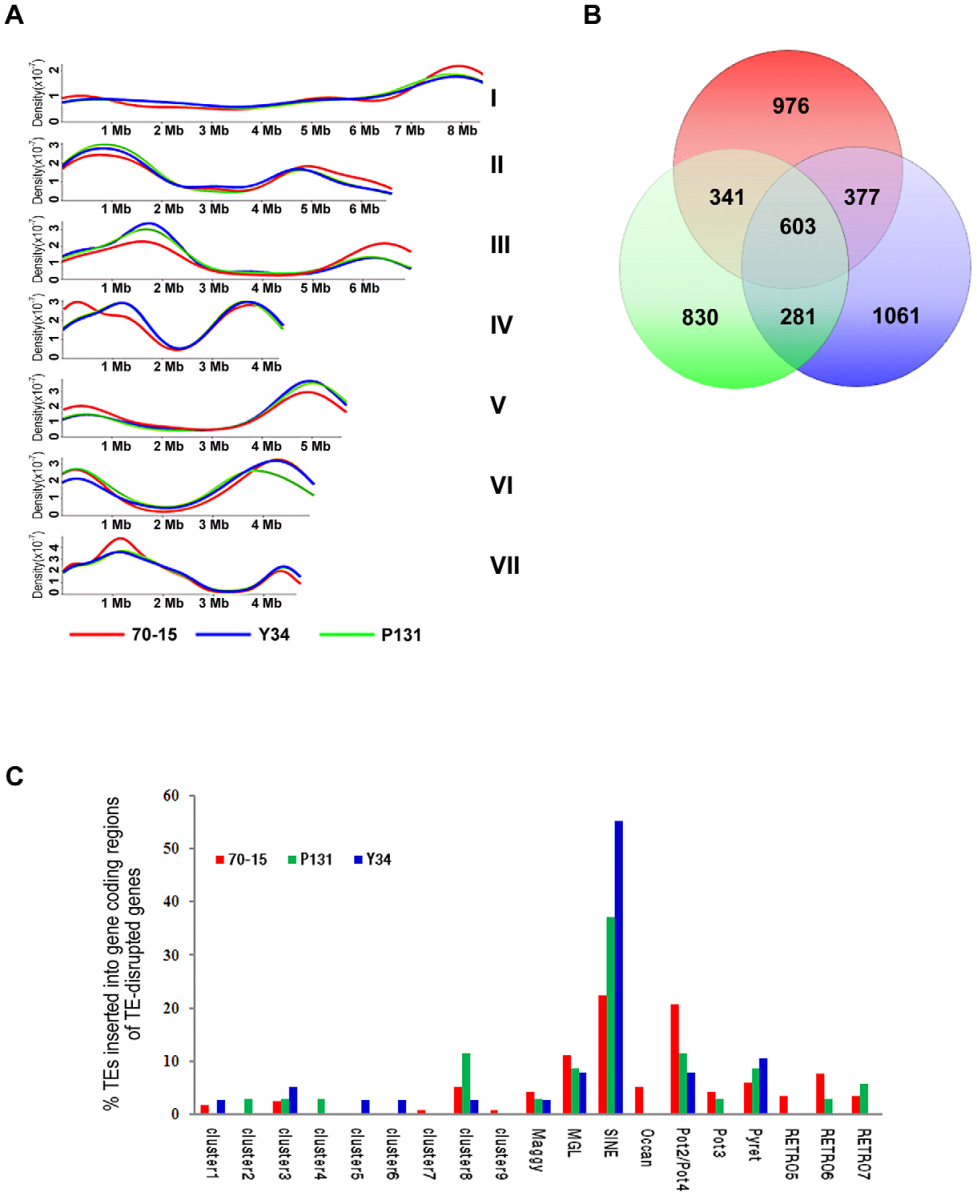
Transposable elements (TEs). (A) Distribution of TEs on seven chromosomes. The y-axis represents the density probability of TEs along the chromosome. (B) Venn diagram showing the number of TEs with conserved genomic positions in 70-15, Y34, and P131. (C) The percentage of each TE element inserted into gene coding regions of TE-disrupted genes. Red, 70-15; blue, Y34; green, P131.

In comparison with 70-15, the two field isolates were more similar in the distribution pattern of repetitive sequences (Figure 1 and 6A). While chromosomal ends tend to have more repetitive sequences, all three isolates had much reduced numbers of TEs in the gene-rich regions of chromosomes III, V, and VI (Figure 6A). For the TEs that could be assembled into the genome sequences, approximately 27% of them had the same locations in all three isolates by comparison of their flanking sequences (Figure 6B). Y34 had more TEs with unique chromosomal positions (1,061) than P131 (830) or 70-15 (976). In addition to the 603 locations of TEs conserved among the three strains, Y34 and P131 also shared 281 TEs with the same chromosomal locations, which was fewer than the 377 between 70-15 and Y34 or the 341 between 70-15 and P131 (Figure 6B). While over two-thirds of the members of some TEs, including Occan, had conserved genomic locations, TEs such as Retro5 and Maggy differed significantly in their chromosomal positions between Y34 and 70-15. Similar results were obtained with the P131 and 70-15 comparison (Table 3). A total of 41.1% and 46.0% of TEs in 70-15 and P131, respectively, had conserved genomic locations. The Pot3, Maggy, Retro5, and Retro7 elements had the highest variation in chromosomal positions between 70-15 and P131.

We also analyzed the impact of TEs on the genome evolution by comparing two-fold coverage Sanger data of P131 and Y34 with the 70-15 assembly. A total of 35, 38, and 116 genes were disrupted by the insertion of TEs in P131, Y34, and 70-15, respectively (Table S13, S14, S15). Over 50% of the gene disruption events were caused by TEs belonging to MGL, Mg-SINE, or Pot2/Pot4. Strain 70-15 had a number of genes disrupted by cluster 7, cluster 9, Occan, and RETRO5 elements, which were not observed in P131 or Y34 (Figure 6C). Some of these genes may have been disrupted by transposition events occurring during generations of cultivation under laboratory conditions, and these genes may play roles in plant infection or survival in the field isolates but were not required for the laboratory isolate. In comparison with 70-15, the field isolates P131 and Y34 had more genes disrupted by SINE (Figure 6C), which may indicate that these SINE elements were more active in these two field isolates.

Among all the genes disrupted by TEs in three isolates, only approximately one third of them have known functions based on their orthologs in GenBank, and most of them are involved in protein metabolism, transportation, transcription, or lipid metabolism. The majority of the TE-disrupted genes encode hypothetical proteins with unknown functions. Interestingly, 23.8% of them contained putative signal peptide sequences, which is significantly higher than the average percentage of predicted extracellular proteins in the genomes of these three strains (Table S13, S14, S15). Some of them may function as effectors involved in fungal-plant interactions, such as *AVR Pi-ta1* in 70-15 (Table S13). In addition, 14.7%, 14.2% and 15.8% of the TE-disrupted genes in 70-15, P131, and Y34, respectively, encoded proteins with putative nuclear localization sequences.

Intriguingly, the regions containing isolate-specific sequences or duplicated genes families were often near areas with high frequency of TEs (Figure S8). In 70-15, several TEs were found within 1.0 kb from 23 duplicated genes families, including the avirulence gene *PWL2* (Table S16) although many of these duplicated sequences were not closely linked or located on different chromosomes. Taken together, it is likely that the transposition events of TEs might be related to translocation of duplicated DNA fragments and presence of isolate-unique sequences in these three strains.

## Discussion

In a number of eukaryotic organisms, comparative analysis of multiple genomes of the same species has been used to improve assembly and annotation and to identify genome variations [31]–[34]. The rice blast fungus is well-known for its natural genetic variation [1], [2]. In this study, we sequenced two field isolates of *M. oryzae* from Asia. Genome analysis indicated that these two field isolates are more closely related to each other than to 70-15, which is a laboratory strain derived from three backcrosses of rice pathogen Guy11 with a progeny of a cross involving a weeping love grass pathogen, and maintained for many years under laboratory conditions. The overall genome content and composition are similar among these three isolates, but the genomes of P131 and Y34 with only A/C/T/G and no N's were slightly larger than that of 70-15.

Although the 70-15 genome has been updated several times, it still has many gaps (www.broadinstitute.org/annotation/genome/magnaporthe_grisea). In this study, a total of 55 gaps of the 70-15 genome (version 6) were filled in with sequences from P131 and Y34, and the results were validated by PCR analyses of 70-15. This number of putative filled gaps with sequences from two isolates may seem low, but because of the short read length, the threshold set may have been too stringent. For 35 gaps, they were filled with consensus sequences found in both field isolates. For the gaps with sequences only available in either Y34 or P131, the filling sequence for 70-15 was less certain, but of high probability because the overall nucleotide sequence identity between 70-15 with P131 or Y34 was over 98%. Besides improving the genome assembly, the sequences of P131 and Y34 were used to improve the annotation of 70-15. We identified 81 genes that were not predicted in the automated annotation of the 70-15 genome sequence, and none of them were related to the sequence gaps. In addition, we identified potential annotation errors in 340 predicted genes of 70-15. Most of them were related to the problems with the prediction of the boundaries of introns and start or stop codons.

Our study revealed that each *M. oryzae* isolate had some unique genomic DNA sequences. Because genome sequences of P131 and Y34 were aligned with that of 70-15, it was impossible to locate most of the sequences unique to Y34 and P131 onto specific chromosomes or chromosomal regions. However, sequences unique to 70-15 were distributed over all seven chromosomes. Because 70-15 was derived from three backcrosses of rice pathogen Guy11 with a progeny of a cross involving a weeping love grass pathogen, we expected that a small portion of its genome was from the weeping love grass pathogen. The isolates Y34, P131, and 70-15 had 136, 51, and 71 unique genes, respectively. Therefore, less than 1% of the predicted genes were unique to each isolate and these genes play diverse roles, some of which might possibly contribute to the specificity of individual isolates. Some of the isolate-specific genes were clustered, suggesting that isolate-specific DNA fragments might be gained or lost during evolution. The P131-specific gene *P131_scaffold00208-2* encoded a hypothetical protein without known homologs in other fungi. Deletion of this gene resulted in reduced virulence toward rice plants. Because it might be involved in plant infection, *P131_scaffold00208-2* may play an isolate-specific role in suppressing or overcoming plant defense responses. These results suggest that some of the field isolate-specific genes may play important roles in plant infection.

In all three *M. oryzae* isolates, most of the duplicated genes are functionally unknown. Duplicated sequences are distributed all over seven chromosomes and appear to be enriched in the telomeric regions. For the duplicated genes with known functions, many of them are predicted to be involved in primary and secondary metabolism and interactions with the host (such as cutinases and Avr proteins), which is consistent with earlier observations with 70-15 [3]. Interestingly, several gene families involved in synthesis and transport of nutrients and secondary metabolites were expanded with different frequencies in these three isolates. Some of these duplicated genes may contribute to the adaption of *M. oryzae* to different environmental conditions.

Among the genes that had undergone diversifying selection in Y34 and P131 in comparison with 70-15, a number of them are known to be important for virulence, suggesting that such genes may have been under strong selection pressure in their natural field environments. There were six genes under positive selection in the two field isolates compared to 70-15. Two of them encoded two hypothetical proteins, a serine/threonine protein kinase, an acyltransferase, a putative catalytic domain of diacylglycerol kinase, and an aspartic-type endopeptidase. Three of them are located on chromosome I. In contrast, there were no genes showing positive selection in the comparison between the field isolates.

Because sexual reproduction has not been observed in the field, it is possible that translocations of the repetitive sequences may be one of the major sources for genome variation and rapid adaption to different host and environmental conditions. Consistent with this hypothesis, over 10% of the genome sequences were found to be repetitive sequences. In addition to TEs that have been identified in previous studies [3], nine new clusters of repetitive sequences were identified in all three *M. oryzae* strains in this study. Most of these TEs have different copy numbers in different isolates. Strikingly, among thousands of TE loci, less than 30% of them were conserved among these isolates, suggesting active transposition of these TEs in *M. oryzae*. Moreover, approximately 200 genes were totally disrupted by TEs in these three strains, and approximately 40% of them encoded extracellular or nuclear proteins, suggesting that transpositions of TEs may contribute to variations in host-microbe interactions and transcriptional regulation. Interestingly, TEs tended to be found near isolate-specific sequences and duplicated DNA fragments. It is possible that translocation of TEs is important for gain or loss of isolate-specific sequences and gene duplication events.

Overall, our results indicate that gain or loss of unique genes, duplications, gene family expansions, and translocations of TEs can be important factors for genome variation in the rice blast fungus. Among these factors, translocation of TEs may be the most important one because of its association with gene duplication and isolate-specific sequences. There are reports on comparative genomic analyses of plant pathogenic oomycetes and fungi, such as *Phytophthora* and *Fusarium* species [35], [36]. However, to our knowledge, this study is the first on comparative analysis of the field and laboratory strains of a plant pathogenic fungus, and this can give insights into the genome variations of the fungus under different environments.

## Materials and Methods

### Sequencing and genome assembly

For Sanger sequencing, genomic libraries with insertion size of 1.5 kb to 3.5 kb were constructed and sequenced at the Beijing Genomic Institute (BGI, Beijing, China). These two isolates were also sequenced with the GS-FLX and GS-FLX Titanium 454 platforms [37] at BGI that generated reads with an average length of 240- and 380-bp, respectively. Reads from Sanger and 454 sequencing were placed into scaffolds using the Newbler assembler (version 1.1.02.15, Roche).

The *M. oryzae* 70-15 genome sequence version 6 was downloaded from the Broad Institute (www.broad.mit.edu/annotation/genome/magnaporthe_grisea). The repetitive sequences in the assembled genomes of laboratory strain 70-15 and the field isolates P131 and Y34 were masked with RepeatMasker (Smit, AFA, Hubley, R & Green, P. *RepeatMasker Open-3.0* at http://repeatmasker.org). Masked genome sequences of the three *M. oryzae* isolates were compared with the MUMMER package [38] to construct chromosome sequences for P131 and Y34 based on 70-15 data. Genomic sequences with nucleotide identity over 92% were considered to be conserved among different isolates.

### Gene prediction and annotation

*De novo* gene prediction of the P131 and Y34 genome sequences was performed with FGENESH [39], which was trained with 79 gene models of *M. oryzae* (kindly provided by Prof. Zhen Su at China Agricultural University). The tRNA genes were identified by tRNAscan [40]. Gene functions were predicted by comparison with the NCBI NR protein database (http://www.ncbi.nlm.nih.gov/) and the Pfam database [41]. InterPro [42] was used for gene ontology annotations. Membrane and sub-cellular localization were predicted by TMHMM 2.0 [43], SignalP3.0 [44], and WoLF PSORT [45].

### Gene pool and distance analyses

Nucleotide sequences of the predicted genes of P131, Y34, or 70-15 were compared separately with genomic sequences of the other two isolates with TBLASTN [46]. Homologous genes with sequence identities of 100%, 80–100%, and 50–80% were defined as identical, similar, and divergent, respectively, while those below 50% were considered non-homologous. Sequences of genes unique to the field isolates were also queried against the unassembled reads of 70-15. Orthologous proteins were clustered with OrthoMCL [47]. Only the clusters containing one protein from each isolate were selected for distance analysis. Individual protein sequences from three isolates were concatenated and aligned with T-Coffee [48], and a distance matrix was calculated with PROTDIST from the PHYLIP package [49]. Finally, a neighbor-joining tree was constructed with NEIGHBOR from the PHYLIP package.

### Ka/Ks analysis

The coding sequences of orthologous genes conserved in all three isolates were aligned with ClustalW [50] to detect large deletions (>12-bp), frame shifts, and null mutations. Orthologous genes without large deletions, frame shifts, or null mutations in the open read frame were analyzed for Ks and Ka with the YN00 program in the PAML package [51].

### Analysis of repetitive sequences and transposable elements

The Sanger reads of P131 and Y34 were assembled with RePS [52] and analyzed for transposable elements with RepeatMasker. New repetitive elements were identified by RepeatScout [53]. For each transposable element (TE) identified in P131 or Y34, its flanking sequences of 30 to 100 bp were extracted and used to search against the 70-15 genome with Standalone BLASTN (e-value<10^−5^). Each TE and its corresponding region in 70-15 genome were aligned with BLAST2seq to assess whether it was conserved. To search for genes disrupted by TEs, unique flanking sequences of TEs in P131 or Y34 were used to search against 70-15 genes (e-value<10^−20^). The search results were removed if more than one hit was found. Similar analyses were performed with P131 and Y34.

### Culture conditions and plant infection assays

The wild-type and mutant strains of 70-15, P131, and Y34 were cultured at 25°C on oatmeal tomato agar (OTA) plates and conidiation assessed [17]. Mycelia collected from two-day-old cultures in complete media (CM) shaken at 150 rpm were used for extraction of fungal DNA and protoplasts. Media were supplemented with 250 µg/ml hygromycin B (Roche, USA) or 400 µg/ml neomycin (Amresco, USA) to select hygromycin-resistant or neomycin-resistant transformants. Four-week-old seedlings of monogenic rice cultivars (Table S1) and eight-day-old seedlings of barley cultivar ‘E8’ were inoculated as previously described [17], [54]. Lesion development was examined 5–7 days after inoculation.

### CHEF electrophoresis

Chromosome-size DNA were prepared with protoplasts isolated from vegetative hyphae as previously described [55], [56], and separated on 0.65% Megabase agarose (Bio-Rad, USA) gels with a Bio-Rad DR III system with switching intervals of 60 min for 48 h, 55 min for 72 h, 45 min for 72 h, and 35 min for 72 h at 1.5 V/cm. Chromosomal DNA of *Schizosaccharomyces pombe* and *Hansenula Wingei* (Bio-Rad, USA) were used as the molecular weight markers.

### Generation of the gene replacement constructs and mutants

To generate the *P131_scaffold00208-2* gene replacement vector pKOPS208-2, its 0.97 kb upstream and 0.82 kb downstream fragments were amplified with primer pairs P131_scaffold00208-2KO_LBf plus P131_scaffold00208-2KO_LBr, and P131_scaffold00208-2KO_RBf plus P131_scaffold00208-2KO_RBr, respectively. The resulting PCR products were cloned into the *Kpn*I-*Hin*dIII and *Eco*RI-*Spe*I sites of pKOV21 [56], [57]. After linearization with *Not*I, pKOPS208-2 was introduced into protoplasts of P131. Hygromycin resistant transformants were isolated and assayed for neomycin-resistance. The resulting transformants were screened by primer pairs P1/P11 and P2/P12. The putative deletion mutants were identified and confirmed by Southern blot analysis. The same approach was used to generate gene replacement constructs and mutants for isolate-specific genes: *P131_scaffold00297-2*, *P131_scaffold00493-1*, *Y34_scaffold00875-1*, *Y34_scaffold00875-3*, *Y34_scaffold00857-6*, *Y34_scaffold01193-2*, *Y34_scaffold00005-1*, *Y34_scaffold01048-2*, *Y34_scaffold00105-1*, *Y34_scaffold00105-2*, and *Y34_scaffold00855-11*. The primer pairs used for generating the gene replacement constructs and for mutant screening are listed in Table S17.

### Accession number

The genome sequence data of Y34 and P131 were deposited in the NCBI Genome Database (www.ncbi.nlm.nih.gov/genome) under the accession numbers AHZS00000000 and AHZT00000000, respectively. The nucleotide sequence data of repetitive sequences and transposable elements are available in the NCBI GenBank database under the following accession numbers: M77661 for Grasshopper, AB024423 for Maggy, AF018033 for MGL, AJ851229 for Mg-MINE, AF314096 for MGRL3, MGU35313 for Mg-SINE, AB074754 for Occan, AF314096 for Pot2, AF333034 for Pot3, AB062507 for Pyret, NC_009594 for Pot4, RETRO5, RETRO6, RETRO7, and Ch-SINE, JQ929664 for Cluster 1, JQ929665 for Cluster 2, JQ929666 for Cluster 3, JQ929667 for Cluster 4, JQ929668 for Cluster 5, JQ929669 for Cluster 6, JQ929670 for Cluster 7, JQ929671 for Cluster 8, and JQ929672 for Cluster 9.

## Supporting Information

Figure S1 Comparison of the isolates P131, Y34, and 70-15 on asexual development and plant infection. (A) Colonies of P131, Y34, and 70-15 on OTA plates photographed at 120 hours after inoculation (hpi). (B) Vegetative mycelia of P131, Y34, and 70-15 shaken in liquid CM, photographed at 48 hpi. (C) Seedlings of the susceptible rice cultivar ‘LTH’ sprayed with conidia of P131, Y34, and 70-15, respectively, photographed 7 days after inoculation (dai).
(TIF)

Figure S2 The whole mitochondrial genomes of P131 or Y34 were compared with that of 70-15. The differences in nucleotide acid substitution or deletion among the three isolates are shown. The mitochondrial genome of Y34 lacks two fragments with a combined length shorter than 350 bp.
(TIF)

Figure S3 PCR validation of the gaps in the assembly of 70-15 filled with genome sequences of P131 and Y34. The genomic DNA of 70-15 was used for PCR amplifications with the primer pairs listed in Table S17.
(TIF)

Figure S4 PCR validation of the selected genes unique to single isolates P131 (A), Y34 (B), or 70-15 (C), and specific to two isolates P131 and Y34 (D), P131 and 70-15 (E), or P131 and Y34 (F). The genomic DNA of isolates P131, Y34, and 70-15 were used for PCR amplification with the primer pairs shown in Table S17.
(TIF)

Figure S5 RT-PCR validation of the isolate-specific genes unique to P131 or Y34. Primers listed in Table S17 were used to amplify sequences unique to P131 or Y34 with cDNA synthesized from RNA isolated from vegetative hyphae.
(TIF)

Figure S6 Functional analyses of 12 field isolate-specific genes. (A) Colonies of the wild-type strain Y34 and the null mutants of Y34-unique genes KOY875-1 (*Y34_scaffold00875-1*), KOY875-3 (*Y34_scaffold00875-3*), KOY857-6 (*Y34_scaffold00857-6*), KOY1193-2 (*Y34_scaffold01193-2*), KOY5-1 (*Y34_scaffold00005-1*), KOY1048-2 (*Y34_scaffold01048-2*), KOY105-1 (*Y34_scaffold00105-1*), KOY105-2 (*Y34_scaffold00105-2*), KOY855-11 (*Y34_scaffold00855-11*), and the wild-type strain P131 and the null mutants of P131-unique genes KOP208-2 (*P131_scaffold00208-2*) and KOP1784-1-2-3 (*P131_scaffold01784-1-2-3*). Representative photographs were taken on OTA plates 5 dai. (B) Barley seedlings sprayed with conidia of the wild-type strains P131 and Y34, and with null mutants of all 12 genes photographed 5 dai.
(TIF)

Figure S7 Verification of genes duplicated specifically to the field isolates. Genomic DNA of P131, Y34, and 70-15 were digested by two restriction enzymes. Amplified fragments of the gene *P131_scaffold01531-1*, *P131_scaffold01428-1*, and *Y34_scaffold00846-5* were used as probes. M, λ-*Hin*dIII ladder.
(TIF)

Figure S8 Co-distribution of the TEs with the duplicated genes families and isolate-specific sequences in the genomes of P131, Y34, and 70-15. The peripheral circle represents seven chromosomes (numbered I–VII) of 70-15 with their sizes marked in Mb. The 2^nd^ to 4^th^ circles represent duplicated genes families along seven chromosomes. The 5^th^ to 7^th^ circles represent the distribution of the TEs in the genomes of three isolates. The 8^th^ to 13^th^ circles represent the percentage of isolate-specific sequences from pair-wise comparisons in 50-kb windows same as the 3^rd^ to 8^th^ circles shown in Figure 1. Red, 70-15; blue, Y34; green, P131.
(TIF)

Table S1 Pathotypes of P131, Y34, and 70-15 based on their infectivity towards different monogenic rice cultivars generated by the International Rice Research Institute.
(DOC)

Table S2 Genes in 70-15 with potential annotation errors adjusted with data from the assembled genomes of P131 and Y34.
(DOC)

Table S3 Genes that were absent in 70-15 version 6 but identified by comparative analysis with the genomes of P131 and Y34, and compared against GenBank NR.
(DOC)

Table S4 Isolate-unique genes in P131, Y34, and 70-15.
(DOC)

Table S5 Genes specific to the field isolates P131 and Y34.
(DOC)

Table S6 Genes predicted within duplicated genomic fragments of P131, Y34, and 70-15.
(DOC)

Table S7 Gene families with the same number of members in all three isolates.
(DOC)

Table S8 Gene families with different numbers of members in each of the three isolates.
(DOC)

Table S9 Gene families specific to two of the three isolates.
(DOC)

Table S10 Isolate-specific gene families.
(DOC)

Table S11 The number of the genes identical between Y34 and P131 but with nucleotide variations in 70-15 and which showed evidence of exposure to diversifying and purifying selection pressures based on GO classification.
(DOC)

Table S12 Genes with only asynonymous nucleotide substitutions in field isolates P131 and Y34 compared with the laboratory strain 70-15.
(DOC)

Table S13 Genes of isolates P131 and Y34 mapped against chromosomal assembly of 70-15 and found to be disrupted by TE.
(DOC)

Table S14 Genes of isolates Y34 and 70-15 mapped against chromosomal assembly of P131 and found to be disrupted by TE.
(DOC)

Table S15 Genes of isolates P131 and 70-15 mapped against chromosomal assembly of Y34 and found to be disrupted by TE.
(DOC)

Table S16 Transposable elements located within 1.0 kb of members of the duplicated gene families in 70-15.
(DOC)

Table S17 PCR primers used in this study.
(DOC)

## References

[1] Valent B, Chumley FG (1991) Molecular genetic analysis of the rice blast fungus, *Magnaporthe grisea* . Annu Rev Phytopathol 29: 443–467. 18479196 10.1146/annurev.py.29.090191.002303

[2] Talbot NJ (2003) On the trail of a cereal killer: Exploring the biology of *Magnaporthe grisea* . Annu Rev Microbiol 57: 177–202. 14527276 10.1146/annurev.micro.57.030502.090957

[3] Dean RA, Talbot NJ, Ebbole DJ, Farman ML, Mitchell TK, et al (2005) The genome sequence of the rice blast fungus *Magnaporthe grisea* . Nature 434: 980–986. 15846337 10.1038/nature03449

[4] Kiyosawa S (1982) Genetics and epidemiological modeling of breakdown of plant disease resistance. Ann Rev Phytopathol 20: 93–117.

[5] Zeigler RS, Leong SA, Teng PS (1994) Rice blast disease. International Rice Research Institute, Wallingford, Oxon (United Kingdom).

[6] Orbach MJ, Farrall L, Sweigard JA, Chumley FG, Valent B (2000) A telomeric avirulence gene determines efficacy for the rice blast resistance gene *Pi-ta* . Plant Cell 12: 2019–2032. 11090206 10.1105/tpc.12.11.2019 PMC152363

[7] Levy M, Correavictoria FJ, Zeigler RS, Xu SZ, Hamer JE (1993) Genetic diversity of the rice blast fungus in a disease nursery in Colombia. Phytopathol 83: 1427–1433.

[8] Kumar J, Nelson RJ, Zeigler RS (1999) Population structure and dynamics of *Magnaporthe grisea* in the Indian Himalayas. Genetics 152: 971–984. 10388817 10.1093/genetics/152.3.971 PMC1460659

[9] Farman ML (2002) Meiotic deletion at the *BUF1* locus of the fungus *Magnaporthe grisea* is controlled by interaction with the homologous chromosome. Genetics 160: 137–148. 11805051 10.1093/genetics/160.1.137 PMC1461934

[10] Leung H, Borromeo ES, Bernardo MA, Notteghem JL (1988) Genetic analysis of virulence in the rice blast fungus *Magnaporthe grisea* . Phytopathol 78: 1227–1233.

[11] Chao C-CT, Ellingboe AH (1991) Selection for mating competence in *Magnaporthe grisea* pathogenic to rice. Can J Bot 69: 2130–2134.

[12] Xu JR, Zhao X, Dean RA (2007) From genes to genomes; a new paradigm for studying fungal pathogenesis in *Magnaporthe oryzae* . Adv Genet 57: 175–218. 17352905 10.1016/S0065-2660(06)57005-1

[13] Zheng F, Yang Q, Zhao Z, Li J (1998) Variability of pathogenicity of *Pyricularia oryzae* . J Yunnan Agric Univ 13: 20–24.

[14] Zeng Y, Li Z, Yang Z, Wang X, Shen S, et al (2001) Ecological and genetic diversity of rice germplasm in Yunnan, China. PGR Newslet 125: 24–28.

[15] Chen QH, Wang YC, Zheng XB (2006) Genetic diversity of *Magnaporthe grisea* in China as revealed by DNA fingerprint haplotypes and pathotypes. J Phytopathol 154: 361–369.

[16] Yamada M, Kiyosawa S, Yamaguchi T, Hirano T, Kobayashi T, et al (1976) Proposal of a new method for differentiating races of *Pyricularia oryzae* in Japan. Ann Phytopathol Society Japan 42: 216–219.

[17] Peng YL, Shishiyama J (1988) Temporal sequence of cytological events in rice leaves infected with *Pyricularia oryzae* . Can J Bot 66: 730–735.

[18] Farman ML, Kim YS (2005) Telomere hypervariability in *Magnaporthe oryzae* . Mol Plant Pathol 6: 287–298. 20565657 10.1111/j.1364-3703.2005.00285.x

[19] Rehmeyer C, Li W, Kusaba M, Kim YS, Brown D, et al (2006) Organization of chromosome ends in the rice blast fungus, *Magnaporthe oryzae* . Nucleic Acids Res 34: 4685–4701. 16963777 10.1093/nar/gkl588 PMC1635262

[20] Kulkarni RD, Kelkar HS, Dean RA (2003) An eight-cysteine-containing CFEM domain unique to a group of fungal membrane proteins. Trends Biochem Sci 28: 118–121. 12633989 10.1016/S0968-0004(03)00025-2

[21] Miki S, Matsui K, Kito H, Otsuka K, Ashizawa T, et al (2009) Molecular cloning and characterization of the *AVR-Pia* locus from a Japanese field isolate of *Magnaporthe oryzae* . Mol Plant Pathol 10: 361–374. 19400839 10.1111/j.1364-3703.2009.00534.x PMC6640357

[22] Sweigard JA, Carroll AM, Kang S, Farrall L, Chumley FG, et al (1995) Identification, cloning, and characterization of *PWL2*, a gene for host species-specificity in the rice blast fungus. Plant Cell 7: 1221–1233. 7549480 10.1105/tpc.7.8.1221 PMC160946

[23] Balhadère PV, Talbot NJ (2001) *PDE1* encodes a P-type ATPase involved in appressorium-mediated plant infection by the rice blast fungus *Magnaporthe grisea* . Plant Cell 13: 1987–2004. 11549759 10.1105/TPC.010056 PMC139447

[24] Gilbert MJ, Thornton CR, Wakley GE, Talbot NJ (2006) A P-type ATPase required for rice blast disease and induction of host resistance. Nature 440: 535–539. 16554820 10.1038/nature04567

[25] Bhambra GK, Wang ZY, Soanes DM, Wakley GE, Talbot NJ (2006) Peroxisomal carnitine acetyl transferase is required for elaboration of penetration hyphae during plant infection by *Magnaporthe grisea* . Mol Microbiol 61: 46–60. 16824094 10.1111/j.1365-2958.2006.05209.x

[26] Jeon J, Goh J, Yoo S, Chi MH, Choi J, et al (2008) A putative MAP kinase kinase kinase, MCK1, is required for cell wall integrity and pathogenicity of the rice blast fungus, *Magnaporthe oryzae* . Mol Plant Microbe Interact 21: 525–534. 18393612 10.1094/MPMI-21-5-0525

[27] Liu H, Suresh A, Willard FS, Siderovski DP, Lu S, et al (2007) Rgs1 regulates multiple Gα subunits in *Magnaporthe* pathogenesis, asexual growth and thigmotropism. EMBO J 26: 690–700. 17255942 10.1038/sj.emboj.7601536 PMC1794393

[28] Soundararajan S, Jedd G, Li X, Ramos-Pamplona M, Chua NH, et al (2004) Woronin body function in *Magnaporthe grisea* is essential for efficient pathogenesis and for survival during nitrogen starvation stress. Plant Cell 16: 1564–1574. 15155882 10.1105/tpc.020677 PMC490046

[29] Kershaw MJ, Talbot NJ (2009) Genome-wide functional analysis reveals that infection-associated fungal autophagy is necessary for rice blast disease. Proc Natl Acad Sci USA 106: 15967–15972. 19717456 10.1073/pnas.0901477106 PMC2747227

[30] Yi M, Park JH, Ahn JH, Lee YH (2008) *MoSNF1* regulates sporulation and pathogenicity in the rice blast fungus *Magnaporthe oryzae* . Fungal Genet Biol 45: 1172–1181. 18595748 10.1016/j.fgb.2008.05.003

[31] Kellis M, Patterson N, Endrizzi M, Birren B, Lander ES (2003) Sequencing and comparison of yeast species to identify genes and regulatory elements. Nature 423: 241–254. 12748633 10.1038/nature01644

[32] Kellis M, Birren BW, Lander ES (2004) Proof and evolutionary analysis of ancient genome duplication in the yeast *Saccharomyces cerevisiae* . Nature 428: 617–624. 15004568 10.1038/nature02424

[33] Novo M, Bigey F, Beyne E, Galeote V, Gavory F, et al (2009) Eukaryote-to-eukaryote gene transfer events revealed by the genome sequence of the wine yeast *Saccharomyces cerevisiae* EC1118. Proc Natl Acad Sci USA 106: 16333–16338. 19805302 10.1073/pnas.0904673106 PMC2740733

[34] Andersen MR, Salazar MP, Schaap PJ, van de Vondervoort PJ, Culley D, et al (2011) Comparative genomics of citric-acid-producing *Aspergillus niger* ATCC 1015 versus enzyme-producing CBS 513.88. Genome Res 21: 885–897. 21543515 10.1101/gr.112169.110 PMC3106321

[35] Ma LJ, van der Does HC, Borkovich KA, Coleman JJ, Daboussi MJ, et al (2010) Comparative genomics reveals mobile pathogenicity chromosomes in *Fusarium* . Nature 464: 367–373. 20237561 10.1038/nature08850 PMC3048781

[36] Tyler BM, Tripathy S, Zhang XM, Dehal P, Jiang RHY, et al (2006) *Phytophthora* genome sequences uncover evolutionary origins and mechanisms of pathogenesis. Science 313: 1261–1266. 16946064 10.1126/science.1128796

[37] Margulies M, Egholm M, Altman WE, Attiya S, Bader JS, et al (2005) Genome sequencing in microfabricated high-density picolitre reactors. Nature 437: 376–380. 16056220 10.1038/nature03959 PMC1464427

[38] Kurtz S, Phillippy A, Delcher AL, Smoot M, Shumway M, et al (2004) Versatile and open software for comparing large genomes. Genome Biol 5: R12. 14759262 10.1186/gb-2004-5-2-r12 PMC395750

[39] Salamov AA, Solovyev VV (2000) Ab initio gene finding in *Drosophila* genomic DNA. Genome Res 10: 516–522. 10779491 10.1101/gr.10.4.516 PMC310882

[40] Lowe TM, Eddy SR (1997) tRNAscan-SE: a program for improved detection of transfer RNA genes in genomic sequence. Nucleic Acids Res 25: 955–964. 9023104 10.1093/nar/25.5.955 PMC146525

[41] Finn RD, Tate J, Mistry J, Coggill PC, Sammut SJ, et al (2008) The Pfam protein families database. Nucleic Acids Res 36: D281–D288. 18039703 10.1093/nar/gkm960 PMC2238907

[42] Mulder NJ, Apweiler R, Attwood TK, Bairoch A, Bateman A, et al (2002) InterPro: an integrated documentation resource for protein families, domains and functional sites. Brief Bioinform 3: 225–235. 12230031 10.1093/bib/3.3.225

[43] Sonnhammer EL, von Heijne G, Krogh A (1998) A hidden Markov model for predicting transmembrane helices in protein sequences. Proc Int Conf Intell Syst Mol Biol 6: 175–182. 9783223

[44] Bendtsen JD, Nielsen H, von Heijne G, Brunak S (2004) Improved prediction of signal peptides: SignalP 3.0. J Mol Biol 340: 783–795. 15223320 10.1016/j.jmb.2004.05.028

[45] Horton P, Park K-J, Obayashi T, Fujita N, Harada H, et al (2007) WoLF PSORT: protein localization predictor. Nucleic Acids Res 35: W585–W587. 17517783 10.1093/nar/gkm259 PMC1933216

[46] Altschul SF, Madden TL, Schäffer AA, Zhang J, Zhang Z, et al (1997) Gapped BLAST and PSI-BLAST: a new generation of protein database search programs. Nucleic Acids Res 25: 3389–3402. 9254694 10.1093/nar/25.17.3389 PMC146917

[47] Li L, Stoeckert CJ, Roos DS (2003) OrthoMCL: identification of ortholog groups for eukaryotic genomes. Genome Res 13: 2178–2189. 12952885 10.1101/gr.1224503 PMC403725

[48] Notredame C, Higgins DG, Heringa J (2000) T-Coffee: a novel method for fast and accurate multiple sequence alignment. J Mol Biol 302: 205–217. 10964570 10.1006/jmbi.2000.4042

[49] Felsenstein J (2005) PHYLIP (Phylogeny Inference Package) version 3.6.

[50] Thompson JD, Higgins DG, Gibson TJ (1994) CLUSTAL W: improving the sensitivity of progressive multiple sequence alignment through sequence weighting, position-specific gap penalties and weight matrix choice. Nucleic Acids Res 22: 4673–4680. 7984417 10.1093/nar/22.22.4673 PMC308517

[51] Yang Z, Nielsen R (2000) Estimating synonymous and nonsynonymous substitution rates under realistic evolutionary models. Molecular Biology and Evolution 17: 32–43. 10666704 10.1093/oxfordjournals.molbev.a026236

[52] Wang J, Wong GKS, Ni PX, Han YJ, Huang XG, et al (2002) RePS: A sequence assembler that masks exact repeats identified from the shotgun data. Genome Res 12: 824–831. 11997349 10.1101/gr.165102 PMC186573

[53] Benson G (1999) Tandem repeats finder: a program to analyze DNA sequences. Nucleic Acids Res 27: 573–580. 9862982 10.1093/nar/27.2.573 PMC148217

[54] Park G, Bruno KS, Staiger CJ, Talbot NJ, Xu JR (2004) Independent genetic mechanisms mediate turgor generation and penetration peg formation during plant infection in the rice blast fungus. Mol Microbiol 53: 1695–1707. 15341648 10.1111/j.1365-2958.2004.04220.x

[55] Luo CX, Yin LF, Koyanagi S, Farman ML, Kusaba M, et al (2005) Genetic mapping and chromosomal assignment of *Magnaporthe oryzae* avirulence genes *AvrPik*, *AvrPiz*, and *AvrPiz-t* controlling cultivar specificity on rice. Phytopathol 95: 640–647. 10.1094/PHYTO-95-0640 18943780

[56] Yang J, Zhao X, Sun J, Kang Z, Ding S, et al (2010) A novel protein Com1 is required for normal conidium morphology and full virulence in *Magnaporthe oryzae* . Mol Plant Microbe Interact 23: 112–123. 19958144 10.1094/MPMI-23-1-0112

[57] Kong L, Yang J, Li G, Qi L, Zhang Y, et al (2012) Different chitin synthase genes are required for various developmental and plant infection processes in the rice blast fungus *Magnaporthe oryzae* . PLoS Pathog 8: e1002526. 22346755 10.1371/journal.ppat.1002526 PMC3276572

