# Supplementary figures and images for: Comparative Analysis of the Genomes of Two Field Isolates of the Rice Blast Fungus Magnaporthe oryzae
Source: PLoS Genet. 2012 Aug 2;8(8):e1002869. doi: 10.1371/journal.pgen.1002869 (PMC3410873; doi:10.1371/journal.pgen.1002869)

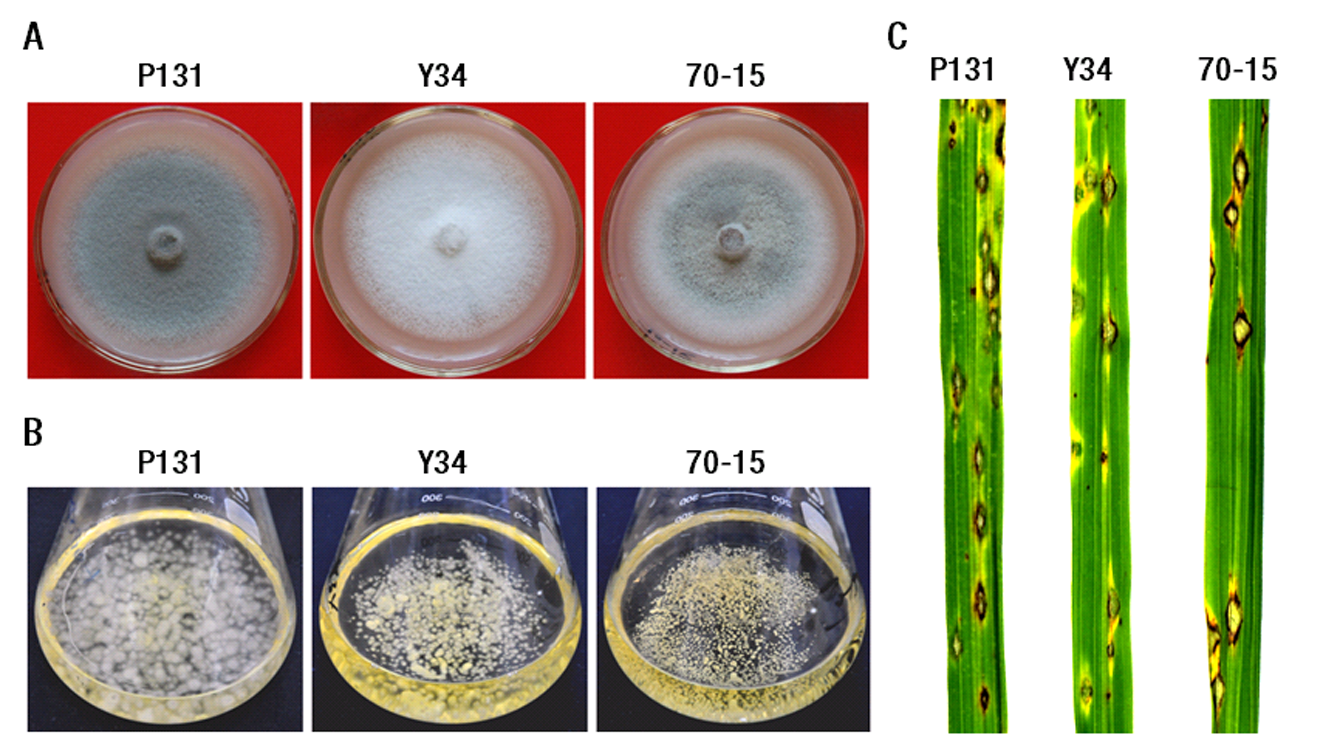

Supplement: Figure S1 — Comparison of the isolates P131, Y34, and 70-15 on asexual development and plant infection. (A) Colonies of P131, Y34, and 70-15 on OTA plates photographed at 120 hours after inoculation (hpi). (B) Vegetative mycelia of P131, Y34, and 70-15 shaken in liquid CM, photographed at 48 hpi. (C) Seedlings of the susceptible rice cultivar ‘LTH’ sprayed with conidia of P131, Y34, and 70-15, respectively, photographed 7 days after inoculation (dai). (TIF) [file pgen.1002869.s001.tif]

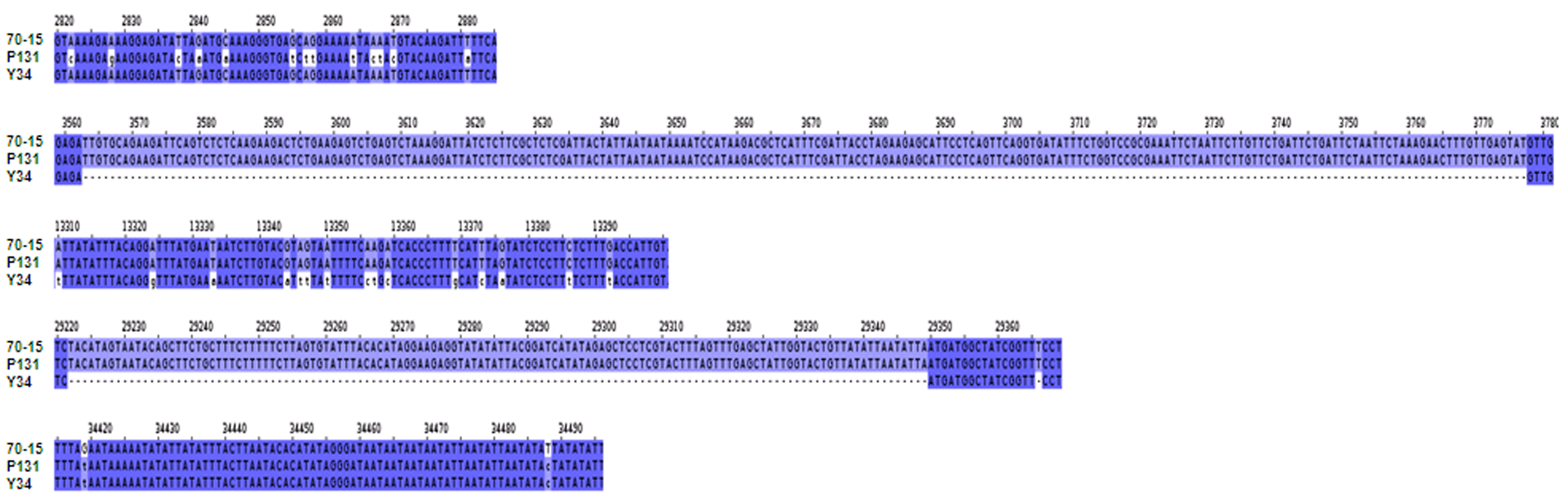

Supplement: Figure S2 — The whole mitochondrial genomes of P131 or Y34 were compared with that of 70-15. The differences in nucleotide acid substitution or deletion among the three isolates are shown. The mitochondrial genome of Y34 lacks two fragments with a combined length shorter than 350 bp. (TIF) [file pgen.1002869.s002.tif]

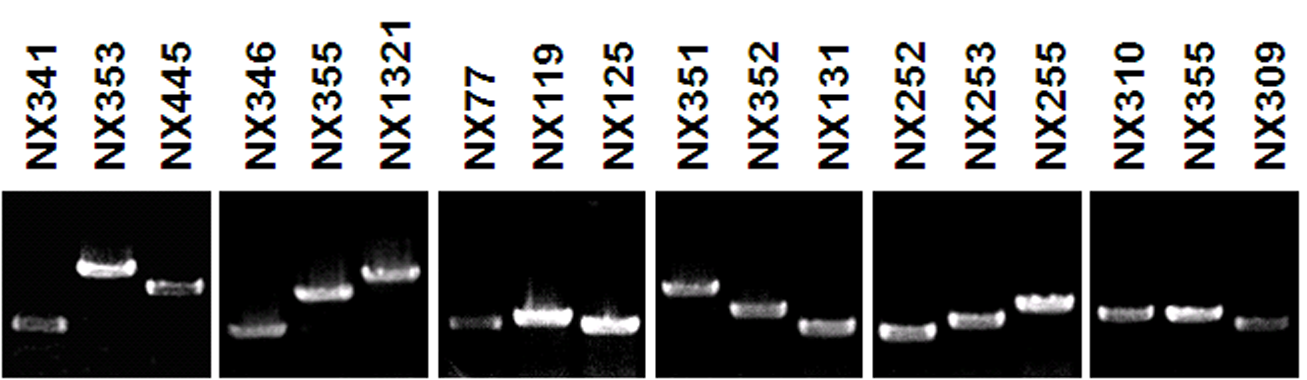

Supplement: Figure S3 — PCR validation of the gaps in the assembly of 70-15 filled with genome sequences of P131 and Y34. The genomic DNA of 70-15 was used for PCR amplifications with the primer pairs listed in Table S17. (TIF) [file pgen.1002869.s003.tif]

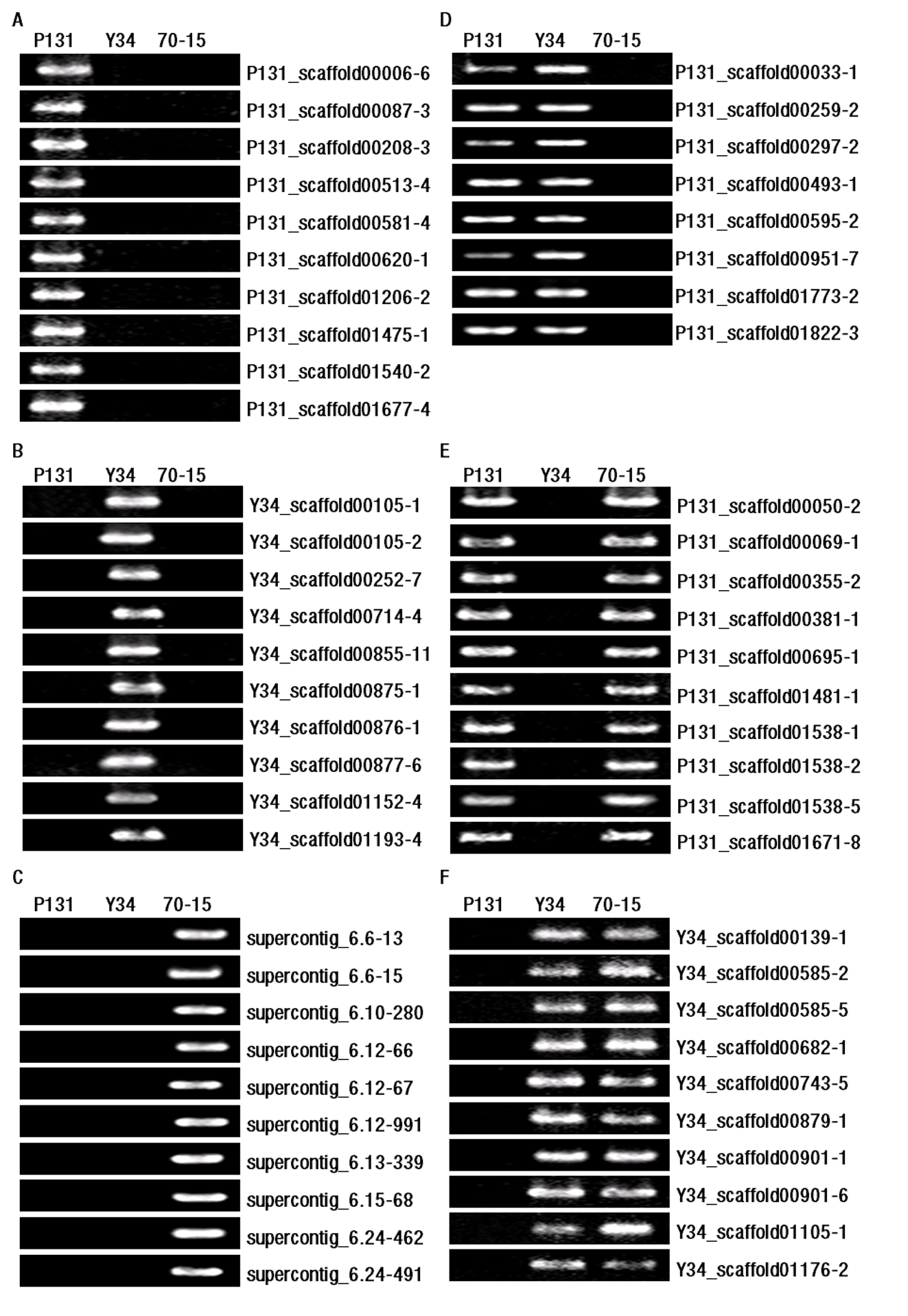

Supplement: Figure S4 — PCR validation of the selected genes unique to single isolates P131 (A), Y34 (B), or 70-15 (C), and specific to two isolates P131 and Y34 (D), P131 and 70-15 (E), or P131 and Y34 (F). The genomic DNA of isolates P131, Y34, and 70-15 were used for PCR amplification with the primer pairs shown in Table S17. (TIF) [file pgen.1002869.s004.tif]

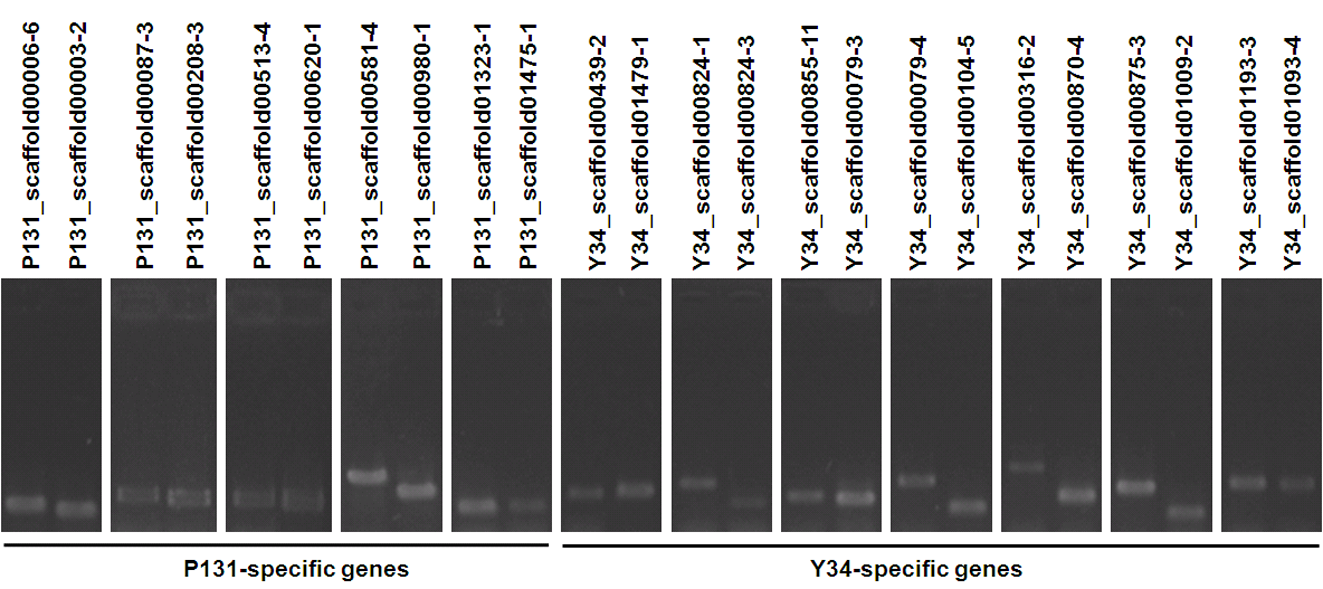

Supplement: Figure S5 — RT-PCR validation of the isolate-specific genes unique to P131 or Y34. Primers listed in Table S17 were used to amplify sequences unique to P131 or Y34 with cDNA synthesized from RNA isolated from vegetative hyphae. (TIF) [file pgen.1002869.s005.tif]

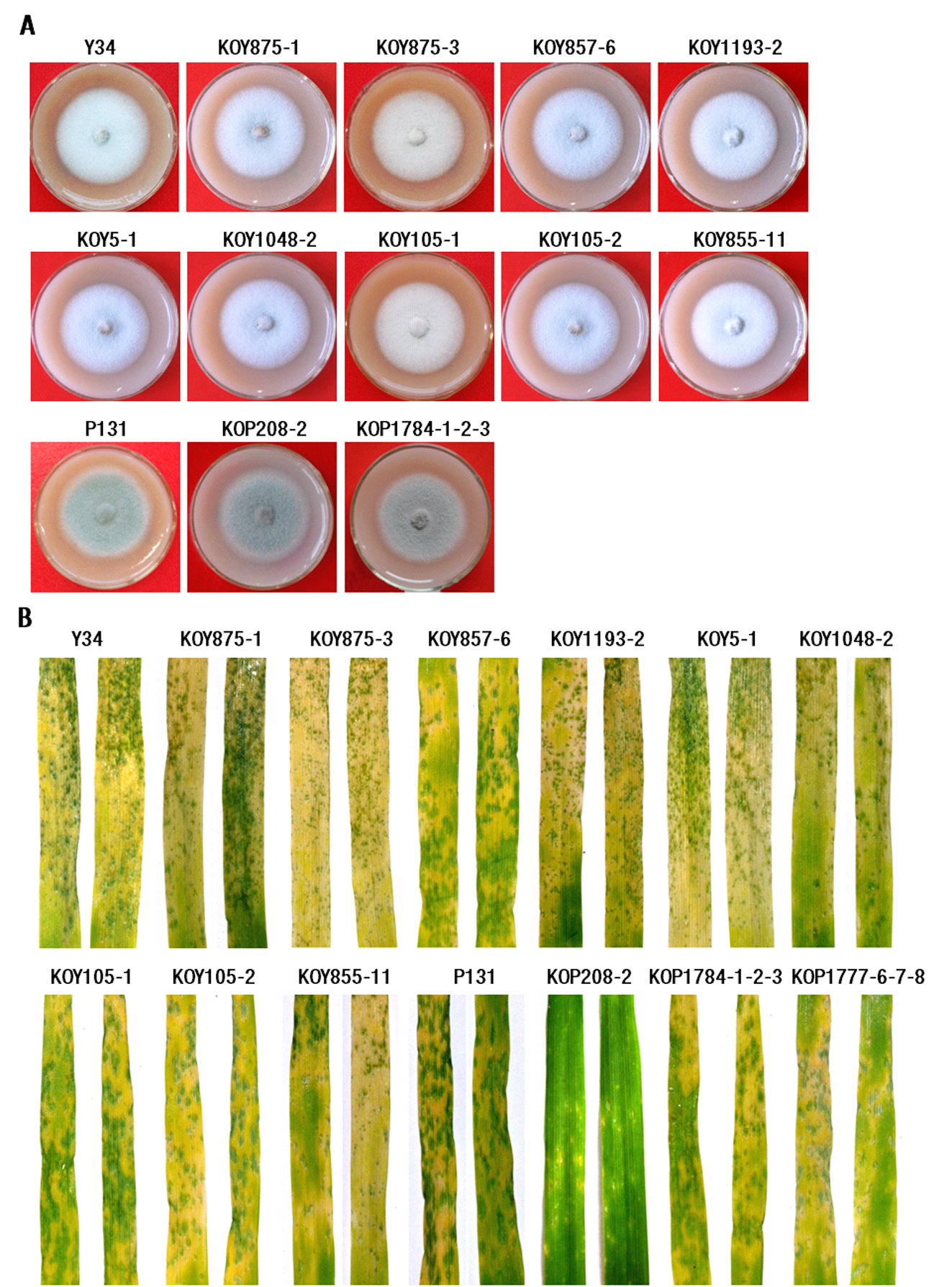

Supplement: Figure S6 — Functional analyses of 12 field isolate-specific genes. (A) Colonies of the wild-type strain Y34 and the null mutants of Y34-unique genes KOY875-1 (Y34_scaffold00875-1), KOY875-3 (Y34_scaffold00875-3), KOY857-6 (Y34_scaffold00857-6), KOY1193-2 (Y34_scaffold01193-2), KOY5-1 (Y34_scaffold00005-1), KOY1048-2 (Y34_scaffold01048-2), KOY105-1 (Y34_scaffold00105-1), KOY105-2 (Y34_scaffold00105-2), KOY855-11 (Y34_scaffold00855-11), and the wild-type strain P131 and the null mutants of P131-unique genes KOP208-2 (P131_scaffold00208-2) and KOP1784-1-2-3 (P131_scaffold01784-1-2-3). Representative photographs were taken on OTA plates 5 dai. (B) Barley seedlings sprayed with conidia of the wild-type strains P131 and Y34, and with null mutants of all 12 genes photographed 5 dai. (TIF) [file pgen.1002869.s006.tif]

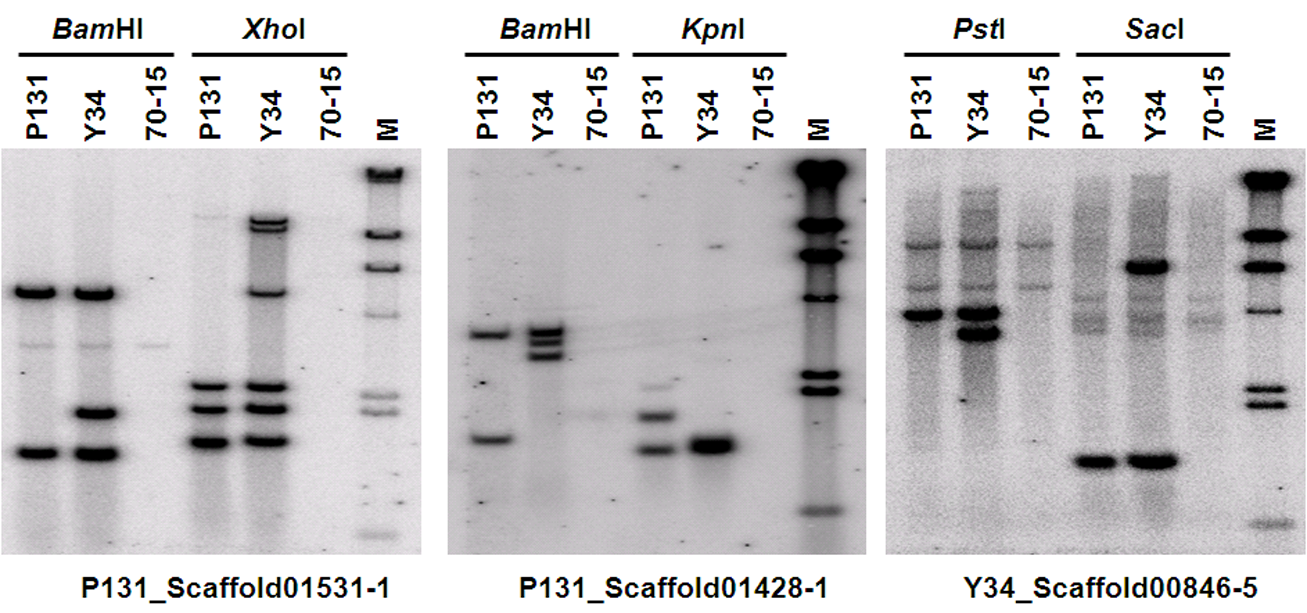

Supplement: Figure S7 — Verification of genes duplicated specifically to the field isolates. Genomic DNA of P131, Y34, and 70-15 were digested by two restriction enzymes. Amplified fragments of the gene P131_scaffold01531-1, P131_scaffold01428-1, and Y34_scaffold00846-5 were used as probes. M, λ-HindIII ladder. (TIF) [file pgen.1002869.s007.tif]
