# Supplementary material for: Comparative Analysis of the Genomes of Two Field Isolates of the Rice Blast Fungus Magnaporthe oryzae
Source: PLoS Genet. 2012 Aug 2;8(8):e1002869. doi: 10.1371/journal.pgen.1002869 (PMC3410873; doi:10.1371/journal.pgen.1002869)
Supplement: Table S1 — Pathotypes of P131, Y34, and 70-15 based on their infectivity towards different monogenic rice cultivars generated by the International Rice Research Institute. (DOC) [file pgen.1002869.s009.doc]

**Table S1** Pathotypes of P131, Y34, and 70-15 based on their infectivity towards different monogenic rice cultivars generated by the International Rice Research Institute.

(a) Avirulence (*Avr*) genes in the isolates P131, Y34, and 70-15.

| **Isolate** | **Avirulence gene** | | | | | | |
| --- | --- | --- | --- | --- | --- | --- | --- |
| P131 | *Avr-Pi-5* | *Avr-Pi-9* | *Avr-Pi-12* | *Avr-Pi-20* | *Avr-Pi-a* | *Avr-Pi-b* | *Avr-Pi-i* |
|  | *Avr-Piz* | *Avr-Pi-zt* |  |  |  |  |  |
| Y34 | *Avr-Pi-1* | *Avr-Pi-3* | *Avr-Pi-5* | *Avr-Pi-7* | *Avr-Pi-11* | *Avr-Pi-12* | *Avr-Pi-20* |
|  | *Avr-Pi-a* | *Avr-Pi-i* | *Avr-Pi-k* | *Avr-Pi-kh* | *Avr-Pi-km* | *Avr-Pi-kp* | *Avr-Pi-ks* |
|  | *Avr-Pi-ta2* | *Avr-Pi-z* |  |  |  |  |  |
| 70-15 | *Avr-Pi-3* | *Avr-Pi-5* | *Avr-Pi-9* | *Avr-Pi-11* | *Avr-Pi-12* | *Avr-Pi-19* | *Avr-Pi-20* |
|  | *Avr-Pi-i* | *Avr-Pi-kh* | *Avr-Pi-km* | *Avr-Pi-sh* | *Avr-Pi-z* |  |  |

(b) Monogenic cultivars used for detecting the reactions of isolates P131, Y34, and 70-15.

| **Monogenic cultivar^1^** | **R gene** | **Donor cultivar** | **Isolate** | | |
| --- | --- | --- | --- | --- | --- |
|  |  |  | **P131** | **Y34** | **70-15** |
| IRBL1-CL | *Pi1* | C101LAC | S**^2^** | R | S |
| IRBL3-CP4 | *Pi3* | C104PKT | S | R | R |
| IRBL5-M | *Pi5* | Moroberekan(RIL249) | R | R | R |
| IRBL7-M | *Pi7* | Moroberekan(RIL29) | S | R | S |
| IRBL9-W | *Pi9* | WHD-1S-75-1-127 | R | S | R |
| IRBL11-Zh | *Pi11* | Zhaiyeqing 8 | S | R | R |
| IRBL12-M | *Pi12* | Moroberekan(RIL10) | R | R | R |
| IRBL19-A | *Pi19* | Aichi Asahi | S | S | R |
| IRBL20-IR24 | *Pi20* | IR24 | R | R | R |
| IRBLa-A | *Pia* | Aichi Asahi | R | R | S |
| IRBLb-B | *Pib* | BL1 | R | S | S |
| IRBLi-F5 | *Pii* | Fujisaki5 | R | R | R |
| IRBLk-Ka | *Pik* | Kanto51 | S | R | S |
| IRBLkh-K3 | *Pikh* | K3 | S | R | R |
| IRBLkm-Ts | *Pikm* | Tsuyuake | S | R | R |
| IRBLkp-K60 | *Pikp* | K60 | S | R | S |
| IRBLks-F5 | *Piks* | Fujisaki5 | S | R | S |
| IRBLsh-S | *Pish* | Shin2 | S | S | R |
| IRBLta2-Re | *Pita2* | Reiho | S | R | S |
| IRBLz-Fu | *Piz* | Fukunishiki | R | R | R |
| IRBLzt-T | *Pizt* | Toride 1 | R | S | S |

^1^The monogenic rice lines were generated by crossing ‘LTH’ (a Japonica rice cultivar without any known R gene from Yunnan, China) with individual donor cultivars containing R gene.

^2^S, susceptible; R, resistant.
