# Supplementary material for: Comparative Analysis of the Genomes of Two Field Isolates of the Rice Blast Fungus Magnaporthe oryzae
Source: PLoS Genet. 2012 Aug 2;8(8):e1002869. doi: 10.1371/journal.pgen.1002869 (PMC3410873; doi:10.1371/journal.pgen.1002869)
Supplement: Table S2 — Genes in 70-15 with potential annotation errors adjusted with data from the assembled genomes of P131 and Y34. (DOC) [file pgen.1002869.s010.doc]

**Table S2** Genes in 70-15 with potential annotation errors adjusted with data from the assembled genomes of P131 and Y34.

| **70-15 ortholog** | **P131 ortholog** | **Y34 ortholog** | **Issue** |
| --- | --- | --- | --- |
| MGG_00417.6 | P131_scaffold00470-10 | Y34_scaffold00203-104 | start codon |
| MGG_00504.6 | P131_scaffold00593-5 | Y34_scaffold00203-3 | start codon |
| MGG_00516.6 | P131_scaffold01046-12 | Y34_scaffold00522-12 | start codon |
| MGG_00522.6 | P131_scaffold01046-19 | Y34_scaffold00522-19 | start codon |
| MGG_00528.6 | P131_scaffold01046-25 | Y34_scaffold00522-25 | intron |
| MGG_00551.6 | P131_scaffold01708-17 | Y34_scaffold00522-53 | start codon |
| MGG_00598.6 | P131_scaffold00267-61 | Y34_scaffold00528-49 | start codon |
| MGG_00633.6 | P131_scaffold00267-5 | Y34_scaffold00134-6 | start codon |
| MGG_00651.6 | P131_scaffold00499-28 | Y34_scaffold00099-28 | start codon |
| MGG_00692.6 | P131_scaffold01213-19 | Y34_scaffold00669-18 | start codon |
| MGG_00759.6 | P131_scaffold01068-27 | Y34_scaffold00669-91 | start codon |
| MGG_00785.6 | P131_scaffold01307-8 | Y34_scaffold00153-13 | start codon |
| MGG_00803.6 | P131_scaffold00602-21 | Y34_scaffold01007-21 | start codon |
| MGG_00809.6 | P131_scaffold00602-15 | Y34_scaffold01007-15 | start codon, intron |
| MGG_00817.6 | P131_scaffold00602-6 | Y34_scaffold01007-6 | stop codon, intron |
| MGG_00819.6 | P131_scaffold00602-3 | Y34_scaffold01007-3 | start codon, intron |
| MGG_00926.6 | P131_scaffold00345-88 | Y34_scaffold00126-120 | start codon |
| MGG_01018.6 | P131_scaffold00001-1 | Y34_scaffold00341-1 | intron |
| MGG_01023.6 | P131_scaffold00713-4 | Y34_scaffold00496-4 | start codon |
| MGG_01043.6 | P131_scaffold01325-61 | Y34_scaffold00496-24 | intron |
| MGG_01050.6 | P131_scaffold01325-53 | Y34_scaffold00496-32 | start codon |
| MGG_01062.6 | P131_scaffold01325-41 | Y34_scaffold00496-44 | start codon |
| MGG_01073.6 | P131_scaffold01325-29 | Y34_scaffold00496-56 | start codon, intron |
| MGG_01124.6 | P131_scaffold00304-16 | Y34_scaffold00697-29 | start codon, stop codon, intron |
| MGG_01180.6 | P131_scaffold01028-18 | Y34_scaffold00476-21 | start codon |
| MGG_01221.6 | P131_scaffold00497-7 | Y34_scaffold00594-5 | intron |
| MGG_01279.6 | P131_scaffold01058-11 | Y34_scaffold00207-51 | start codon |
| MGG_01338.6 | P131_scaffold01041-2 | Y34_scaffold00635-2 | intron |
| MGG_01356.6 | P131_scaffold00131-3 | Y34_scaffold00530-12 | start codon |
| MGG_01753.6 | P131_scaffold00974-37 | Y34_scaffold00295-8 | intron |
| MGG_01819.6 | P131_scaffold00254-11 | Y34_scaffold00641-56 | start codon |
| MGG_01849.6 | P131_scaffold00491-4 | Y34_scaffold00641-21 | start codon |
| MGG_01850.6 | P131_scaffold00491-3 | Y34_scaffold00641-20 | start codon |
| MGG_01862.6 | P131_scaffold00246-5 | Y34_scaffold00641-5 | intron |
| MGG_01868.6 | P131_scaffold00263-4 | Y34_scaffold00261-3 | stop codon, intron |
| MGG_02341.6 | P131_scaffold01098-2 | Y34_scaffold00940-2 | start codon |
| MGG_02371.6 | P131_scaffold00395-8 | Y34_scaffold00665-7 | start codon, intron |
| MGG_02403.6 | P131_scaffold00408-7 | Y34_scaffold00686-11 | start codon, intron |
| MGG_02442.6 | P131_scaffold01116-17 | Y34_scaffold00538-2 | start codon, stop codon, intron |
| MGG_02444.6 | P131_scaffold00400-44 | Y34_scaffold00289-7 | start codon |
| MGG_02457.6 | P131_scaffold00400-27 | Y34_scaffold00725-12 | intron |
| MGG_02459.6 | P131_scaffold00400-25 | Y34_scaffold00725-14 | start codon |
| MGG_02464.6 | P131_scaffold00400-20 | Y34_scaffold00725-19 | start codon |
| MGG_02475.6 | P131_scaffold00400-9 | Y34_scaffold00725-30 | start codon |
| MGG_02495.6 | P131_scaffold00546-24 | Y34_scaffold00712-24 | start codon |
| MGG_02507.6 | P131_scaffold00546-3 | Y34_scaffold00712-3 | start codon |
| MGG_02511.6 | P131_scaffold00343-4 | Y34_scaffold00278-28 | start codon |
| MGG_02568.6 | P131_scaffold00326-7 | Y34_scaffold00107-7 | stop codon |
| MGG_02569.6 | P131_scaffold00326-8 | Y34_scaffold00107-8 | intron |
| MGG_02615.6 | P131_scaffold01027-34 | Y34_scaffold00666-201 | start codon |
| MGG_02626.6 | P131_scaffold01027-19 | Y34_scaffold00666-186 | start codon |
| MGG_02692.6 | P131_scaffold00372-7 | Y34_scaffold00666-100 | start codon |
| MGG_02708.6 | P131_scaffold00596-14 | Y34_scaffold00666-79 | start codon |
| MGG_02712.6 | P131_scaffold00596-10 | Y34_scaffold00666-75 | start codon |
| MGG_02737.6 | P131_scaffold00283-6 | Y34_scaffold00666-47 | intron |
| MGG_02748.6 | P131_scaffold01340-7 | Y34_scaffold00666-36 | start codon |
| MGG_02752.6 | P131_scaffold01340-3 | Y34_scaffold00666-32 | start codon |
| MGG_02797.6 | P131_scaffold01054-54 | Y34_scaffold00301-25 | start codon |
| MGG_02814.6 | P131_scaffold01054-74 | Y34_scaffold00301-45 | start codon, intron |
| MGG_02880.6 | P131_scaffold00601-5 | Y34_scaffold00150-9 | start codon |
| MGG_02891.6 | P131_scaffold00941-5 | Y34_scaffold00458-49 | start codon |
| MGG_02983.6 | P131_scaffold01153-7 | Y34_scaffold00581-8 | intron |
| MGG_02986.6 | P131_scaffold01153-10 | Y34_scaffold00581-5 | stop codon, intron |
| MGG_02996.6 | P131_scaffold01007-56 | Y34_scaffold00275-59 | start codon |
| MGG_03005.6 | P131_scaffold01007-46 | Y34_scaffold00275-49 | start codon |
| MGG_03021.6 | P131_scaffold01007-26 | Y34_scaffold00275-29 | start codon, intron |
| MGG_03167.6 | P131_scaffold01199-18 | Y34_scaffold00155-30 | start codon |
| MGG_03434.6 | P131_scaffold00357-9 | Y34_scaffold00437-14 | intron |
| MGG_03497.6 | P131_scaffold00333-3 | Y34_scaffold00448-62 | start codon, intron |
| MGG_03518.6 | P131_scaffold01276-14 | Y34_scaffold00668-14 | start codon, intron |
| MGG_03548.6 | P131_scaffold01337-15 | Y34_scaffold00662-14 | start codon, intron |
| MGG_03557.6 | P131_scaffold01337-24 | Y34_scaffold00662-5 | start codon, intron |
| MGG_03622.6 | P131_scaffold01320-26 | Y34_scaffold00654-26 | start codon |
| MGG_03627.6 | P131_scaffold00429-31 | Y34_scaffold00654-30 | intron |
| MGG_03695.6 | P131_scaffold01168-43 | Y34_scaffold00542-30 | intron |
| MGG_03705.6 | P131_scaffold01168-54 | Y34_scaffold00542-41 | start codon |
| MGG_03862.6 | P131_scaffold01331-23 | Y34_scaffold00487-19 | start codon |
| MGG_03871.6 | P131_scaffold01331-8 | Y34_scaffold00487-34 | start codon |
| MGG_03888.6 | P131_scaffold01181-8 | Y34_scaffold00487-54 | start codon |
| MGG_03957.6 | P131_scaffold01195-3 | Y34_scaffold00514-3 | start codon |
| MGG_03994.6 | P131_scaffold00186-12 | Y34_scaffold00514-42 | start codon |
| MGG_04030.6 | P131_scaffold01683-7 | Y34_scaffold00514-85 | start codon |
| MGG_04089.6 | P131_scaffold01683-69 | Y34_scaffold00689-5 | intron |
| MGG_04095.6 | P131_scaffold01349-5 | Y34_scaffold00255-76 | start codon |
| MGG_04133.6 | P131_scaffold00751-20 | Y34_scaffold00255-32 | intron |
| MGG_04162.6 | P131_scaffold01393-14 | Y34_scaffold00579-2 | intron |
| MGG_04210.6 | P131_scaffold01057-5 | Y34_scaffold00540-40 | start codon |
| MGG_04237.6 | P131_scaffold00562-14 | Y34_scaffold00540-71 | intron |
| MGG_04382.6 | P131_scaffold01166-3 | Y34_scaffold00744-3 | start codon |
| MGG_04453.6 | P131_scaffold01358-24 | Y34_scaffold00744-57 | start codon |
| MGG_04525.6 | P131_scaffold01358-51 | Y34_scaffold00619-14 | start codon |
| MGG_04536.6 | P131_scaffold01358-63 | Y34_scaffold00619-26 | intron |
| MGG_04570.6 | P131_scaffold01358-75 | Y34_scaffold00619-37 | stop codon, intron |
| MGG_04599.6 | P131_scaffold00377-4 | Y34_scaffold00500-4 | start codon |
| MGG_04600.6 | P131_scaffold01358-92 | Y34_scaffold00619-54 | start codon |
| MGG_04617.6 | P131_scaffold01358-106 | Y34_scaffold00619-68 | intron |
| MGG_04660.6 | P131_scaffold00308-6 | Y34_scaffold00749-11 | start codon |
| MGG_04665.6 | P131_scaffold00308-1 | Y34_scaffold00749-16 | start codon, intron |
| MGG_04673.6 | P131_scaffold00467-8 | Y34_scaffold00194-105 | start codon, intron |
| MGG_04724.6 | P131_scaffold00538-51 | Y34_scaffold00194-79 | intron |
| MGG_04788.6 | P131_scaffold00538-18 | Y34_scaffold00194-46 | intron |
| MGG_04843.6 | P131_scaffold01383-5 | Y34_scaffold00745-3 | start codon |
| MGG_04873.6 | P131_scaffold00314-10 | Y34_scaffold00745-38 | start codon |
| MGG_04879.6 | P131_scaffold00314-16 | Y34_scaffold00745-44 | start codon, intron |
| MGG_04889.6 | P131_scaffold00314-28 | Y34_scaffold00745-56 | start codon |
| MGG_04916.6 | P131_scaffold00314-61 | Y34_scaffold00745-89 | start codon |
| MGG_04929.6 | P131_scaffold00314-74 | Y34_scaffold00745-102 | start codon |
| MGG_04942.6 | P131_scaffold00314-89 | Y34_scaffold00071-66 | start codon, intron |
| MGG_04947.6 | P131_scaffold00314-93 | Y34_scaffold00071-61 | start codon |
| MGG_05009.6 | P131_scaffold00302-8 | Y34_scaffold00174-15 | start codon |
| MGG_05013.6 | P131_scaffold00302-4 | Y34_scaffold00174-19 | start codon |
| MGG_05033.6 | P131_scaffold00266-48 | Y34_scaffold00174-43 | start codon |
| MGG_05035.6 | P131_scaffold00266-46 | Y34_scaffold00174-45 | start codon, intron |
| MGG_05051.6 | P131_scaffold00266-28 | Y34_scaffold00174-64 | start codon |
| MGG_05110.6 | P131_scaffold01120-11 | Y34_scaffold00510-18 | start codon |
| MGG_05150.6 | P131_scaffold00982-34 | Y34_scaffold00516-30 | start codon |
| MGG_05156.6 | P131_scaffold00982-42 | Y34_scaffold00516-38 | start codon |
| MGG_05216.6 | P131_scaffold01122-6 | Y34_scaffold00516-114 | intron |
| MGG_05219.6 | P131_scaffold01012-9 | Y34_scaffold00526-3 | start codon |
| MGG_05225.6 | P131_scaffold00159-4 | Y34_scaffold00526-14 | start codon, intron |
| MGG_05252.6 | P131_scaffold01006-20 | Y34_scaffold00711-22 | intron |
| MGG_05261.6 | P131_scaffold01006-11 | Y34_scaffold00711-13 | start codon |
| MGG_05279.6 | P131_scaffold00262-1 | Y34_scaffold00171-9 | start codon |
| MGG_05284.6 | P131_scaffold00566-5 | Y34_scaffold00621-4 | start codon, intron |
| MGG_05312.6 | P131_scaffold00095-33 | Y34_scaffold00033-25 | intron |
| MGG_05327.6 | P131_scaffold00095-15 | Y34_scaffold00033-43 | start codon |
| MGG_05351.6 | P131_scaffold01111-2 | Y34_scaffold00589-23 | intron |
| MGG_05379.6 | P131_scaffold00083-32 | Y34_scaffold00790-10 | start codon |
| MGG_05400.6 | P131_scaffold00524-1 | Y34_scaffold00141-3 | start codon, intron |
| MGG_05445.6 | P131_scaffold00366-10 | Y34_scaffold00467-14 | intron |
| MGG_05622.6 | P131_scaffold00130-5 | Y34_scaffold00624-65 | start codon |
| MGG_05633.6 | P131_scaffold00130-16 | Y34_scaffold00624-54 | intron |
| MGG_05654.6 | P131_scaffold01379-10 | Y34_scaffold00624-31 | start codon |
| MGG_05677.6 | P131_scaffold00294-23 | Y34_scaffold00576-32 | start codon |
| MGG_05687.6 | P131_scaffold00294-10 | Y34_scaffold00576-19 | start codon |
| MGG_05722.6 | P131_scaffold01338-63 | Y34_scaffold00567-26 | intron |
| MGG_05746.6 | P131_scaffold01338-31 | Y34_scaffold00567-55 | intron |
| MGG_05747.6 | P131_scaffold01338-30 | Y34_scaffold00567-56 | stop codon |
| MGG_05765.6 | P131_scaffold01338-5 | Y34_scaffold00567-81 | start codon |
| MGG_05829.6 | P131_scaffold01347-5 | Y34_scaffold00453-5 | intron |
| MGG_05853.6 | P131_scaffold00170-5 | Y34_scaffold00036-2 | intron |
| MGG_05901.6 | P131_scaffold00360-4 | Y34_scaffold00620-6 | start codon |
| MGG_06151.6 | P131_scaffold00455-8 | Y34_scaffold00192-6 | start codon |
| MGG_06153.6 | P131_scaffold00455-10 | Y34_scaffold00192-8 | start codon |
| MGG_06181.6 | P131_scaffold00455-46 | Y34_scaffold00192-44 | start codon |
| MGG_06212.6 | P131_scaffold00365-6 | Y34_scaffold00062-11 | stop codon, intron |
| MGG_06215.6 | P131_scaffold00365-2 | Y34_scaffold00062-15 | start codon |
| MGG_06247.6 | P131_scaffold00027-8 | Y34_scaffold00748-12 | start codon |
| MGG_06257.6 | P131_scaffold01189-21 | Y34_scaffold00748-23 | start codon, intron |
| MGG_06264.6 | P131_scaffold01189-13 | Y34_scaffold00748-31 | start codon |
| MGG_06297.6 | P131_scaffold01171-14 | Y34_scaffold00559-36 | intron |
| MGG_06301.6 | P131_scaffold01171-18 | Y34_scaffold00559-32 | start codon, intron |
| MGG_06302.6 | P131_scaffold01171-19 | Y34_scaffold00559-31 | start codon |
| MGG_06312.6 | P131_scaffold00322-10 | Y34_scaffold00559-20 | start codon |
| MGG_06317.6 | P131_scaffold00568-2 | Y34_scaffold00559-15 | start codon |
| MGG_06350.6 | P131_scaffold00151-3 | Y34_scaffold00095-30 | intron |
| MGG_06366.6 | P131_scaffold00505-11 | Y34_scaffold00095-10 | start codon |
| MGG_06467.6 | P131_scaffold01381-34 | Y34_scaffold00247-39 | start codon |
| MGG_06486.6 | P131_scaffold01381-11 | Y34_scaffold00247-63 | stop codon, intron |
| MGG_06546.6 | P131_scaffold00265-11 | Y34_scaffold00037-25 | start codon |
| MGG_06583.6 | P131_scaffold00539-20 | Y34_scaffold00182-18 | start codon |
| MGG_06689.6 | P131_scaffold00991-6 | Y34_scaffold00533-57 | start codon |
| MGG_06745.6 | P131_scaffold00177-37 | Y34_scaffold00534-37 | start codon |
| MGG_06924.6 | P131_scaffold00328-10 | Y34_scaffold00140-10 | start codon |
| MGG_06986.6 | P131_scaffold01188-5 | Y34_scaffold00663-15 | start codon |
| MGG_06988.6 | P131_scaffold01188-7 | Y34_scaffold00663-13 | start codon |
| MGG_07013.6 | P131_scaffold00255-5 | Y34_scaffold00649-19 | intron |
| MGG_07043.6 | P131_scaffold00522-2 | Y34_scaffold00707-79 | start codon |
| MGG_07063.6 | P131_scaffold01192-4 | Y34_scaffold00707-58 | start codon |
| MGG_07103.6 | P131_scaffold01192-56 | Y34_scaffold00707-5 | stop codon, intron |
| MGG_07138.6 | P131_scaffold01179-30 | Y34_scaffold00773-21 | start codon |
| MGG_07238.6 | P131_scaffold00340-10 | Y34_scaffold00493-25 | start codon |
| MGG_07267.6 | P131_scaffold00421-8 | Y34_scaffold00597-27 | start codon |
| MGG_07286.6 | P131_scaffold01066-33 | Y34_scaffold00597-5 | start codon |
| MGG_07319.6 | P131_scaffold00535-2 | Y34_scaffold00768-5 | start codon |
| MGG_07414.6 | P131_scaffold00453-1 | Y34_scaffold00568-1 | start codon, intron |
| MGG_07423.6 | P131_scaffold01326-4 | Y34_scaffold00552-126 | start codon |
| MGG_07430.6 | P131_scaffold01326-15 | Y34_scaffold00552-115 | start codon |
| MGG_07441.6 | P131_scaffold00115-2 | Y34_scaffold00552-100 | start codon |
| MGG_07487.6 | P131_scaffold01198-17 | Y34_scaffold00552-51 | start codon |
| MGG_07493.6 | P131_scaffold01198-23 | Y34_scaffold00552-45 | start codon, intron |
| MGG_07517.6 | P131_scaffold01198-51 | Y34_scaffold00552-17 | start codon, intron |
| MGG_07561.6 | P131_scaffold00121-10 | Y34_scaffold00736-1 | start codon |
| MGG_07568.6 | P131_scaffold00961-9 | Y34_scaffold00132-3 | intron |
| MGG_07607.6 | P131_scaffold00555-18 | Y34_scaffold00719-47 | start codon |
| MGG_07636.6 | P131_scaffold01139-4 | Y34_scaffold00719-13 | start codon, intron |
| MGG_07724.6 | P131_scaffold00984-29 | Y34_scaffold00287-4 | start codon |
| MGG_07728.6 | P131_scaffold00984-26 | Y34_scaffold00287-7 | start codon |
| MGG_07806.6 | P131_scaffold00638-2 | Y34_scaffold00630-2 | start codon, intron |
| MGG_07859.6 | P131_scaffold00465-1 | Y34_scaffold00733-15 | intron |
| MGG_07949.6 | P131_scaffold01770-6 | Y34_scaffold00732-1 | start codon |
| MGG_07997.6 | P131_scaffold00750-7 | Y34_scaffold00200-2 | start codon |
| MGG_07999.6 | P131_scaffold00750-9 | Y34_scaffold00200-4 | start codon |
| MGG_08021.6 | P131_scaffold00062-19 | Y34_scaffold00927-19 | start codon |
| MGG_08022.6 | P131_scaffold00062-20 | Y34_scaffold00927-20 | start codon |
| MGG_08048.6 | P131_scaffold01519-16 | Y34_scaffold00492-47 | start codon, intron |
| MGG_08058.6 | P131_scaffold00264-36 | Y34_scaffold00492-36 | start codon |
| MGG_08060.6 | P131_scaffold00264-34 | Y34_scaffold00492-34 | start codon, intron |
| MGG_08084.6 | P131_scaffold00264-8 | Y34_scaffold00492-8 | start codon |
| MGG_08111.6 | P131_scaffold00067-2 | Y34_scaffold00094-23 | start codon |
| MGG_08130.6 | P131_scaffold01076-13 | Y34_scaffold00094-45 | intron |
| MGG_08134.6 | P131_scaffold01076-18 | Y34_scaffold00094-50 | start codon |
| MGG_08175.6 | P131_scaffold01039-3 | Y34_scaffold00638-8 | intron |
| MGG_08239.6 | P131_scaffold01186-3 | Y34_scaffold00027-3 | intron |
| MGG_08320.6 | P131_scaffold00274-18 | Y34_scaffold00323-4 | start codon |
| MGG_08395.6 | P131_scaffold00621-2 | Y34_scaffold00138-2 | start codon |
| MGG_08412.6 | P131_scaffold01649-4 | Y34_scaffold00355-3 | start codon |
| MGG_08462.6 | P131_scaffold00599-10 | Y34_scaffold00628-6 | start codon |
| MGG_08463.6 | P131_scaffold00599-11 | Y34_scaffold00628-4 | start codon |
| MGG_08467.6 | P131_scaffold00972-3 | Y34_scaffold00403-3 | intron |
| MGG_08532.6 | P131_scaffold00942-5 | Y34_scaffold00983-2 | start codon |
| MGG_08536.6 | P131_scaffold00942-10 | Y34_scaffold00983-7 | intron |
| MGG_08555.6 | P131_scaffold01117-4 | Y34_scaffold00211-15 | start codon, intron |
| MGG_08587.6 | P131_scaffold01302-21 | Y34_scaffold00370-19 | start codon |
| MGG_08622.6 | P131_scaffold00367-6 | Y34_scaffold00685-12 | start codon, intron |
| MGG_08656.6 | P131_scaffold01208-9 | Y34_scaffold01090-9 | start codon |
| MGG_08688.6 | P131_scaffold00278-4 | Y34_scaffold01018-29 | start codon |
| MGG_08738.6 | P131_scaffold00152-9 | Y34_scaffold01075-27 | intron |
| MGG_08891.6 | P131_scaffold01214-18 | Y34_scaffold00240-54 | start codon, intron |
| MGG_09022.6 | P131_scaffold00516-20 | Y34_scaffold00969-32 | start codon |
| MGG_09049.6 | P131_scaffold01220-3 | Y34_scaffold00962-6 | start codon |
| MGG_09129.6 | P131_scaffold00628-2 | Y34_scaffold00539-8 | start codon, intron |
| MGG_09144.6 | P131_scaffold01169-1 | Y34_scaffold00791-1 | start codon |
| MGG_09212.6 | P131_scaffold00382-21 | Y34_scaffold00283-72 | start codon, intron |
| MGG_09215.6 | P131_scaffold00382-17 | Y34_scaffold00283-68 | start codon |
| MGG_09223.6 | P131_scaffold00382-9 | Y34_scaffold00283-60 | start codon |
| MGG_09341.6 | P131_scaffold00275-3 | Y34_scaffold00631-5 | start codon, intron |
| MGG_09360.6 | P131_scaffold01336-2 | Y34_scaffold00485-4 | start codon |
| MGG_09381.6 | P131_scaffold01043-6 | Y34_scaffold00258-8 | start codon |
| MGG_09469.6 | P131_scaffold00484-6 | Y34_scaffold00511-35 | intron |
| MGG_09543.6 | P131_scaffold00320-18 | Y34_scaffold00548-48 | intron |
| MGG_09544.6 | P131_scaffold00320-17 | Y34_scaffold00548-49 | start codon |
| MGG_09553.6 | P131_scaffold00320-8 | Y34_scaffold00548-58 | start codon, intron |
| MGG_09604.6 | P131_scaffold00182-7 | Y34_scaffold00101-10 | intron |
| MGG_09697.6 | P131_scaffold01291-8 | Y34_scaffold01003-33 | start codon, intron |
| MGG_09710.6 | P131_scaffold01291-24 | Y34_scaffold01003-17 | start codon |
| MGG_09794.6 | P131_scaffold00180-4 | Y34_scaffold01106-5 | start codon, intron |
| MGG_09830.6 | P131_scaffold00272-6 | Y34_scaffold00435-5 | intron |
| MGG_09860.6 | P131_scaffold00045-16 | Y34_scaffold00021-25 | start codon |
| MGG_09887.6 | P131_scaffold01201-42 | Y34_scaffold00648-9 | start codon |
| MGG_09899.6 | P131_scaffold01201-30 | Y34_scaffold00648-21 | start codon |
| MGG_09959.6 | P131_scaffold00349-5 | Y34_scaffold00106-5 | start codon |
| MGG_09994.6 | P131_scaffold01178-2 | Y34_scaffold00694-9 | start codon |
| MGG_10192.6 | P131_scaffold00195-13 | Y34_scaffold00087-14 | start codon |
| MGG_10196.6 | P131_scaffold00195-17 | Y34_scaffold00087-10 | intron |
| MGG_10212.6 | P131_scaffold01003-9 | Y34_scaffold00713-1 | intron |
| MGG_10216.6 | P131_scaffold01003-3 | Y34_scaffold00713-7 | intron |
| MGG_10257.6 | P131_scaffold01335-8 | Y34_scaffold00867-4 | start codon, intron |
| MGG_10266.6 | P131_scaffold00271-1 | Y34_scaffold00508-2 | start codon |
| MGG_10327.6 | P131_scaffold01088-14 | Y34_scaffold00937-7 | start codon, intron |
| MGG_10544.6 | P131_scaffold01133-5 | Y34_scaffold00678-8 | start codon, intron |
| MGG_10579.6 | P131_scaffold00344-22 | Y34_scaffold00334-29 | start codon |
| MGG_10581.6 | P131_scaffold00344-20 | Y34_scaffold00334-27 | start codon |
| MGG_10618.6 | P131_scaffold01697-23 | Y34_scaffold00498-23 | start codon |
| MGG_10737.6 | P131_scaffold01689-15 | Y34_scaffold00679-14 | start codon |
| MGG_10799.6 | P131_scaffold00610-15 | Y34_scaffold00109-19 | intron |
| MGG_10853.6 | P131_scaffold01380-11 | Y34_scaffold00193-9 | intron |
| MGG_10911.6 | P131_scaffold00743-5 | Y34_scaffold00783-9 | start codon |
| MGG_10932.6 | P131_scaffold00102-8 | Y34_scaffold00969-8 | stop codon |
| MGG_11067.6 | P131_scaffold00755-2 | Y34_scaffold00905-2 | start codon |
| MGG_11261.6 | P131_scaffold00497-1 | Y34_scaffold00594-11 | intron |
| MGG_11311.6 | P131_scaffold00625-10 | Y34_scaffold00240-12 | start codon, intron |
| MGG_11362.6 | P131_scaffold00538-53 | Y34_scaffold00194-81 | start codon, intron |
| MGG_11371.6 | P131_scaffold00467-12 | Y34_scaffold00194-101 | start codon |
| MGG_11466.6 | P131_scaffold00345-85 | Y34_scaffold00126-117 | intron |
| MGG_11495.6 | P131_scaffold01307-13 | Y34_scaffold00153-8 | start codon |
| MGG_11526.6 | P131_scaffold00499-5 | Y34_scaffold00099-5 | start codon, intron |
| MGG_11534.6 | P131_scaffold00267-9 | Y34_scaffold00134-10 | intron |
| MGG_11820.6 | P131_scaffold01187-14 | Y34_scaffold00140-119 | intron |
| MGG_11906.6 | P131_scaffold00400-42 | Y34_scaffold00289-5 | start codon |
| MGG_11917.6 | P131_scaffold01308-4 | Y34_scaffold00686-1 | start codon |
| MGG_11998.6 | P131_scaffold00275-1 | Y34_scaffold00631-3 | start codon |
| MGG_12015.6 | P131_scaffold01043-1 | Y34_scaffold00258-3 | start codon |
| MGG_12123.6 | P131_scaffold00423-3 | Y34_scaffold00514-21 | start codon |
| MGG_12138.6 | P131_scaffold01683-41 | Y34_scaffold00514-119 | start codon |
| MGG_12184.6 | P131_scaffold00562-8 | Y34_scaffold00540-67 | intron |
| MGG_12211.6 | P131_scaffold01143-15 | Y34_scaffold00290-34 | intron |
| MGG_12345.6 | P131_scaffold01192-59 | Y34_scaffold00707-2 | start codon |
| MGG_12551.6 | P131_scaffold00388-20 | Y34_scaffold00199-6 | intron |
| MGG_12568.6 | P131_scaffold00941-9 | Y34_scaffold00458-53 | start codon |
| MGG_12612.6 | P131_scaffold00372-5 | Y34_scaffold00666-102 | start codon |
| MGG_12615.6 | P131_scaffold00124-4 | Y34_scaffold00666-110 | start codon |
| MGG_12869.6 | P131_scaffold01189-5 | Y34_scaffold00748-39 | start codon, intron |
| MGG_12949.6 | P131_scaffold00266-22 | Y34_scaffold00174-70 | intron |
| MGG_12973.6 | P131_scaffold00314-22 | Y34_scaffold00745-50 | start codon |
| MGG_13001.6 | P131_scaffold00247-3 | Y34_scaffold00548-28 | start codon |
| MGG_13006.6 | P131_scaffold00142-13 | Y34_scaffold00548-73 | start codon |
| MGG_13079.6 | P131_scaffold00295-9 | Y34_scaffold00445-9 | start codon, intron |
| MGG_13119.6 | P131_scaffold01004-4 | Y34_scaffold00277-10 | intron |
| MGG_13161.6 | P131_scaffold01338-60 | Y34_scaffold00567-29 | start codon |
| MGG_13171.6 | P131_scaffold01338-27 | Y34_scaffold00567-59 | intron |
| MGG_13187.6 | P131_scaffold00455-47 | Y34_scaffold00192-45 | start codon |
| MGG_13269.6 | P131_scaffold00688-2 | Y34_scaffold00413-2 | start codon |
| MGG_13409.6 | P131_scaffold01198-41 | Y34_scaffold00552-27 | start codon, intron |
| MGG_13420.6 | P131_scaffold00961-3 | Y34_scaffold00132-9 | start codon |
| MGG_13426.6 | P131_scaffold01451-3 | Y34_scaffold01054-3 | stop codon, intron |
| MGG_13442.6 | P131_scaffold01139-5 | Y34_scaffold00719-14 | start codon |
| MGG_13474.6 | P131_scaffold00378-31 | Y34_scaffold00542-56 | start codon |
| MGG_13486.6 | P131_scaffold01168-5 | Y34_scaffold00161-8 | start codon |
| MGG_13498.6 | P131_scaffold01320-22 | Y34_scaffold00654-22 | start codon, intron |
| MGG_13562.6 | P131_scaffold00330-8 | Y34_scaffold00502-9 | start codon, intron |
| MGG_13646.6 | P131_scaffold00943-18 | Y34_scaffold00720-4 | start codon |
| MGG_13656.6 | P131_scaffold01291-18 | Y34_scaffold01003-23 | start codon |
| MGG_13805.6 | P131_scaffold01138-52 | Y34_scaffold01005-25 | intron |
| MGG_14045.6 | P131_scaffold01008-9 | Y34_scaffold00412-6 | start codon |
| MGG_14069.6 | P131_scaffold01289-18 | Y34_scaffold00535-22 | start codon |
| MGG_14071.6 | P131_scaffold01289-12 | Y34_scaffold00535-16 | start codon |
| MGG_14136.6 | P131_scaffold00515-9 | Y34_scaffold00312-9 | start codon, intron |
| MGG_14202.6 | P131_scaffold01615-10 | Y34_scaffold00916-10 | start codon |
| MGG_14205.6 | P131_scaffold01615-11 | Y34_scaffold00916-9 | start codon |
| MGG_14580.6 | P131_scaffold00546-25 | Y34_scaffold00712-25 | start codon |
| MGG_14583.6 | P131_scaffold00949-11 | Y34_scaffold00836-3 | start codon |
| MGG_14655.6 | P131_scaffold00123-21 | Y34_scaffold00126-11 | intron |
| MGG_14749.6 | P131_scaffold00217-2 | Y34_scaffold00194-2 | start codon |
| MGG_14754.6 | P131_scaffold00399-28 | Y34_scaffold00228-26 | start codon |
| MGG_14763.6 | P131_scaffold01214-38 | Y34_scaffold00240-35 | start codon, intron |
| MGG_14768.6 | P131_scaffold01561-3 | Y34_scaffold00550-2 | start codon, intron |
| MGG_14773.6 | P131_scaffold00420-8 | Y34_scaffold00207-13 | start codon |
| MGG_14840.6 | P131_scaffold01131-7 | Y34_scaffold00091-6 | start codon, intron |
| MGG_14905.6 | P131_scaffold01393-51 | Y34_scaffold00540-11 | intron |
| MGG_14927.6 | P131_scaffold00477-6 | Y34_scaffold00180-2 | stop codon, intron |
| MGG_14986.6 | P131_scaffold01007-7 | Y34_scaffold00275-11 | start codon |
| MGG_15039.6 | P131_scaffold01299-12 | Y34_scaffold00308-40 | stop codon, intron |
| MGG_15043.6 | P131_scaffold00968-2 | Y34_scaffold00821-2 | start codon, intron |
| MGG_15086.6 | P131_scaffold01066-41 | Y34_scaffold00597-13 | start codon, intron |
| MGG_15159.6 | P131_scaffold00130-12 | Y34_scaffold00624-58 | start codon |
| MGG_15194.6 | P131_scaffold01585-14 | Y34_scaffold01081-14 | start codon |
| MGG_15203.6 | P131_scaffold01326-22 | Y34_scaffold00552-108 | start codon |
| MGG_15218.6 | P131_scaffold01103-13 | Y34_scaffold00480-6 | start codon |
| MGG_15245.6 | P131_scaffold00984-32 | Y34_scaffold00287-1 | start codon |
| MGG_15284.6 | P131_scaffold01320-17 | Y34_scaffold00654-17 | start codon |
| MGG_15287.6 | P131_scaffold01168-36 | Y34_scaffold00542-23 | start codon |
| MGG_15290.6 | P131_scaffold00378-17 | Y34_scaffold00542-69 | start codon |
| MGG_15304.6 | P131_scaffold01211-6 | Y34_scaffold00500-30 | start codon |
| MGG_15324.6 | P131_scaffold00974-9 | Y34_scaffold00584-8 | start codon |
| MGG_15329.6 | P131_scaffold00263-7 | Y34_scaffold00261-6 | start codon, intron |
| MGG_15340.6 | P131_scaffold00062-10 | Y34_scaffold00927-10 | intron |
| MGG_15342.6 | P131_scaffold00062-21 | Y34_scaffold00927-21 | stop codon, intron |
| MGG_15427.6 | P131_scaffold01672-10 | Y34_scaffold00997-10 | stop codon, intron |
| MGG_15428.6 | P131_scaffold01672-11 | Y34_scaffold00997-11 | start codon |
