# Supplementary material for: Comparative Analysis of the Genomes of Two Field Isolates of the Rice Blast Fungus Magnaporthe oryzae
Source: PLoS Genet. 2012 Aug 2;8(8):e1002869. doi: 10.1371/journal.pgen.1002869 (PMC3410873; doi:10.1371/journal.pgen.1002869)
Supplement: Table S3 — Genes that were absent in 70-15 version 6 but identified by comparative analysis with the genomes of P131 and Y34, and compared against GenBank NR. (DOC) [file pgen.1002869.s011.doc]

**Table S3 Genes that were absent in 70-15 version 6 but identified by comparative analysis with the genomes of P131 and Y34, and compared against GenBank NR.**

| **70-15 ortholog** | **P131 ortholog** | **Y34 ortholog** | **Protein length** | **Annotation** |
| --- | --- | --- | --- | --- |
| supercontig_6.15-561 | P131_scaffold00074-19 | Y34_scaffold00589-18 | 216 | hypothetical protein |
| supercontig_6.25-146 | P131_scaffold00152-17 | Y34_scaffold01075-35 | 55 | no match |
| supercontig_6.22-641 | P131_scaffold00159-24 | Y34_scaffold00526-34 | 24 | no match |
| supercontig_6.27-437 | P131_scaffold00195-24 | Y34_scaffold00087-4 | 171 | no match |
| supercontig_6.21-1157 | P131_scaffold00214-19 | Y34_scaffold00793-23 | 74 | no match |
| supercontig_6.21-1152 | P131_scaffold00214-24 | Y34_scaffold00793-18 | 126 | no match |
| supercontig_6.21-965 | P131_scaffold00220-36 | Y34_scaffold00608-7 | 47 | no match |
| supercontig_6.27-958 | P131_scaffold00252-5 | Y34_scaffold00590-6 | 40 | no match |
| supercontig_6.18-224 | P131_scaffold00255-14 | Y34_scaffold00649-28 | 61 | no match |
| supercontig_6.18-206 | P131_scaffold00255-31 | Y34_scaffold00283-107 | 70 | no match |
| supercontig_6.18-192 | P131_scaffold00255-45 | Y34_scaffold00283-93 | 42 | no match |
| supercontig_6.22-405 | P131_scaffold00266-26 | Y34_scaffold00174-66 | 37 | no match |
| supercontig_6.22-367 | P131_scaffold00266-64 | Y34_scaffold00174-27 | 48 | no match |
| supercontig_6.18-1232 | P131_scaffold00290-12 | Y34_scaffold00165-11 | 41 | no match |
| supercontig_6.15-338 | P131_scaffold00294-16 | Y34_scaffold00576-25 | 42 | no match |
| supercontig_6.15-332 | P131_scaffold00294-22 | Y34_scaffold00576-31 | 77 | no match |
| supercontig_6.18-365 | P131_scaffold00328-40 | Y34_scaffold00140-39 | 41 | no match |
| supercontig_6.26-87 | P131_scaffold00333-20 | Y34_scaffold00448-44 | 85 | no match |
| supercontig_6.18-693 | P131_scaffold00343-31 | Y34_scaffold00533-2 | 60 | no match |
| supercontig_6.18-723 | P131_scaffold00343-61 | Y34_scaffold00533-32 | 47 | no match |
| supercontig_6.8-10 | P131_scaffold00344-3 | Y34_scaffold00334-10 | 57 | no match |
| supercontig_6.21-324 | P131_scaffold00345-34 | Y34_scaffold00126-65 | 85 | no match |
| supercontig_6.21-320 | P131_scaffold00345-38 | Y34_scaffold00126-69 | 80 | no match |
| supercontig_6.15-137 | P131_scaffold00366-1 | Y34_scaffold00467-5 | 167 | hypothetical protein |
| supercontig_6.24-532 | P131_scaffold00373-1 | Y34_scaffold00986-1 | 31 | no match |
| supercontig_6.18-524 | P131_scaffold00408-13 | Y34_scaffold00686-5 | 78 | no match |
| supercontig_6.27-805 | P131_scaffold00426-5 | Y34_scaffold00207-36 | 28 | no match |
| supercontig_6.24-57 | P131_scaffold00455-37 | Y34_scaffold00192-35 | 28 | no match |
| supercontig_6.18-729 | P131_scaffold00480-10 | Y34_scaffold00533-38 | 38 | no match |
| supercontig_6.13-511 | P131_scaffold00505-13 | Y34_scaffold00095-8 | 45 | no match |
| supercontig_6.27-941 | P131_scaffold00507-2 | Y34_scaffold00245-13 | 92 | no match |
| supercontig_6.18-644 | P131_scaffold00546-19 | Y34_scaffold00712-19 | 56 | no match |
| supercontig_6.13-452 | P131_scaffold00568-6 | Y34_scaffold00559-11 | 84 | no match |
| supercontig_6.24-354 | P131_scaffold00688-3 | Y34_scaffold00413-3 | 55 | no match |
| supercontig_6.25-256 | P131_scaffold00875-2 | Y34_scaffold00978-2 | 97 | no match |
| supercontig_6.21-1372 | P131_scaffold00916-23 | Y34_scaffold00217-12 | 48 | no match |
| supercontig_6.13-105 | P131_scaffold00943-17 | Y34_scaffold00720-5 | 23 | no match |
| supercontig_6.4-80 | P131_scaffold00973-1 | Y34_scaffold00641-75 | 32 | no match |
| supercontig_6.4-89 | P131_scaffold00973-10 | Y34_scaffold00641-66 | 48 | no match |
| supercontig_6.22-527 | P131_scaffold00982-39 | Y34_scaffold00516-35 | 31 | no match |
| supercontig_6.28-43 | P131_scaffold00984-15 | Y34_scaffold00287-18 | 41 | no match |
| supercontig_6.27-667 | P131_scaffold01028-2 | Y34_scaffold00476-5 | 28 | no match |
| supercontig_6.27-694 | P131_scaffold01028-30 | Y34_scaffold00476-33 | 35 | no match |
| supercontig_6.29-190 | P131_scaffold01039-34 | Y34_scaffold00610-24 | 26 | no match |
| supercontig_6.18-860 | P131_scaffold01049-12 | Y34_scaffold00534-88 | 76 | hypothetical protein |
| supercontig_6.27-829 | P131_scaffold01058-2 | Y34_scaffold00772-2 | 69 | no match |
| supercontig_6.27-748 | P131_scaffold01065-12 | Y34_scaffold00594-13 | 22 | no match |
| supercontig_6.18-1426 | P131_scaffold01083-6 | Y34_scaffold00414-8 | 59 | no match |
| supercontig_6.21-247 | P131_scaffold01094-11 | Y34_scaffold00832-10 | 39 | no match |
| supercontig_6.21-254 | P131_scaffold01094-4 | Y34_scaffold00832-3 | 144 | no match |
| supercontig_6.22-466 | P131_scaffold01120-2 | Y34_scaffold00510-9 | 38 | no match |
| supercontig_6.16-120 | P131_scaffold01138-72 | Y34_scaffold01005-2 | 76 | hypothetical protein |
| supercontig_6.18-969 | P131_scaffold01156-3 | Y34_scaffold00908-3 | 124 | no match |
| supercontig_6.18-1013 | P131_scaffold01164-7 | Y34_scaffold00164-8 | 39 | no match |
| supercontig_6.29-360 | P131_scaffold01179-35 | Y34_scaffold00773-16 | 34 | no match |
| supercontig_6.18-177 | P131_scaffold01182-3 | Y34_scaffold00283-78 | 27 | no match |
| supercontig_6.29-263 | P131_scaffold01192-5 | Y34_scaffold00707-57 | 121 | no match |
| supercontig_6.18-74 | P131_scaffold01201-26 | Y34_scaffold00648-25 | 72 | no match |
| supercontig_6.21-558 | P131_scaffold01213-12 | Y34_scaffold00669-11 | 23 | no match |
| supercontig_6.21-554 | P131_scaffold01213-16 | Y34_scaffold00669-15 | 64 | no match |
| supercontig_6.21-527 | P131_scaffold01213-43 | Y34_scaffold00669-42 | 57 | no match |
| supercontig_6.21-524 | P131_scaffold01213-46 | Y34_scaffold00669-45 | 102 | no match |
| supercontig_6.21-518 | P131_scaffold01213-52 | Y34_scaffold00669-51 | 37 | no match |
| supercontig_6.22-110 | P131_scaffold01234-1 | Y34_scaffold00511-22 | 58 | no match |
| supercontig_6.27-1287 | P131_scaffold01287-5 | Y34_scaffold00162-77 | 51 | no match |
| supercontig_6.13-30 | P131_scaffold01291-17 | Y34_scaffold01003-24 | 116 | no match |
| supercontig_6.13-20 | P131_scaffold01291-7 | Y34_scaffold01003-34 | 45 | no match |
| supercontig_6.23-227 | P131_scaffold01310-16 | Y34_scaffold00458-28 | 124 | no match |
| supercontig_6.12-732 | P131_scaffold01314-12 | Y34_scaffold00658-2 | 64 | no match |
| supercontig_6.15-163 | P131_scaffold01321-1 | Y34_scaffold00771-1 | 67 | no match |
| supercontig_6.21-109 | P131_scaffold01325-33 | Y34_scaffold00496-52 | 46 | no match |
| supercontig_6.21-140 | P131_scaffold01325-63 | Y34_scaffold00496-22 | 81 | no match |
| supercontig_6.12-574 | P131_scaffold01331-36 | Y34_scaffold00487-6 | 72 | no match |
| supercontig_6.26-156 | P131_scaffold01337-4 | Y34_scaffold00662-25 | 113 | no match |
| supercontig_6.26-192 | P131_scaffold01337-41 | Y34_scaffold00655-39 | 98 | hypothetical protein |
| supercontig_6.27-204 | P131_scaffold01358-81 | Y34_scaffold00619-43 | 71 | no match |
| supercontig_6.13-477 | P131_scaffold01365-8 | Y34_scaffold00095-41 | 68 | no match |
| supercontig_6.13-1116 | P131_scaffold01377-8 | Y34_scaffold00726-61 | 28 | no match |
| supercontig_6.13-643 | P131_scaffold01381-13 | Y34_scaffold00247-60 | 56 | no match |
| supercontig_6.13-654 | P131_scaffold01381-3 | Y34_scaffold00247-71 | 30 | no match |
| supercontig_6.12-216 | P131_scaffold01393-1 | Y34_scaffold00255-12 | 40 | no match |
