# Supplementary material for: Comparative Analysis of the Genomes of Two Field Isolates of the Rice Blast Fungus Magnaporthe oryzae
Source: PLoS Genet. 2012 Aug 2;8(8):e1002869. doi: 10.1371/journal.pgen.1002869 (PMC3410873; doi:10.1371/journal.pgen.1002869)
Supplement: Table S4 — Isolate-unique genes in P131, Y34, and 70-15. (DOC) [file pgen.1002869.s012.doc]

**Table S4** Isolate-unique genes in P131, Y34, and 70-15.

| **Gene** | **Isolate** | **Annotation** | **Secreted** | **TM** | **NLS** |
| --- | --- | --- | --- | --- | --- |
| P131_scaffold00003-2 | P131 | no match | NO | 0 | NO |
| P131_scaffold00006-1 | P131 | predicted protein | NO | 0 | NO |
| P131_scaffold00006-2 | P131 | HD domain containing adenylate kinase | NO | 0 | YES |
| P131_scaffold00006-6 | P131 | no match | NO | 0 | YES |
| P131_scaffold00006-7 | P131 | hypothetical protein | NO | 3 | NO |
| P131_scaffold00010-1 | P131 | no match | YES | 0 | NO |
| P131_scaffold00075-1 | P131 | no match | NO | 0 | NO |
| P131_scaffold00087-1 | P131 | hypothetical protein | NO | 0 | NO |
| P131_scaffold00087-3 | P131 | hypothetical protein | NO | 0 | NO |
| P131_scaffold00087-5 | P131 | no match | NO | 0 | NO |
| P131_scaffold00101-1 | P131 | no match | NO | 0 | NO |
| P131_scaffold00208-2 | P131 | no match | NO | 0 | NO |
| P131_scaffold00208-3 | P131 | zinc finger protein | NO | 0 | NO |
| P131_scaffold00513-4 | P131 | hypothetical protein | NO | 0 | YES |
| P131_scaffold00563-1 | P131 | no match | YES | 0 | NO |
| P131_scaffold00581-4 | P131 | no match | NO | 0 | NO |
| P131_scaffold00620-1 | P131 | no match | NO | 0 | NO |
| P131_scaffold00620-2 | P131 | hypothetical protein | NO | 0 | NO |
| P131_scaffold00980-1 | P131 | NmrA-like family protein | NO | 0 | NO |
| P131_scaffold01010-3 | P131 | no match | NO | 0 | NO |
| P131_scaffold01042-2 | P131 | no match | NO | 0 | YES |
| P131_scaffold01206-2 | P131 | no match | NO | 0 | NO |
| P131_scaffold01269-1 | P131 | no match | NO | 0 | NO |
| P131_scaffold01323-1 | P131 | no match | YES | 0 | NO |
| P131_scaffold01377-24 | P131 | no match | YES | 3 | NO |
| P131_scaffold01393-42 | P131 | no match | NO | 0 | NO |
| P131_scaffold01474-3 | P131 | no match | NO | 0 | NO |
| P131_scaffold01475-1 | P131 | no match | NO | 0 | NO |
| P131_scaffold01477-1 | P131 | no match | NO | 0 | NO |
| P131_scaffold01477-2 | P131 | no match | NO | 0 | NO |
| P131_scaffold01491-3 | P131 | transposase | NO | 0 | NO |
| P131_scaffold01531-1 | P131 | predicted protein | NO | 0 | NO |
| P131_scaffold01540-2 | P131 | hypothetical protein | NO | 0 | NO |
| P131_scaffold01579-6 | P131 | no match | NO | 0 | NO |
| P131_scaffold01579-7 | P131 | no match | NO | 0 | NO |
| P131_scaffold01677-4 | P131 | acetyltransferase | NO | 0 | NO |
| P131_scaffold01677-5 | P131 | no match | NO | 0 | NO |
| P131_scaffold01678-5 | P131 | no match | NO | 0 | NO |
| P131_scaffold01711-1 | P131 | hypothetical protein | NO | 0 | NO |
| P131_scaffold01726-1 | P131 | hypothetical protein | NO | 0 | NO |
| P131_scaffold01742-1 | P131 | predicted protein | NO | 0 | NO |
| P131_scaffold01754-3 | P131 | Pfs, NACHT, and Ankyrin domain protein | NO | 0 | NO |
| P131_scaffold01754-4 | P131 | no match | YES | 0 | NO |
| P131_scaffold01777-6 | P131 | hypothetical protein | NO | 0 | NO |
| P131_scaffold01777-7 | P131 | no match | NO | 0 | YES |
| P131_scaffold01777-8 | P131 | no match | NO | 0 | NO |
| P131_scaffold01784-1 | P131 | ankyrin repeat domain containing protein | NO | 0 | NO |
| P131_scaffold01784-2 | P131 | no match | NO | 0 | NO |
| P131_scaffold01784-3 | P131 | hypothetical protein | NO | 1 | NO |
| P131_scaffold01812-1 | P131 | hypothetical protein | NO | 0 | NO |
| P131_scaffold01822-1 | P131 | predicted protein | YES | 2 | NO |
| Y34_scaffold00005-1 | Y34 | hypothetical protein | NO | 0 | NO |
| Y34_scaffold00017-1 | Y34 | no match | NO | 0 | NO |
| Y34_scaffold00079-1 | Y34 | no match | NO | 0 | NO |
| Y34_scaffold00079-3 | Y34 | predicted protein | NO | 0 | NO |
| Y34_scaffold00079-4 | Y34 | no match | NO | 0 | NO |
| Y34_scaffold00086-1 | Y34 | no match | NO | 0 | NO |
| Y34_scaffold00104-5 | Y34 | hypothetical protein | NO | 0 | NO |
| Y34_scaffold00105-1 | Y34 | FAD binding domain containing protein | YES | 0 | NO |
| Y34_scaffold00105-2 | Y34 | hypothetical protein | NO | 6 | NO |
| Y34_scaffold00110-1 | Y34 | hypothetical protein | NO | 0 | NO |
| Y34_scaffold00116-2 | Y34 | hypothetical protein | NO | 0 | NO |
| Y34_scaffold00116-5 | Y34 | no match | NO | 0 | NO |
| Y34_scaffold00170-1 | Y34 | hypothetical protein | NO | 0 | NO |
| Y34_scaffold00170-3 | Y34 | no match | NO | 0 | NO |
| Y34_scaffold00242-2 | Y34 | C6 zinc finger domain containing protein | NO | 0 | NO |
| Y34_scaffold00252-6 | Y34 | no match | NO | 0 | NO |
| Y34_scaffold00252-7 | Y34 | no match | YES | 0 | NO |
| Y34_scaffold00305-3 | Y34 | no match | NO | 0 | NO |
| Y34_scaffold00316-2 | Y34 | LipA and NB-ARC domain protein | NO | 0 | NO |
| Y34_scaffold00335-1 | Y34 | phospholipase | NO | 0 | YES |
| Y34_scaffold00346-1 | Y34 | no match | NO | 0 | NO |
| Y34_scaffold00375-2 | Y34 | no match | NO | 1 | NO |
| Y34_scaffold00439-1 | Y34 | hypothetical protein | NO | 0 | NO |
| Y34_scaffold00439-2 | Y34 | SUMO1/sentrin specific protease | NO | 0 | NO |
| Y34_scaffold00440-1 | Y34 | no match | NO | 0 | NO |
| Y34_scaffold00479-1 | Y34 | no match | NO | 0 | NO |
| Y34_scaffold00479-2 | Y34 | predicted protein | NO | 0 | NO |
| Y34_scaffold00479-3 | Y34 | YD repeat protein | NO | 4 | NO |
| Y34_scaffold00499-3 | Y34 | hypothetical protein | NO | 0 | NO |
| Y34_scaffold00540-2 | Y34 | no match | NO | 0 | NO |
| Y34_scaffold00582-1 | Y34 | no match | NO | 0 | NO |
| Y34_scaffold00582-3 | Y34 | AAA family ATPase | NO | 0 | NO |
| Y34_scaffold00588-1 | Y34 | TPR repeat-containing protein | NO | 0 | NO |
| Y34_scaffold00588-6 | Y34 | hypothetical protein | NO | 0 | NO |
| Y34_scaffold00588-7 | Y34 | hypothetical protein | YES | 1 | NO |
| Y34_scaffold00600-1 | Y34 | no match | NO | 0 | NO |
| Y34_scaffold00605-2 | Y34 | no match | NO | 0 | NO |
| Y34_scaffold00605-5 | Y34 | no match | NO | 0 | NO |
| Y34_scaffold00623-1 | Y34 | no match | YES | 0 | YES |
| Y34_scaffold00661-1 | Y34 | no match | NO | 0 | NO |
| Y34_scaffold00661-2 | Y34 | sarcosine oxidase | NO | 0 | YES |
| Y34_scaffold00664-1 | Y34 | predicted protein | YES | 0 | NO |
| Y34_scaffold00664-2 | Y34 | no match | NO | 0 | NO |
| Y34_scaffold00664-3 | Y34 | no match | NO | 0 | NO |
| Y34_scaffold00708-1 | Y34 | hypothetical protein | NO | 0 | NO |
| Y34_scaffold00714-4 | Y34 | P450 domain containing protein | YES | 1 | NO |
| Y34_scaffold00714-5 | Y34 | transcription factor | NO | 0 | NO |
| Y34_scaffold00714-6 | Y34 | no match | NO | 0 | NO |
| Y34_scaffold00714-7 | Y34 | geranylgeranyl diphosphate synthase | NO | 0 | NO |
| Y34_scaffold00780-1 | Y34 | no match | NO | 0 | NO |
| Y34_scaffold00820-1 | Y34 | AGC kinase | NO | 0 | NO |
| Y34_scaffold00820-7 | Y34 | no match | YES | 0 | YES |
| Y34_scaffold00824-1 | Y34 | tankyrase | NO | 0 | NO |
| Y34_scaffold00824-3 | Y34 | SET domain containing protein | NO | 0 | NO |
| Y34_scaffold00824-5 | Y34 | no match | NO | 1 | NO |
| Y34_scaffold00826-3 | Y34 | no match | NO | 0 | NO |
| Y34_scaffold00831-1 | Y34 | putative transposase | NO | 0 | NO |
| Y34_scaffold00831-4 | Y34 | FtsJ-like methyltransferase family protein | NO | 0 | NO |
| Y34_scaffold00855-11 | Y34 | Ankyrin domain protein | YES | 0 | NO |
| Y34_scaffold00855-12 | Y34 | no match | NO | 0 | NO |
| Y34_scaffold00857-6 | Y34 | similar to ankyrin 2,3/unc44 | NO | 0 | NO |
| Y34_scaffold00870-4 | Y34 | no match | NO | 0 | NO |
| Y34_scaffold00874-1 | Y34 | no match | NO | 0 | NO |
| Y34_scaffold00875-1 | Y34 | P450 domain containing protein | YES | 1 | NO |
| Y34_scaffold00875-2 | Y34 | multi-functional prenyl transferase | NO | 0 | NO |
| Y34_scaffold00875-3 | Y34 | fusicoccadiene 8-ol C-15 hydroxylase | NO | 1 | NO |
| Y34_scaffold00875-4 | Y34 | fusicoccadiene synthase | NO | 0 | NO |
| Y34_scaffold00875-6 | Y34 | hypothetical protein | NO | 0 | NO |
| Y34_scaffold00876-1 | Y34 | no match | NO | 0 | NO |
| Y34_scaffold00877-2 | Y34 | no match | NO | 0 | NO |
| Y34_scaffold00877-6 | Y34 | no match | NO | 0 | NO |
| Y34_scaffold00877-7 | Y34 | no match | NO | 0 | NO |
| Y34_scaffold00944-1 | Y34 | no match | NO | 0 | NO |
| Y34_scaffold00964-1 | Y34 | hypothetical protein | NO | 0 | NO |
| Y34_scaffold00964-5 | Y34 | no match | NO | 1 | NO |
| Y34_scaffold00967-1 | Y34 | hypothetical protein | NO | 0 | NO |
| Y34_scaffold00982-1 | Y34 | no match | NO | 0 | NO |
| Y34_scaffold00982-5 | Y34 | no match | NO | 0 | NO |
| Y34_scaffold00982-6 | Y34 | C2H2 transcription factor | NO | 0 | NO |
| Y34_scaffold01006-1 | Y34 | hypothetical protein | NO | 0 | NO |
| Y34_scaffold01009-1 | Y34 | no match | NO | 0 | NO |
| Y34_scaffold01009-2 | Y34 | hypothetical protein | NO | 0 | NO |
| Y34_scaffold01037-2 | Y34 | hypothetical protein | NO | 0 | NO |
| Y34_scaffold01039-4 | Y34 | no match | NO | 0 | NO |
| Y34_scaffold01039-5 | Y34 | no match | NO | 0 | NO |
| Y34_scaffold01040-2 | Y34 | no match | NO | 0 | NO |
| Y34_scaffold01048-1 | Y34 | no match | NO | 0 | NO |
| Y34_scaffold01048-2 | Y34 | polyketide synthase | NO | 0 | NO |
| Y34_scaffold01093-2 | Y34 | no match | NO | 0 | NO |
| Y34_scaffold01093-3 | Y34 | hypothetical protein | NO | 0 | NO |
| Y34_scaffold01093-4 | Y34 | hypothetical protein | NO | 0 | NO |
| Y34_scaffold01093-5 | Y34 | no match | NO | 0 | NO |
| Y34_scaffold01097-1 | Y34 | no match | NO | 0 | NO |
| Y34_scaffold01098-1 | Y34 | no match | NO | 1 | NO |
| Y34_scaffold01101-3 | Y34 | predicted protein | NO | 0 | NO |
| Y34_scaffold01103-1 | Y34 | hypothetical protein | NO | 0 | NO |
| Y34_scaffold01110-1 | Y34 | hypothetical protein | NO | 0 | NO |
| Y34_scaffold01112-1 | Y34 | no match | NO | 0 | NO |
| Y34_scaffold01112-10 | Y34 | no match | NO | 0 | NO |
| Y34_scaffold01112-4 | Y34 | no match | NO | 0 | NO |
| Y34_scaffold01112-5 | Y34 | NADH:ubiquinone oxidoreductase subunit 1 | YES | 1 | NO |
| Y34_scaffold01112-6 | Y34 | no match | NO | 0 | NO |
| Y34_scaffold01112-7 | Y34 | no match | NO | 0 | NO |
| Y34_scaffold01112-8 | Y34 | probable intron-encoded endonuclease bI1 | NO | 0 | NO |
| Y34_scaffold01112-9 | Y34 | no match | NO | 0 | NO |
| Y34_scaffold01119-1 | Y34 | heat shock transcription factor 4 | NO | 0 | NO |
| Y34_scaffold01121-1 | Y34 | ATP-dependent RNA helicase A-like protein | NO | 0 | NO |
| Y34_scaffold01123-1 | Y34 | chitin deacetylase | YES | 0 | NO |
| Y34_scaffold01123-2 | Y34 | hypothetical protein | NO | 0 | NO |
| Y34_scaffold01124-1 | Y34 | no match | YES | 1 | NO |
| Y34_scaffold01125-1 | Y34 | copper amine oxidase | NO | 0 | NO |
| Y34_scaffold01130-1 | Y34 | hypothetical protein | NO | 0 | NO |
| Y34_scaffold01134-1 | Y34 | hypothetical protein | NO | 0 | NO |
| Y34_scaffold01138-1 | Y34 | hypothetical protein | NO | 0 | NO |
| Y34_scaffold01143-1 | Y34 | hypothetical protein | YES | 0 | NO |
| Y34_scaffold01145-1 | Y34 | TPR domain protein | NO | 0 | NO |
| Y34_scaffold01152-4 | Y34 | no match | NO | 0 | NO |
| Y34_scaffold01152-6 | Y34 | no match | NO | 0 | NO |
| Y34_scaffold01167-1 | Y34 | no match | NO | 0 | NO |
| Y34_scaffold01167-2 | Y34 | no match | NO | 0 | NO |
| Y34_scaffold01167-4 | Y34 | ulp1 protease family protein | NO | 0 | NO |
| Y34_scaffold01171-3 | Y34 | no match | NO | 0 | NO |
| Y34_scaffold01171-4 | Y34 | no match | NO | 0 | NO |
| Y34_scaffold01171-5 | Y34 | no match | NO | 0 | NO |
| Y34_scaffold01177-1 | Y34 | no match | NO | 0 | NO |
| Y34_scaffold01182-1 | Y34 | no match | NO | 0 | NO |
| Y34_scaffold01184-1 | Y34 | no match | NO | 0 | NO |
| Y34_scaffold01184-5 | Y34 | no match | NO | 0 | NO |
| Y34_scaffold01184-7 | Y34 | no match | NO | 0 | NO |
| Y34_scaffold01190-1 | Y34 | no match | NO | 0 | NO |
| Y34_scaffold01191-3 | Y34 | no match | NO | 0 | NO |
| Y34_scaffold01191-4 | Y34 | M-phase inducer phosphatase | NO | 0 | NO |
| Y34_scaffold01193-1 | Y34 | alpha-ketoglutarate dependent xanthine dioxygenase | NO | 0 | NO |
| Y34_scaffold01193-2 | Y34 | P450 domain containing protein | NO | 0 | NO |
| Y34_scaffold01193-3 | Y34 | capsule polysaccharide biosynthesis protein | NO | 0 | NO |
| Y34_scaffold01193-4 | Y34 | fusicoccadiene C-8 hydroxylase | NO | 1 | NO |
| supercontig_6.1-4 | 70-15 | no match | NO | 0 | NO |
| supercontig_6.10-108 | 70-15 | hypothetical protein | YES | 0 | NO |
| supercontig_6.10-135 | 70-15 | hypothetical protein | NO | 0 | NO |
| supercontig_6.10-280 | 70-15 | hypothetical protein | YES | 0 | NO |
| supercontig_6.11-73 | 70-15 | hypothetical protein | NO | 0 | NO |
| supercontig_6.12-607 | 70-15 | multidrug resistance protein fnx1 | NO | 11 | NO |
| supercontig_6.12-608 | 70-15 | P450 domain containing protein | YES | 1 | NO |
| supercontig_6.12-634 | 70-15 | pfs domain containing protein | NO | 0 | NO |
| supercontig_6.12-640 | 70-15 | no match | NO | 1 | NO |
| supercontig_6.12-66 | 70-15 | hypothetical protein | NO | 0 | NO |
| supercontig_6.12-669 | 70-15 | hypothetical protein | NO | 0 | NO |
| supercontig_6.12-67 | 70-15 | hypothetical protein | YES | 0 | NO |
| supercontig_6.12-991 | 70-15 | hypothetical protein | YES | 0 | NO |
| supercontig_6.12-992 | 70-15 | monooxygenase | YES | 0 | NO |
| supercontig_6.12-993 | 70-15 | no match | YES | 0 | NO |
| supercontig_6.13-216 | 70-15 | no match | NO | 0 | NO |
| supercontig_6.13-339 | 70-15 | hypothetical protein | NO | 0 | YES |
| supercontig_6.15-68 | 70-15 | hypothetical protein | YES | 7 | NO |
| supercontig_6.15-69 | 70-15 | hypothetical protein | NO | 0 | NO |
| supercontig_6.16-6 | 70-15 | hypothetical protein | NO | 0 | NO |
| supercontig_6.17-44 | 70-15 | hypothetical protein | NO | 0 | NO |
| supercontig_6.17-45 | 70-15 | no match | NO | 0 | NO |
| supercontig_6.17-48 | 70-15 | no match | NO | 0 | NO |
| supercontig_6.17-52 | 70-15 | phosphotransferase family protein | NO | 0 | NO |
| supercontig_6.17-54 | 70-15 | hypothetical protein | NO | 0 | NO |
| supercontig_6.18-1098 | 70-15 | no match | NO | 0 | NO |
| supercontig_6.18-1185 | 70-15 | hypothetical protein | NO | 0 | NO |
| supercontig_6.18-509 | 70-15 | no match | NO | 0 | NO |
| supercontig_6.18-877 | 70-15 | no match | NO | 0 | NO |
| supercontig_6.18-900 | 70-15 | sugar transporter | NO | 10 | NO |
| supercontig_6.18-918 | 70-15 | no match | NO | 0 | NO |
| supercontig_6.20-527 | 70-15 | no match | NO | 0 | NO |
| supercontig_6.20-531 | 70-15 | beta-galactosidase | NO | 0 | NO |
| supercontig_6.21-1324 | 70-15 | no match | NO | 0 | NO |
| supercontig_6.24-462 | 70-15 | hypothetical protein | YES | 0 | NO |
| supercontig_6.24-489 | 70-15 | hypothetical protein | NO | 0 | NO |
| supercontig_6.24-490 | 70-15 | no match | NO | 0 | NO |
| supercontig_6.24-491 | 70-15 | hypothetical protein | NO | 7 | NO |
| supercontig_6.24-492 | 70-15 | no match | NO | 0 | NO |
| supercontig_6.24-493 | 70-15 | cutinase | YES | 0 | NO |
| supercontig_6.24-494 | 70-15 | ADP-heptose: LPS heptosyltransferase-like protein | YES | 1 | NO |
| supercontig_6.26-144 | 70-15 | no match | NO | 0 | NO |
| supercontig_6.27-1364 | 70-15 | beta-lactamase | NO | 0 | NO |
| supercontig_6.27-641 | 70-15 | hypothetical protein | NO | 1 | NO |
| supercontig_6.28-148 | 70-15 | hypothetical protein | NO | 0 | NO |
| supercontig_6.28-152 | 70-15 | no match | NO | 0 | NO |
| supercontig_6.28-186 | 70-15 | hypothetical protein | YES | 0 | NO |
| supercontig_6.28-188 | 70-15 | ankyrin repeat containing protein | NO | 0 | NO |
| supercontig_6.28-265 | 70-15 | hypothetical protein | NO | 0 | NO |
| supercontig_6.28-266 | 70-15 | hypothetical protein | NO | 0 | NO |
| supercontig_6.28-267 | 70-15 | hypothetical protein | NO | 0 | NO |
| supercontig_6.28-268 | 70-15 | hypothetical protein | NO | 0 | YES |
| supercontig_6.28-269 | 70-15 | similar to retroelement pol polyprotein | NO | 0 | NO |
| supercontig_6.28-270 | 70-15 | hypothetical protein | YES | 0 | NO |
| supercontig_6.28-283 | 70-15 | hypothetical protein | NO | 0 | NO |
| supercontig_6.29-799 | 70-15 | no match | NO | 0 | NO |
| supercontig_6.29-800 | 70-15 | no match | NO | 0 | NO |
| supercontig_6.31-3 | 70-15 | no match | NO | 0 | NO |
| supercontig_6.6-13 | 70-15 | hypothetical protein | YES | 0 | NO |
| supercontig_6.6-14 | 70-15 | allantoate permease | NO | 10 | NO |
| supercontig_6.6-15 | 70-15 | hypothetical protein | YES | 0 | NO |
| supercontig_6.7-90 | 70-15 | phosphotransferase family protein | NO | 0 | NO |
| supercontig_6.7-91 | 70-15 | TPR domain containing protein | NO | 0 | NO |
| supercontig_6.7-92 | 70-15 | hypothetical protein | NO | 0 | NO |
| supercontig_6.8-155 | 70-15 | no match | NO | 0 | NO |
| supercontig_6.8-206 | 70-15 | hypothetical protein | NO | 1 | NO |
| supercontig_6.8-207 | 70-15 | hypothetical protein | NO | 0 | NO |
| supercontig_6.8-82 | 70-15 | no match | NO | 0 | NO |
| supercontig_6.9-78 | 70-15 | resolvase | NO | 0 | NO |
| supercontig_6.9-79 | 70-15 | no match | NO | 0 | YES |
| supercontig_6.9-98 | 70-15 | P450 domain containing protein | NO | 0 | NO |

Secreted, secreted proteins; TM, transmembrane domains; NLS, nuclear localization signals.
