# Supplementary material for: Comparative Analysis of the Genomes of Two Field Isolates of the Rice Blast Fungus Magnaporthe oryzae
Source: PLoS Genet. 2012 Aug 2;8(8):e1002869. doi: 10.1371/journal.pgen.1002869 (PMC3410873; doi:10.1371/journal.pgen.1002869)
Supplement: Table S5 — Genes specific to the field isolates P131 and Y34. (DOC) [file pgen.1002869.s013.doc]

**Table S5** Genes specific to the field isolates P131 and Y34.

| **Gene** | **Annotation** | **Secreted** | **TM** | **NLS** |
| --- | --- | --- | --- | --- |
| P131_scaffold01409-2 | no match | NO | 0 | YES |
| P131_scaffold01574-1 | alkyl sulfatase-related hydrolase | NO | 0 | NO |
| P131_scaffold00995-1 | no match | NO | 0 | NO |
| P131_scaffold00848-1 | no match | NO | 0 | NO |
| P131_scaffold01579-8 | no match | NO | 0 | NO |
| P131_scaffold01449-1 | no match | NO | 0 | NO |
| P131_scaffold01392-3 | no match | NO | 0 | NO |
| P131_scaffold00885-1 | no match | NO | 0 | NO |
| P131_scaffold00033-1 | kruppel-like factor 11 | NO | 0 | NO |
| P131_scaffold01235-3 | no match | NO | 0 | NO |
| P131_scaffold00587-2 | no match | NO | 0 | NO |
| P131_scaffold01164-14 | Ankyrin and HET domain protein | NO | 0 | NO |
| P131_scaffold01591-1 | no match | NO | 0 | NO |
| P131_scaffold01575-1 | no match | YES | 1 | NO |
| P131_scaffold01292-3 | hypothetical protein | NO | 0 | NO |
| P131_scaffold00611-3 | hypothetical protein | YES | 1 | NO |
| P131_scaffold01821-5 | nuclear pore complex subunit Nup133 | NO | 0 | NO |
| P131_scaffold00953-1 | no match | YES | 1 | NO |
| P131_scaffold01563-2 | no match | NO | 0 | NO |
| P131_scaffold01245-2 | CAMK family protein kinase | NO | 0 | NO |
| P131_scaffold00501-2 | hypothetical protein | NO | 0 | NO |
| P131_scaffold00259-2 | chitinase | YES | 1 | NO |
| P131_scaffold01513-4 | no match | NO | 0 | NO |
| P131_scaffold00951-7 | neutral protease | YES | 0 | NO |
| P131_scaffold00405-2 | no match | NO | 1 | NO |
| P131_scaffold01248-3 | no match | NO | 0 | NO |
| P131_scaffold00132-2 | no match | NO | 0 | NO |
| P131_scaffold01467-1 | no match | NO | 0 | NO |
| P131_scaffold01113-3 | no match | NO | 0 | NO |
| P131_scaffold01405-2 | no match | NO | 0 | NO |
| P131_scaffold01764-2 | no match | NO | 4 | NO |
| P131_scaffold00667-1 | no match | NO | 0 | NO |
| P131_scaffold00167-2 | arylsulfatase B precursor | NO | 0 | NO |
| P131_scaffold01579-12 | hypothetical protein | NO | 0 | NO |
| P131_scaffold01298-3 | thioredoxin | NO | 0 | NO |
| P131_scaffold01248-2 | hypothetical protein | NO | 0 | NO |
| P131_scaffold01510-1 | LipA and NB-ARC domain-containing protein | NO | 0 | NO |
| P131_scaffold01420-3 | hypothetical protein | NO | 5 | NO |
| P131_scaffold00099-3 | serine/threonine-protein kinase | NO | 0 | NO |
| P131_scaffold01822-3 | thymidylate kinase | YES | 0 | NO |
| P131_scaffold01579-14 | ferric-chelate reductase | NO | 2 | NO |
| P131_scaffold00279-20 | hypothetical protein | NO | 0 | NO |
| P131_scaffold00502-1 | hypothetical protein | YES | 0 | NO |
| P131_scaffold01579-3 | transmembrane protein subunit of the glycosylphosphatidylinositol transamidase complex | NO | 0 | NO |
| P131_scaffold01577-1 | CFEM domain containing protein | NO | 6 | NO |
| P131_scaffold00516-19 | no match | NO | 0 | NO |
| P131_scaffold00297-2 | no match | YES | 0 | YES |
| P131_scaffold01513-2 | calcium/calmodulin-dependent protein kinase I | NO | 0 | NO |
| P131_scaffold00773-1 | no match | NO | 0 | NO |
| P131_scaffold01265-1 | maturase K | NO | 0 | NO |
| P131_scaffold00564-2 | hypothetical protein | YES | 0 | NO |
| P131_scaffold01197-1 | NAD(P)H-dependent glutamate synthase | NO | 0 | NO |
| P131_scaffold01235-5 | osmosensitive K channel His kinase sensor | NO | 0 | NO |
| P131_scaffold01517-1 | hypothetical protein | YES | 0 | NO |
| P131_scaffold01600-1 | no match | YES | 0 | NO |
| P131_scaffold01222-1 | MFS transporter | NO | 8 | NO |
| P131_scaffold01521-1 | no match | NO | 0 | NO |
| P131_scaffold01315-4 | no match | YES | 0 | NO |
| P131_scaffold00611-6 | no match | YES | 0 | NO |
| P131_scaffold01536-1 | predicted protein | NO | 0 | YES |
| P131_scaffold01315-1 | MFS transporter | YES | 3 | NO |
| P131_scaffold00848-2 | no match | NO | 0 | NO |
| P131_scaffold01493-2 | hypothetical protein | NO | 0 | NO |
| P131_scaffold01773-2 | hypothetical protein | YES | 1 | NO |
| P131_scaffold00582-1 | no match | NO | 0 | NO |
| P131_scaffold01292-2 | hypothetical protein | NO | 2 | NO |
| P131_scaffold01643-3 | hypothetical protein | NO | 0 | NO |
| P131_scaffold01635-2 | no match | YES | 0 | NO |
| P131_scaffold01671-4 | no match | NO | 0 | NO |
| P131_scaffold00032-6 | hypothetical protein | NO | 0 | NO |
| P131_scaffold00493-1 | no match | YES | 0 | NO |
| P131_scaffold00857-4 | no match | NO | 0 | NO |
| P131_scaffold00506-12 | C6 transcription factor | NO | 1 | NO |
| P131_scaffold01577-3 | efflux pump antibiotic resistance protein | YES | 5 | NO |
| P131_scaffold00581-5 | no match | NO | 0 | NO |
| P131_scaffold00212-1 | indole-3-glycerol-phosphate synthase | NO | 1 | NO |
| P131_scaffold00851-5 | serine/threonine kinase family protein | NO | 0 | NO |
| P131_scaffold00032-1 | hypothetical protein | NO | 0 | NO |
| P131_scaffold01491-2 | no match | NO | 0 | NO |
| P131_scaffold01458-1 | no match | NO | 0 | NO |
| P131_scaffold01596-2 | phyllocladan-16a-ol synthase | NO | 0 | NO |
| P131_scaffold01292-1 | hypothetical protein | NO | 0 | NO |
| P131_scaffold01579-1 | Patatin-like serine hydrolase | NO | 0 | NO |
| P131_scaffold01773-3 | no match | NO | 3 | NO |
| P131_scaffold01574-4 | no match | NO | 0 | NO |
| P131_scaffold01643-4 | no match | NO | 0 | NO |
| P131_scaffold01528-1 | no match | NO | 0 | NO |
| P131_scaffold01514-1 | no match | NO | 0 | NO |
| P131_scaffold01704-1 | hypothetical protein | NO | 0 | NO |
| P131_scaffold00132-1 | no match | NO | 0 | YES |
| P131_scaffold01020-4 | hypothetical protein | NO | 0 | NO |
| P131_scaffold00045-33 | no match | NO | 0 | NO |
| P131_scaffold01596-1 | hypothetical protein | NO | 0 | NO |
| P131_scaffold00978-2 | addiction module toxin | NO | 0 | NO |
| P131_scaffold01563-1 | no match | YES | 0 | NO |
| P131_scaffold01136-1 | transposase | NO | 0 | NO |
| P131_scaffold00107-7 | fungal cellulose binding domain containing protein | YES | 0 | NO |
| P131_scaffold00405-1 | CopA family copper-resistance protein | NO | 0 | YES |
| P131_scaffold01315-2 | hypothetical protein | NO | 0 | NO |
| P131_scaffold00072-3 | ankyrin | NO | 0 | NO |
| P131_scaffold01766-2 | no match | NO | 0 | NO |
| P131_scaffold00629-4 | geranylgeranyl pyrophosphate synthetase | NO | 1 | NO |
| P131_scaffold00595-1 | glycosyl transferase | NO | 0 | NO |
| P131_scaffold01806-1 | no match | NO | 0 | NO |
| P131_scaffold01513-8 | no match | NO | 0 | NO |
| P131_scaffold00436-6 | hypothetical protein | NO | 0 | NO |
| P131_scaffold01369-1 | ATP-binding protein | NO | 0 | NO |
| P131_scaffold01206-1 | hypothetical protein | NO | 0 | NO |
| P131_scaffold00292-4 | no match | NO | 0 | NO |
| P131_scaffold00051-8 | no match | NO | 0 | NO |
| P131_scaffold00032-3 | hypothetical protein | NO | 0 | NO |
| P131_scaffold01420-4 | amino acid transporter | YES | 0 | NO |
| P131_scaffold00817-2 | no match | YES | 0 | NO |
| P131_scaffold01300-1 | SNF2 family DNA/RNA helicase | NO | 0 | NO |
| P131_scaffold01164-13 | Type II restriction enzyme TaqI | YES | 0 | NO |
| P131_scaffold01579-4 | interferon-induced GTP-binding protein Mx1 | NO | 0 | NO |
| P131_scaffold00761-3 | hypothetical protein | NO | 0 | NO |
| P131_scaffold00823-1 | no match | NO | 0 | NO |
| P131_scaffold01787-1 | hypothetical protein | NO | 0 | YES |
| P131_scaffold01157-1 | no match | NO | 0 | YES |
| P131_scaffold00502-2 | no match | NO | 0 | NO |
| P131_scaffold01392-2 | no match | NO | 0 | NO |
| P131_scaffold01219-1 | no match | NO | 0 | NO |
| P131_scaffold00629-3 | no match | NO | 0 | NO |
| P131_scaffold01737-5 | AAA family ATPase | NO | 0 | NO |
| P131_scaffold01269-2 | geranylgeranyl pyrophosphate synthetase | NO | 0 | NO |
| P131_scaffold01783-1 | hypothetical protein | YES | 0 | NO |
| P131_scaffold01074-1 | hypothetical protein | NO | 0 | NO |
| P131_scaffold00006-5 | hypothetical protein | NO | 6 | NO |
| P131_scaffold01513-1 | no match | NO | 0 | NO |
| P131_scaffold01435-2 | no match | NO | 0 | NO |
| P131_scaffold01222-2 | no match | NO | 0 | NO |
| P131_scaffold01821-6 | no match | NO | 0 | NO |
| P131_scaffold00595-2 | hypothetical protein | NO | 0 | NO |
| P131_scaffold00107-4 | no match | NO | 0 | NO |
| P131_scaffold00611-4 | polyvinylalcohol dehydrogenase | YES | 0 | NO |
| P131_scaffold00406-2 | hypothetical protein | YES | 0 | NO |
| P131_scaffold01315-3 | 3-carboxymuconate cyclase | YES | 0 | NO |
| P131_scaffold01697-25 | no match | YES | 0 | NO |
| P131_scaffold01101-3 | trichothecene C-8 hydroxylase | NO | 1 | NO |
| P131_scaffold01405-1 | hypothetical protein | NO | 0 | NO |
| P131_scaffold01512-3 | IBR finger domain protein | NO | 0 | YES |
| P131_scaffold01643-2 | cytochrome P450 monooxygenase | NO | 0 | NO |
| P131_scaffold00587-1 | AVR-Pia | YES | 0 | NO |
| P131_scaffold01245-1 | hypothetical protein | NO | 0 | NO |
| P131_scaffold00409-1 | hypothetical protein | NO | 0 | NO |
| P131_scaffold01577-2 | D-mandelate dehydrogenase | NO | 0 | NO |
| P131_scaffold00582-2 | hypothetical protein | NO | 0 | NO |
| P131_scaffold00581-1 | no match | NO | 0 | NO |
| P131_scaffold01799-1 | hypothetical protein | NO | 0 | NO |
| P131_scaffold00025-1 | no match | NO | 0 | NO |
| P131_scaffold00951-6 | alpha/beta fold family hydrolase | NO | 0 | NO |
| P131_scaffold01579-11 | Peroxisome biogenesis factor 10 isoform 1 | NO | 1 | NO |
| P131_scaffold00506-11 | hypothetical protein | NO | 0 | NO |
| P131_scaffold01574-3 | no match | NO | 0 | NO |
| P131_scaffold00099-2 | hypothetical protein | NO | 0 | NO |
| P131_scaffold00629-2 | MFS monosaccharide transporter | NO | 7 | NO |
| P131_scaffold01420-2 | hypothetical protein | YES | 0 | NO |
| P131_scaffold01174-1 | no match | NO | 0 | NO |
| P131_scaffold01821-3 | no match | NO | 0 | NO |
| P131_scaffold00155-1 | predicted protein | NO | 2 | NO |
| P131_scaffold00616-1 | pfs domain-containing protein | NO | 0 | NO |
| P131_scaffold01164-16 | hypothetical protein | NO | 0 | NO |
| P131_scaffold01341-1 | hypothetical protein | NO | 0 | NO |
| P131_scaffold00978-1 | no match | NO | 0 | NO |
| P131_scaffold01579-13 | Response regulator receiver | NO | 0 | NO |
| P131_scaffold00629-5 | no match | NO | 0 | NO |
| P131_scaffold01514-2 | no match | NO | 0 | NO |
| P131_scaffold01392-1 | no match | NO | 0 | NO |
| P131_scaffold01536-3 | hypothetical protein | NO | 0 | YES |
| P131_scaffold01235-4 | MAP kinase kinase skh1/pek1 | NO | 0 | NO |
| P131_scaffold00995-2 | hypothetical protein | NO | 0 | NO |
| P131_scaffold00575-1 | trypsin | YES | 0 | NO |
| P131_scaffold00099-1 | APSES transcription factor | NO | 0 | NO |
| P131_scaffold01423-1 | no match | YES | 0 | NO |
| P131_scaffold00006-3 | no match | NO | 5 | NO |
| P131_scaffold01821-1 | transcriptional activator | NO | 0 | NO |
| P131_scaffold01677-3 | hypothetical protein | NO | 0 | NO |
| P131_scaffold01164-15 | DNA polymerase LigD | NO | 0 | NO |
| P131_scaffold00279-31 | succinate dehydrogenase flavoprotein subunit | NO | 1 | NO |
| P131_scaffold00041-4 | ankyrin repeat domain | NO | 0 | NO |
| P131_scaffold00107-6 | hypothetical protein | YES | 0 | NO |
| P131_scaffold01409-1 | hypothetical protein | YES | 0 | NO |
| P131_scaffold01369-2 | DegT/DnrJ/EryC1/StrS aminotransferase | NO | 0 | NO |
| P131_scaffold01227-5 | hypothetical protein | YES | 0 | NO |
| P131_scaffold01821-4 | no match | YES | 2 | NO |
| P131_scaffold01579-10 | no match | NO | 0 | NO |
| P131_scaffold01212-1 | kinase domain containing protein | NO | 0 | NO |
| P131_scaffold00910-2 | no match | NO | 0 | NO |
| P131_scaffold01579-5 | no match | NO | 0 | NO |
| P131_scaffold01254-1 | no match | YES | 1 | NO |
| P131_scaffold01667-1 | hypothetical protein | NO | 0 | NO |
| P131_scaffold01821-7 | hypothetical protein | NO | 0 | NO |
| P131_scaffold01428-1 | no match | NO | 0 | NO |
| P131_scaffold00208-1 | no match | NO | 0 | NO |
| P131_scaffold01777-2 | no match | NO | 0 | NO |
| P131_scaffold00611-2 | hypothetical protein | YES | 0 | NO |
| P131_scaffold00978-3 | no match | NO | 0 | NO |
| Y34_scaffold00971-1 | maturase K | NO | 0 | NO |
| Y34_scaffold01045-2 | hypothetical protein | NO | 0 | NO |
| Y34_scaffold00878-2 | no match | NO | 0 | NO |
| Y34_scaffold00944-2 | no match | NO | 0 | NO |
| Y34_scaffold00399-3 | hypothetical protein | NO | 5 | NO |
| Y34_scaffold00820-2 | hypothetical protein | NO | 0 | NO |
| Y34_scaffold00455-1 | no match | NO | 0 | NO |
| Y34_scaffold01175-8 | DNAJ domain containing protein | NO | 0 | NO |
| Y34_scaffold00972-6 | hypothetical protein | NO | 0 | NO |
| Y34_scaffold01175-4 | phosphofructokinase | NO | 0 | NO |
| Y34_scaffold00647-1 | dynamin GTPase | NO | 0 | NO |
| Y34_scaffold01135-1 | no match | NO | 0 | NO |
| Y34_scaffold00123-2 | thymidylate kinase | YES | 0 | NO |
| Y34_scaffold00100-5 | hypothetical protein | NO | 0 | NO |
| Y34_scaffold00166-2 | cytochrome P450 monooxygenase | NO | 0 | NO |
| Y34_scaffold01167-3 | no match | YES | 0 | NO |
| Y34_scaffold00057-9 | trypsin | YES | 0 | NO |
| Y34_scaffold00815-1 | no match | NO | 1 | NO |
| Y34_scaffold01011-1 | no match | NO | 0 | YES |
| Y34_scaffold01179-2 | hypothetical protein | NO | 0 | NO |
| Y34_scaffold00846-7 | hypothetical protein | YES | 0 | YES |
| Y34_scaffold00320-1 | no match | YES | 0 | NO |
| Y34_scaffold00846-10 | no match | NO | 0 | NO |
| Y34_scaffold00104-2 | hypothetical protein | NO | 0 | NO |
| Y34_scaffold00310-2 | hypothetical protein | NO | 0 | NO |
| Y34_scaffold00730-1 | hypothetical protein | NO | 0 | NO |
| Y34_scaffold01172-4 | hypothetical protein | NO | 0 | NO |
| Y34_scaffold01152-7 | no match | NO | 0 | NO |
| Y34_scaffold01160-2 | no match | NO | 0 | NO |
| Y34_scaffold00367-1 | no match | YES | 0 | NO |
| Y34_scaffold00877-3 | no match | NO | 0 | NO |
| Y34_scaffold00804-1 | no match | NO | 0 | NO |
| Y34_scaffold01115-7 | no match | NO | 0 | NO |
| Y34_scaffold00170-4 | phosphatidyl synthase | NO | 0 | NO |
| Y34_scaffold00082-2 | hypothetical protein | YES | 0 | NO |
| Y34_scaffold00993-2 | no match | YES | 0 | NO |
| Y34_scaffold00374-2 | no match | NO | 0 | NO |
| Y34_scaffold00614-3 | hypothetical protein | NO | 0 | NO |
| Y34_scaffold00399-4 | amino acid transporter | YES | 0 | NO |
| Y34_scaffold01172-8 | no match | NO | 0 | NO |
| Y34_scaffold00804-5 | no match | NO | 0 | NO |
| Y34_scaffold00252-8 | no match | NO | 0 | NO |
| Y34_scaffold00079-7 | no match | NO | 0 | NO |
| Y34_scaffold00846-1 | no match | NO | 0 | NO |
| Y34_scaffold01152-2 | CAMK family protein kinase | NO | 0 | NO |
| Y34_scaffold00305-4 | no match | NO | 0 | NO |
| Y34_scaffold00920-4 | hypothetical protein | YES | 0 | NO |
| Y34_scaffold00310-1 | no match | YES | 0 | NO |
| Y34_scaffold00714-2 | phyllocladan-16a-ol synthase | NO | 0 | NO |
| Y34_scaffold01147-4 | no match | NO | 0 | NO |
| Y34_scaffold00990-2 | chitinase | YES | 1 | NO |
| Y34_scaffold00377-2 | no match | NO | 1 | NO |
| Y34_scaffold00082-4 | MFS transporter | NO | 13 | NO |
| Y34_scaffold00862-1 | no match | NO | 2 | NO |
| Y34_scaffold01175-7 | no match | NO | 0 | NO |
| Y34_scaffold00346-7 | no match | NO | 0 | NO |
| Y34_scaffold01172-1 | kinase domain containing protein | NO | 0 | NO |
| Y34_scaffold00972-4 | hypothetical protein | NO | 0 | YES |
| Y34_scaffold00243-1 | hypothetical protein | NO | 0 | NO |
| Y34_scaffold00252-2 | no match | NO | 0 | NO |
| Y34_scaffold00700-1 | no match | NO | 0 | NO |
| Y34_scaffold00870-5 | Ankyrin repeat protein | NO | 1 | NO |
| Y34_scaffold00296-3 | Patatin-like phospholipase | NO | 0 | NO |
| Y34_scaffold00614-2 | hypothetical protein | NO | 2 | NO |
| Y34_scaffold01172-10 | hypothetical protein | NO | 0 | NO |
| Y34_scaffold00876-2 | no match | NO | 0 | YES |
| Y34_scaffold00622-1 | DNA polymerase LigD | NO | 0 | NO |
| Y34_scaffold01019-8 | no match | NO | 0 | NO |
| Y34_scaffold00696-4 | hypothetical protein | YES | 0 | NO |
| Y34_scaffold01184-2 | no match | NO | 0 | NO |
| Y34_scaffold00846-9 | ferric reductase transmembrane component 3 | YES | 2 | NO |
| Y34_scaffold00067-2 | D-mandelate dehydrogenase | NO | 0 | NO |
| Y34_scaffold00025-3 | ankyrin repeat domain | NO | 0 | NO |
| Y34_scaffold00051-8 | hypothetical protein | YES | 0 | NO |
| Y34_scaffold01188-1 | hypothetical protein | NO | 0 | NO |
| Y34_scaffold00002-1 | no match | NO | 0 | NO |
| Y34_scaffold00252-3 | no match | NO | 0 | NO |
| Y34_scaffold01181-1 | hypothetical protein | NO | 0 | NO |
| Y34_scaffold01073-26 | no match | YES | 0 | NO |
| Y34_scaffold01147-5 | no match | NO | 0 | NO |
| Y34_scaffold00860-1 | predicted protein | NO | 0 | NO |
| Y34_scaffold00300-7 | C2H2 transcription factor RfeC | NO | 0 | NO |
| Y34_scaffold01175-11 | no match | NO | 0 | NO |
| Y34_scaffold00450-1 | no match | NO | 0 | NO |
| Y34_scaffold00491-3 | alpha/beta fold family hydrolase | NO | 0 | NO |
| Y34_scaffold00100-2 | hypothetical protein | NO | 0 | NO |
| Y34_scaffold00078-1 | ATP-binding protein | NO | 0 | NO |
| Y34_scaffold00399-2 | hypothetical protein | YES | 0 | NO |
| Y34_scaffold00016-1 | interferon-induced GTP-binding protein Mx1 | NO | 0 | NO |
| Y34_scaffold01050-1 | no match | NO | 0 | NO |
| Y34_scaffold00804-3 | no match | NO | 0 | NO |
| Y34_scaffold00346-6 | no match | NO | 0 | NO |
| Y34_scaffold00857-7 | ankyrin | NO | 0 | NO |
| Y34_scaffold01179-3 | no match | NO | 0 | NO |
| Y34_scaffold01175-5 | no match | NO | 0 | NO |
| Y34_scaffold00846-3 | no match | NO | 0 | NO |
| Y34_scaffold01135-2 | no match | YES | 0 | NO |
| Y34_scaffold00526-1 | no match | NO | 0 | NO |
| Y34_scaffold00643-1 | no match | NO | 0 | NO |
| Y34_scaffold01073-25 | hypothetical protein | YES | 0 | NO |
| Y34_scaffold00622-2 | hypothetical protein | NO | 0 | NO |
| Y34_scaffold01040-3 | no match | YES | 0 | NO |
| Y34_scaffold00730-2 | Type II restriction enzyme TaqI | YES | 0 | NO |
| Y34_scaffold01160-3 | no match | YES | 0 | NO |
| Y34_scaffold00820-5 | no match | NO | 0 | NO |
| Y34_scaffold01191-2 | no match | NO | 0 | NO |
| Y34_scaffold01043-3 | two-component sensor histidine kinase/response regulator hybrid protein | NO | 0 | YES |
| Y34_scaffold00082-1 | no match | YES | 0 | NO |
| Y34_scaffold00418-1 | no match | NO | 0 | NO |
| Y34_scaffold00420-4 | hypothetical protein | NO | 0 | NO |
| Y34_scaffold00078-2 | DegT/DnrJ/EryC1/StrS aminotransferase | NO | 0 | NO |
| Y34_scaffold01050-2 | AVR-Pia | YES | 0 | NO |
| Y34_scaffold00838-5 | hypothetical protein | YES | 4 | NO |
| Y34_scaffold00067-1 | CFEM domain containing protein | NO | 7 | NO |
| Y34_scaffold01014-5 | AAA family ATPase | NO | 0 | NO |
| Y34_scaffold00459-1 | no match | YES | 0 | NO |
| Y34_scaffold00920-1 | no match | YES | 1 | NO |
| Y34_scaffold01175-3 | response regulator receiver | NO | 0 | NO |
| Y34_scaffold00450-3 | no match | NO | 0 | NO |
| Y34_scaffold00453-12 | hypothetical protein | NO | 0 | NO |
| Y34_scaffold01152-3 | no match | NO | 0 | NO |
| Y34_scaffold01020-1 | no match | NO | 0 | NO |
| Y34_scaffold00626-4 | no match | YES | 0 | YES |
| Y34_scaffold00362-1 | no match | NO | 0 | NO |
| Y34_scaffold00643-2 | putative agmatinase | NO | 0 | YES |
| Y34_scaffold01031-11 | hypothetical protein | NO | 0 | NO |
| Y34_scaffold01115-3 | calcium/calmodulin-dependent protein kinase I | NO | 0 | NO |
| Y34_scaffold00714-3 | cytochrome P450 | NO | 1 | NO |
| Y34_scaffold00305-5 | no match | NO | 0 | NO |
| Y34_scaffold00863-2 | no match | NO | 0 | NO |
| Y34_scaffold00818-5 | trichothecene C-8 hydroxylase | NO | 1 | NO |
| Y34_scaffold00442-5 | hypothetical protein | YES | 0 | NO |
| Y34_scaffold00358-1 | no match | NO | 0 | NO |
| Y34_scaffold00696-3 | hypothetical protein | YES | 1 | NO |
| Y34_scaffold01191-1 | no match | NO | 0 | NO |
| Y34_scaffold00296-2 | phospholipase | NO | 0 | YES |
| Y34_scaffold00434-2 | no match | NO | 0 | NO |
| Y34_scaffold01175-2 | ferric-chelate reductase | YES | 3 | NO |
| Y34_scaffold00750-1 | hypothetical protein | NO | 0 | NO |
| Y34_scaffold01167-6 | NAD(P)H-dependent glutamate synthase | NO | 0 | NO |
| Y34_scaffold00820-3 | serine/threonine-protein kinase | NO | 0 | NO |
| Y34_scaffold01031-12 | C6 transcription factor | NO | 1 | NO |
| Y34_scaffold00741-2 | no match | NO | 0 | NO |
| Y34_scaffold00972-5 | no match | NO | 0 | NO |
| Y34_scaffold00972-1 | predicted protein | NO | 0 | YES |
| Y34_scaffold00505-3 | thioredoxin | NO | 0 | NO |
| Y34_scaffold00605-3 | no match | NO | 0 | NO |
| Y34_scaffold01083-3 | no match | NO | 0 | NO |
| Y34_scaffold01179-1 | ferric-chelate reductase | NO | 0 | NO |
| Y34_scaffold00166-3 | hypothetical protein | NO | 0 | NO |
| Y34_scaffold01172-2 | transcriptional activator | NO | 0 | NO |
| Y34_scaffold00242-3 | no match | NO | 0 | NO |
| Y34_scaffold00700-3 | no match | NO | 0 | NO |
| Y34_scaffold00487-99 | arylsulfatase B precursor | NO | 0 | NO |
| Y34_scaffold00513-2 | indole-3-glycerol-phosphate synthase | NO | 1 | NO |
| Y34_scaffold01011-2 | no match | NO | 0 | NO |
| Y34_scaffold00067-3 | efflux pump antibiotic resistance protein | YES | 5 | NO |
| Y34_scaffold00551-1 | hypothetical protein | NO | 0 | NO |
| Y34_scaffold01171-6 | no match | NO | 0 | NO |
| Y34_scaffold00846-6 | no match | NO | 0 | NO |
| Y34_scaffold00083-2 | no match | NO | 0 | YES |
| Y34_scaffold00816-1 | serine/threonine kinase family protein | NO | 0 | NO |
| Y34_scaffold00838-3 | geranylgeranyl pyrophosphate synthetase | NO | 1 | NO |
| Y34_scaffold00104-1 | glycosyl transferase | NO | 0 | NO |
| Y34_scaffold01040-1 | no match | NO | 0 | NO |
| Y34_scaffold00700-4 | no match | NO | 0 | NO |
| Y34_scaffold00842-1 | no match | NO | 1 | NO |
| Y34_scaffold00491-2 | neutral protease | YES | 0 | NO |
| Y34_scaffold01152-1 | hypothetical protein | NO | 0 | NO |
| Y34_scaffold01115-6 | F-box protein | NO | 0 | NO |
| Y34_scaffold00281-1 | no match | NO | 0 | NO |
| Y34_scaffold00868-2 | hypothetical protein | NO | 0 | NO |
| Y34_scaffold01172-9 | MAP kinase kinase skh1/pek1 | NO | 0 | NO |
| Y34_scaffold00166-4 | no match | NO | 0 | NO |
| Y34_scaffold00692-40 | succinate dehydrogenase flavoprotein subunit | NO | 1 | NO |
| Y34_scaffold01175-1 | no match | NO | 0 | NO |
| Y34_scaffold00082-3 | hypothetical protein | NO | 0 | NO |
| Y34_scaffold00016-2 | cadherin associated protein | NO | 0 | NO |
| Y34_scaffold00755-2 | hypothetical protein | YES | 0 | NO |
| Y34_scaffold00499-2 | no match | NO | 0 | NO |
| Y34_scaffold00804-4 | no match | NO | 0 | NO |
| Y34_scaffold00346-3 | no match | NO | 0 | NO |
| Y34_scaffold00781-1 | LysM domain-containing protein | YES | 0 | NO |
| Y34_scaffold00498-25 | no match | YES | 0 | NO |
| Y34_scaffold01039-8 | no match | NO | 0 | NO |
| Y34_scaffold00505-1 | no match | YES | 0 | NO |
| Y34_scaffold00920-3 | no match | NO | 0 | NO |
| Y34_scaffold00311-2 | no match | NO | 0 | NO |
| Y34_scaffold01023-1 | no match | NO | 0 | NO |
| Y34_scaffold00846-5 | calcium/calmodulin-dependent protein kinase I | NO | 0 | NO |
| Y34_scaffold00558-1 | hypothetical protein | NO | 0 | NO |
| Y34_scaffold00300-8 | hypothetical protein | NO | 0 | NO |
| Y34_scaffold00714-1 | hypothetical protein | NO | 0 | NO |
| Y34_scaffold00969-33 | no match | NO | 0 | NO |
| Y34_scaffold01043-2 | addiction module toxin | NO | 0 | NO |
| Y34_scaffold00614-1 | hypothetical protein | NO | 0 | NO |
| Y34_scaffold00920-2 | no match | YES | 0 | NO |
| Y34_scaffold00859-2 | hypothetical protein | NO | 0 | NO |
| Y34_scaffold00863-3 | no match | NO | 0 | NO |
| Y34_scaffold00442-4 | fungal cellulose binding domain containing protein | YES | 0 | NO |
| Y34_scaffold00696-2 | polyvinylalcohol dehydrogenase | YES | 0 | NO |
| Y34_scaffold01037-1 | no match | NO | 0 | YES |
| Y34_scaffold00838-4 | no match | NO | 0 | NO |
| Y34_scaffold01115-4 | no match | NO | 0 | NO |
| Y34_scaffold00804-6 | hypothetical protein | NO | 0 | NO |
| Y34_scaffold01190-2 | no match | NO | 0 | NO |
| Y34_scaffold00869-1 | hypothetical protein | NO | 0 | NO |
| Y34_scaffold00846-8 | hypothetical protein | NO | 0 | NO |
| Y34_scaffold00252-4 | no match | NO | 0 | YES |
| Y34_scaffold00845-1 | no match | NO | 0 | NO |
| Y34_scaffold00845-3 | no match | NO | 0 | NO |
| Y34_scaffold00268-2 | BTB/POZ domain protein | NO | 0 | NO |
| Y34_scaffold00238-1 | no match | NO | 1 | NO |
| Y34_scaffold01172-5 | predicted protein | NO | 0 | NO |
| Y34_scaffold00311-3 | no match | NO | 0 | NO |
| Y34_scaffold00824-7 | no match | NO | 0 | NO |
| Y34_scaffold00692-29 | hypothetical protein | NO | 0 | NO |
| Y34_scaffold00173-4 | no match | YES | 1 | NO |
| Y34_scaffold00239-1 | hypothetical protein | NO | 0 | YES |
| Y34_scaffold00642-5 | hypothetical protein | YES | 0 | NO |

Secreted, secreted proteins; TM, transmembrane domains; NLS, nuclear localization signals.
