# Supplementary material for: Comparative Analysis of the Genomes of Two Field Isolates of the Rice Blast Fungus Magnaporthe oryzae
Source: PLoS Genet. 2012 Aug 2;8(8):e1002869. doi: 10.1371/journal.pgen.1002869 (PMC3410873; doi:10.1371/journal.pgen.1002869)
Supplement: Table S6 — Genes predicted within duplicated genomic fragments of P131, Y34, and 70-15. (DOC) [file pgen.1002869.s014.doc]

**Table S6** Genes predicted within duplicated genomic fragments of P131, Y34, and 70-15.

| **Gene** | **Annotation** | **Secreted** | **TM** |
| --- | --- | --- | --- |
| P131_scaffold00060-1 P131_scaffold01816-1 | LPS glycosyltransferase | YES | 1 |
| P131_scaffold00592-1 P131_scaffold01676-1 | BCS1-like ATPase | NO | 0 |
| P131_scaffold01174-1 P131_scaffold01514-1 | no match | NO | 0 |
| P131_scaffold01409-1 P131_scaffold01783-1 | hypothetical protein | YES | 0 |
| P131_scaffold01491-1 P131_scaffold01781-1 | no match | NO | 0 |
| P131_scaffold01677-6 P131_scaffold01424-1 | hypothetical protein | NO | 0 |
| P131_scaffold01742-1 P131_scaffold01531-1 | hypothetical protein | NO | 0 |
| P131_scaffold01745-1 P131_scaffold01779-1 | no match | NO | 0 |
| Y34_scaffold00001-1 Y34_scaffold01145-2 | kinesin light chain 3 | NO | 0 |
| Y34_scaffold00300-3 Y34_scaffold00969-52 | LPS glycosyltransferase | YES | 1 |
| Y34_scaffold00316-3 Y34_scaffold00624-79 | hypothetical protein | NO | 0 |
| Y34_scaffold00676-3 Y34_scaffold00079-5 | no match | NO | 0 |
| Y34_scaffold00804-2 Y34_scaffold00863-1 | glycosyltransferase alpha 1 | NO | 0 |
| Y34_scaffold00804-7 Y34_scaffold01101-2 | no match | NO | 0 |
| Y34_scaffold00846-6 Y34_scaffold01115-4 | no match | NO | 0 |
| Y34_scaffold00995-1 Y34_scaffold01180-12 | hypothetical protein | NO | 0 |
| Y34_scaffold01033-2 Y34_scaffold00870-7 | hypothetical protein | NO | 0 |
| Y34_scaffold01174-1 Y34_scaffold01164-1 | hypothetical protein | NO | 0 |
| supercontig_6.10-152 supercontig_6.10-282 | no match | NO | 0 |
| supercontig_6.11-102 supercontig_6.12-834 | no match | NO | 0 |
| supercontig_6.11-45 supercontig_6.4-204 supercontig_6.8-210 | hypothetical protein | YES | 0 |
| supercontig_6.11-71 supercontig_6.9-149 | MFS transporter | NO | 13 |
| supercontig_6.12-57 supercontig_6.28-158 | PWL2 | YES | 1 |
| supercontig_6.12-59 supercontig_6.28-160 | hypothetical protein | NO | 0 |
| supercontig_6.12-60 supercontig_6.12-645 supercontig_6.28-161 | no match | NO | 0 |
| supercontig_6.12-62 supercontig_6.28-163 | MFS transporter | NO | 13 |
| supercontig_6.12-649 supercontig_6.28-162 | hypothetical protein | NO | 0 |
| supercontig_6.12-833 supercontig_6.12-61 | hypothetical protein | NO | 0 |
| supercontig_6.12-919 supercontig_6.16-262 supercontig_6.25-79 | hypothetical protein | YES | 0 |
| supercontig_6.13-1180 supercontig_6.13-8 | hypothetical protein | NO | 0 |
| supercontig_6.13-1181 supercontig_6.13-7 | class II Aldolase and Adducin N-terminal domain protein | NO | 0 |
| supercontig_6.13-1186 supercontig_6.13-4 | gluconolactonase precursor | NO | 0 |
| supercontig_6.13-1187 supercontig_6.13-3 | sugar transporter STL1 | YES | 12 |
| supercontig_6.13-287 supercontig_6.25-254 | no match | NO | 0 |
| supercontig_6.13-338 supercontig_6.13-340 | hypothetical protein | NO | 0 |
| supercontig_6.14-111 supercontig_6.4-196 supercontig_6.6-16 | no match | NO | 0 |
| supercontig_6.14-98 supercontig_6.6-23 | hypothetical protein | YES | 0 |
| supercontig_6.15-171 supercontig_6.15-174 | hypothetical protein | NO | 0 |
| supercontig_6.15-177 supercontig_6.17-19 | hypothetical protein | NO | 9 |
| supercontig_6.15-179 supercontig_6.17-30 | hypothetical protein | YES | 1 |
| supercontig_6.15-180 supercontig_6.17-29 | hypothetical protein | NO | 0 |
| supercontig_6.15-181 supercontig_6.17-28 | no match | NO | 0 |
| supercontig_6.15-182 supercontig_6.17-27 | hypothetical protein | NO | 0 |
| supercontig_6.15-183 supercontig_6.17-26 | hypothetical protein | NO | 0 |
| supercontig_6.15-184 supercontig_6.17-25 | hypothetical protein | NO | 0 |
| supercontig_6.15-49 supercontig_6.7-103 | hypothetical protein | YES | 0 |
| supercontig_6.15-51 supercontig_6.4-198 supercontig_6.7-105 | hypothetical protein | YES | 0 |
| supercontig_6.15-522 supercontig_6.17-2 | hypothetical protein | YES | 0 |
| supercontig_6.15-523 supercontig_6.17-3 | hypothetical protein | YES | 0 |
| supercontig_6.15-524 supercontig_6.17-4 | carboxypeptidase | YES | 0 |
| supercontig_6.15-526 supercontig_6.17-6 | hypothetical protein | NO | 0 |
| supercontig_6.15-527 supercontig_6.17-7 | no match | NO | 0 |
| supercontig_6.15-611 supercontig_6.17-20 | hypothetical protein | NO | 0 |
| supercontig_6.16-273 supercontig_6.7-101 | hypothetical protein | NO | 2 |
| supercontig_6.17-10 supercontig_6.18-953 | cutinase | YES | 0 |
| supercontig_6.17-51 supercontig_6.7-89 | hypothetical protein | NO | 0 |
| supercontig_6.17-52 supercontig_6.7-90 | phosphotransferase family protein | NO | 0 |
| supercontig_6.17-54 supercontig_6.7-92 | hypothetical protein | NO | 0 |
| supercontig_6.17-56 supercontig_6.7-94 | hypothetical protein | NO | 3 |
| supercontig_6.17-58 supercontig_6.7-96 | elongation factor 2 kinase | NO | 0 |
| supercontig_6.17-59 supercontig_6.7-97 | no match | NO | 2 |
| supercontig_6.17-60 supercontig_6.7-98 | hypothetical protein | NO | 0 |
| supercontig_6.17-61 supercontig_6.7-99 | hypothetical protein | NO | 0 |
| supercontig_6.17-62 supercontig_6.7-88 | hypothetical protein | NO | 0 |
| supercontig_6.17-9 supercontig_6.18-954 | hypothetical protein | YES | 0 |
| supercontig_6.18-1317 supercontig_6.2-15 supercontig_6.21-1410 | hypothetical protein | NO | 0 |
| supercontig_6.18-1378 supercontig_6.21-1302 | no match | YES | 0 |
| supercontig_6.18-1379 supercontig_6.21-1301 | no match | NO | 0 |
| supercontig_6.20-4 supercontig_6.23-755 | hypothetical protein | NO | 0 |
| supercontig_6.20-5 supercontig_6.23-756 | hypothetical protein | NO | 0 |
| supercontig_6.21-1321 supercontig_6.9-103 | hypothetical protein | NO | 0 |
| supercontig_6.21-1334 supercontig_6.21-1399 | no match | YES | 0 |
| supercontig_6.21-1413 supercontig_6.29-917 | hypothetical protein | YES | 0 |
| supercontig_6.25-72 supercontig_6.7-77 | no match | NO | 0 |
| supercontig_6.28-130 supercontig_6.28-165 | hypothetical protein | YES | 0 |
| supercontig_6.28-134 supercontig_6.28-169 | hypothetical protein | YES | 0 |
| supercontig_6.28-137 supercontig_6.28-173 | predicted protein | YES | 1 |
| supercontig_6.28-138 supercontig_6.28-175 | hypothetical protein | NO | 0 |
| supercontig_6.28-139 supercontig_6.28-176 | hypothetical protein | NO | 0 |
| supercontig_6.28-140 supercontig_6.28-177 | hypothetical protein | NO | 0 |
| supercontig_6.28-141 supercontig_6.28-178 | no match | NO | 1 |
| supercontig_6.28-147 supercontig_6.8-213 | hypothetical protein | NO | 0 |
| supercontig_6.28-157 supercontig_6.4-193 supercontig_6.8-62 | sterigmatocystin 8-O-methyltransferase | NO | 0 |
| supercontig_6.29-890 supercontig_6.29-895 | no match | NO | 0 |
| supercontig_6.3-2 supercontig_6.31-9 | no match | NO | 0 |
| supercontig_6.4-188 supercontig_6.6-12 | carbonic anhydrase 2 | NO | 0 |
| supercontig_6.4-195 supercontig_6.9-154 | multidrug resistance protein 1 | NO | 12 |
| supercontig_6.4-199 supercontig_6.15-52 supercontig_6.7-106 | hypothetical protein | NO | 0 |
| supercontig_6.7-100 supercontig_6.16-274 | hypothetical protein | NO | 0 |
| supercontig_6.8-219 supercontig_6.8-66 supercontig_6.8-72 | hypothetical protein | NO | 0 |
| supercontig_6.8-65 supercontig_6.8-73 | hypothetical protein | NO | 0 |

Secreted, secreted proteins; TM, transmembrane domains.
