# Supplementary material for: Comparative Analysis of the Genomes of Two Field Isolates of the Rice Blast Fungus Magnaporthe oryzae
Source: PLoS Genet. 2012 Aug 2;8(8):e1002869. doi: 10.1371/journal.pgen.1002869 (PMC3410873; doi:10.1371/journal.pgen.1002869)
Supplement: Table S7 — Gene families with the same number of members in all three isolates. (DOC) [file pgen.1002869.s015.doc]

**Table S7 Gene families with the same number of members in all three isolates.**

| **ORTHOMCL** | **Gene** | **Annotation** | **Secreted** | **TM** | **Protein length** |
| --- | --- | --- | --- | --- | --- |
| ORTHOMCL2 |  |  |  |  |  |
|  | supercontig_6.12-147 | beta-glucosidase | NO | 1 | 827 |
|  | supercontig_6.25-26 | glycosyl hydrolase | YES | 0 | 814 |
|  | supercontig_6.26-118 | glycosyl hydrolase | YES | 0 | 765 |
|  | supercontig_6.4-173 | beta-glucosidase | YES | 0 | 775 |
|  | Y34_scaffold00448-75 | beta-glucosidase | NO | 0 | 707 |
|  | Y34_scaffold00540-29 | beta-glucosidase | NO | 1 | 789 |
|  | Y34_scaffold00706-11 | glycosyl hydrolase | YES | 0 | 810 |
|  | Y34_scaffold00887-6 | beta-glucosidase | YES | 0 | 969 |
|  | P131_scaffold00145-6 | glycosyl hydrolase | YES | 0 | 810 |
|  | P131_scaffold00153-5 | beta-glucosidase | NO | 1 | 832 |
|  | P131_scaffold00509-6 | beta-glucosidase | YES | 0 | 775 |
|  | P131_scaffold01276-2 | glycosyl hydrolase | YES | 0 | 765 |
| ORTHOMCL10 |  |  |  |  |  |
|  | supercontig_6.18-297 | predicted protein | NO | 0 | 651 |
|  | supercontig_6.18-654 | glucose-regulated protein precursor | YES | 1 | 664 |
|  | supercontig_6.21-568 | hypothetical protein | NO | 0 | 614 |
|  | Y34_scaffold00669-1 | hypothetical protein | NO | 0 | 614 |
|  | Y34_scaffold00684-20 | predicted protein | NO | 0 | 651 |
|  | Y34_scaffold00712-9 | glucose-regulated protein precursor | YES | 1 | 664 |
|  | P131_scaffold00199-9 | predicted protein | NO | 0 | 651 |
|  | P131_scaffold00546-9 | glucose-regulated protein precursor | YES | 1 | 664 |
|  | P131_scaffold01213-2 | hypothetical protein | NO | 0 | 614 |
| ORTHOMCL11 |  |  |  |  |  |
|  | supercontig_6.12-10 | subtilisin-like proprotein convertase P domain | YES | 0 | 562 |
|  | supercontig_6.22-124 | subtilisin-like proprotein convertase P domain | YES | 0 | 557 |
|  | supercontig_6.28-106 | subtilisin-like proprotein convertase P domain | NO | 0 | 559 |
|  | Y34_scaffold00050-9 | subtilisin-like proprotein convertase P domain | NO | 0 | 559 |
|  | Y34_scaffold00069-2 | subtilisin-like proprotein convertase P domain | YES | 0 | 562 |
|  | Y34_scaffold00511-35 | subtilisin-like proprotein convertase P domain | YES | 0 | 557 |
|  | P131_scaffold00197-6 | subtilisin-like proprotein convertase P domain | YES | 0 | 562 |
|  | P131_scaffold00484-6 | subtilisin-like proprotein convertase P domain | YES | 0 | 557 |
|  | P131_scaffold01126-5 | subtilisin-like proprotein convertase P domain | NO | 0 | 559 |
| ORTHOMCL12 |  |  |  |  |  |
|  | supercontig_6.13-1014 | alpha-mannosidase | NO | 14 | 1923 |
|  | supercontig_6.16-29 | alpha-mannosidase | NO | 0 | 1073 |
|  | supercontig_6.18-1145 | sodium/chloride dependent neurotransmitter transporter | NO | 15 | 705 |
|  | Y34_scaffold00216-11 | sodium/chloride dependent neurotransmitter transporter | NO | 15 | 705 |
|  | Y34_scaffold00765-97 | alpha-mannosidase | NO | 14 | 1923 |
|  | Y34_scaffold01005-99 | alpha-mannosidase | NO | 0 | 1073 |
|  | P131_scaffold00166-11 | sodium/chloride dependent neurotransmitter transporter | NO | 15 | 705 |
|  | P131_scaffold01159-9 | alpha-mannosidase | NO | 0 | 1073 |
|  | P131_scaffold01311-54 | alpha-mannosidase | NO | 14 | 1923 |
| ORTHOMCL6 |  |  |  |  |  |
|  | supercontig_6.13-169 | hypothetical protein | NO | 13 | 1594 |
|  | supercontig_6.20-114 | ABC multidrug transporter | NO | 11 | 1469 |
|  | supercontig_6.28-199 | ABC multidrug transporter | NO | 10 | 1463 |
|  | Y34_scaffold00505-5 | ABC multidrug transporter | NO | 7 | 1375 |
|  | Y34_scaffold00733-1 | ABC multidrug transporter | NO | 10 | 1463 |
|  | Y34_scaffold00775-9 | hypothetical protein | NO | 13 | 1594 |
|  | P131_scaffold01271-5 | hypothetical protein | NO | 13 | 1594 |
|  | P131_scaffold01298-1 | ABC multidrug transporter | NO | 7 | 1366 |
|  | P131_scaffold01364-5 | multidrug resistance protein CDR1 | NO | 10 | 1547 |
| ORTHOMCL7 |  |  |  |  |  |
|  | supercontig_6.21-1034 | alpha-1 | YES | 0 | 827 |
|  | supercontig_6.21-547 | alpha-1 | NO | 0 | 890 |
|  | supercontig_6.29-863 | alpha-1 | YES | 0 | 787 |
|  | Y34_scaffold00555-11 | alpha-1 | YES | 0 | 644 |
|  | Y34_scaffold00608-75 | alpha-1 | YES | 0 | 827 |
|  | Y34_scaffold00669-22 | alpha-1 | NO | 0 | 890 |
|  | P131_scaffold00431-10 | alpha-1 | YES | 0 | 644 |
|  | P131_scaffold01213-23 | alpha-1 | NO | 0 | 890 |
|  | P131_scaffold01455-4 | alpha-1 | YES | 0 | 827 |
| ORTHOMCL9 |  |  |  |  |  |
|  | supercontig_6.15-86 | bifunctional P-450:NADPH-P450 reductase | NO | 0 | 1116 |
|  | supercontig_6.25-39 | bifunctional P-450:NADPH-P450 reductase | NO | 0 | 1120 |
|  | supercontig_6.25-7 | bifunctional P-450:NADPH-P450 reductase | NO | 0 | 1083 |
|  | Y34_scaffold00015-4 | bifunctional P-450:NADPH-P450 reductase | NO | 0 | 1083 |
|  | Y34_scaffold00141-4 | bifunctional P-450:NADPH-P450 reductase | NO | 0 | 1116 |
|  | Y34_scaffold00706-24 | bifunctional P-450:NADPH-P450 reductase | NO | 0 | 1120 |
|  | P131_scaffold00280-8 | bifunctional P-450:NADPH-P450 reductase | NO | 0 | 1120 |
|  | P131_scaffold00524-2 | bifunctional P-450:NADPH-P450 reductase | NO | 0 | 1116 |
|  | P131_scaffold01548-6 | bifunctional P-450:NADPH-P450 reductase | NO | 0 | 1083 |
| ORTHOMCL19 |  |  |  |  |  |
|  | supercontig_6.15-488 | dynamin family protein | NO | 0 | 742 |
|  | supercontig_6.7-79 | dynamin family protein | NO | 0 | 365 |
|  | Y34_scaffold00498-4 | dynamin family protein | NO | 0 | 742 |
|  | Y34_scaffold01070-1 | dynamin family protein | NO | 0 | 719 |
|  | P131_scaffold01697-4 | dynamin family protein | NO | 0 | 742 |
|  | P131_scaffold01777-1 | dynamin family protein | NO | 0 | 737 |
| ORTHOMCL20 |  |  |  |  |  |
|  | supercontig_6.23-879 | laccase | YES | 0 | 597 |
|  | supercontig_6.24-504 | laccase | YES | 0 | 572 |
|  | Y34_scaffold00117-2 | laccase | YES | 0 | 597 |
|  | Y34_scaffold00653-2 | laccase | YES | 0 | 572 |
|  | P131_scaffold01660-2 | laccase | YES | 0 | 597 |
|  | P131_scaffold01755-1 | laccase | YES | 0 | 572 |
| ORTHOMCL21 |  |  |  |  |  |
|  | supercontig_6.12-602 | cytochrome P450 | NO | 1 | 538 |
|  | supercontig_6.18-924 | cytochrome P450 | NO | 1 | 543 |
|  | Y34_scaffold00779-3 | cytochrome P450 | NO | 1 | 543 |
|  | Y34_scaffold01069-5 | cytochrome P450 | NO | 1 | 538 |
|  | P131_scaffold01576-1 | cytochrome P450 | NO | 1 | 538 |
|  | P131_scaffold01668-6 | cytochrome P450 | NO | 1 | 543 |
| ORTHOMCL22 |  |  |  |  |  |
|  | supercontig_6.10-177 | related to aconitate hydratase precursor | NO | 0 | 801 |
|  | supercontig_6.26-133 | related to aconitate hydratase precursor | NO | 0 | 784 |
|  | Y34_scaffold00370-22 | related to aconitate hydratase precursor | NO | 0 | 801 |
|  | Y34_scaffold00668-11 | aconitate hydratase-like protein | NO | 0 | 784 |
|  | P131_scaffold01276-17 | aconitate hydratase-like protein | NO | 0 | 784 |
|  | P131_scaffold01302-18 | related to aconitate hydratase precursor | NO | 0 | 801 |
| ORTHOMCL23 |  |  |  |  |  |
|  | supercontig_6.12-788 | oligopeptide transporter | NO | 9 | 781 |
|  | supercontig_6.21-1025 | oligopeptide transporter | NO | 11 | 774 |
|  | Y34_scaffold00547-3 | oligopeptide transporter | NO | 9 | 799 |
|  | Y34_scaffold00608-66 | oligopeptide transporter | NO | 11 | 774 |
|  | P131_scaffold01258-12 | oligopeptide transporter | NO | 9 | 781 |
|  | P131_scaffold01557-4 | oligopeptide transporter | YES | 10 | 647 |
| ORTHOMCL24 |  |  |  |  |  |
|  | supercontig_6.20-274 | pre-mRNA-splicing factor ATP-dependent RNA helicase PRP16 | NO | 0 | 1016 |
|  | supercontig_6.25-240 | ATP-dependent RNA helicase DHX8 | NO | 0 | 1207 |
|  | Y34_scaffold00552-34 | pre-mRNA-splicing factor ATP-dependent RNA helicase PRP16 | NO | 0 | 1016 |
|  | Y34_scaffold01073-13 | ATP-dependent RNA helicase DHX8 | NO | 0 | 1207 |
|  | P131_scaffold01198-34 | pre-mRNA-splicing factor ATP-dependent RNA helicase PRP16 | NO | 0 | 1016 |
|  | P131_scaffold01517-13 | ATP-dependent RNA helicase DHX8 | NO | 0 | 1207 |
| ORTHOMCL26 |  |  |  |  |  |
|  | supercontig_6.12-820 | cyclohexanone 1 | NO | 0 | 647 |
|  | supercontig_6.21-395 | cyclohexanone 1 | NO | 0 | 641 |
|  | Y34_scaffold00251-22 | cyclohexanone 1 | NO | 0 | 647 |
|  | Y34_scaffold00640-3 | cyclohexanone 1 | NO | 0 | 641 |
|  | P131_scaffold01185-19 | cyclohexanone 1 | NO | 0 | 647 |
|  | P131_scaffold01638-3 | cyclohexanone 1 | NO | 0 | 641 |
| ORTHOMCL27 |  |  |  |  |  |
|  | supercontig_6.10-178 | beta-glucosidase | NO | 0 | 876 |
|  | supercontig_6.20-430 | avenacinase | YES | 0 | 782 |
|  | Y34_scaffold00370-23 | beta-glucosidase | NO | 0 | 911 |
|  | Y34_scaffold00719-15 | avenacinase | YES | 0 | 782 |
|  | P131_scaffold01139-6 | avenacinase | YES | 0 | 782 |
|  | P131_scaffold01302-17 | beta-glucosidase | NO | 0 | 1204 |
| ORTHOMCL28 |  |  |  |  |  |
|  | supercontig_6.23-753 | urea active transporter | NO | 15 | 687 |
|  | supercontig_6.27-94 | urea active transporter | NO | 13 | 658 |
|  | Y34_scaffold00420-5 | urea active transporter | NO | 15 | 687 |
|  | Y34_scaffold00744-6 | urea active transporter | NO | 13 | 658 |
|  | P131_scaffold01020-5 | urea active transporter | NO | 15 | 687 |
|  | P131_scaffold01166-6 | urea active transporter | NO | 13 | 658 |
| ORTHOMCL29 |  |  |  |  |  |
|  | supercontig_6.18-1347 | vitamin H transporter | NO | 10 | 537 |
|  | supercontig_6.8-78 | vitamin H transporter | NO | 11 | 552 |
|  | Y34_scaffold00609-1 | vitamin H transporter | NO | 11 | 552 |
|  | Y34_scaffold00945-7 | vitamin H transporter | NO | 10 | 514 |
|  | P131_scaffold00990-1 | vitamin H transporter | NO | 9 | 428 |
|  | P131_scaffold01586-1 | vitamin H transporter | NO | 11 | 552 |
| ORTHOMCL30 |  |  |  |  |  |
|  | supercontig_6.13-423 | histone core | NO | 0 | 103 |
|  | supercontig_6.21-2 | histone core | NO | 0 | 103 |
|  | Y34_scaffold00359-2 | histone core | NO | 0 | 103 |
|  | Y34_scaffold00559-40 | histone core | NO | 0 | 103 |
|  | P131_scaffold00746-2 | histone core | NO | 0 | 103 |
|  | P131_scaffold01171-10 | histone core | NO | 0 | 103 |
| ORTHOMCL31 |  |  |  |  |  |
|  | supercontig_6.12-242 | sulfate permease II | NO | 10 | 878 |
|  | supercontig_6.16-161 | sulfate permease | NO | 11 | 844 |
|  | Y34_scaffold00255-39 | sulfate permease II | NO | 10 | 934 |
|  | Y34_scaffold00500-34 | sulfate permease | NO | 11 | 844 |
|  | P131_scaffold00606-10 | sulfate permease II | NO | 11 | 802 |
|  | P131_scaffold01211-10 | sulfate permease | NO | 11 | 844 |
| ORTHOMCL32 |  |  |  |  |  |
|  | supercontig_6.24-368 | cytochrome P450 52A12 | YES | 0 | 513 |
|  | supercontig_6.27-605 | cytochrome P450 alkane hydroxylase | YES | 1 | 509 |
|  | Y34_scaffold00478-3 | cytochrome P450 52A12 | YES | 0 | 513 |
|  | Y34_scaffold00501-3 | cytochrome P450 alkane hydroxylase | YES | 1 | 509 |
|  | P131_scaffold00556-2 | cytochrome P450 alkane hydroxylase | YES | 1 | 509 |
|  | P131_scaffold01327-9 | cytochrome P450 52A12 | YES | 0 | 513 |
| ORTHOMCL33 |  |  |  |  |  |
|  | supercontig_6.12-599 | trichodiene oxygenase | NO | 0 | 518 |
|  | supercontig_6.13-773 | trichodiene oxygenase | YES | 0 | 521 |
|  | Y34_scaffold00085-20 | trichodiene oxygenase | NO | 0 | 518 |
|  | Y34_scaffold00182-25 | trichodiene oxygenase | YES | 0 | 521 |
|  | P131_scaffold00539-27 | trichodiene oxygenase | YES | 0 | 521 |
|  | P131_scaffold01331-61 | trichodiene oxygenase | NO | 0 | 469 |
| ORTHOMCL34 |  |  |  |  |  |
|  | supercontig_6.25-266 | beta-glucosidase precursor | NO | 0 | 848 |
|  | supercontig_6.29-99 | glycoside hydrolase family 3 | NO | 0 | 835 |
|  | Y34_scaffold00094-38 | glycoside hydrolase family 3 | NO | 0 | 835 |
|  | Y34_scaffold01031-15 | beta-glucosidase precursor | NO | 0 | 849 |
|  | P131_scaffold00506-15 | beta-glucosidase precursor | NO | 0 | 849 |
|  | P131_scaffold01076-6 | glycoside hydrolase family 3 | NO | 0 | 835 |
| ORTHOMCL35 |  |  |  |  |  |
|  | supercontig_6.13-1105 | glycosyl hydrolase | YES | 0 | 796 |
|  | supercontig_6.29-370 | glycosyl hydrolase | YES | 1 | 892 |
|  | Y34_scaffold00726-73 | glycosyl hydrolase | YES | 0 | 796 |
|  | Y34_scaffold00773-6 | glycosyl hydrolase | YES | 1 | 892 |
|  | P131_scaffold00459-57 | glycosyl hydrolase | YES | 0 | 796 |
|  | P131_scaffold00922-9 | glycosyl hydrolase | YES | 1 | 892 |
| ORTHOMCL36 |  |  |  |  |  |
|  | supercontig_6.16-239 | bilirubin oxidase | YES | 0 | 629 |
|  | supercontig_6.20-272 | polyphenol oxidase | YES | 0 | 627 |
|  | Y34_scaffold00214-11 | bilirubin oxidase | YES | 0 | 629 |
|  | Y34_scaffold00552-36 | polyphenol oxidase | YES | 0 | 589 |
|  | P131_scaffold00435-11 | bilirubin oxidase | YES | 0 | 629 |
|  | P131_scaffold01198-32 | polyphenol oxidase | YES | 0 | 627 |
| ORTHOMCL37 |  |  |  |  |  |
|  | supercontig_6.27-698 | glycosyltransferase family 2 | NO | 4 | 483 |
|  | Y34_scaffold00808-5 | glycosyltransferase family 2 | YES | 3 | 694 |
|  | Y34_scaffold00846-2 | glycosyltransferase family 2 | NO | 3 | 415 |
|  | Y34_scaffold01171-2 | glycosyltransferase family 2 | NO | 4 | 605 |
|  | P131_scaffold00418-1 | glycosyltransferase family 2 | NO | 4 | 483 |
|  | P131_scaffold01513-5 | glycosyltransferase family 2 | NO | 3 | 411 |
| ORTHOMCL38 |  |  |  |  |  |
|  | supercontig_6.25-192 | sugar transporter | NO | 11 | 1085 |
|  | supercontig_6.7-27 | quinate permease | NO | 10 | 1212 |
|  | Y34_scaffold00312-9 | sugar transporter | NO | 11 | 1085 |
|  | Y34_scaffold00685-7 | quinate permease | NO | 10 | 1212 |
|  | P131_scaffold00367-1 | quinate permease | NO | 10 | 1212 |
|  | P131_scaffold00515-9 | sugar transporter | NO | 11 | 1081 |
| ORTHOMCL39 |  |  |  |  |  |
|  | supercontig_6.20-269 | geranylgeranyl pyrophosphate synthase | NO | 0 | 728 |
|  | supercontig_6.26-27 | fusicoccadiene synthase | NO | 0 | 711 |
|  | Y34_scaffold00502-24 | fusicoccadiene synthase | NO | 0 | 711 |
|  | Y34_scaffold00552-39 | polyprenyl synthetase | NO | 0 | 643 |
|  | P131_scaffold00357-5 | fusicoccadiene synthase | NO | 0 | 711 |
|  | P131_scaffold01198-29 | geranylgeranyl pyrophosphate synthase | NO | 0 | 728 |
| ORTHOMCL41 |  |  |  |  |  |
|  | supercontig_6.18-1221 | related to sialidase | NO | 0 | 575 |
|  | supercontig_6.27-50 | related to sialidase | NO | 0 | 581 |
|  | Y34_scaffold00716-3 | related to sialidase | NO | 0 | 575 |
|  | Y34_scaffold00979-39 | related to sialidase | NO | 0 | 581 |
|  | P131_scaffold00290-1 | related to sialidase | NO | 0 | 575 |
|  | P131_scaffold01075-6 | related to sialidase | NO | 0 | 581 |
| ORTHOMCL42 |  |  |  |  |  |
|  | supercontig_6.18-379 | glutamine synthetase | NO | 0 | 347 |
|  | supercontig_6.25-40 | glutamine synthetase | NO | 0 | 358 |
|  | Y34_scaffold00140-52 | glutamine synthetase | NO | 0 | 347 |
|  | Y34_scaffold00706-25 | glutamine synthetase | NO | 0 | 358 |
|  | P131_scaffold00280-7 | glutamine synthetase | NO | 0 | 358 |
|  | P131_scaffold01268-6 | glutamine synthetase | NO | 0 | 347 |
| ORTHOMCL43 |  |  |  |  |  |
|  | supercontig_6.13-732 | triacylglycerol lipase FGL4 | YES | 0 | 556 |
|  | supercontig_6.23-325 | lipase | YES | 0 | 560 |
|  | Y34_scaffold00037-12 | triacylglycerol lipase FGL4 | YES | 0 | 556 |
|  | Y34_scaffold00301-57 | lipase | YES | 0 | 560 |
|  | P131_scaffold00265-24 | triacylglycerol lipase FGL4 | YES | 0 | 556 |
|  | P131_scaffold01387-4 | lipase | YES | 0 | 560 |
| ORTHOMCL44 |  |  |  |  |  |
|  | supercontig_6.13-927 | acetyl-coenzyme A synthetase | NO | 0 | 661 |
|  | supercontig_6.16-121 | acetyl-coenzyme A synthetase | NO | 1 | 639 |
|  | Y34_scaffold00765-11 | acetyl-coenzyme A synthetase | NO | 0 | 661 |
|  | Y34_scaffold01005-1 | acetyl-coenzyme A synthetase | NO | 1 | 691 |
|  | P131_scaffold00256-7 | acetyl-coenzyme A synthetase | NO | 0 | 661 |
|  | P131_scaffold01138-73 | acetyl-coenzyme A synthetase | NO | 1 | 691 |
| ORTHOMCL45 |  |  |  |  |  |
|  | supercontig_6.15-638 | L-amino-acid oxidase | NO | 0 | 785 |
|  | supercontig_6.27-947 | L-amino-acid oxidase | YES | 0 | 691 |
|  | Y34_scaffold00245-7 | L-amino-acid oxidase | YES | 0 | 691 |
|  | Y34_scaffold00445-12 | L-amino-acid oxidase | NO | 0 | 819 |
|  | P131_scaffold00252-17 | L-amino-acid oxidase | YES | 0 | 818 |
|  | P131_scaffold00295-12 | L-amino-acid oxidase | NO | 0 | 819 |
| ORTHOMCL46 |  |  |  |  |  |
|  | supercontig_6.12-7 | sterol 24-C-methyltransferase | NO | 0 | 390 |
|  | supercontig_6.28-379 | sterol 24-C-methyltransferase | NO | 0 | 392 |
|  | Y34_scaffold00069-5 | sterol 24-C-methyltransferase | NO | 0 | 390 |
|  | Y34_scaffold00193-1 | sterol 24-C-methyltransferase | NO | 0 | 306 |
|  | P131_scaffold00197-3 | sterol 24-C-methyltransferase | NO | 0 | 390 |
|  | P131_scaffold01380-3 | sterol 24-C-methyltransferase | NO | 0 | 392 |
| ORTHOMCL47 |  |  |  |  |  |
|  | supercontig_6.12-19 | catalase-peroxidase 1 | NO | 0 | 738 |
|  | supercontig_6.14-55 | catalase-peroxidase 1 | YES | 0 | 786 |
|  | Y34_scaffold00290-9 | catalase-peroxidase 1 | NO | 0 | 738 |
|  | Y34_scaffold00435-1 | catalase-peroxidase 1 | YES | 0 | 786 |
|  | P131_scaffold00197-15 | catalase-peroxidase 1 | NO | 0 | 738 |
|  | P131_scaffold00272-2 | catalase-peroxidase 1 | YES | 0 | 786 |
| ORTHOMCL48 |  |  |  |  |  |
|  | supercontig_6.24-439 | macrolide phosphotransferase k | NO | 13 | 583 |
|  | supercontig_6.29-711 | methylenomycin A resistance protein | NO | 14 | 577 |
|  | Y34_scaffold00138-15 | methylenomycin A resistance protein | NO | 14 | 577 |
|  | Y34_scaffold00807-5 | macrolide phosphotransferase k | NO | 13 | 583 |
|  | P131_scaffold00190-4 | macrolide phosphotransferase k | NO | 13 | 583 |
|  | P131_scaffold00329-7 | methylenomycin A resistance protein | NO | 14 | 577 |
| ORTHOMCL49 |  |  |  |  |  |
|  | supercontig_6.18-1311 | cytochrome P450 | NO | 0 | 572 |
|  | supercontig_6.8-183 | cytochrome P450 | NO | 0 | 554 |
|  | Y34_scaffold00133-1 | cytochrome P450 | NO | 0 | 554 |
|  | Y34_scaffold00517-16 | cytochrome P450 | YES | 0 | 591 |
|  | P131_scaffold00182-1 | cytochrome P450 | NO | 0 | 554 |
|  | P131_scaffold01772-12 | cytochrome P450 | NO | 0 | 417 |
| ORTHOMCL50 |  |  |  |  |  |
|  | supercontig_6.12-752 | HET-C domain-containing protein HetC | YES | 1 | 918 |
|  | supercontig_6.27-874 | HET-C domain-containing protein HetC | YES | 1 | 759 |
|  | Y34_scaffold00258-11 | hypothetical protein | YES | 1 | 901 |
|  | Y34_scaffold00657-12 | HET-C domain-containing protein HetC | YES | 1 | 759 |
|  | P131_scaffold00178-16 | HET-C domain-containing protein HetC | YES | 1 | 759 |
|  | P131_scaffold01043-9 | hypothetical protein | YES | 1 | 901 |
| ORTHOMCL51 |  |  |  |  |  |
|  | supercontig_6.18-1149 | amino-acid permease inda1 | NO | 11 | 571 |
|  | supercontig_6.21-1043 | amino-acid permease inda1 | NO | 11 | 577 |
|  | Y34_scaffold00216-7 | amino-acid permease inda1 | NO | 11 | 571 |
|  | Y34_scaffold00608-84 | amino-acid permease inda1 | NO | 11 | 577 |
|  | P131_scaffold00166-7 | amino-acid permease inda1 | NO | 11 | 571 |
|  | P131_scaffold00325-1 | amino-acid permease inda1 | NO | 11 | 577 |
| ORTHOMCL52 |  |  |  |  |  |
|  | supercontig_6.18-1146 | sodium P-type ATPase | NO | 8 | 1089 |
|  | supercontig_6.9-118 | sodium P-type ATPase | NO | 10 | 1094 |
|  | Y34_scaffold00216-10 | sodium P-type ATPase | NO | 8 | 1089 |
|  | Y34_scaffold00679-21 | sodium P-type ATPase | NO | 10 | 1094 |
|  | P131_scaffold00166-10 | sodium P-type ATPase | NO | 8 | 1089 |
|  | P131_scaffold01689-22 | sodium P-type ATPase | NO | 10 | 1094 |
| ORTHOMCL53 |  |  |  |  |  |
|  | supercontig_6.15-221 | polyketide synthase | NO | 0 | 2252 |
|  | supercontig_6.16-244 | fatty acid synthase S-acetyltransferase | NO | 0 | 2227 |
|  | Y34_scaffold00173-3 | polyketide synthase | NO | 0 | 2405 |
|  | Y34_scaffold00214-16 | fatty acid synthase S-acetyltransferase | NO | 0 | 2227 |
|  | P131_scaffold00150-8 | polyketide synthase | NO | 0 | 2354 |
|  | P131_scaffold00435-16 | fatty acid synthase S-acetyltransferase | NO | 0 | 2227 |
| ORTHOMCL54 |  |  |  |  |  |
|  | supercontig_6.21-632 | neutral ceramidase | YES | 1 | 770 |
|  | supercontig_6.29-249 | neutral ceramidase precursor | NO | 1 | 837 |
|  | Y34_scaffold00134-14 | neutral ceramidase | YES | 1 | 770 |
|  | Y34_scaffold00707-71 | neutral ceramidase precursor | NO | 1 | 837 |
|  | P131_scaffold00141-11 | neutral ceramidase precursor | NO | 1 | 651 |
|  | P131_scaffold00267-13 | neutral ceramidase | YES | 1 | 770 |
| ORTHOMCL56 |  |  |  |  |  |
|  | supercontig_6.12-439 | cellobiohydrolase precursor | YES | 0 | 449 |
|  | supercontig_6.28-129 | exoglucanase 1 precursor | YES | 0 | 448 |
|  | Y34_scaffold00180-1 | cellobiohydrolase precursor | YES | 0 | 449 |
|  | Y34_scaffold00873-1 | exoglucanase 1 precursor | YES | 0 | 448 |
|  | P131_scaffold00116-1 | exoglucanase 1 precursor | YES | 0 | 448 |
|  | P131_scaffold00477-5 | cellobiohydrolase precursor | YES | 0 | 449 |
| ORTHOMCL57 |  |  |  |  |  |
|  | supercontig_6.17-22 | DUF895 domain membrane protein | NO | 10 | 481 |
|  | supercontig_6.20-173 | DUF895 domain membrane protein | NO | 10 | 450 |
|  | Y34_scaffold00650-3 | DUF895 domain membrane protein | NO | 10 | 450 |
|  | Y34_scaffold00871-5 | DUF895 domain membrane protein | NO | 10 | 481 |
|  | P131_scaffold00111-5 | DUF895 domain membrane protein | NO | 10 | 481 |
|  | P131_scaffold01140-3 | DUF895 domain membrane protein | NO | 10 | 450 |
| ORTHOMCL58 |  |  |  |  |  |
|  | supercontig_6.16-84 | cellobiose dehydrogenase | YES | 0 | 1138 |
|  | supercontig_6.22-750 | cellobiose dehydrogenase | YES | 0 | 840 |
|  | Y34_scaffold00033-56 | cellobiose dehydrogenase | YES | 0 | 840 |
|  | Y34_scaffold01005-42 | cellobiose dehydrogenase | YES | 0 | 1138 |
|  | P131_scaffold00095-2 | cellobiose dehydrogenase | YES | 0 | 771 |
|  | P131_scaffold01138-36 | cellobiose dehydrogenase | YES | 0 | 1138 |
| ORTHOMCL59 |  |  |  |  |  |
|  | supercontig_6.14-4 | guanyl-nucleotide exchange factor | NO | 0 | 2409 |
|  | supercontig_6.14-5 | L-serine dehydratase | NO | 0 | 358 |
|  | Y34_scaffold00021-4 | guanyl-nucleotide exchange factor | NO | 0 | 2409 |
|  | Y34_scaffold00021-5 | L-serine dehydratase | NO | 0 | 358 |
|  | P131_scaffold00045-36 | NAD-dependent deacetylase sirtuin-2 | NO | 0 | 796 |
|  | P131_scaffold00119-4 | guanyl-nucleotide exchange factor | NO | 0 | 1872 |

Secreted, secreted proteins; TM, transmembrane domains.
