# Supplementary material for: Comparative Analysis of the Genomes of Two Field Isolates of the Rice Blast Fungus Magnaporthe oryzae
Source: PLoS Genet. 2012 Aug 2;8(8):e1002869. doi: 10.1371/journal.pgen.1002869 (PMC3410873; doi:10.1371/journal.pgen.1002869)
Supplement: Table S8 — Gene families with different numbers of members in each of the three isolates. (DOC) [file pgen.1002869.s016.doc]

**Table S8** Gene families with different numbers of members in each of the three isolates.

| **ORTHOMCL** | **Gene** | **Annotation** | **Secreted** | **TM** | **Protein length** |
| --- | --- | --- | --- | --- | --- |
| ORTHOMCL1 |  |  |  |  |  |
|  | supercontig_6.10-3 | hypothetical protein | NO | 1 | 1140 |
|  | supercontig_6.11-89 | hypothetical protein | NO | 1 | 666 |
|  | supercontig_6.11-92 | hypothetical protein | NO | 0 | 504 |
|  | supercontig_6.13-1 | hypothetical protein | NO | 1 | 1057 |
|  | supercontig_6.13-1189 | hypothetical protein | NO | 0 | 1525 |
|  | supercontig_6.14-114 | hypothetical protein | NO | 0 | 1531 |
|  | supercontig_6.19-4 | hypothetical protein | NO | 0 | 1464 |
|  | supercontig_6.20-2 | hypothetical protein | NO | 0 | 1446 |
|  | supercontig_6.28-412 | hypothetical protein | NO | 0 | 1597 |
|  | supercontig_6.6-25 | hypothetical protein | NO | 0 | 1737 |
|  | supercontig_6.8-221 | hypothetical protein | NO | 0 | 1394 |
|  | supercontig_6.9-156 | hypothetical protein | NO | 0 | 756 |
|  | Y34_scaffold00362-3 | hypothetical protein | NO | 0 | 1189 |
|  | P131_scaffold01532-1 | hypothetical protein | NO | 0 | 738 |
|  | P131_scaffold01793-1 | hypothetical protein | NO | 0 | 179 |
| ORTHOMCL3 |  |  |  |  |  |
|  | supercontig_6.23-275 | hypothetical protein | NO | 0 | 794 |
|  | Y34_scaffold00242-1 | hypothetical protein | NO | 0 | 629 |
|  | Y34_scaffold00311-12 | hypothetical protein | NO | 0 | 579 |
|  | Y34_scaffold00674-1 | hypothetical protein | NO | 0 | 515 |
|  | Y34_scaffold00863-4 | hypothetical protein | NO | 0 | 550 |
|  | Y34_scaffold01164-1 | hypothetical protein | NO | 0 | 443 |
|  | Y34_scaffold01167-5 | hypothetical protein | NO | 0 | 618 |
|  | Y34_scaffold01174-1 | hypothetical protein | NO | 0 | 448 |
|  | P131_scaffold00601-17 | hypothetical protein | NO | 0 | 784 |
|  | P131_scaffold01206-3 | hypothetical protein | NO | 0 | 661 |
|  | P131_scaffold01688-1 | hypothetical protein | NO | 0 | 396 |
| ORTHOMCL4 |  |  |  |  |  |
|  | supercontig_6.18-897 | ABC multidrug transporter Mdr1 | NO | 12 | 1269 |
|  | supercontig_6.21-1235 | multidrug resistance protein 1 | NO | 12 | 1333 |
|  | supercontig_6.4-195 | multidrug resistance protein 1 | NO | 12 | 1350 |
|  | supercontig_6.9-154 | multidrug resistance protein 1 | NO | 12 | 1350 |
|  | Y34_scaffold00129-6 | ABC multidrug transporter Mdr1 | NO | 12 | 1269 |
|  | Y34_scaffold00267-31 | ABC multidrug transporter Mdr1 | NO | 12 | 1333 |
|  | Y34_scaffold01046-1 | multidrug resistance protein 1 | NO | 12 | 1350 |
|  | P131_scaffold00525-31 | ABC multidrug transporter Mdr1 | NO | 12 | 1333 |
|  | P131_scaffold00896-1 | multidrug resistance protein 1 | NO | 12 | 1350 |
|  | P131_scaffold01609-3 | multidrug resistance protein 1 | NO | 12 | 1199 |
| ORTHOMCL5 |  |  |  |  |  |
|  | supercontig_6.13-335 | hypothetical protein | NO | 0 | 689 |
|  | supercontig_6.18-1166 | hypothetical protein | NO | 0 | 3019 |
|  | Y34_scaffold00014-1 | hypothetical protein | NO | 0 | 1208 |
|  | Y34_scaffold00264-2 | hypothetical protein | NO | 0 | 1448 |
|  | Y34_scaffold01173-2 | hypothetical protein | YES | 0 | 1501 |
|  | P131_scaffold00022-2 | hypothetical protein | NO | 0 | 2545 |
|  | P131_scaffold00460-2 | hypothetical protein | NO | 0 | 1448 |
|  | P131_scaffold00571-4 | hypothetical protein | YES | 1 | 828 |
|  | P131_scaffold01352-1 | hypothetical protein | NO | 0 | 662 |
|  | P131_scaffold01809-1 | hypothetical protein | NO | 0 | 598 |
| ORTHOMCL13 |  |  |  |  |  |
|  | supercontig_6.18-108 | C6 zinc finger domain-containing protein | NO | 0 | 1226 |
|  | Y34_scaffold00242-2 | C6 zinc finger domain-containing protein | NO | 0 | 2332 |
|  | Y34_scaffold00283-9 | C6 zinc finger domain-containing protein | NO | 0 | 1226 |
|  | Y34_scaffold00838-2 | C6 zinc finger domain-containing protein | NO | 0 | 939 |
|  | Y34_scaffold01087-1 | C6 zinc finger domain-containing protein | YES | 0 | 1035 |
|  | P131_scaffold00006-8 | C6 zinc finger domain-containing protein | NO | 0 | 1243 |
|  | P131_scaffold00165-1 | C6 zinc finger domain-containing protein | NO | 0 | 489 |
|  | P131_scaffold00629-6 | C6 zinc finger domain-containing protein | NO | 0 | 939 |
|  | P131_scaffold00929-2 | C6 zinc finger domain-containing protein | NO | 0 | 1226 |
| ORTHOMCL8 |  |  |  |  |  |
|  | supercontig_6.13-1182 | Amidase family protein | NO | 11 | 1116 |
|  | supercontig_6.13-1183 | C6 transcription factor | NO | 0 | 689 |
|  | supercontig_6.13-1184 | flavin-containing amine oxidase | NO | 0 | 343 |
|  | supercontig_6.13-6 | acetamidase | NO | 11 | 2159 |
|  | supercontig_6.26-100 | acetamidase | NO | 0 | 560 |
|  | Y34_scaffold00186-1 | flavin-containing amine oxidase | NO | 11 | 2392 |
|  | Y34_scaffold00448-57 | acetamidase | NO | 0 | 560 |
|  | P131_scaffold00333-8 | acetamidase | NO | 0 | 547 |
|  | P131_scaffold00585-3 | flavin-containing amine oxidase | NO | 11 | 2392 |
| ORTHOMCL14 |  |  |  |  |  |
|  | supercontig_6.24-517 | invertase | YES | 0 | 659 |
|  | supercontig_6.28-189 | invertase | YES | 0 | 610 |
|  | supercontig_6.9-136 | invertase | YES | 0 | 719 |
|  | Y34_scaffold00463-2 | invertase | YES | 0 | 677 |
|  | Y34_scaffold00679-3 | invertase | YES | 0 | 719 |
|  | Y34_scaffold01055-2 | invertase | NO | 0 | 497 |
|  | P131_scaffold00895-2 | invertase | YES | 0 | 659 |
|  | P131_scaffold01689-4 | invertase | YES | 0 | 719 |
| ORTHOMCL15 |  |  |  |  |  |
|  | supercontig_6.18-630 | putative calcium P-type ATPase | NO | 9 | 1447 |
|  | supercontig_6.22-216 | putative calcium P-type ATPase | NO | 8 | 1274 |
|  | Y34_scaffold00696-5 | putative calcium P-type ATPase | NO | 10 | 1276 |
|  | Y34_scaffold00712-33 | putative calcium P-type ATPase | NO | 9 | 1447 |
|  | Y34_scaffold00745-57 | putative calcium P-type ATPase | NO | 8 | 1274 |
|  | P131_scaffold00314-29 | putative calcium P-type ATPase | NO | 8 | 1274 |
|  | P131_scaffold00546-33 | putative calcium P-type ATPase | NO | 9 | 1447 |
|  | P131_scaffold00611-1 | putative calcium P-type ATPase | NO | 10 | 1276 |
| ORTHOMCL16 |  |  |  |  |  |
|  | supercontig_6.12-62 | MFS transporter | NO | 13 | 560 |
|  | supercontig_6.28-163 | MFS transporter | NO | 13 | 560 |
|  | supercontig_6.9-90 | MFS transporter | NO | 12 | 593 |
|  | Y34_scaffold00717-6 | MFS transporter | NO | 12 | 593 |
|  | Y34_scaffold00858-2 | MFS transporter | NO | 13 | 560 |
|  | P131_scaffold01372-3 | MFS transporter | NO | 12 | 593 |
|  | P131_scaffold01617-1 | MFS transporter | NO | 12 | 538 |
| ORTHOMCL17 |  |  |  |  |  |
|  | supercontig_6.15-177 | hypothetical protein | NO | 9 | 485 |
|  | supercontig_6.17-19 | hypothetical protein | NO | 9 | 485 |
|  | Y34_scaffold00859-1 | hypothetical protein | NO | 5 | 394 |
|  | Y34_scaffold00871-2 | hypothetical protein | NO | 9 | 485 |
|  | P131_scaffold00111-2 | hypothetical protein | NO | 9 | 485 |
|  | P131_scaffold01506-2 | hypothetical protein | NO | 6 | 489 |
|  | P131_scaffold01735-1 | hypothetical protein | NO | 6 | 434 |
| ORTHOMCL18 |  |  |  |  |  |
|  | supercontig_6.21-1415 | LPS glycosyltransferase | YES | 1 | 393 |
|  | supercontig_6.23-902 | LPS glycosyltransferase | YES | 1 | 376 |
|  | supercontig_6.29-915 | LPS glycosyltransferase | YES | 1 | 345 |
|  | Y34_scaffold00300-3 | LPS glycosyltransferase | YES | 1 | 393 |
|  | Y34_scaffold00969-52 | LPS glycosyltransferase | YES | 1 | 393 |
|  | P131_scaffold00060-1 | LPS glycosyltransferase | YES | 1 | 393 |
|  | P131_scaffold01816-1 | LPS glycosyltransferase | YES | 1 | 393 |
| ORTHOMCL25 |  |  |  |  |  |
|  | supercontig_6.12-923 | carbonic anhydrase 2 | NO | 0 | 518 |
|  | supercontig_6.28-142 | carbonic anhydrase 2 | NO | 0 | 516 |
|  | supercontig_6.4-188 | carbonic anhydrase 2 | NO | 0 | 850 |
|  | supercontig_6.6-12 | carbonic anhydrase 2 | NO | 0 | 850 |
|  | Y34_scaffold00446-1 | carbonic anhydrase 2 | NO | 0 | 642 |
|  | P131_scaffold01191-1 | carbonic anhydrase 2 | NO | 0 | 759 |
| ORTHOMCL55 |  |  |  |  |  |
|  | supercontig_6.12-366 | BCS1-like ATPase | NO | 1 | 610 |
|  | supercontig_6.18-1167 | BCS1-like ATPase | NO | 0 | 566 |
|  | Y34_scaffold00514-68 | BCS1-like ATPase | NO | 1 | 610 |
|  | P131_scaffold00134-26 | BCS1-like ATPase | NO | 1 | 610 |
|  | P131_scaffold00592-1 | BCS1-like ATPase | NO | 0 | 339 |
|  | P131_scaffold01676-1 | BCS1-like ATPase | NO | 0 | 319 |
| ORTHOMCL102 |  |  |  |  |  |
|  | supercontig_6.18-1193 | hypothetical protein | NO | 0 | 699 |
|  | Y34_scaffold00870-7 | hypothetical protein | NO | 0 | 455 |
|  | Y34_scaffold01033-2 | hypothetical protein | NO | 0 | 458 |
|  | P131_scaffold00208-6 | hypothetical protein | NO | 0 | 351 |
|  | P131_scaffold00682-1 | hypothetical protein | NO | 0 | 584 |
| ORTHOMCL103 |  |  |  |  |  |
|  | supercontig_6.21-1299 | hypothetical protein | YES | 7 | 572 |
|  | supercontig_6.28-338 | hypothetical protein | NO | 7 | 599 |
|  | Y34_scaffold00833-10 | hypothetical protein | NO | 7 | 599 |
|  | Y34_scaffold00901-5 | hypothetical protein | YES | 7 | 572 |
|  | P131_scaffold00203-10 | hypothetical protein | NO | 7 | 599 |
| ORTHOMCL104 |  |  |  |  |  |
|  | supercontig_6.26-105 | hypothetical protein | NO | 3 | 589 |
|  | Y34_scaffold00100-4 | hypothetical protein | NO | 3 | 591 |
|  | Y34_scaffold00448-62 | hypothetical protein | NO | 3 | 589 |
|  | P131_scaffold00113-1 | hypothetical protein | YES | 2 | 454 |
|  | P131_scaffold00333-3 | hypothetical protein | NO | 3 | 589 |
| ORTHOMCL106 |  |  |  |  |  |
|  | supercontig_6.13-705 | hypothetical protein | NO | 6 | 746 |
|  | Y34_scaffold00037-39 | hypothetical protein | NO | 6 | 746 |
|  | Y34_scaffold00982-2 | hypothetical protein | NO | 6 | 685 |
|  | Y34_scaffold00992-1 | hypothetical protein | YES | 5 | 532 |
|  | P131_scaffold00082-27 | hypothetical protein | NO | 6 | 746 |
| ORTHOMCL108 |  |  |  |  |  |
|  | supercontig_6.11-98 | cytochrome P450 monooxygenase | NO | 1 | 523 |
|  | supercontig_6.21-1307 | cytochrome P450 monooxygenase | NO | 2 | 541 |
|  | Y34_scaffold00918-1 | cytochrome P450 monooxygenase | NO | 2 | 541 |
|  | Y34_scaffold01072-1 | cytochrome P450 monooxygenase | NO | 1 | 576 |
|  | P131_scaffold00049-1 | cytochrome P450 monooxygenase | NO | 0 | 351 |
| ORTHOMCL61 |  |  |  |  |  |
|  | supercontig_6.2-4 | hypothetical protein | NO | 0 | 1145 |
|  | supercontig_6.23-110 | hypothetical protein | NO | 0 | 883 |
|  | supercontig_6.6-20 | hypothetical protein | NO | 0 | 815 |
|  | Y34_scaffold00313-1 | hypothetical protein | NO | 0 | 1130 |
|  | P131_scaffold01696-1 | hypothetical protein | NO | 0 | 863 |
| ORTHOMCL62 |  |  |  |  |  |
|  | supercontig_6.15-52 | hypothetical protein | NO | 0 | 313 |
|  | supercontig_6.4-199 | hypothetical protein | NO | 0 | 326 |
|  | supercontig_6.7-106 | hypothetical protein | NO | 0 | 313 |
|  | Y34_scaffold00671-2 | hypothetical protein | YES | 0 | 335 |
|  | P131_scaffold01526-3 | hypothetical protein | NO | 0 | 263 |
| ORTHOMCL76 |  |  |  |  |  |
|  | supercontig_6.8-219 | hypothetical protein | NO | 0 | 412 |
|  | supercontig_6.8-66 | hypothetical protein | NO | 0 | 412 |
|  | supercontig_6.8-72 | hypothetical protein | NO | 0 | 412 |
|  | Y34_scaffold00827-1 | hypothetical protein | NO | 0 | 373 |
|  | P131_scaffold01013-1 | hypothetical protein | NO | 0 | 412 |
| ORTHOMCL78 |  |  |  |  |  |
|  | supercontig_6.13-227 | beta-1 | YES | 0 | 621 |
|  | Y34_scaffold00722-1 | beta-1 | YES | 0 | 621 |
|  | Y34_scaffold01147-3 | beta-1 | NO | 0 | 557 |
|  | P131_scaffold00981-6 | beta-1 | YES | 0 | 621 |
|  | P131_scaffold01671-3 | beta-1 | NO | 0 | 557 |
| ORTHOMCL83 |  |  |  |  |  |
|  | supercontig_6.18-1301 | ent-copalyl diphosphate/ent-kaurene synthase | NO | 0 | 962 |
|  | Y34_scaffold00146-2 | ent-copalyl diphosphate/ent-kaurene synthase | NO | 0 | 727 |
|  | Y34_scaffold00517-10 | ent-copalyl diphosphate/ent-kaurene synthase | NO | 0 | 962 |
|  | P131_scaffold00634-1 | ent-copalyl diphosphate/ent-kaurene synthase | NO | 0 | 727 |
|  | P131_scaffold01772-6 | ent-copalyl diphosphate/ent-kaurene synthase | NO | 0 | 962 |
| ORTHOMCL84 |  |  |  |  |  |
|  | supercontig_6.29-845 | reducing polyketide synthase | NO | 0 | 2362 |
|  | Y34_scaffold00766-2 | reducing polyketide synthase | NO | 0 | 2325 |
|  | Y34_scaffold01043-1 | reducing polyketide synthase | NO | 0 | 866 |
|  | P131_scaffold00600-2 | reducing polyketide synthase | NO | 0 | 2360 |
|  | P131_scaffold01491-4 | reducing polyketide synthase | NO | 0 | 573 |
| ORTHOMCL88 |  |  |  |  |  |
|  | supercontig_6.9-24 | ankyrin repeat protein | NO | 1 | 999 |
|  | Y34_scaffold00531-10 | ankyrin repeat protein | NO | 1 | 631 |
|  | Y34_scaffold00535-1 | hypothetical protein | NO | 0 | 331 |
|  | P131_scaffold00396-1 | hypothetical protein | NO | 0 | 331 |
|  | P131_scaffold01357-1 | ankyrin repeat protein | NO | 0 | 467 |
| ORTHOMCL97 |  |  |  |  |  |
|  | supercontig_6.23-890 | extracellular alpha-1 | YES | 0 | 906 |
|  | supercontig_6.28-290 | hypothetical protein | YES | 3 | 804 |
|  | Y34_scaffold00692-23 | hypothetical protein | YES | 3 | 804 |
|  | P131_scaffold00279-14 | hypothetical protein | YES | 3 | 804 |
|  | P131_scaffold00381-1 | extracellular alpha-1 | YES | 0 | 916 |
| ORTHOMCL112 |  |  |  |  |  |
|  | supercontig_6.21-1413 | hypothetical protein | YES | 0 | 133 |
|  | supercontig_6.29-917 | hypothetical protein | YES | 0 | 133 |
|  | Y34_scaffold00300-1 | hypothetical protein | YES | 0 | 133 |
|  | P131_scaffold01816-3 | hypothetical protein | YES | 0 | 133 |
| ORTHOMCL113 |  |  |  |  |  |
|  | supercontig_6.21-1414 | glycoside hydrolase family 62 | YES | 0 | 459 |
|  | supercontig_6.23-904 | glycoside hydrolase family 62 | YES | 0 | 390 |
|  | Y34_scaffold00969-51 | putative arabinofuranosidase | YES | 0 | 271 |
|  | P131_scaffold01816-2 | alpha-N-arabinofuranosidase | NO | 0 | 339 |
| ORTHOMCL114 |  |  |  |  |  |
|  | supercontig_6.17-60 | hypothetical protein | NO | 0 | 468 |
|  | supercontig_6.7-98 | hypothetical protein | NO | 0 | 468 |
|  | Y34_scaffold00890-2 | hypothetical protein | NO | 0 | 468 |
|  | P131_scaffold01810-5 | hypothetical protein | NO | 0 | 394 |
| ORTHOMCL115 |  |  |  |  |  |
|  | supercontig_6.17-58 | elongation factor 2 kinase | NO | 0 | 239 |
|  | supercontig_6.7-96 | elongation factor 2 kinase | NO | 0 | 239 |
|  | Y34_scaffold00890-4 | elongation factor 2 kinase | NO | 0 | 239 |
|  | P131_scaffold01810-4 | elongation factor 2 kinase | NO | 0 | 239 |
| ORTHOMCL116 |  |  |  |  |  |
|  | supercontig_6.17-56 | hypothetical protein | NO | 3 | 210 |
|  | supercontig_6.7-94 | hypothetical protein | NO | 3 | 210 |
|  | Y34_scaffold00890-6 | hypothetical protein | NO | 3 | 210 |
|  | P131_scaffold01810-2 | hypothetical protein | NO | 3 | 210 |
| ORTHOMCL117 |  |  |  |  |  |
|  | supercontig_6.18-1164 | MAP kinase kinase skh1/pek1 | NO | 0 | 707 |
|  | Y34_scaffold00870-3 | MAP kinase kinase skh1/pek1 | NO | 0 | 631 |
|  | Y34_scaffold01101-5 | MAP kinase kinase skh1/pek1 | NO | 0 | 671 |
|  | P131_scaffold01804-3 | MAP kinase kinase skh1/pek1 | NO | 0 | 734 |
| ORTHOMCL118 |  |  |  |  |  |
|  | supercontig_6.12-747 | N-acetyltransferase family protein | NO | 0 | 285 |
|  | Y34_scaffold00252-5 | N-acetyltransferase family protein | NO | 0 | 371 |
|  | Y34_scaffold00375-1 | N-acetyltransferase family protein | NO | 0 | 173 |
|  | P131_scaffold01780-1 | N-acetyltransferase family protein | NO | 0 | 308 |
| ORTHOMCL119 |  |  |  |  |  |
|  | supercontig_6.18-1206 | JmjC domain-containing histone demethylation protein 3D | NO | 1 | 1112 |
|  | Y34_scaffold00079-2 | JmjC domain-containing histone demethylation protein 3D | NO | 0 | 610 |
|  | Y34_scaffold00676-12 | JmjC domain-containing histone demethylation protein 3D | NO | 1 | 1112 |
|  | P131_scaffold01768-8 | JmjC domain-containing histone demethylation protein 3D | NO | 1 | 1112 |
| ORTHOMCL120 |  |  |  |  |  |
|  | supercontig_6.12-70 | dynamin GTPase | NO | 0 | 520 |
|  | supercontig_6.18-1179 | dynamin GTPase | NO | 0 | 639 |
|  | Y34_scaffold00004-1 | dynamin GTPase | NO | 0 | 679 |
|  | P131_scaffold01740-9 | dynamin GTPase | NO | 0 | 520 |
| ORTHOMCL121 |  |  |  |  |  |
|  | supercontig_6.16-256 | carbonic anhydrase 2 | NO | 0 | 178 |
|  | supercontig_6.18-461 | carbonic anhydrase 2 | NO | 0 | 502 |
|  | Y34_scaffold01185-1 | hypothetical protein | NO | 0 | 596 |
|  | P131_scaffold01722-1 | hypothetical protein | NO | 0 | 667 |
| ORTHOMCL122 |  |  |  |  |  |
|  | supercontig_6.18-1192 | related to histone-lysine N-methyltransferase | NO | 0 | 419 |
|  | Y34_scaffold00823-1 | related to histone-lysine N-methyltransferase | NO | 0 | 419 |
|  | Y34_scaffold00857-1 | related to histone-lysine N-methyltransferase | NO | 0 | 464 |
|  | P131_scaffold01715-1 | related to histone-lysine N-methyltransferase | NO | 0 | 148 |
| ORTHOMCL123 |  |  |  |  |  |
|  | supercontig_6.3-2 | no match | NO | 0 | 58 |
|  | supercontig_6.31-9 | no match | NO | 0 | 58 |
|  | Y34_scaffold01091-2 | no match | NO | 0 | 58 |
|  | P131_scaffold01678-11 | no match | NO | 0 | 58 |
| ORTHOMCL125 |  |  |  |  |  |
|  | supercontig_6.28-147 | hypothetical protein | NO | 0 | 415 |
|  | supercontig_6.8-213 | hypothetical protein | NO | 0 | 415 |
|  | Y34_scaffold00361-1 | hypothetical protein | NO | 0 | 415 |
|  | P131_scaffold01620-1 | hypothetical protein | NO | 0 | 415 |
| ORTHOMCL126 |  |  |  |  |  |
|  | supercontig_6.13-287 | no match | NO | 0 | 44 |
|  | supercontig_6.25-254 | no match | NO | 0 | 44 |
|  | Y34_scaffold00490-1 | no match | NO | 0 | 44 |
|  | P131_scaffold01538-6 | no match | NO | 0 | 78 |
| ORTHOMCL127 |  |  |  |  |  |
|  | supercontig_6.18-1241 | ankyrin repeat protein | NO | 0 | 566 |
|  | Y34_scaffold00870-6 | ankyrin repeat protein | NO | 0 | 789 |
|  | Y34_scaffold01189-3 | ankyrin repeat protein | NO | 0 | 731 |
|  | P131_scaffold01537-6 | ankyrin repeat protein | NO | 0 | 566 |
| ORTHOMCL128 |  |  |  |  |  |
|  | supercontig_6.8-195 | hypothetical protein | NO | 0 | 89 |
|  | Y34_scaffold00841-8 | hypothetical protein | NO | 1 | 100 |
|  | Y34_scaffold00972-4 | hypothetical protein | NO | 0 | 510 |
|  | P131_scaffold01536-3 | hypothetical protein | NO | 0 | 488 |
| ORTHOMCL130 |  |  |  |  |  |
|  | supercontig_6.4-197 | hypothetical protein | NO | 0 | 535 |
|  | supercontig_6.6-17 | hypothetical protein | NO | 0 | 438 |
|  | Y34_scaffold01194-2 | hypothetical protein | NO | 0 | 487 |
|  | P131_scaffold01526-1 | hypothetical protein | NO | 0 | 521 |
| ORTHOMCL131 |  |  |  |  |  |
|  | supercontig_6.17-61 | hypothetical protein | NO | 0 | 199 |
|  | supercontig_6.7-99 | hypothetical protein | NO | 0 | 199 |
|  | Y34_scaffold00890-1 | hypothetical protein | NO | 0 | 199 |
|  | P131_scaffold01524-1 | hypothetical protein | YES | 0 | 210 |
| ORTHOMCL134 |  |  |  |  |  |
|  | supercontig_6.15-527 | no match | NO | 0 | 73 |
|  | supercontig_6.17-7 | no match | NO | 0 | 73 |
|  | Y34_scaffold00810-3 | no match | NO | 0 | 73 |
|  | P131_scaffold01312-6 | no match | NO | 0 | 73 |
| ORTHOMCL135 |  |  |  |  |  |
|  | supercontig_6.15-524 | carboxypeptidase | YES | 0 | 549 |
|  | supercontig_6.17-4 | carboxypeptidase | YES | 0 | 549 |
|  | Y34_scaffold00059-3 | carboxypeptidase | YES | 0 | 549 |
|  | P131_scaffold01312-3 | carboxypeptidase | YES | 0 | 549 |
| ORTHOMCL136 |  |  |  |  |  |
|  | supercontig_6.15-523 | hypothetical protein | YES | 0 | 72 |
|  | supercontig_6.17-3 | hypothetical protein | YES | 0 | 72 |
|  | Y34_scaffold00059-2 | hypothetical protein | YES | 0 | 72 |
|  | P131_scaffold01312-2 | hypothetical protein | YES | 0 | 72 |
| ORTHOMCL137 |  |  |  |  |  |
|  | supercontig_6.15-522 | hypothetical protein | YES | 0 | 162 |
|  | supercontig_6.17-2 | hypothetical protein | YES | 0 | 162 |
|  | Y34_scaffold00059-1 | hypothetical protein | YES | 0 | 162 |
|  | P131_scaffold01312-1 | hypothetical protein | YES | 0 | 162 |
| ORTHOMCL140 |  |  |  |  |  |
|  | supercontig_6.10-152 | no match | NO | 0 | 132 |
|  | supercontig_6.10-282 | no match | NO | 0 | 132 |
|  | Y34_scaffold00181-3 | no match | NO | 0 | 132 |
|  | P131_scaffold01270-3 | no match | NO | 0 | 132 |
| ORTHOMCL142 |  |  |  |  |  |
|  | supercontig_6.11-72 | rerric reductase like transmembrane component | YES | 7 | 499 |
|  | Y34_scaffold00792-3 | cell surface metalloreductase FreA-like | YES | 6 | 452 |
|  | Y34_scaffold00845-4 | cell surface metalloreductase | NO | 2 | 261 |
|  | P131_scaffold01248-1 | cell surface metalloreductase | NO | 2 | 261 |
| ORTHOMCL143 |  |  |  |  |  |
|  | supercontig_6.18-1171 | hypothetical protein | NO | 0 | 79 |
|  | Y34_scaffold01152-2 | CAMK family protein kinase | NO | 0 | 706 |
|  | P131_scaffold01245-2 | CAMK family protein kinase | NO | 0 | 707 |
|  | P131_scaffold01740-3 | hypothetical protein | NO | 0 | 79 |
| ORTHOMCL147 |  |  |  |  |  |
|  | supercontig_6.20-532 | C6 zinc finger domain-containing protein | NO | 0 | 354 |
|  | supercontig_6.20-96 | C6 zinc finger domain-containing protein | NO | 0 | 527 |
|  | Y34_scaffold00791-6 | C6 zinc finger domain-containing protein | NO | 0 | 471 |
|  | P131_scaffold01169-6 | C6 zinc finger domain-containing protein | NO | 0 | 471 |
| ORTHOMCL149 |  |  |  |  |  |
|  | supercontig_6.11-45 | hypothetical protein | YES | 0 | 126 |
|  | supercontig_6.8-210 | hypothetical protein | YES | 0 | 126 |
|  | Y34_scaffold00232-1 | hypothetical protein | YES | 0 | 126 |
|  | P131_scaffold01050-1 | hypothetical protein | YES | 0 | 126 |
| ORTHOMCL150 |  |  |  |  |  |
|  | supercontig_6.20-4 | hypothetical protein | NO | 0 | 43 |
|  | supercontig_6.23-755 | hypothetical protein | NO | 0 | 43 |
|  | Y34_scaffold00420-2 | hypothetical protein | NO | 0 | 43 |
|  | P131_scaffold01020-2 | hypothetical protein | NO | 0 | 43 |
| ORTHOMCL151 |  |  |  |  |  |
|  | supercontig_6.20-5 | hypothetical protein | NO | 0 | 67 |
|  | supercontig_6.23-756 | hypothetical protein | NO | 0 | 67 |
|  | Y34_scaffold00420-1 | hypothetical protein | NO | 0 | 67 |
|  | P131_scaffold01020-1 | hypothetical protein | NO | 0 | 67 |
| ORTHOMCL152 |  |  |  |  |  |
|  | supercontig_6.28-134 | hypothetical protein | YES | 0 | 85 |
|  | supercontig_6.28-169 | hypothetical protein | YES | 0 | 85 |
|  | Y34_scaffold00525-3 | hypothetical protein | YES | 0 | 85 |
|  | P131_scaffold00997-3 | hypothetical protein | YES | 0 | 85 |
| ORTHOMCL154 |  |  |  |  |  |
|  | supercontig_6.25-71 | hypothetical protein | NO | 0 | 706 |
|  | supercontig_6.7-76 | hypothetical protein | NO | 0 | 797 |
|  | Y34_scaffold00104-4 | hypothetical protein | NO | 0 | 829 |
|  | P131_scaffold00951-4 | hypothetical protein | NO | 0 | 497 |
| ORTHOMCL156 |  |  |  |  |  |
|  | supercontig_6.13-1187 | sugar transporter STL1 | YES | 12 | 512 |
|  | supercontig_6.13-3 | sugar transporter STL1 | YES | 12 | 512 |
|  | Y34_scaffold00840-1 | sugar transporter STL1 | YES | 12 | 512 |
|  | P131_scaffold00585-6 | sugar transporter STL1 | YES | 12 | 490 |
| ORTHOMCL157 |  |  |  |  |  |
|  | supercontig_6.13-1186 | gluconolactonase precursor | NO | 0 | 335 |
|  | supercontig_6.13-4 | gluconolactonase precursor | NO | 0 | 335 |
|  | Y34_scaffold00840-2 | gluconolactonase precursor | NO | 0 | 335 |
|  | P131_scaffold00585-4 | gluconolactonase precursor | NO | 0 | 313 |
| ORTHOMCL158 |  |  |  |  |  |
|  | supercontig_6.13-1181 | class II Aldolase and Adducin N-terminal domain protein | NO | 0 | 297 |
|  | supercontig_6.13-7 | class II Aldolase and Adducin N-terminal domain protein | NO | 0 | 297 |
|  | Y34_scaffold00186-2 | class II Aldolase and Adducin N-terminal domain protein | NO | 0 | 297 |
|  | P131_scaffold00585-2 | class II Aldolase and Adducin N-terminal domain protein | NO | 0 | 297 |
| ORTHOMCL159 |  |  |  |  |  |
|  | supercontig_6.13-1180 | hypothetical protein | NO | 0 | 146 |
|  | supercontig_6.13-8 | hypothetical protein | NO | 0 | 146 |
|  | Y34_scaffold00186-3 | hypothetical protein | YES | 0 | 88 |
|  | P131_scaffold00585-1 | hypothetical protein | YES | 0 | 88 |
| ORTHOMCL160 |  |  |  |  |  |
|  | supercontig_6.11-80 | hypothetical protein | NO | 0 | 424 |
|  | Y34_scaffold01036-7 | hypothetical protein | NO | 0 | 380 |
|  | P131_scaffold00571-3 | hypothetical protein | NO | 0 | 511 |
|  | P131_scaffold01597-7 | hypothetical protein | NO | 0 | 380 |
| ORTHOMCL161 |  |  |  |  |  |
|  | supercontig_6.15-520 | cyclin CCL1 | NO | 0 | 1144 |
|  | supercontig_6.17-37 | cyclin CCL1 | NO | 0 | 544 |
|  | Y34_scaffold00651-19 | cyclin CCL1 | NO | 0 | 1103 |
|  | P131_scaffold00557-9 | cyclin CCL1 | NO | 0 | 1103 |
| ORTHOMCL162 |  |  |  |  |  |
|  | supercontig_6.15-521 | ATP-dependent Zn protease | NO | 0 | 474 |
|  | supercontig_6.17-1 | ATP-dependent Zn protease | NO | 0 | 412 |
|  | Y34_scaffold00651-20 | ATP-dependent Zn protease | NO | 0 | 474 |
|  | P131_scaffold00557-10 | ATP-dependent Zn protease | NO | 0 | 474 |
| ORTHOMCL163 |  |  |  |  |  |
|  | supercontig_6.13-760 | related to monooxigenase | NO | 0 | 612 |
|  | supercontig_6.28-251 | related to monooxigenase | NO | 0 | 557 |
|  | Y34_scaffold00182-12 | related to monooxigenase | NO | 0 | 612 |
|  | P131_scaffold00539-14 | related to monooxigenase | NO | 0 | 612 |
| ORTHOMCL164 |  |  |  |  |  |
|  | supercontig_6.18-1159 | kinesin light chain | NO | 0 | 873 |
|  | Y34_scaffold00099-31 | kinesin light chain | NO | 0 | 776 |
|  | P131_scaffold00489-4 | kinesin light chain | NO | 0 | 587 |
|  | P131_scaffold01754-1 | kinesin light chain | NO | 0 | 357 |
| ORTHOMCL167 |  |  |  |  |  |
|  | supercontig_6.13-1079 | hypothetical protein | NO | 0 | 472 |
|  | supercontig_6.18-1163 | hypothetical protein | NO | 0 | 401 |
|  | Y34_scaffold00726-98 | hypothetical protein | NO | 0 | 472 |
|  | P131_scaffold00459-31 | hypothetical protein | NO | 0 | 433 |
| ORTHOMCL168 |  |  |  |  |  |
|  | supercontig_6.20-29 | E3 ubiquitin-protein ligase MARCH6 | NO | 17 | 1817 |
|  | Y34_scaffold00019-2 | E3 ubiquitin-protein ligase MARCH6 | NO | 7 | 781 |
|  | Y34_scaffold00734-6 | E3 ubiquitin-protein ligase MARCH6 | NO | 17 | 1817 |
|  | P131_scaffold00449-8 | E3 ubiquitin-protein ligase MARCH6 | NO | 17 | 1817 |
| ORTHOMCL169 |  |  |  |  |  |
|  | supercontig_6.15-182 | hypothetical protein | NO | 0 | 337 |
|  | supercontig_6.17-27 | hypothetical protein | NO | 0 | 337 |
|  | Y34_scaffold00056-2 | hypothetical protein | NO | 0 | 616 |
|  | P131_scaffold00427-4 | hypothetical protein | NO | 0 | 617 |
| ORTHOMCL170 |  |  |  |  |  |
|  | supercontig_6.15-181 | no match | NO | 0 | 41 |
|  | supercontig_6.17-28 | no match | NO | 0 | 41 |
|  | Y34_scaffold00056-1 | no match | NO | 0 | 41 |
|  | P131_scaffold00427-3 | no match | NO | 0 | 41 |
| ORTHOMCL171 |  |  |  |  |  |
|  | supercontig_6.15-180 | hypothetical protein | NO | 0 | 108 |
|  | supercontig_6.17-29 | hypothetical protein | NO | 0 | 108 |
|  | Y34_scaffold00928-2 | hypothetical protein | NO | 0 | 108 |
|  | P131_scaffold00427-2 | hypothetical protein | NO | 0 | 108 |
| ORTHOMCL172 |  |  |  |  |  |
|  | supercontig_6.15-179 | hypothetical protein | YES | 1 | 142 |
|  | supercontig_6.17-30 | hypothetical protein | YES | 1 | 142 |
|  | Y34_scaffold00928-1 | hypothetical protein | YES | 1 | 142 |
|  | P131_scaffold00427-1 | hypothetical protein | YES | 1 | 142 |
| ORTHOMCL175 |  |  |  |  |  |
|  | supercontig_6.25-52 | FAD binding domain containing protein | NO | 0 | 503 |
|  | Y34_scaffold00854-4 | FAD binding domain containing protein | NO | 0 | 503 |
|  | P131_scaffold00383-4 | FAD binding domain containing protein | NO | 0 | 503 |
|  | P131_scaffold01023-1 | FAD binding domain containing protein | YES | 0 | 519 |
| ORTHOMCL177 |  |  |  |  |  |
|  | supercontig_6.17-9 | hypothetical protein | YES | 0 | 115 |
|  | supercontig_6.18-954 | hypothetical protein | YES | 0 | 115 |
|  | Y34_scaffold00047-1 | hypothetical protein | YES | 0 | 115 |
|  | P131_scaffold00364-13 | hypothetical protein | YES | 0 | 115 |
| ORTHOMCL181 |  |  |  |  |  |
|  | supercontig_6.28-140 | hypothetical protein | NO | 0 | 622 |
|  | supercontig_6.28-177 | hypothetical protein | NO | 0 | 622 |
|  | Y34_scaffold00763-1 | hypothetical protein | NO | 0 | 239 |
|  | P131_scaffold00309-9 | hypothetical protein | NO | 0 | 1029 |
| ORTHOMCL182 |  |  |  |  |  |
|  | supercontig_6.28-141 | no match | NO | 1 | 132 |
|  | supercontig_6.28-178 | no match | NO | 1 | 132 |
|  | Y34_scaffold00763-2 | no match | NO | 1 | 132 |
|  | P131_scaffold00309-8 | no match | NO | 1 | 132 |
| ORTHOMCL183 |  |  |  |  |  |
|  | supercontig_6.28-137 | predicted protein | YES | 1 | 161 |
|  | supercontig_6.28-173 | predicted protein | YES | 1 | 161 |
|  | Y34_scaffold00879-3 | predicted protein | YES | 1 | 161 |
|  | P131_scaffold00309-12 | predicted protein | YES | 1 | 161 |
| ORTHOMCL184 |  |  |  |  |  |
|  | supercontig_6.28-138 | hypothetical protein | NO | 0 | 601 |
|  | supercontig_6.28-175 | hypothetical protein | NO | 0 | 573 |
|  | Y34_scaffold00879-4 | hypothetical protein | NO | 0 | 140 |
|  | P131_scaffold00309-10 | hypothetical protein | NO | 0 | 533 |
| ORTHOMCL186 |  |  |  |  |  |
|  | supercontig_6.14-98 | hypothetical protein | YES | 0 | 156 |
|  | supercontig_6.6-23 | hypothetical protein | YES | 0 | 156 |
|  | Y34_scaffold00220-1 | hypothetical protein | YES | 0 | 156 |
|  | P131_scaffold00296-1 | hypothetical protein | YES | 0 | 156 |
| ORTHOMCL196 |  |  |  |  |  |
|  | supercontig_6.15-184 | hypothetical protein | NO | 0 | 535 |
|  | supercontig_6.17-25 | hypothetical protein | NO | 0 | 535 |
|  | Y34_scaffold00871-8 | hypothetical protein | NO | 0 | 426 |
|  | P131_scaffold00111-8 | hypothetical protein | NO | 0 | 426 |
| ORTHOMCL197 |  |  |  |  |  |
|  | supercontig_6.15-611 | hypothetical protein | NO | 0 | 211 |
|  | supercontig_6.17-20 | hypothetical protein | NO | 0 | 211 |
|  | Y34_scaffold00871-3 | hypothetical protein | NO | 0 | 211 |
|  | P131_scaffold00111-3 | hypothetical protein | NO | 0 | 211 |
| ORTHOMCL198 |  |  |  |  |  |
|  | supercontig_6.18-880 | hypothetical protein | NO | 0 | 131 |
|  | supercontig_6.18-933 | hypothetical protein | YES | 0 | 300 |
|  | Y34_scaffold00469-2 | hypothetical protein | NO | 0 | 131 |
|  | P131_scaffold00088-4 | hypothetical protein | YES | 0 | 300 |
| ORTHOMCL199 |  |  |  |  |  |
|  | supercontig_6.15-171 | hypothetical protein | NO | 0 | 88 |
|  | supercontig_6.15-174 | hypothetical protein | NO | 0 | 88 |
|  | Y34_scaffold00445-20 | hypothetical protein | NO | 0 | 88 |
|  | P131_scaffold00078-1 | hypothetical protein | NO | 0 | 88 |
| ORTHOMCL200 |  |  |  |  |  |
|  | supercontig_6.15-49 | hypothetical protein | YES | 0 | 643 |
|  | supercontig_6.7-103 | hypothetical protein | YES | 0 | 577 |
|  | Y34_scaffold00365-2 | hypothetical protein | YES | 0 | 492 |
|  | P131_scaffold00074-1 | hypothetical protein | YES | 0 | 512 |
| ORTHOMCL202 |  |  |  |  |  |
|  | supercontig_6.25-73 | hypothetical protein | NO | 0 | 942 |
|  | Y34_scaffold01053-4 | hypothetical protein | NO | 0 | 886 |
|  | P131_scaffold00032-4 | hypothetical protein | NO | 0 | 831 |
|  | P131_scaffold00951-1 | hypothetical protein | NO | 0 | 1059 |
| ORTHOMCL203 |  |  |  |  |  |
|  | supercontig_6.13-337 | hypothetical protein | NO | 0 | 726 |
|  | Y34_scaffold01089-1 | hypothetical protein | NO | 0 | 675 |
|  | P131_scaffold00032-2 | hypothetical protein | NO | 0 | 696 |
|  | P131_scaffold01264-1 | hypothetical protein | NO | 0 | 597 |
| ORTHOMCL204 |  |  |  |  |  |
|  | supercontig_6.29-852 | non-reducing polyketide synthase | NO | 0 | 2202 |
|  | supercontig_6.30-14 | non-reducing polyketide synthase | NO | 0 | 1280 |
|  | Y34_scaffold00555-1 | non-reducing polyketide synthase | NO | 0 | 2286 |
|  | P131_scaffold00009-1 | non-reducing polyketide synthase | NO | 0 | 2233 |

Secreted, secreted proteins; TM, transmembrane domains.
