# Supplementary material for: Comparative Analysis of the Genomes of Two Field Isolates of the Rice Blast Fungus Magnaporthe oryzae
Source: PLoS Genet. 2012 Aug 2;8(8):e1002869. doi: 10.1371/journal.pgen.1002869 (PMC3410873; doi:10.1371/journal.pgen.1002869)
Supplement: Table S9 — Gene families specific to two of the three isolates. (DOC) [file pgen.1002869.s017.doc]

**Table S9** Gene families specific to two of the three isolates.

| **ORTHOMCL** | **Gene** | **Annotation** | **Secreted** | **TM** | **Protein length** |
| --- | --- | --- | --- | --- | --- |
| ORTHOMCL109 |  |  |  |  |  |
|  | Y34_scaffold00639-1 | hypothetical protein | NO | 0 | 618 |
|  | Y34_scaffold01002-1 | hypothetical protein | NO | 0 | 885 |
|  | P131_scaffold00019-1 | hypothetical protein | NO | 0 | 1075 |
|  | P131_scaffold00513-1 | hypothetical protein | NO | 0 | 584 |
|  | P131_scaffold01614-3 | hypothetical protein | NO | 0 | 378 |
| ORTHOMCL132 |  |  |  |  |  |
|  | Y34_scaffold00858-3 | no match | NO | 0 | 25 |
|  | Y34_scaffold00960-1 | no match | NO | 0 | 25 |
|  | P131_scaffold01470-1 | no match | NO | 0 | 25 |
|  | P131_scaffold01617-2 | no match | NO | 0 | 25 |
| ORTHOMCL1000 |  |  |  |  |  |
|  | Y34_scaffold00804-6 | hypothetical protein | NO | 0 | 699 |
|  | Y34_scaffold00846-7 | hypothetical protein | YES | 0 | 628 |
|  | P131_scaffold01409-2 | hypothetical protein | NO | 0 | 502 |
| ORTHOMCL1001 |  |  |  |  |  |
|  | Y34_scaffold00846-8 | hypothetical protein | NO | 0 | 515 |
|  | P131_scaffold01409-1 | hypothetical protein | YES | 0 | 274 |
|  | P131_scaffold01783-1 | hypothetical protein | YES | 0 | 274 |
| ORTHOMCL225 |  |  |  |  |  |
|  | Y34_scaffold00862-1 | no match | NO | 2 | 153 |
|  | Y34_scaffold01171-6 | no match | NO | 0 | 171 |
|  | P131_scaffold01821-4 | no match | YES | 2 | 206 |
| ORTHOMCL2864 |  |  |  |  |  |
|  | Y34_scaffold00558-1 | hypothetical protein | NO | 0 | 542 |
|  | Y34_scaffold00869-1 | hypothetical protein | NO | 0 | 413 |
|  | P131_scaffold01206-1 | hypothetical protein | NO | 0 | 457 |
| ORTHOMCL915 |  |  |  |  |  |
|  | Y34_scaffold00846-5 | similar to calcium/calmodulin-dependent protein kinase I | NO | 0 | 434 |
|  | Y34_scaffold01115-3 | similar to calcium/calmodulin-dependent protein kinase I | NO | 0 | 570 |
|  | P131_scaffold01513-2 | similar to calcium/calmodulin-dependent protein kinase I | NO | 0 | 556 |
| ORTHOMCL998 |  |  |  |  |  |
|  | Y34_scaffold00853-1 | hypothetical protein | NO | 0 | 375 |
|  | P131_scaffold01424-1 | hypothetical protein | NO | 0 | 615 |
|  | P131_scaffold01677-6 | hypothetical protein | NO | 0 | 622 |
| ORTHOMCL110 |  |  |  |  |  |
|  | supercontig_6.13-338 | hypothetical protein | NO | 0 | 186 |
|  | supercontig_6.13-340 | hypothetical protein | NO | 0 | 186 |
|  | Y34_scaffold01039-2 | hypothetical protein | NO | 0 | 273 |
|  | Y34_scaffold01093-6 | hypothetical protein | NO | 0 | 273 |
| ORTHOMCL111 |  |  |  |  |  |
|  | supercontig_6.10-1 | DEAD/DEAH box helicase | NO | 0 | 84 |
|  | supercontig_6.13-1190 | DEAD/DEAH box helicase | NO | 0 | 84 |
|  | supercontig_6.20-1 | DEAD/DEAH box helicase | NO | 0 | 84 |
|  | Y34_scaffold00362-4 | DEAD/DEAH box helicase | NO | 0 | 84 |
| ORTHOMCL209 |  |  |  |  |  |
|  | supercontig_6.12-59 | hypothetical protein | NO | 0 | 71 |
|  | supercontig_6.28-160 | hypothetical protein | NO | 0 | 71 |
|  | Y34_scaffold01183-2 | hypothetical protein | NO | 0 | 71 |
| ORTHOMCL210 |  |  |  |  |  |
|  | supercontig_6.17-59 | no match | NO | 2 | 227 |
|  | supercontig_6.7-97 | no match | NO | 2 | 227 |
|  | Y34_scaffold00972-3 | no match | NO | 0 | 154 |
| ORTHOMCL211 |  |  |  |  |  |
|  | supercontig_6.21-1334 | no match | YES | 0 | 96 |
|  | supercontig_6.21-1399 | no match | YES | 0 | 96 |
|  | Y34_scaffold00947-2 | no match | YES | 0 | 96 |
| ORTHOMCL212 |  |  |  |  |  |
|  | supercontig_6.18-1379 | no match | NO | 0 | 132 |
|  | supercontig_6.21-1301 | no match | NO | 0 | 132 |
|  | Y34_scaffold00901-7 | no match | NO | 0 | 132 |
| ORTHOMCL213 |  |  |  |  |  |
|  | supercontig_6.28-139 | hypothetical protein | NO | 0 | 358 |
|  | supercontig_6.28-176 | hypothetical protein | NO | 0 | 358 |
|  | Y34_scaffold00879-5 | hypothetical protein | NO | 0 | 141 |
| ORTHOMCL214 |  |  |  |  |  |
|  | supercontig_6.12-57 | hypothetical protein | YES | 1 | 145 |
|  | supercontig_6.28-158 | hypothetical protein | YES | 1 | 145 |
|  | Y34_scaffold00879-1 | hypothetical protein | YES | 1 | 145 |
| ORTHOMCL215 |  |  |  |  |  |
|  | supercontig_6.18-1235 | no match | NO | 0 | 31 |
|  | Y34_scaffold00857-4 | no match | NO | 0 | 116 |
|  | Y34_scaffold01189-8 | no match | YES | 0 | 49 |
| ORTHOMCL216 |  |  |  |  |  |
|  | supercontig_6.11-71 | MFS transporter | NO | 13 | 510 |
|  | supercontig_6.9-149 | MFS transporter | NO | 13 | 510 |
|  | Y34_scaffold00792-1 | MFS transporter | NO | 13 | 509 |
| ORTHOMCL218 |  |  |  |  |  |
|  | supercontig_6.21-1321 | hypothetical protein | NO | 0 | 72 |
|  | supercontig_6.9-103 | hypothetical protein | NO | 0 | 72 |
|  | Y34_scaffold00317-1 | hypothetical protein | NO | 0 | 72 |
| ORTHOMCL221 |  |  |  |  |  |
|  | supercontig_6.7-116 | no match | NO | 0 | 41 |
|  | Y34_scaffold00025-1 | no match | NO | 0 | 102 |
|  | Y34_scaffold01084-1 | no match | NO | 0 | 139 |
| ORTHOMCL124 |  |  |  |  |  |
|  | supercontig_6.28-157 | sterigmatocystin 8-O-methyltransferase | NO | 0 | 371 |
|  | supercontig_6.4-193 | sterigmatocystin 8-O-methyltransferase | NO | 0 | 371 |
|  | supercontig_6.8-62 | sterigmatocystin 8-O-methyltransferase | NO | 0 | 371 |
|  | P131_scaffold01666-2 | sterigmatocystin 8-O-methyltransferase | NO | 0 | 371 |
| ORTHOMCL129 |  |  |  |  |  |
|  | supercontig_6.15-51 | hypothetical protein | YES | 0 | 116 |
|  | supercontig_6.4-198 | hypothetical protein | YES | 0 | 116 |
|  | supercontig_6.7-105 | hypothetical protein | YES | 0 | 116 |
|  | P131_scaffold01526-2 | hypothetical protein | YES | 0 | 116 |
| ORTHOMCL227 |  |  |  |  |  |
|  | supercontig_6.11-102 | no match | NO | 0 | 54 |
|  | supercontig_6.12-834 | no match | NO | 0 | 54 |
|  | P131_scaffold01785-1 | no match | NO | 0 | 54 |
| ORTHOMCL3150 |  |  |  |  |  |
|  | supercontig_6.4-189 | WD-repeat protein | NO | 0 | 444 |
|  | supercontig_6.6-11 | WD-repeat protein | NO | 0 | 485 |
|  | P131_scaffold01191-2 | WD-repeat protein | NO | 0 | 444 |
| ORTHOMCL5524 |  |  |  |  |  |
|  | supercontig_6.28-130 | hypothetical protein | YES | 0 | 115 |
|  | supercontig_6.28-165 | hypothetical protein | YES | 0 | 115 |
|  | P131_scaffold00914-1 | hypothetical protein | YES | 0 | 115 |

Secreted, secreted proteins; TM, transmembrane domains.
