# Supplementary material for: Comparative Analysis of the Genomes of Two Field Isolates of the Rice Blast Fungus Magnaporthe oryzae
Source: PLoS Genet. 2012 Aug 2;8(8):e1002869. doi: 10.1371/journal.pgen.1002869 (PMC3410873; doi:10.1371/journal.pgen.1002869)
Supplement: Table S10 — Isolate-specific gene families. (DOC) [file pgen.1002869.s018.doc]

**Table S10** Isolate-specific gene families.

| **ORTHOMCL** | **Gene** | **Annotation** | **Secreted** | **TM** | **Protein length** |
| --- | --- | --- | --- | --- | --- |
| ORTHOMCL60 |  |  |  |  |  |
|  | supercontig_6.16-273 | hypothetical protein | NO | 2 | 219 |
|  | supercontig_6.16-274 | hypothetical protein | NO | 0 | 217 |
|  | supercontig_6.29-896 | hypothetical protein | NO | 0 | 544 |
|  | supercontig_6.7-100 | hypothetical protein | NO | 0 | 228 |
|  | supercontig_6.7-101 | hypothetical protein | NO | 0 | 219 |
| ORTHOMCL207 |  |  |  |  |  |
|  | supercontig_6.28-415 | no match | NO | 2 | 146 |
|  | supercontig_6.28-419 | no match | NO | 0 | 81 |
|  | supercontig_6.28-420 | no match | NO | 0 | 81 |
| ORTHOMCL208 |  |  |  |  |  |
|  | supercontig_6.14-111 | no match | NO | 0 | 38 |
|  | supercontig_6.4-196 | no match | NO | 0 | 38 |
|  | supercontig_6.6-16 | no match | NO | 0 | 38 |
| ORTHOMCL11444 |  |  |  |  |  |
|  | supercontig_6.8-65 | hypothetical protein | NO | 0 | 33 |
|  | supercontig_6.8-73 | hypothetical protein | NO | 0 | 33 |
| ORTHOMCL11445 |  |  |  |  |  |
|  | supercontig_6.29-890 | no match | NO | 0 | 44 |
|  | supercontig_6.29-895 | no match | NO | 0 | 44 |
| ORTHOMCL11446 |  |  |  |  |  |
|  | supercontig_6.21-1324 | no match | NO | 0 | 29 |
|  | supercontig_6.8-155 | no match | NO | 0 | 29 |
| ORTHOMCL11447 |  |  |  |  |  |
|  | supercontig_6.2-15 | hypothetical protein | NO | 0 | 119 |
|  | supercontig_6.21-1410 | hypothetical protein | NO | 0 | 119 |
| ORTHOMCL11448 |  |  |  |  |  |
|  | supercontig_6.18-1378 | no match | YES | 0 | 33 |
|  | supercontig_6.21-1302 | no match | YES | 0 | 33 |
| ORTHOMCL11449 |  |  |  |  |  |
|  | supercontig_6.17-62 | hypothetical protein | NO | 0 | 115 |
|  | supercontig_6.7-88 | hypothetical protein | NO | 0 | 115 |
| ORTHOMCL11450 |  |  |  |  |  |
|  | supercontig_6.17-54 | hypothetical protein | NO | 0 | 236 |
|  | supercontig_6.7-92 | hypothetical protein | NO | 0 | 236 |
| ORTHOMCL11451 |  |  |  |  |  |
|  | supercontig_6.17-52 | phosphotransferase family protein | NO | 0 | 305 |
|  | supercontig_6.7-90 | phosphotransferase family protein | NO | 0 | 305 |
| ORTHOMCL11452 |  |  |  |  |  |
|  | supercontig_6.17-51 | hypothetical protein | NO | 0 | 138 |
|  | supercontig_6.7-89 | hypothetical protein | NO | 0 | 138 |
| ORTHOMCL11453 |  |  |  |  |  |
|  | supercontig_6.15-526 | hypothetical protein | NO | 0 | 156 |
|  | supercontig_6.17-6 | hypothetical protein | NO | 0 | 156 |
| ORTHOMCL11454 |  |  |  |  |  |
|  | supercontig_6.15-183 | hypothetical protein | NO | 0 | 263 |
|  | supercontig_6.17-26 | hypothetical protein | NO | 0 | 263 |
| ORTHOMCL11455 |  |  |  |  |  |
|  | supercontig_6.12-919 | hypothetical protein | YES | 0 | 208 |
|  | supercontig_6.16-262 | hypothetical protein | YES | 0 | 208 |
| ORTHOMCL11456 |  |  |  |  |  |
|  | supercontig_6.12-63 | bcs1 AAA-type ATPase | NO | 0 | 289 |
|  | supercontig_6.28-164 | bcs1 AAA-type ATPase | NO | 0 | 269 |
| ORTHOMCL11457 |  |  |  |  |  |
|  | supercontig_6.11-88 | DEAD/DEAH box helicase | NO | 1 | 83 |
|  | supercontig_6.28-413 | DEAD/DEAH box helicase | NO | 1 | 83 |
| ORTHOMCL11458 |  |  |  |  |  |
|  | supercontig_6.11-73 | hypothetical protein | NO | 0 | 144 |
|  | supercontig_6.12-66 | hypothetical protein | NO | 0 | 164 |
| ORTHOMCL217 |  |  |  |  |  |
|  | Y34_scaffold00374-2 | hypothetical protein | NO | 0 | 220 |
|  | Y34_scaffold00860-1 | hypothetical protein | NO | 0 | 160 |
|  | Y34_scaffold01188-1 | hypothetical protein | NO | 0 | 447 |
| ORTHOMCL219 |  |  |  |  |  |
|  | Y34_scaffold00305-4 | no match | NO | 0 | 138 |
|  | Y34_scaffold01190-2 | no match | NO | 0 | 175 |
|  | Y34_scaffold01191-1 | no match | NO | 0 | 90 |
| ORTHOMCL220 |  |  |  |  |  |
|  | Y34_scaffold00116-6 | hypothetical protein | NO | 0 | 185 |
|  | Y34_scaffold00300-4 | hypothetical protein | NO | 0 | 336 |
|  | Y34_scaffold01032-2 | hypothetical protein | NO | 0 | 332 |
| ORTHOMCL11467 |  |  |  |  |  |
|  | Y34_scaffold01101-1 | hypothetical protein | NO | 0 | 327 |
|  | Y34_scaffold01184-4 | hypothetical protein | NO | 0 | 532 |
| ORTHOMCL11473 |  |  |  |  |  |
|  | Y34_scaffold01023-1 | no match | NO | 0 | 114 |
|  | Y34_scaffold01115-7 | no match | NO | 0 | 197 |
| ORTHOMCL11489 |  |  |  |  |  |
|  | Y34_scaffold00877-2 | no match | NO | 0 | 278 |
|  | Y34_scaffold01039-5 | no match | NO | 0 | 80 |
| ORTHOMCL11490 |  |  |  |  |  |
|  | Y34_scaffold00862-3 | hypothetical protein | NO | 0 | 156 |
|  | Y34_scaffold01171-1 | hypothetical protein | NO | 0 | 181 |
| ORTHOMCL11548 |  |  |  |  |  |
|  | Y34_scaffold00623-1 | no match | YES | 0 | 84 |
|  | Y34_scaffold00820-7 | no match | YES | 0 | 86 |
| ORTHOMCL11588 |  |  |  |  |  |
|  | Y34_scaffold00499-3 | hypothetical protein | NO | 0 | 483 |
|  | Y34_scaffold00982-6 | C2H2 transcription factor | NO | 0 | 702 |
| ORTHOMCL11601 |  |  |  |  |  |
|  | Y34_scaffold00439-1 | hypothetical protein | NO | 0 | 276 |
|  | Y34_scaffold00964-1 | hypothetical protein | NO | 0 | 104 |
| ORTHOMCL11622 |  |  |  |  |  |
|  | Y34_scaffold00311-10 | JmjC domain-containing histone demethylation protein | NO | 0 | 996 |
|  | Y34_scaffold01175-9 | JmjC domain-containing histone demethylation protein | NO | 0 | 1032 |
| ORTHOMCL11683 |  |  |  |  |  |
|  | Y34_scaffold00019-1 | no match | NO | 0 | 72 |
|  | Y34_scaffold00588-4 | no match | NO | 0 | 114 |
| ORTHOMCL11685 |  |  |  |  |  |
|  | Y34_scaffold00001-1 | kinesin light chain 3 | NO | 0 | 117 |
|  | Y34_scaffold01145-2 | kinesin light chain 3 | NO | 0 | 115 |
| ORTHOMCL206 |  |  |  |  |  |
|  | P131_scaffold00002-1 | no match | NO | 2 | 238 |
|  | P131_scaffold00006-3 | no match | NO | 5 | 396 |
|  | P131_scaffold01764-2 | no match | NO | 4 | 282 |
|  | P131_scaffold01773-3 | no match | NO | 3 | 313 |
| ORTHOMCL11333 |  |  |  |  |  |
|  | P131_scaffold00032-5 | hypothetical protein | NO | 0 | 218 |
|  | P131_scaffold00513-2 | hypothetical protein | NO | 0 | 394 |
|  | P131_scaffold00581-2 | hypothetical protein | NO | 0 | 210 |
| ORTHOMCL11707 |  |  |  |  |  |
|  | P131_scaffold01745-1 | no match | NO | 0 | 35 |
|  | P131_scaffold01779-1 | no match | NO | 0 | 35 |
| ORTHOMCL11717 |  |  |  |  |  |
|  | P131_scaffold01730-1 | related to amino-terminal amidase | NO | 0 | 508 |
|  | P131_scaffold01741-1 | related to amino-terminal amidase | NO | 0 | 483 |
| ORTHOMCL11773 |  |  |  |  |  |
|  | P131_scaffold01579-6 | no match | NO | 0 | 145 |
|  | P131_scaffold01777-8 | no match | NO | 0 | 138 |
| ORTHOMCL11809 |  |  |  |  |  |
|  | P131_scaffold01531-1 | hypothetical protein | NO | 0 | 245 |
|  | P131_scaffold01742-1 | hypothetical protein | NO | 0 | 257 |
| ORTHOMCL11829 |  |  |  |  |  |
|  | P131_scaffold01491-1 | no match | NO | 0 | 119 |
|  | P131_scaffold01781-1 | no match | NO | 0 | 115 |
| Secreted, secreted proteins; TM, transmembrane domains. | | | | | |
