# Supplementary material for: Comparative Analysis of the Genomes of Two Field Isolates of the Rice Blast Fungus Magnaporthe oryzae
Source: PLoS Genet. 2012 Aug 2;8(8):e1002869. doi: 10.1371/journal.pgen.1002869 (PMC3410873; doi:10.1371/journal.pgen.1002869)
Supplement: Table S11 — The number of the genes identical between Y34 and P131 but with nucleotide variations in 70-15 and which showed evidence of exposure to diversifying and purifying selection pressures based on GO classification. (DOC) [file pgen.1002869.s019.doc]

**Table S11** The number of the genes identical between Y34 and P131 but with nucleotide variations in 70-15 and which showed evidence of exposure to diversifying and purifying selection pressures based on GO classification.

| **GO category** | **Diversifying selection** | **Purifying selection** |
| --- | --- | --- |
| 0044238 primary metabolic process | 107 | 110 |
| 0044237 cellular metabolic process | 94 | 98 |
| 0043170 macromolecule metabolic process | 65 | 69 |
| 0006807 nitrogen compound metabolic process | 64 | 53 |
| 0009058 biosynthetic process | 47 | 48 |
| 0055114 oxidation-reduction process | 45 | 44 |
| 0006810 transport | 38 | 27 |
| 0044281 small molecule metabolic process | 35 | 29 |
| 0019222 regulation of metabolic process | 34 | 19 |
| 0055085 transmembrane transport | 30 | 22 |
| 0009056 catabolic process | 22 | 19 |
| 0051716 cellular response to stimulus | 17 | 6 |
| 0065009 regulation of molecular function | 11 | 2 |
| 0051641 cellular localization | 7 | 4 |
| 0006950 response to stress | 5 | 4 |
| 0019637 organophosphate metabolic process | 5 | 0 |
| 0033036 macromolecule localization | 4 | 4 |
| 0045184 establishment of protein localization | 4 | 4 |
