# Supplementary material for: Comparative Analysis of the Genomes of Two Field Isolates of the Rice Blast Fungus Magnaporthe oryzae
Source: PLoS Genet. 2012 Aug 2;8(8):e1002869. doi: 10.1371/journal.pgen.1002869 (PMC3410873; doi:10.1371/journal.pgen.1002869)
Supplement: Table S12 — Genes with only asynonymous nucleotide substitutions in field isolates P131 and Y34 compared with the laboratory strain 70-15. (DOC) [file pgen.1002869.s020.doc]

**Table S12** Genes with only asynonymous nucleotide substitutions in field isolates P131 and Y34 compared with the laboratory strain 70-15.

| **70-15 gene** | **Y34 gene** | **P131 gene** | **Annotation** |
| --- | --- | --- | --- |
| supercontig_6.10-111 | Y34_scaffold00111-1 | P131_scaffold01362-1 | hypothetical protein |
| supercontig_6.10-120 | Y34_scaffold00892-5 | P131_scaffold00445-7 | hypothetical protein |
| supercontig_6.10-170 | Y34_scaffold00370-15 | P131_scaffold01302-25 | hypothetical protein |
| supercontig_6.10-191 | Y34_scaffold00370-36 | P131_scaffold01302-4 | mRNA cleavage factor complex component Pcf11 |
| supercontig_6.10-215 | Y34_scaffold00211-18 | P131_scaffold00216-1 | armadillo repeat protein |
| supercontig_6.10-227 | Y34_scaffold00211-5 | P131_scaffold01119-3 | endoglucanase E precursor |
| supercontig_6.10-247 | Y34_scaffold00983-4 | P131_scaffold00942-7 | ankyrin 2 |
| supercontig_6.10-258 | Y34_scaffold00159-8 | P131_scaffold01637-1 | thiol protease |
| supercontig_6.10-32 | Y34_scaffold00456-25 | P131_scaffold00986-1 | terpene synthase metal binding domain protein |
| supercontig_6.10-50 | Y34_scaffold00222-13 | P131_scaffold00511-6 | hypothetical protein |
| supercontig_6.10-90 | Y34_scaffold00628-15 | P131_scaffold00599-2 | F-box domain-containing protein |
| supercontig_6.11-28 | Y34_scaffold00688-4 | P131_scaffold00994-2 | no_hit |
| supercontig_6.11-32 | Y34_scaffold00660-5 | P131_scaffold00610-21 | hypothetical protein |
| supercontig_6.11-33 | Y34_scaffold00660-4 | P131_scaffold00610-20 | hypothetical protein |
| supercontig_6.11-7 | Y34_scaffold00109-8 | P131_scaffold00610-4 | hypothetical protein |
| supercontig_6.11-96 | Y34_scaffold00577-7 | P131_scaffold01391-1 | hypothetical protein |
| supercontig_6.12-142 | Y34_scaffold00540-34 | P131_scaffold01261-5 | no_hit |
| supercontig_6.12-169 | Y34_scaffold00540-7 | P131_scaffold01393-47 | hypothetical protein |
| supercontig_6.12-171 | Y34_scaffold00540-5 | P131_scaffold01393-45 | hypothetical protein |
| supercontig_6.12-185 | Y34_scaffold00302-6 | P131_scaffold01393-31 | ankyrin repeat containing protein |
| supercontig_6.12-186 | Y34_scaffold00302-5 | P131_scaffold01393-30 | hypothetical protein |
| supercontig_6.12-194 | Y34_scaffold00579-10 | P131_scaffold01393-22 | hypothetical protein |
| supercontig_6.12-198 | Y34_scaffold00579-6 | P131_scaffold01393-18 | salicylate hydroxylase |
| supercontig_6.12-201 | Y34_scaffold00579-3 | P131_scaffold01393-15 | hypothetical protein |
| supercontig_6.12-205 | Y34_scaffold00255-1 | P131_scaffold01393-12 | dishevelled |
| supercontig_6.12-207 | Y34_scaffold00255-3 | P131_scaffold01393-10 | polyamine transporter 2 |
| supercontig_6.12-251 | Y34_scaffold00255-48 | P131_scaffold00606-1 | fatty acid synthase alpha subunit reductase |
| supercontig_6.12-257 | Y34_scaffold00255-54 | P131_scaffold01349-26 | ribose 5-phosphate isomerase-like protein |
| supercontig_6.12-259 | Y34_scaffold00255-56 | P131_scaffold01349-24 | hypothetical protein |
| supercontig_6.12-26 | Y34_scaffold00290-16 | P131_scaffold00197-22 | CBS domain-containing protein |
| supercontig_6.12-262 | Y34_scaffold00255-59 | P131_scaffold01349-21 | transcriptional activator protein acu-15 |
| supercontig_6.12-263 | Y34_scaffold00255-60 | P131_scaffold01349-20 | SET domain-containing protein |
| supercontig_6.12-264 | Y34_scaffold00255-61 | P131_scaffold01349-19 | GTP-binding protein |
| supercontig_6.12-27 | Y34_scaffold00290-17 | P131_scaffold00197-23 | MFS transporter |
| supercontig_6.12-274 | Y34_scaffold00255-71 | P131_scaffold01349-9 | hypothetical protein |
| supercontig_6.12-29 | Y34_scaffold00290-19 | P131_scaffold00197-25 | hypothetical protein |
| supercontig_6.12-319 | Y34_scaffold00514-115 | P131_scaffold01683-37 | TRS120 targeting complex (TRAPP) component involved in ER to Golgi membrane traffic |
| supercontig_6.12-32 | Y34_scaffold00290-22 | P131_scaffold01143-3 | WSC domain-containing protein |
| supercontig_6.12-344 | Y34_scaffold00514-89 | P131_scaffold01683-11 | hypothetical protein |
| supercontig_6.12-346 | Y34_scaffold00514-87 | P131_scaffold01683-9 | hypothetical protein |
| supercontig_6.12-385 | Y34_scaffold00514-49 | P131_scaffold00134-7 | CorA family metal ion transporter |
| supercontig_6.12-394 | Y34_scaffold00514-41 | P131_scaffold00186-11 | tuberin |
| supercontig_6.12-40 | Y34_scaffold00290-31 | P131_scaffold01143-12 | hypothetical protein |
| supercontig_6.12-411 | Y34_scaffold00514-24 | P131_scaffold00188-1 | no_hit |
| supercontig_6.12-441 | Y34_scaffold00251-2 | P131_scaffold00477-3 | cyanide hydratase |
| supercontig_6.12-447 | Y34_scaffold00251-8 | P131_scaffold01185-4 | guanyl-specific ribonuclease F1 |
| supercontig_6.12-463 | Y34_scaffold00248-20 | P131_scaffold00559-21 | hypothetical protein |
| supercontig_6.12-49 | Y34_scaffold00290-40 | P131_scaffold01143-21 | nuclear membrane fusion protein Kar5 |
| supercontig_6.12-510 | Y34_scaffold00487-70 | P131_scaffold00303-28 | MYB DNA binding protein |
| supercontig_6.12-511 | Y34_scaffold00487-69 | P131_scaffold00303-29 | hypothetical protein |
| supercontig_6.12-521 | Y34_scaffold00487-59 | P131_scaffold01181-3 | hypothetical protein |
| supercontig_6.12-523 | Y34_scaffold00487-57 | P131_scaffold01181-5 | histidine acid phosphatase |
| supercontig_6.12-544 | Y34_scaffold00487-36 | P131_scaffold01331-6 | pumilio-family RNA binding repeat containing protein |
| supercontig_6.12-577 | Y34_scaffold00487-3 | P131_scaffold01331-39 | DUF1445 domain-containing protein |
| supercontig_6.12-598 | Y34_scaffold00085-19 | P131_scaffold01331-60 | hypothetical protein |
| supercontig_6.12-605 | Y34_scaffold01069-2 | P131_scaffold01576-4 | peptidase family T4 protein |
| supercontig_6.12-620 | Y34_scaffold00578-3 | P131_scaffold00282-3 | hypothetical protein |
| supercontig_6.12-622 | Y34_scaffold00578-1 | P131_scaffold00282-1 | hypothetical protein |
| supercontig_6.12-635 | Y34_scaffold00898-1 | P131_scaffold00105-5 | hypothetical protein |
| supercontig_6.12-671 | Y34_scaffold00092-3 | P131_scaffold00368-1 | hypothetical protein |
| supercontig_6.12-672 | Y34_scaffold00092-4 | P131_scaffold00368-2 | toxin biosynthesis protein Tri7-like |
| supercontig_6.12-681 | Y34_scaffold00092-12 | P131_scaffold00368-10 | small nuclear ribonucleoprotein |
| supercontig_6.12-713 | Y34_scaffold00432-9 | P131_scaffold00434-1 | 3-oxoacyl-(acyl-carrier-protein) reductase |
| supercontig_6.12-717 | Y34_scaffold00432-5 | P131_scaffold00434-5 | aldose 1-epimerase |
| supercontig_6.12-736 | Y34_scaffold00658-6 | P131_scaffold01314-8 | MACRO domain-containing protein |
| supercontig_6.12-737 | Y34_scaffold00658-7 | P131_scaffold01314-7 | glutaminyl-tRNA synthetase |
| supercontig_6.12-740 | Y34_scaffold00658-10 | P131_scaffold01314-4 | hypothetical protein |
| supercontig_6.12-784 | Y34_scaffold00485-1 | P131_scaffold01258-15 | hypothetical protein |
| supercontig_6.12-791 | Y34_scaffold00547-6 | P131_scaffold01258-9 | penicillopepsin |
| supercontig_6.12-8 | Y34_scaffold00069-4 | P131_scaffold00197-4 | cytochrome P450 monooxygenase |
| supercontig_6.12-827 | Y34_scaffold00251-17 | P131_scaffold01185-13 | no_hit |
| supercontig_6.12-831 | Y34_scaffold00251-13 | P131_scaffold01185-9 | nitrate reductase |
| supercontig_6.12-866 | Y34_scaffold00514-13 | P131_scaffold01204-9 | hypothetical protein |
| supercontig_6.12-880 | Y34_scaffold00691-10 | P131_scaffold00147-18 | hypothetical protein |
| supercontig_6.12-908 | Y34_scaffold00427-8 | P131_scaffold00147-1 | hypothetical protein |
| supercontig_6.12-950 | Y34_scaffold00905-1 | P131_scaffold00755-1 | ABC transporter |
| supercontig_6.12-960 | Y34_scaffold01031-2 | P131_scaffold00506-2 | hypothetical protein |
| supercontig_6.12-997 | Y34_scaffold00789-4 | P131_scaffold00392-3 | hypothetical protein |
| supercontig_6.13-1012 | Y34_scaffold00765-95 | P131_scaffold01311-52 | nucleolar protein |
| supercontig_6.13-1017 | Y34_scaffold00765-100 | P131_scaffold01311-57 | SH3 domain containing protein |
| supercontig_6.13-1028 | Y34_scaffold00765-111 | P131_scaffold00317-20 | pathway-specific nitrogen regulator |
| supercontig_6.13-1038 | Y34_scaffold00765-120 | P131_scaffold00317-10 | related to RING finger protein Dorfin |
| supercontig_6.13-1060 | Y34_scaffold00709-1 | P131_scaffold00459-12 | WD domain |
| supercontig_6.13-108 | Y34_scaffold00720-2 | P131_scaffold00943-20 | GTP binding protein |
| supercontig_6.13-1085 | Y34_scaffold00726-92 | P131_scaffold00459-37 | oligopeptide transporter 2 |
| supercontig_6.13-1123 | Y34_scaffold00726-53 | P131_scaffold01377-15 | hypothetical protein |
| supercontig_6.13-1129 | Y34_scaffold00726-47 | P131_scaffold01377-21 | CUE domain protein |
| supercontig_6.13-1135 | Y34_scaffold00726-42 | P131_scaffold00358-16 | Putative sensor/transporter protein involved in cell wall biogenesis |
| supercontig_6.13-1147 | Y34_scaffold00726-30 | P131_scaffold00358-4 | hypothetical protein |
| supercontig_6.13-1150 | Y34_scaffold00726-27 | P131_scaffold00358-1 | hypothetical protein |
| supercontig_6.13-1154 | Y34_scaffold00726-23 | P131_scaffold00149-25 | DnaJ domain containing protein |
| supercontig_6.13-1156 | Y34_scaffold00726-21 | P131_scaffold00149-23 | methyltransferase small domain protein |
| supercontig_6.13-1170 | Y34_scaffold00726-7 | P131_scaffold00149-9 | cyclopropane-fatty-acyl-phospholipid synthase |
| supercontig_6.13-1175 | Y34_scaffold00726-3 | P131_scaffold00149-3 | immunoglobulin I-set domain protein |
| supercontig_6.13-127 | Y34_scaffold00254-15 | P131_scaffold00956-4 | short-chain dehydrogenase |
| supercontig_6.13-128 | Y34_scaffold00254-16 | P131_scaffold00956-3 | RNA polymerase II mediator complex component Srb8 |
| supercontig_6.13-140 | Y34_scaffold00074-5 | P131_scaffold00117-20 | monoxygenase |
| supercontig_6.13-158 | Y34_scaffold00097-14 | P131_scaffold00117-2 | hypothetical protein |
| supercontig_6.13-167 | Y34_scaffold00775-7 | P131_scaffold01271-7 | subtilisin-like serine protease |
| supercontig_6.13-184 | Y34_scaffold00519-24 | P131_scaffold00962-3 | eukaryotic translation initiation factor 2C |
| supercontig_6.13-185 | Y34_scaffold00519-23 | P131_scaffold00962-4 | hypothetical protein |
| supercontig_6.13-189 | Y34_scaffold00519-19 | P131_scaffold01177-2 | hypothetical protein |
| supercontig_6.13-205 | Y34_scaffold00519-4 | P131_scaffold01177-17 | hypothetical protein |
| supercontig_6.13-243 | Y34_scaffold00923-19 | P131_scaffold01546-2 | flavonol synthase |
| supercontig_6.13-247 | Y34_scaffold00923-15 | P131_scaffold01546-6 | CFEM domain-containing protein |
| supercontig_6.13-254 | Y34_scaffold00923-8 | P131_scaffold01546-13 | hypothetical protein |
| supercontig_6.13-259 | Y34_scaffold00923-3 | P131_scaffold01546-18 | hypothetical protein |
| supercontig_6.13-267 | Y34_scaffold00851-4 | P131_scaffold00110-2 | NAD dependent epimerase/dehydratase |
| supercontig_6.13-303 | Y34_scaffold00204-1 | P131_scaffold00281-4 | hypothetical protein |
| supercontig_6.13-325 | Y34_scaffold00946-8 | P131_scaffold00154-3 | regulator of chromosome condensation family protein |
| supercontig_6.13-336 | Y34_scaffold01175-10 | P131_scaffold01777-3 | hypothetical protein |
| supercontig_6.13-34 | Y34_scaffold01003-21 | P131_scaffold01291-20 | aerobactin siderophore biosynthesis protein iucB |
| supercontig_6.13-353 | Y34_scaffold00091-4 | P131_scaffold01131-9 | hypothetical protein |
| supercontig_6.13-356 | Y34_scaffold00091-1 | P131_scaffold01131-12 | hypothetical protein |
| supercontig_6.13-361 | Y34_scaffold00748-3 | P131_scaffold00027-17 | hypothetical protein |
| supercontig_6.13-371 | Y34_scaffold00748-13 | P131_scaffold00027-7 | GMP synthase |
| supercontig_6.13-39 | Y34_scaffold01003-16 | P131_scaffold01291-25 | hypothetical protein |
| supercontig_6.13-393 | Y34_scaffold00748-35 | P131_scaffold01189-9 | hypothetical protein |
| supercontig_6.13-404 | Y34_scaffold00363-3 | P131_scaffold00075-5 | binuclear zinc transcription factor |
| supercontig_6.13-41 | Y34_scaffold01003-14 | P131_scaffold01291-27 | negative regulator of DNA transposition (Rtt106) |
| supercontig_6.13-422 | Y34_scaffold00559-41 | P131_scaffold01171-9 | nuclear protein bimA |
| supercontig_6.13-429 | Y34_scaffold00559-34 | P131_scaffold01171-16 | phosphatidylinositol 4-kinase PIK1 |
| supercontig_6.13-459 | Y34_scaffold00559-4 | P131_scaffold00568-13 | hypothetical protein |
| supercontig_6.13-479 | Y34_scaffold00095-39 | P131_scaffold01365-6 | hypothetical protein |
| supercontig_6.13-493 | Y34_scaffold00095-25 | P131_scaffold00151-8 | C6 transcription factor |
| supercontig_6.13-498 | Y34_scaffold00095-20 | P131_scaffold00151-13 | actin binding protein |
| supercontig_6.13-505 | Y34_scaffold00095-14 | P131_scaffold00505-7 | adhesin protein Mad1 |
| supercontig_6.13-525 | Y34_scaffold00088-7 | P131_scaffold00126-15 | no_hit |
| supercontig_6.13-534 | Y34_scaffold00088-16 | P131_scaffold00126-6 | coronin |
| supercontig_6.13-541 | Y34_scaffold00088-23 | P131_scaffold00442-2 | guanylate kinase |
| supercontig_6.13-543 | Y34_scaffold00088-25 | P131_scaffold00442-4 | eukaryotic initiation factor 4F subunit p130 |
| supercontig_6.13-547 | Y34_scaffold00088-29 | P131_scaffold00402-2 | protein kinase Yak1 |
| supercontig_6.13-558 | Y34_scaffold00088-40 | P131_scaffold00402-13 | dcp2 |
| supercontig_6.13-566 | Y34_scaffold00088-48 | P131_scaffold00097-2 | peptidyl-prolyl cis-trans isomerase-like 2 |
| supercontig_6.13-570 | Y34_scaffold00088-52 | P131_scaffold00097-6 | calcium/calmodulin-dependent protein kinase |
| supercontig_6.13-58 | Y34_scaffold00705-4 | P131_scaffold01297-6 | MFS transporter |
| supercontig_6.13-608 | Y34_scaffold00247-25 | P131_scaffold01381-48 | hypothetical protein |
| supercontig_6.13-624 | Y34_scaffold00247-41 | P131_scaffold01381-32 | potassium transporter |
| supercontig_6.13-652 | Y34_scaffold00247-69 | P131_scaffold01381-5 | transcription factor TamA |
| supercontig_6.13-658 | Y34_scaffold00683-3 | P131_scaffold00415-2 | RNA binding effector protein Scp160 |
| supercontig_6.13-665 | Y34_scaffold00683-10 | P131_scaffold00415-9 | no_hit |
| supercontig_6.13-668 | Y34_scaffold00683-13 | P131_scaffold00415-12 | mRNA cap guanine-N7 methyltransferase |
| supercontig_6.13-689 | Y34_scaffold00037-55 | P131_scaffold00082-10 | histidine acid phosphatase family protein |
| supercontig_6.13-695 | Y34_scaffold00037-49 | P131_scaffold00082-17 | hypothetical protein |
| supercontig_6.13-743 | Y34_scaffold00037-3 | P131_scaffold00753-3 | cAMP-independent regulatory protein pac2 |
| supercontig_6.13-761 | Y34_scaffold00182-13 | P131_scaffold00539-15 | hypothetical protein |
| supercontig_6.13-775 | Y34_scaffold00693-1 | P131_scaffold00937-3 | putative secreted glycosylase |
| supercontig_6.13-785 | Y34_scaffold00693-11 | P131_scaffold00229-8 | pfs domain protein |
| supercontig_6.13-798 | Y34_scaffold00224-3 | P131_scaffold01296-36 | putative lipase |
| supercontig_6.13-800 | Y34_scaffold00224-5 | P131_scaffold01296-34 | hypothetical protein |
| supercontig_6.13-810 | Y34_scaffold00224-15 | P131_scaffold01296-24 | hypothetical protein |
| supercontig_6.13-818 | Y34_scaffold00224-23 | P131_scaffold01296-16 | hypothetical protein |
| supercontig_6.13-848 | Y34_scaffold00262-21 | P131_scaffold00626-15 | hypothetical protein |
| supercontig_6.13-849 | Y34_scaffold00262-20 | P131_scaffold00626-16 | hypothetical protein |
| supercontig_6.13-855 | Y34_scaffold00262-14 | P131_scaffold00626-22 | leucine carboxyl methyltransferase 2 |
| supercontig_6.13-857 | Y34_scaffold00262-12 | P131_scaffold00626-24 | hypothetical protein |
| supercontig_6.13-879 | Y34_scaffold00155-38 | P131_scaffold01199-10 | YdiU domain-containing protein |
| supercontig_6.13-882 | Y34_scaffold00155-35 | P131_scaffold01199-13 | related to cytokinesis inhibitor byr4 |
| supercontig_6.13-895 | Y34_scaffold00155-22 | P131_scaffold01199-26 | hypothetical protein |
| supercontig_6.13-905 | Y34_scaffold00155-12 | P131_scaffold01199-36 | C6 transcription factor |
| supercontig_6.13-911 | Y34_scaffold00155-6 | P131_scaffold01199-42 | hypothetical protein |
| supercontig_6.13-912 | Y34_scaffold00155-5 | P131_scaffold01199-43 | spastin |
| supercontig_6.13-953 | Y34_scaffold00765-37 | P131_scaffold00168-6 | hypothetical protein |
| supercontig_6.13-970 | Y34_scaffold00765-54 | P131_scaffold01311-11 | hypothetical protein |
| supercontig_6.13-977 | Y34_scaffold00765-61 | P131_scaffold01311-18 | related to heat shock transcription factor |
| supercontig_6.13-979 | Y34_scaffold00765-63 | P131_scaffold01311-20 | vacuolar ATP synthase subunit B |
| supercontig_6.13-984 | Y34_scaffold00765-68 | P131_scaffold01311-25 | ARS-binding protein |
| supercontig_6.13-990 | Y34_scaffold00765-74 | P131_scaffold01311-31 | TBC domain protein |
| supercontig_6.14-10 | Y34_scaffold00021-10 | P131_scaffold00045-30 | malate dehydrogenase |
| supercontig_6.14-28 | Y34_scaffold00021-28 | P131_scaffold00045-13 | alcohol dehydrogenase |
| supercontig_6.14-52 | Y34_scaffold00477-12 | P131_scaffold00754-5 | FYVE zinc finger domain containing protein |
| supercontig_6.15-100 | Y34_scaffold00602-3 | P131_scaffold00014-3 | hypothetical protein |
| supercontig_6.15-102 | Y34_scaffold00602-2 | P131_scaffold00014-2 | MFS transporter |
| supercontig_6.15-135 | Y34_scaffold00467-7 | P131_scaffold00366-3 | lysocardiolipin acyltransferase |
| supercontig_6.15-146 | Y34_scaffold00494-1 | P131_scaffold00964-1 | hypothetical protein |
| supercontig_6.15-155 | Y34_scaffold00771-9 | P131_scaffold01321-9 | hypothetical protein |
| supercontig_6.15-196 | Y34_scaffold00277-10 | P131_scaffold01004-4 | hypothetical protein |
| supercontig_6.15-202 | Y34_scaffold00277-4 | P131_scaffold01004-10 | hypothetical protein |
| supercontig_6.15-218 | Y34_scaffold00482-1 | P131_scaffold00987-1 | actin |
| supercontig_6.15-243 | Y34_scaffold00624-87 | P131_scaffold00440-14 | hypothetical protein |
| supercontig_6.15-266 | Y34_scaffold00624-63 | P131_scaffold00130-7 | hypothetical protein |
| supercontig_6.15-29 | Y34_scaffold00249-5 | P131_scaffold00073-5 | hypothetical protein |
| supercontig_6.15-304 | Y34_scaffold00624-27 | P131_scaffold01379-6 | hypothetical protein |
| supercontig_6.15-305 | Y34_scaffold00624-26 | P131_scaffold01379-5 | putative Zn(II)2Cys6 transcription factor |
| supercontig_6.15-314 | Y34_scaffold00624-17 | P131_scaffold01194-5 | carboxypeptidase Y precursor |
| supercontig_6.15-329 | Y34_scaffold00624-3 | P131_scaffold01194-20 | hypothetical protein |
| supercontig_6.15-336 | Y34_scaffold00576-27 | P131_scaffold00294-18 | potassium channel regulatory factor |
| supercontig_6.15-339 | Y34_scaffold00576-24 | P131_scaffold00294-15 | no_hit |
| supercontig_6.15-340 | Y34_scaffold00576-23 | P131_scaffold00294-14 | C6 transcription factor |
| supercontig_6.15-357 | Y34_scaffold00576-6 | P131_scaffold01338-94 | DUF1339 domain protein |
| supercontig_6.15-358 | Y34_scaffold00576-5 | P131_scaffold01338-93 | ATP-dependent helicase SGS1 |
| supercontig_6.15-361 | Y34_scaffold00576-2 | P131_scaffold01338-90 | hypothetical protein |
| supercontig_6.15-369 | Y34_scaffold00567-5 | P131_scaffold01338-83 | HLH transcription factor |
| supercontig_6.15-408 | Y34_scaffold00567-42 | P131_scaffold01338-45 | oxidoreductase |
| supercontig_6.15-409 | Y34_scaffold00567-43 | P131_scaffold01338-44 | nuclear pore complex protein Nup107 |
| supercontig_6.15-416 | Y34_scaffold00567-50 | P131_scaffold01338-36 | hypothetical protein |
| supercontig_6.15-452 | Y34_scaffold01012-1 | P131_scaffold01635-1 | hypothetical protein |
| supercontig_6.15-476 | Y34_scaffold00498-16 | P131_scaffold01697-16 | metalloreductase |
| supercontig_6.15-518 | Y34_scaffold00651-17 | P131_scaffold00557-7 | BAR domain-containing protein |
| supercontig_6.15-564 | Y34_scaffold00589-21 | P131_scaffold00074-22 | kinesin |
| supercontig_6.15-565 | Y34_scaffold00589-22 | P131_scaffold01111-1 | hypothetical protein |
| supercontig_6.15-574 | Y34_scaffold00589-31 | P131_scaffold00083-5 | hypothetical protein |
| supercontig_6.15-578 | Y34_scaffold00589-35 | P131_scaffold00083-9 | no_hit |
| supercontig_6.15-586 | Y34_scaffold00790-25 | P131_scaffold00083-17 | glutathione transferase omega-1 |
| supercontig_6.15-609 | Y34_scaffold00790-1 | P131_scaffold00083-41 | hypothetical protein |
| supercontig_6.15-61 | Y34_scaffold00702-1 | P131_scaffold00191-6 | no_hit |
| supercontig_6.15-631 | Y34_scaffold00445-5 | P131_scaffold00295-5 | putative xylanase 27 |
| supercontig_6.15-636 | Y34_scaffold00445-10 | P131_scaffold00295-10 | beta-Ig-H3/Fasciclin |
| supercontig_6.15-71 | Y34_scaffold00082-5 | P131_scaffold01062-1 | hypothetical protein |
| supercontig_6.15-89 | Y34_scaffold00141-7 | P131_scaffold00524-5 | hypothetical protein |
| supercontig_6.16-106 | Y34_scaffold01005-18 | P131_scaffold01138-58 | hypothetical protein |
| supercontig_6.16-114 | Y34_scaffold01005-8 | P131_scaffold01138-66 | hypothetical protein |
| supercontig_6.16-129 | Y34_scaffold00500-3 | P131_scaffold00377-3 | hypothetical protein |
| supercontig_6.16-131 | Y34_scaffold00500-5 | P131_scaffold00377-5 | high affinity nitrate transporter NrtB |
| supercontig_6.16-147 | Y34_scaffold00500-21 | P131_scaffold00377-20 | hypothetical protein |
| supercontig_6.16-155 | Y34_scaffold00500-28 | P131_scaffold01211-4 | hypothetical protein |
| supercontig_6.16-162 | Y34_scaffold00500-35 | P131_scaffold01211-11 | hypothetical protein |
| supercontig_6.16-164 | Y34_scaffold00500-37 | P131_scaffold01211-13 | hypothetical protein |
| supercontig_6.16-175 | Y34_scaffold00500-48 | P131_scaffold01211-24 | cytochrome P450 phenylacetate 2-hydroxylase |
| supercontig_6.16-183 | Y34_scaffold00500-55 | P131_scaffold01211-32 | zinc knuckle domain containing protein |
| supercontig_6.16-190 | Y34_scaffold00500-62 | P131_scaffold01211-39 | pre-mRNA-splicing factor ATP-dependent RNA helicase PRP22 |
| supercontig_6.16-196 | Y34_scaffold00751-6 | P131_scaffold01370-5 | hypothetical protein |
| supercontig_6.16-197 | Y34_scaffold00751-7 | P131_scaffold01370-6 | hypothetical protein |
| supercontig_6.16-200 | Y34_scaffold00751-10 | P131_scaffold01370-9 | hypothetical protein |
| supercontig_6.16-207 | Y34_scaffold00751-17 | P131_scaffold01370-16 | bromodomain associated domain-containing protein |
| supercontig_6.16-247 | Y34_scaffold00214-19 | P131_scaffold00435-19 | hypothetical protein |
| supercontig_6.16-268 | Y34_scaffold00009-1 | P131_scaffold00565-1 | hypothetical protein |
| supercontig_6.16-38 | Y34_scaffold01005-90 | P131_scaffold01147-10 | hypothetical protein |
| supercontig_6.16-51 | Y34_scaffold01005-77 | P131_scaffold01138-4 | solute carrier family 25 member 42 |
| supercontig_6.16-70 | Y34_scaffold01005-56 | P131_scaffold01138-22 | hypothetical protein |
| supercontig_6.16-88 | Y34_scaffold01005-37 | P131_scaffold01138-40 | SacI domain and endonuclease/exonuclease/phosphatase |
| supercontig_6.16-9 | Y34_scaffold00058-12 | P131_scaffold01159-29 | valyl-tRNA synthetase |
| supercontig_6.17-24 | Y34_scaffold00871-7 | P131_scaffold00111-7 | xenobiotic compound monooxygenase |
| supercontig_6.18-1017 | Y34_scaffold00164-4 | P131_scaffold01164-3 | extensin |
| supercontig_6.18-1041 | Y34_scaffold00556-2 | P131_scaffold01625-2 | hypothetical protein |
| supercontig_6.18-1043 | Y34_scaffold00556-4 | P131_scaffold01625-4 | hypothetical protein |
| supercontig_6.18-1044 | Y34_scaffold00556-5 | P131_scaffold01625-5 | efflux pump antibiotic resistance protein |
| supercontig_6.18-1045 | Y34_scaffold00556-6 | P131_scaffold01625-6 | hypothetical protein |
| supercontig_6.18-1101 | Y34_scaffold00580-25 | P131_scaffold00940-2 | short-chain dehydrogenase/reductase |
| supercontig_6.18-1147 | Y34_scaffold00216-9 | P131_scaffold00166-9 | hypothetical protein |
| supercontig_6.18-125 | Y34_scaffold00283-26 | P131_scaffold00417-3 | hypothetical protein |
| supercontig_6.18-1285 | Y34_scaffold00767-4 | P131_scaffold01155-4 | protein kinase domain containing protein |
| supercontig_6.18-130 | Y34_scaffold00283-31 | P131_scaffold00417-8 | hypothetical protein |
| supercontig_6.18-1337 | Y34_scaffold00812-5 | P131_scaffold00990-11 | hypothetical protein |
| supercontig_6.18-134 | Y34_scaffold00283-35 | P131_scaffold00417-12 | hypothetical protein |
| supercontig_6.18-1341 | Y34_scaffold00812-1 | P131_scaffold00990-7 | hypothetical protein |
| supercontig_6.18-1343 | Y34_scaffold00945-11 | P131_scaffold00990-5 | hypothetical protein |
| supercontig_6.18-1364 | Y34_scaffold00491-11 | P131_scaffold00339-3 | hypothetical protein |
| supercontig_6.18-1389 | Y34_scaffold00414-45 | P131_scaffold01069-32 | similar to novel radial spoke domain containing protein |
| supercontig_6.18-1405 | Y34_scaffold00414-29 | P131_scaffold01069-16 | arginine N-methyltransferase 2 |
| supercontig_6.18-1429 | Y34_scaffold00414-5 | P131_scaffold01083-9 | hypothetical protein |
| supercontig_6.18-156 | Y34_scaffold00283-57 | P131_scaffold00382-6 | acetate permease |
| supercontig_6.18-172 | Y34_scaffold00283-73 | P131_scaffold00382-22 | FAD binding domain protein |
| supercontig_6.18-175 | Y34_scaffold00283-76 | P131_scaffold01182-1 | phosphatase DCR2 |
| supercontig_6.18-198 | Y34_scaffold00283-99 | P131_scaffold00255-39 | glucose/galactose transporter |
| supercontig_6.18-205 | Y34_scaffold00283-106 | P131_scaffold00255-32 | related to regulator of deoxyribodipyrimidine photo-lyase PHR1 |
| supercontig_6.18-232 | Y34_scaffold00649-20 | P131_scaffold00255-6 | helicase SWR1 |
| supercontig_6.18-241 | Y34_scaffold00649-11 | P131_scaffold00380-4 | 40S ribosomal protein S0 |
| supercontig_6.18-245 | Y34_scaffold00649-7 | P131_scaffold00380-8 | related to protein kinase PAK1 |
| supercontig_6.18-260 | Y34_scaffold00663-8 | P131_scaffold00443-6 | hypothetical protein |
| supercontig_6.18-27 | Y34_scaffold00215-5 | P131_scaffold00889-4 | histone acetyltransferase GCN5 |
| supercontig_6.18-272 | Y34_scaffold00276-8 | P131_scaffold00448-2 | probable carnitine acetyl transferase FacC |
| supercontig_6.18-290 | Y34_scaffold00684-27 | P131_scaffold00199-16 | hypothetical protein |
| supercontig_6.18-3 | Y34_scaffold00106-3 | P131_scaffold00174-2 | WD repeat-containing protein |
| supercontig_6.18-338 | Y34_scaffold00140-12 | P131_scaffold00328-13 | probable myosin MYO2 |
| supercontig_6.18-358 | Y34_scaffold00140-32 | P131_scaffold00328-33 | related to sec7-domain protein |
| supercontig_6.18-36 | Y34_scaffold00172-6 | P131_scaffold00481-14 | serine/threonine-protein phosphatase 2A activator 2 |
| supercontig_6.18-390 | Y34_scaffold00140-63 | P131_scaffold01268-17 | ATP-dependent permease MDL2 |
| supercontig_6.18-411 | Y34_scaffold00140-83 | P131_scaffold01035-12 | pyruvate kinase |
| supercontig_6.18-439 | Y34_scaffold00140-111 | P131_scaffold01187-5 | chromosome-associated kinesin KIF4 |
| supercontig_6.18-445 | Y34_scaffold00140-117 | P131_scaffold01187-12 | aldo-keto reductase family 1 member E1 |
| supercontig_6.18-446 | Y34_scaffold00140-118 | P131_scaffold01187-13 | hypothetical protein |
| supercontig_6.18-48 | Y34_scaffold00145-8 | P131_scaffold00481-2 | mediator of RNA polymerase II transcription subunit 14 |
| supercontig_6.18-522 | Y34_scaffold00686-3 | P131_scaffold01308-2 | DENN domain-containing protein |
| supercontig_6.18-528 | Y34_scaffold00686-9 | P131_scaffold00408-9 | HIT finger domain protein |
| supercontig_6.18-54 | Y34_scaffold00145-14 | P131_scaffold01201-6 | aromatic amino acid aminotransferase |
| supercontig_6.18-546 | Y34_scaffold00151-8 | P131_scaffold00135-8 | phosphatidate cytidylyltransferase family protein |
| supercontig_6.18-562 | Y34_scaffold00512-2 | P131_scaffold01116-2 | 6-O-methylguanine DNA methyltransferase |
| supercontig_6.18-585 | Y34_scaffold00289-3 | P131_scaffold00400-40 | TFIIIC transcription initiation factor complex subunits Tfc3 |
| supercontig_6.18-588 | Y34_scaffold00725-1 | P131_scaffold00400-37 | hypothetical protein |
| supercontig_6.18-604 | Y34_scaffold00725-17 | P131_scaffold00400-22 | small nuclear ribonucleoprotein |
| supercontig_6.18-618 | Y34_scaffold00725-31 | P131_scaffold00400-8 | 3'-5' exoribonuclease |
| supercontig_6.18-619 | Y34_scaffold00725-32 | P131_scaffold00400-7 | SH3 domain protein |
| supercontig_6.18-620 | Y34_scaffold00725-33 | P131_scaffold00400-6 | tubulin-tyrosine ligase |
| supercontig_6.18-624 | Y34_scaffold00725-37 | P131_scaffold00400-2 | hypothetical protein |
| supercontig_6.18-650 | Y34_scaffold00712-13 | P131_scaffold00546-13 | CHY zinc finger domain-containing protein |
| supercontig_6.18-694 | Y34_scaffold00533-3 | P131_scaffold00343-32 | related to two-component histidine kinase chk-1 |
| supercontig_6.18-701 | Y34_scaffold00533-10 | P131_scaffold00343-39 | pantothenate transporter liz1 |
| supercontig_6.18-706 | Y34_scaffold00533-15 | P131_scaffold00343-44 | phosphatase |
| supercontig_6.18-72 | Y34_scaffold00648-27 | P131_scaffold01201-24 | DNA binding protein |
| supercontig_6.18-771 | Y34_scaffold00836-12 | P131_scaffold00949-2 | related to lustrin A |
| supercontig_6.18-773 | Y34_scaffold00534-1 | P131_scaffold00177-1 | cobalamin-independent synthase |
| supercontig_6.18-780 | Y34_scaffold00534-8 | P131_scaffold00177-8 | ATP-citrate synthase subunit 1 |
| supercontig_6.18-789 | Y34_scaffold00534-17 | P131_scaffold00177-17 | related to GTP-binding protein beta subunit-like protein |
| supercontig_6.18-793 | Y34_scaffold00534-21 | P131_scaffold00177-21 | hypothetical protein |
| supercontig_6.18-796 | Y34_scaffold00534-24 | P131_scaffold00177-24 | hypothetical protein |
| supercontig_6.18-811 | Y34_scaffold00534-39 | P131_scaffold00177-39 | glutathione S-transferase |
| supercontig_6.18-812 | Y34_scaffold00534-40 | P131_scaffold00177-40 | zinc knuckle domain containing protein |
| supercontig_6.18-814 | Y34_scaffold00534-42 | P131_scaffold00177-42 | hypothetical protein |
| supercontig_6.18-819 | Y34_scaffold00534-47 | P131_scaffold00177-47 | CFEM domain-containing protein |
| supercontig_6.18-827 | Y34_scaffold00534-55 | P131_scaffold00177-55 | FAD binding domain protein |
| supercontig_6.18-832 | Y34_scaffold00534-60 | P131_scaffold00177-60 | DnaJ domain containing protein |
| supercontig_6.18-846 | Y34_scaffold00534-74 | P131_scaffold00350-14 | putative DNA helicase INO80 0.0 |
| supercontig_6.18-857 | Y34_scaffold00534-85 | P131_scaffold01049-9 | exosome complex exonuclease RRP41 |
| supercontig_6.18-902 | Y34_scaffold00035-2 | P131_scaffold00939-5 | hypothetical protein |
| supercontig_6.18-914 | Y34_scaffold00291-5 | P131_scaffold01098-7 | short chain dehydrogenase |
| supercontig_6.18-923 | Y34_scaffold00779-4 | P131_scaffold01668-5 | hypothetical protein |
| supercontig_6.18-951 | Y34_scaffold00047-4 | P131_scaffold00364-10 | kinase-related protein |
| supercontig_6.18-952 | Y34_scaffold00047-3 | P131_scaffold00364-11 | base excision DNA repair protein |
| supercontig_6.18-96 | Y34_scaffold00648-3 | P131_scaffold01201-48 | hypothetical protein |
| supercontig_6.18-979 | Y34_scaffold00045-4 | P131_scaffold00012-4 | hypothetical protein |
| supercontig_6.18-997 | Y34_scaffold00626-3 | P131_scaffold00297-3 | hypothetical protein |
| supercontig_6.18-998 | Y34_scaffold00626-2 | P131_scaffold00297-4 | FAD binding domain containing protein |
| supercontig_6.19-5 | Y34_scaffold00965-2 | P131_scaffold01693-1 | cytochrome P450 |
| supercontig_6.19-63 | Y34_scaffold00213-6 | P131_scaffold01128-8 | ATP-dependent RNA helicase MSS116 |
| supercontig_6.19-69 | Y34_scaffold00213-12 | P131_scaffold01128-14 | arylsulfatase |
| supercontig_6.20-135 | Y34_scaffold01081-2 | P131_scaffold01585-2 | hypothetical protein |
| supercontig_6.20-175 | Y34_scaffold00650-4 | P131_scaffold01140-4 | tyrosinase |
| supercontig_6.20-181 | Y34_scaffold00552-126 | P131_scaffold01326-4 | hypothetical protein |
| supercontig_6.20-182 | Y34_scaffold00552-125 | P131_scaffold01326-5 | hypothetical protein |
| supercontig_6.20-206 | Y34_scaffold00552-101 | P131_scaffold00115-1 | related to anaphase control protein cut9 |
| supercontig_6.20-232 | Y34_scaffold00552-76 | P131_scaffold00472-9 | hypothetical protein |
| supercontig_6.20-250 | Y34_scaffold00552-58 | P131_scaffold01198-10 | tetratricopeptide repeat protein |
| supercontig_6.20-255 | Y34_scaffold00552-53 | P131_scaffold01198-15 | amino acid transporter (predicted) |
| supercontig_6.20-264 | Y34_scaffold00552-44 | P131_scaffold01198-24 | CDF manganese transporter |
| supercontig_6.20-290 | Y34_scaffold00552-18 | P131_scaffold01198-50 | hypothetical protein |
| supercontig_6.20-296 | Y34_scaffold00552-12 | P131_scaffold01198-56 | related to exo-alpha-sialidase / neuraminidase |
| supercontig_6.20-306 | Y34_scaffold00552-2 | P131_scaffold01198-66 | 75k gamma secalin |
| supercontig_6.20-307 | Y34_scaffold00552-1 | P131_scaffold01198-67 | hypothetical protein 0.0 |
| supercontig_6.20-329 | Y34_scaffold00736-8 | P131_scaffold00121-3 | CFEM domain-containing protein |
| supercontig_6.20-335 | Y34_scaffold00736-4 | P131_scaffold00121-7 | hypothetical protein |
| supercontig_6.20-369 | Y34_scaffold00449-1 | P131_scaffold00143-3 | tyrosinase |
| supercontig_6.20-418 | Y34_scaffold00719-27 | P131_scaffold01139-18 | hypothetical protein |
| supercontig_6.20-424 | Y34_scaffold00719-21 | P131_scaffold01139-12 | related to benzoylformate decarboxylase |
| supercontig_6.20-425 | Y34_scaffold00719-20 | P131_scaffold01139-11 | flavin-binding monooxygenase-like protein |
| supercontig_6.20-439 | Y34_scaffold00719-7 | P131_scaffold00854-11 | no_hit |
| supercontig_6.20-444 | Y34_scaffold00719-2 | P131_scaffold00854-6 | peptidase M14 |
| supercontig_6.20-458 | Y34_scaffold00012-2 | P131_scaffold01549-2 | lipase/esterase |
| supercontig_6.20-460 | Y34_scaffold00012-4 | P131_scaffold01549-4 | class II aldolase/adducin domain protein |
| supercontig_6.20-462 | Y34_scaffold00012-6 | P131_scaffold01549-6 | proline iminopeptidase |
| supercontig_6.20-468 | Y34_scaffold00613-5 | P131_scaffold01103-5 | carbohydrate-binding module family 21 |
| supercontig_6.20-482 | Y34_scaffold00480-12 | P131_scaffold01103-19 | hypothetical protein |
| supercontig_6.20-497 | Y34_scaffold00926-16 | P131_scaffold01555-7 | hypothetical protein |
| supercontig_6.20-507 | Y34_scaffold00926-6 | P131_scaffold01555-17 | protein kinase |
| supercontig_6.20-510 | Y34_scaffold00926-3 | P131_scaffold01555-20 | maltose permease |
| supercontig_6.20-512 | Y34_scaffold00926-1 | P131_scaffold00387-2 | hypothetical protein |
| supercontig_6.20-57 | Y34_scaffold00274-2 | P131_scaffold01290-2 | MFS transporter |
| supercontig_6.20-65 | Y34_scaffold00893-5 | P131_scaffold01367-3 | hypothetical protein |
| supercontig_6.20-93 | Y34_scaffold00791-3 | P131_scaffold01169-3 | RTA1 domain protein |
| supercontig_6.21-1007 | Y34_scaffold00608-49 | P131_scaffold00220-78 | fungal specific transcription factor domain-containing protein |
| supercontig_6.21-1011 | Y34_scaffold00608-53 | P131_scaffold00534-1 | sterol esterase precursor |
| supercontig_6.21-1029 | Y34_scaffold00608-70 | P131_scaffold01455-9 | cytochrome P450 3A17 |
| supercontig_6.21-1030 | Y34_scaffold00608-71 | P131_scaffold01455-8 | probable cytochrome P450 monooxygenase |
| supercontig_6.21-1033 | Y34_scaffold00608-74 | P131_scaffold01455-5 | hypothetical protein |
| supercontig_6.21-1037 | Y34_scaffold00608-78 | P131_scaffold01455-1 | hypothetical protein |
| supercontig_6.21-1042 | Y34_scaffold00608-83 | P131_scaffold00325-2 | hypothetical protein |
| supercontig_6.21-1044 | Y34_scaffold00608-85 | P131_scaffold00462-1 | laccase |
| supercontig_6.21-1045 | Y34_scaffold00608-86 | P131_scaffold00462-2 | G-patch domain-containing protein |
| supercontig_6.21-1052 | Y34_scaffold00608-93 | P131_scaffold00185-2 | hypothetical protein |
| supercontig_6.21-1063 | Y34_scaffold00190-3 | P131_scaffold01375-3 | hypothetical protein |
| supercontig_6.21-1068 | Y34_scaffold00672-3 | P131_scaffold01087-3 | hypothetical protein |
| supercontig_6.21-1074 | Y34_scaffold00672-9 | P131_scaffold01087-9 | glycoside hydrolase family 55 |
| supercontig_6.21-1082 | Y34_scaffold00672-16 | P131_scaffold01087-16 | cytosolic non-specific dipeptidase |
| supercontig_6.21-1105 | Y34_scaffold00931-2 | P131_scaffold01294-3 | polyketide synthase |
| supercontig_6.21-1141 | Y34_scaffold00793-7 | P131_scaffold00214-35 | hypothetical protein |
| supercontig_6.21-1148 | Y34_scaffold00793-14 | P131_scaffold00214-28 | hypothetical protein |
| supercontig_6.21-1210 | Y34_scaffold00267-6 | P131_scaffold00525-6 | hypothetical protein |
| supercontig_6.21-122 | Y34_scaffold00496-39 | P131_scaffold01325-46 | hypothetical protein |
| supercontig_6.21-1247 | Y34_scaffold00206-10 | P131_scaffold00034-43 | siderochrome-iron transporter |
| supercontig_6.21-126 | Y34_scaffold00496-36 | P131_scaffold01325-49 | rab guanyl-nucleotide exchange factor |
| supercontig_6.21-1265 | Y34_scaffold00119-10 | P131_scaffold00034-25 | GTPase-activating protein gyp3 |
| supercontig_6.21-1268 | Y34_scaffold00119-13 | P131_scaffold00034-22 | hypothetical protein |
| supercontig_6.21-136 | Y34_scaffold00496-26 | P131_scaffold01325-59 | arrestin domain-containing protein |
| supercontig_6.21-177 | Y34_scaffold00462-59 | P131_scaffold01132-17 | hypothetical protein |
| supercontig_6.21-18 | Y34_scaffold00697-57 | P131_scaffold01079-6 | acyl-CoA thioesterase |
| supercontig_6.21-180 | Y34_scaffold00462-56 | P131_scaffold01132-14 | hypothetical protein |
| supercontig_6.21-189 | Y34_scaffold00462-47 | P131_scaffold01132-5 | hypothetical protein |
| supercontig_6.21-196 | Y34_scaffold00462-40 | P131_scaffold00532-3 | hypothetical protein |
| supercontig_6.21-200 | Y34_scaffold00462-36 | P131_scaffold00532-7 | intermediate filament |
| supercontig_6.21-205 | Y34_scaffold00462-31 | P131_scaffold00532-12 | RNA binding protein Rnp24 |
| supercontig_6.21-209 | Y34_scaffold00462-27 | P131_scaffold00114-1 | aspartyl proteinase |
| supercontig_6.21-212 | Y34_scaffold00462-24 | P131_scaffold00114-4 | syntaxin 2 |
| supercontig_6.21-219 | Y34_scaffold00462-17 | P131_scaffold00114-12 | hypothetical protein |
| supercontig_6.21-223 | Y34_scaffold00462-13 | P131_scaffold00114-16 | mitochondrial porin |
| supercontig_6.21-228 | Y34_scaffold00462-8 | P131_scaffold00114-21 | hypothetical protein |
| supercontig_6.21-238 | Y34_scaffold00832-18 | P131_scaffold01094-19 | oxidoreductase domain containing protein |
| supercontig_6.21-249 | Y34_scaffold00832-8 | P131_scaffold01094-9 | related to phosphoethanolamine cytidylyltransferase |
| supercontig_6.21-264 | Y34_scaffold00126-123 | P131_scaffold00345-91 | sulfite reductase flavoprotein component |
| supercontig_6.21-265 | Y34_scaffold00126-122 | P131_scaffold00345-90 | sec7 domain-containing protein |
| supercontig_6.21-275 | Y34_scaffold00126-112 | P131_scaffold00345-80 | related to transcription regulator SPT7 |
| supercontig_6.21-277 | Y34_scaffold00126-110 | P131_scaffold00345-78 | vacuolar protease A-like protein |
| supercontig_6.21-280 | Y34_scaffold00126-107 | P131_scaffold00345-75 | GMP synthase |
| supercontig_6.21-281 | Y34_scaffold00126-106 | P131_scaffold00345-74 | hypothetical protein |
| supercontig_6.21-301 | Y34_scaffold00126-86 | P131_scaffold00345-55 | related to tubulin folding cofactor C |
| supercontig_6.21-308 | Y34_scaffold00126-80 | P131_scaffold00345-49 | Pfs |
| supercontig_6.21-313 | Y34_scaffold00126-75 | P131_scaffold00345-44 | hypothetical protein |
| supercontig_6.21-318 | Y34_scaffold00126-71 | P131_scaffold00345-40 | transcription initiation factor TFIID subunit 12 |
| supercontig_6.21-321 | Y34_scaffold00126-68 | P131_scaffold00345-37 | hypothetical protein |
| supercontig_6.21-323 | Y34_scaffold00126-66 | P131_scaffold00345-35 | Bck1-like MAP kinase kinase kinase,MCK1 |
| supercontig_6.21-331 | Y34_scaffold00126-58 | P131_scaffold00345-27 | ubiquitin carboxyl-terminal hydrolase |
| supercontig_6.21-334 | Y34_scaffold00126-55 | P131_scaffold00345-24 | hypothetical protein |
| supercontig_6.21-340 | Y34_scaffold00126-49 | P131_scaffold00345-18 | predicted protein |
| supercontig_6.21-348 | Y34_scaffold00126-41 | P131_scaffold00345-10 | oligopeptide transporter |
| supercontig_6.21-355 | Y34_scaffold00126-34 | P131_scaffold00345-3 | hypothetical protein |
| supercontig_6.21-364 | Y34_scaffold00126-25 | P131_scaffold00123-7 | LipA and NB-ARC domain-containing protein |
| supercontig_6.21-375 | Y34_scaffold00126-14 | P131_scaffold00123-18 | hypothetical protein |
| supercontig_6.21-379 | Y34_scaffold00126-10 | P131_scaffold00123-22 | related to alpha-ribazole-5'-phosphate phosphatase |
| supercontig_6.21-383 | Y34_scaffold00126-6 | P131_scaffold00123-26 | flotillin domain-containing protein |
| supercontig_6.21-39 | Y34_scaffold00697-37 | P131_scaffold00304-7 | hypothetical protein |
| supercontig_6.21-406 | Y34_scaffold01007-8 | P131_scaffold00602-8 | hypothetical protein |
| supercontig_6.21-416 | Y34_scaffold01007-18 | P131_scaffold00602-18 | polyketide synthase |
| supercontig_6.21-419 | Y34_scaffold01007-21 | P131_scaffold00602-21 | probable serine/threonine protein kinase MoSNF1 |
| supercontig_6.21-423 | Y34_scaffold00574-6 | P131_scaffold00602-26 | peptide-N4-(N-acetyl-beta-glucosaminyl)asparagine amidase A |
| supercontig_6.21-433 | Y34_scaffold00153-21 | P131_scaffold00602-36 | hypothetical protein |
| supercontig_6.21-435 | Y34_scaffold00153-19 | P131_scaffold01307-2 | eukaryotic translation initiation factor 2 beta subunit |
| supercontig_6.21-447 | Y34_scaffold00153-7 | P131_scaffold01307-14 | dual specificity mitogen-activated protein kinase kinase 1 |
| supercontig_6.21-449 | Y34_scaffold00153-5 | P131_scaffold01307-16 | choline dehydrogenase |
| supercontig_6.21-460 | Y34_scaffold00183-12 | P131_scaffold01307-27 | related to 5-oxoprolinase |
| supercontig_6.21-471 | Y34_scaffold00183-1 | P131_scaffold01307-38 | hypothetical protein |
| supercontig_6.21-473 | Y34_scaffold00669-98 | P131_scaffold01307-40 | hypothetical protein |
| supercontig_6.21-486 | Y34_scaffold00669-83 | P131_scaffold01068-19 | hypothetical protein |
| supercontig_6.21-490 | Y34_scaffold00669-79 | P131_scaffold01068-15 | palmitoyltransferase PFA4 |
| supercontig_6.21-498 | Y34_scaffold00669-71 | P131_scaffold01068-7 | cleavage factor two protein 1 |
| supercontig_6.21-5 | Y34_scaffold00359-5 | P131_scaffold00746-5 | related to U4/U6 small nuclear ribonucleoprotein hPrp3 |
| supercontig_6.21-506 | Y34_scaffold00669-63 | P131_scaffold00474-5 | hypothetical protein |
| supercontig_6.21-525 | Y34_scaffold00669-44 | P131_scaffold01213-45 | hypothetical protein |
| supercontig_6.21-535 | Y34_scaffold00669-34 | P131_scaffold01213-35 | CRAL/TRIO domain-containing protein |
| supercontig_6.21-536 | Y34_scaffold00669-33 | P131_scaffold01213-34 | related to POL12 (DNA-directed DNA polymerase alpha) |
| supercontig_6.21-540 | Y34_scaffold00669-29 | P131_scaffold01213-30 | hypothetical protein |
| supercontig_6.21-550 | Y34_scaffold00669-19 | P131_scaffold01213-20 | hypothetical protein |
| supercontig_6.21-559 | Y34_scaffold00669-10 | P131_scaffold01213-11 | hypothetical protein |
| supercontig_6.21-573 | Y34_scaffold00484-5 | P131_scaffold00318-3 | hypothetical protein |
| supercontig_6.21-586 | Y34_scaffold00099-10 | P131_scaffold00499-10 | hypothetical protein |
| supercontig_6.21-602 | Y34_scaffold00099-25 | P131_scaffold00499-25 | EF hand domain-containing protein |
| supercontig_6.21-604 | Y34_scaffold00099-27 | P131_scaffold00499-27 | salicylaldehyde dehydrogenase |
| supercontig_6.21-620 | Y34_scaffold00134-2 | P131_scaffold00267-2 | GPI mannosyltransferase 2 |
| supercontig_6.21-626 | Y34_scaffold00134-8 | P131_scaffold00267-7 | hypothetical protein |
| supercontig_6.21-641 | Y34_scaffold00528-9 | P131_scaffold00267-22 | hypothetical protein |
| supercontig_6.21-649 | Y34_scaffold00528-17 | P131_scaffold00267-30 | developmental regulator VosA |
| supercontig_6.21-663 | Y34_scaffold00528-32 | P131_scaffold00267-44 | phosphotransferase enzyme family protein |
| supercontig_6.21-674 | Y34_scaffold00528-43 | P131_scaffold00267-55 | bZIP transcription factor CpcA |
| supercontig_6.21-686 | Y34_scaffold00528-55 | P131_scaffold00267-67 | cholinesterase |
| supercontig_6.21-689 | Y34_scaffold00528-58 | P131_scaffold00267-70 | hypothetical protein |
| supercontig_6.21-702 | Y34_scaffold00528-71 | P131_scaffold00240-16 | endoglucanase |
| supercontig_6.21-703 | Y34_scaffold00528-72 | P131_scaffold00240-15 | hypothetical protein |
| supercontig_6.21-707 | Y34_scaffold00528-76 | P131_scaffold00240-11 | hypothetical protein |
| supercontig_6.21-709 | Y34_scaffold00528-78 | P131_scaffold00240-9 | hypothetical protein |
| supercontig_6.21-714 | Y34_scaffold00528-83 | P131_scaffold00240-4 | arabinosidase |
| supercontig_6.21-740 | Y34_scaffold00522-49 | P131_scaffold01708-13 | hydrolase |
| supercontig_6.21-760 | Y34_scaffold00522-29 | P131_scaffold01046-29 | hypothetical protein |
| supercontig_6.21-769 | Y34_scaffold00522-20 | P131_scaffold01046-20 | pre-mRNA-splicing factor ATP-dependent RNA helicase PRP43 |
| supercontig_6.21-783 | Y34_scaffold00522-6 | P131_scaffold01046-6 | hypothetical protein |
| supercontig_6.21-79 | Y34_scaffold00759-2 | P131_scaffold01325-2 | putative glucoamylase GMY1 |
| supercontig_6.21-80 | Y34_scaffold00759-3 | P131_scaffold01325-3 | DNA binding protein SART-1 |
| supercontig_6.21-800 | Y34_scaffold00203-10 | P131_scaffold00593-12 | putative ATP-binding endoribonuclease |
| supercontig_6.21-802 | Y34_scaffold00203-12 | P131_scaffold00593-14 | no_hit |
| supercontig_6.21-804 | Y34_scaffold00203-15 | P131_scaffold00224-21 | hypothetical protein |
| supercontig_6.21-815 | Y34_scaffold00203-26 | P131_scaffold00224-10 | hypothetical protein |
| supercontig_6.21-834 | Y34_scaffold00203-45 | P131_scaffold00225-11 | sad1/UNC domain-containing protein |
| supercontig_6.21-851 | Y34_scaffold00203-62 | P131_scaffold00225-28 | variant SH3 domain containing protein |
| supercontig_6.21-86 | Y34_scaffold00759-9 | P131_scaffold01325-9 | DNA polymerase iota |
| supercontig_6.21-885 | Y34_scaffold00203-96 | P131_scaffold00470-2 | hypothetical protein |
| supercontig_6.21-886 | Y34_scaffold00203-97 | P131_scaffold00470-3 | o-methyltransferase |
| supercontig_6.21-90 | Y34_scaffold00053-4 | P131_scaffold01325-13 | hypothetical protein |
| supercontig_6.21-904 | Y34_scaffold00203-115 | P131_scaffold01024-6 | yip1 domain containing protein |
| supercontig_6.21-924 | Y34_scaffold00546-2 | P131_scaffold01073-14 | NACHT domain protein |
| supercontig_6.21-954 | Y34_scaffold00198-15 | P131_scaffold00220-25 | hypothetical protein |
| supercontig_6.21-962 | Y34_scaffold00608-4 | P131_scaffold00220-33 | DNA repair metallo-beta-lactamase family protein |
| supercontig_6.21-965 | Y34_scaffold00608-7 | P131_scaffold00220-36 | no_hit |
| supercontig_6.21-980 | Y34_scaffold00608-22 | P131_scaffold00220-51 | hypothetical protein 0.0 |
| supercontig_6.21-985 | Y34_scaffold00608-27 | P131_scaffold00220-56 | PCI domain containing protein |
| supercontig_6.21-997 | Y34_scaffold00608-39 | P131_scaffold00220-68 | hypothetical protein |
| supercontig_6.22-105 | Y34_scaffold00511-17 | P131_scaffold01234-5 | hypothetical protein |
| supercontig_6.22-113 | Y34_scaffold00511-25 | P131_scaffold00250-3 | dolichol kinase |
| supercontig_6.22-120 | Y34_scaffold00511-31 | P131_scaffold00484-2 | CobW domain protein |
| supercontig_6.22-133 | Y34_scaffold00511-44 | P131_scaffold00484-16 | UV-damage endonuclease |
| supercontig_6.22-163 | Y34_scaffold00745-4 | P131_scaffold01383-4 | hypothetical protein |
| supercontig_6.22-17 | Y34_scaffold00548-71 | P131_scaffold00142-15 | Oxidation resistance protein 1 |
| supercontig_6.22-177 | Y34_scaffold00745-18 | P131_scaffold01118-2 | ATP-dependent bile acid permease |
| supercontig_6.22-213 | Y34_scaffold00745-54 | P131_scaffold00314-26 | hexose transporter |
| supercontig_6.22-228 | Y34_scaffold00745-69 | P131_scaffold00314-41 | multidrug resistance protein 2 |
| supercontig_6.22-229 | Y34_scaffold00745-70 | P131_scaffold00314-42 | alkaline phosphatase |
| supercontig_6.22-237 | Y34_scaffold00745-78 | P131_scaffold00314-50 | M protein repeat protein |
| supercontig_6.22-246 | Y34_scaffold00745-87 | P131_scaffold00314-59 | ankyrin repeat-containing protein |
| supercontig_6.22-260 | Y34_scaffold00745-101 | P131_scaffold00314-73 | carbohydrate-binding module family 13 protein |
| supercontig_6.22-278 | Y34_scaffold00071-64 | P131_scaffold00314-91 | malate dehydrogenase |
| supercontig_6.22-280 | Y34_scaffold00071-62 | P131_scaffold00314-92 | DUF1295 domain protein |
| supercontig_6.22-293 | Y34_scaffold00071-49 | P131_scaffold00314-105 | hypothetical protein |
| supercontig_6.22-311 | Y34_scaffold00071-30 | P131_scaffold00314-123 | hypothetical protein |
| supercontig_6.22-329 | Y34_scaffold00071-12 | P131_scaffold00314-141 | condensin complex component SMC3 |
| supercontig_6.22-343 | Y34_scaffold00174-3 | P131_scaffold00412-4 | GTP binding protein (Bud4) |
| supercontig_6.22-347 | Y34_scaffold00174-7 | P131_scaffold00412-8 | ubiquitin C-terminal hydrolase |
| supercontig_6.22-348 | Y34_scaffold00174-8 | P131_scaffold00302-15 | hypothetical protein |
| supercontig_6.22-360 | Y34_scaffold00174-20 | P131_scaffold00302-3 | DUF858 domain protein |
| supercontig_6.22-361 | Y34_scaffold00174-21 | P131_scaffold00302-2 | regucalcin |
| supercontig_6.22-376 | Y34_scaffold00174-36 | P131_scaffold00266-55 | ankyrin repeat protein E4_2 |
| supercontig_6.22-390 | Y34_scaffold00174-50 | P131_scaffold00266-41 | BRCA1 C Terminus (BRCT) domain containing protein |
| supercontig_6.22-4 | Y34_scaffold00548-84 | P131_scaffold00142-2 | hypothetical protein |
| supercontig_6.22-407 | Y34_scaffold00174-68 | P131_scaffold00266-24 | phosphatidylethanolamine binding protein |
| supercontig_6.22-410 | Y34_scaffold00174-71 | P131_scaffold00266-21 | low-affinity zinc transporter of the plasma membrane |
| supercontig_6.22-426 | Y34_scaffold00174-87 | P131_scaffold00266-5 | hypothetical protein |
| supercontig_6.22-434 | Y34_scaffold00451-20 | P131_scaffold01684-16 | no_hit |
| supercontig_6.22-459 | Y34_scaffold00510-2 | P131_scaffold00613-2 | nucleoporin Nup157/170 |
| supercontig_6.22-472 | Y34_scaffold00510-15 | P131_scaffold01120-8 | general amino acid permease AGP3 |
| supercontig_6.22-479 | Y34_scaffold00510-22 | P131_scaffold01120-15 | ATP-dependent RNA helicase MRH4 |
| supercontig_6.22-486 | Y34_scaffold00510-29 | P131_scaffold01120-21 | hypothetical protein |
| supercontig_6.22-487 | Y34_scaffold00510-30 | P131_scaffold01120-22 | hypothetical protein |
| supercontig_6.22-499 | Y34_scaffold00516-7 | P131_scaffold00982-11 | no_hit |
| supercontig_6.22-518 | Y34_scaffold00516-26 | P131_scaffold00982-30 | ribosomal RNA-processing protein |
| supercontig_6.22-528 | Y34_scaffold00516-36 | P131_scaffold00982-40 | glyoxylate reductase |
| supercontig_6.22-540 | Y34_scaffold00516-48 | P131_scaffold00982-52 | related to brefeldin A resistance protein |
| supercontig_6.22-549 | Y34_scaffold00516-57 | P131_scaffold00982-61 | peroxin 26 |
| supercontig_6.22-560 | Y34_scaffold00516-68 | P131_scaffold00982-72 | hypothetical protein |
| supercontig_6.22-573 | Y34_scaffold00516-81 | P131_scaffold00982-85 | vacuolar ABC heavy metal transporter (Hmt1) |
| supercontig_6.22-580 | Y34_scaffold00516-88 | P131_scaffold00982-91 | acetoacetyl-CoA synthase |
| supercontig_6.22-582 | Y34_scaffold00516-90 | P131_scaffold00982-93 | possible transcription factor IIIc-like protein |
| supercontig_6.22-595 | Y34_scaffold00516-103 | P131_scaffold00432-3 | related to GTP-binding protein FZO1 |
| supercontig_6.22-597 | Y34_scaffold00516-105 | P131_scaffold00432-1 | hypothetical protein 0.0 |
| supercontig_6.22-616 | Y34_scaffold00526-9 | P131_scaffold01012-3 | F-box domain protein |
| supercontig_6.22-628 | Y34_scaffold00526-21 | P131_scaffold00159-11 | hypothetical protein |
| supercontig_6.22-633 | Y34_scaffold00526-26 | P131_scaffold00159-16 | transcription regulator PAB1642 |
| supercontig_6.22-644 | Y34_scaffold00711-34 | P131_scaffold01305-4 | hypothetical protein |
| supercontig_6.22-653 | Y34_scaffold00711-24 | P131_scaffold01006-22 | hypothetical protein |
| supercontig_6.22-656 | Y34_scaffold00711-21 | P131_scaffold01006-19 | hypothetical protein |
| supercontig_6.22-663 | Y34_scaffold00711-14 | P131_scaffold01006-12 | S1 RNA binding domain containing protein |
| supercontig_6.22-70 | Y34_scaffold00548-18 | P131_scaffold00321-8 | hypothetical protein |
| supercontig_6.22-708 | Y34_scaffold00033-15 | P131_scaffold00095-43 | hypothetical protein |
| supercontig_6.22-710 | Y34_scaffold00033-17 | P131_scaffold00095-41 | erythromycin esterase |
| supercontig_6.22-719 | Y34_scaffold00033-26 | P131_scaffold00095-32 | hypothetical protein |
| supercontig_6.22-723 | Y34_scaffold00033-30 | P131_scaffold00095-28 | hypothetical protein |
| supercontig_6.22-738 | Y34_scaffold00033-45 | P131_scaffold00095-13 | hypothetical protein |
| supercontig_6.22-81 | Y34_scaffold00548-7 | P131_scaffold00321-19 | E3 ubiquitin-protein ligase |
| supercontig_6.22-97 | Y34_scaffold00511-9 | P131_scaffold01234-13 | hypothetical protein |
| supercontig_6.23-11 | Y34_scaffold00202-4 | P131_scaffold01535-12 | hypothetical protein |
| supercontig_6.23-134 | Y34_scaffold00581-14 | P131_scaffold01153-1 | mating type transcriptional activator |
| supercontig_6.23-221 | Y34_scaffold00458-22 | P131_scaffold01310-22 | hypothetical protein |
| supercontig_6.23-238 | Y34_scaffold00458-39 | P131_scaffold01310-6 | hypothetical protein |
| supercontig_6.23-253 | Y34_scaffold00458-54 | P131_scaffold00941-10 | hypothetical protein |
| supercontig_6.23-264 | Y34_scaffold00150-10 | P131_scaffold00601-6 | fungal specific transcription factor |
| supercontig_6.23-284 | Y34_scaffold00210-8 | P131_scaffold01031-9 | NAD dependent epimerase/dehydratase |
| supercontig_6.23-302 | Y34_scaffold00233-10 | P131_scaffold00284-7 | hypothetical protein |
| supercontig_6.23-328 | Y34_scaffold00301-54 | P131_scaffold01387-7 | TATA-binding protein-associated phosphoprotein |
| supercontig_6.23-359 | Y34_scaffold00301-24 | P131_scaffold01054-53 | hypothetical protein |
| supercontig_6.23-372 | Y34_scaffold00301-11 | P131_scaffold01054-40 | hexose transporter |
| supercontig_6.23-375 | Y34_scaffold00301-8 | P131_scaffold01054-37 | hypothetical protein |
| supercontig_6.23-380 | Y34_scaffold00301-3 | P131_scaffold01054-31 | specific RNA polymerase II transcription factor |
| supercontig_6.23-397 | Y34_scaffold00666-16 | P131_scaffold01054-13 | G1/S-specific cyclin pas1 |
| supercontig_6.23-416 | Y34_scaffold00666-35 | P131_scaffold01340-6 | hypothetical protein |
| supercontig_6.23-436 | Y34_scaffold00666-55 | P131_scaffold01221-10 | hypothetical protein |
| supercontig_6.23-450 | Y34_scaffold00666-68 | P131_scaffold00596-4 | hypothetical protein |
| supercontig_6.23-452 | Y34_scaffold00666-70 | P131_scaffold00596-6 | myosin class II heavy chain (MHC) |
| supercontig_6.23-458 | Y34_scaffold00666-76 | P131_scaffold00596-11 | 60S ribosomal protein L24 |
| supercontig_6.23-467 | Y34_scaffold00666-85 | P131_scaffold00372-23 | hypothetical protein |
| supercontig_6.23-472 | Y34_scaffold00666-89 | P131_scaffold00372-18 | zinc finger protein DHHC domain containing protein |
| supercontig_6.23-478 | Y34_scaffold00666-95 | P131_scaffold00372-12 | DUF814 domain-containing protein |
| supercontig_6.23-479 | Y34_scaffold00666-96 | P131_scaffold00372-11 | woronin body major protein HEX1 |
| supercontig_6.23-481 | Y34_scaffold00666-98 | P131_scaffold00372-9 | hypothetical protein |
| supercontig_6.23-487 | Y34_scaffold00666-104 | P131_scaffold00372-4 | polyketide synthase |
| supercontig_6.23-493 | Y34_scaffold00666-110 | P131_scaffold00124-4 | rab proteins geranylgeranyltransferase component A |
| supercontig_6.23-500 | Y34_scaffold00666-117 | P131_scaffold01072-3 | phospholipase C |
| supercontig_6.23-561 | Y34_scaffold00666-179 | P131_scaffold01027-12 | related to ethanolaminephosphotransferase |
| supercontig_6.23-564 | Y34_scaffold00666-182 | P131_scaffold01027-15 | pH-response regulator protein palI/RIM9 |
| supercontig_6.23-569 | Y34_scaffold00666-187 | P131_scaffold01027-20 | rho guanyl nucleotide exchange factor |
| supercontig_6.23-571 | Y34_scaffold00666-189 | P131_scaffold01027-22 | staphylococcal nuclease domain-containing protein |
| supercontig_6.23-573 | Y34_scaffold00666-191 | P131_scaffold01027-24 | aflYe/ orf/ Ser -Thr protein phosphatase family protein |
| supercontig_6.23-599 | Y34_scaffold00666-217 | P131_scaffold01027-50 | hypothetical protein |
| supercontig_6.23-666 | Y34_scaffold00084-4 | P131_scaffold00169-4 | hypothetical protein |
| supercontig_6.23-672 | Y34_scaffold00265-5 | P131_scaffold00904-4 | low-affinity methionine permease |
| supercontig_6.23-720 | Y34_scaffold00308-41 | P131_scaffold01299-11 | no_hit |
| supercontig_6.23-723 | Y34_scaffold00308-44 | P131_scaffold01299-8 | myosin type II heavy chain |
| supercontig_6.23-728 | Y34_scaffold00308-49 | P131_scaffold01299-3 | aorsin endoprotease precursor |
| supercontig_6.23-731 | Y34_scaffold00308-52 | P131_scaffold01304-2 | RNA binding protein |
| supercontig_6.23-752 | Y34_scaffold00420-6 | P131_scaffold01020-6 | hypothetical protein |
| supercontig_6.23-812 | Y34_scaffold00777-23 | P131_scaffold01378-10 | hypothetical protein |
| supercontig_6.23-818 | Y34_scaffold00777-29 | P131_scaffold01378-16 | hypothetical protein |
| supercontig_6.23-865 | Y34_scaffold00539-6 | P131_scaffold00628-4 | hypothetical protein |
| supercontig_6.23-923 | Y34_scaffold00969-31 | P131_scaffold00516-21 | hypothetical protein |
| supercontig_6.23-930 | Y34_scaffold00969-25 | P131_scaffold00516-28 | putative protein of unknown function; the authentic |
| supercontig_6.23-931 | Y34_scaffold00969-24 | P131_scaffold00516-29 | hypothetical protein |
| supercontig_6.23-935 | Y34_scaffold00969-20 | P131_scaffold00102-20 | microbody (peroxisome) biogenesis protein peroxin 16 |
| supercontig_6.23-953 | Y34_scaffold00713-5 | P131_scaffold01003-5 | lignostilbene dioxygenase |
| supercontig_6.24-111 | Y34_scaffold00460-7 | P131_scaffold00957-17 | bZIP family transcription factor |
| supercontig_6.24-118 | Y34_scaffold00149-2 | P131_scaffold00331-23 | DEAD/DEAH box helicase |
| supercontig_6.24-125 | Y34_scaffold00149-9 | P131_scaffold00331-16 | hypothetical protein |
| supercontig_6.24-127 | Y34_scaffold00149-11 | P131_scaffold00331-14 | hypothetical protein |
| supercontig_6.24-145 | Y34_scaffold00464-121 | P131_scaffold00234-13 | phosphoinositide 3-kinase regulatory subunit 4 |
| supercontig_6.24-170 | Y34_scaffold00464-96 | P131_scaffold00550-9 | hypothetical protein |
| supercontig_6.24-192 | Y34_scaffold00464-73 | P131_scaffold00270-15 | phosphatidylinositol-specific phospholipase C |
| supercontig_6.24-197 | Y34_scaffold00464-68 | P131_scaffold00270-20 | hypothetical protein |
| supercontig_6.24-211 | Y34_scaffold00464-54 | P131_scaffold00270-34 | histone deacetylase HosB |
| supercontig_6.24-223 | Y34_scaffold00464-40 | P131_scaffold00439-22 | related to nicotinamide mononucleotide permease |
| supercontig_6.24-224 | Y34_scaffold00464-39 | P131_scaffold00439-21 | hypothetical protein |
| supercontig_6.24-233 | Y34_scaffold00464-30 | P131_scaffold00439-12 | glucan 1 |
| supercontig_6.24-234 | Y34_scaffold00464-29 | P131_scaffold00439-11 | hypothetical protein |
| supercontig_6.24-239 | Y34_scaffold00464-24 | P131_scaffold00439-6 | RNA polymerase I specific transcription initiation factor Rrn7 |
| supercontig_6.24-242 | Y34_scaffold00464-21 | P131_scaffold00439-3 | hypothetical protein |
| supercontig_6.24-250 | Y34_scaffold00464-13 | P131_scaffold00463-13 | hypothetical protein |
| supercontig_6.24-261 | Y34_scaffold00464-2 | P131_scaffold00463-2 | ubiquitin C-terminal hydrolase |
| supercontig_6.24-264 | Y34_scaffold00995-36 | P131_scaffold01322-2 | hypothetical protein |
| supercontig_6.24-265 | Y34_scaffold00995-35 | P131_scaffold01322-3 | sulfite oxidase |
| supercontig_6.24-266 | Y34_scaffold00995-34 | P131_scaffold01322-4 | 26S proteasome non-ATPase regulatory subunit 1 |
| supercontig_6.24-285 | Y34_scaffold00995-16 | P131_scaffold01322-22 | linoleate diol synthase precursor |
| supercontig_6.24-303 | Y34_scaffold00325-5 | P131_scaffold01322-40 | bZIP transcription factor HapX |
| supercontig_6.24-306 | Y34_scaffold00325-8 | P131_scaffold01322-43 | glutamyl-tRNA synthetase |
| supercontig_6.24-335 | Y34_scaffold00325-37 | P131_scaffold00184-8 | modin |
| supercontig_6.24-360 | Y34_scaffold00596-2 | P131_scaffold01327-1 | monoxygenase |
| supercontig_6.24-371 | Y34_scaffold00478-6 | P131_scaffold01327-12 | plasma membrane zinc ion transporter |
| supercontig_6.24-372 | Y34_scaffold00478-7 | P131_scaffold01327-13 | hypothetical protein |
| supercontig_6.24-378 | Y34_scaffold00620-5 | P131_scaffold00360-5 | meiotic helicase |
| supercontig_6.24-383 | Y34_scaffold00116-14 | P131_scaffold00035-1 | hypothetical protein |
| supercontig_6.24-388 | Y34_scaffold00116-10 | P131_scaffold00574-4 | beta-galactosidase |
| supercontig_6.24-398 | Y34_scaffold00163-1 | P131_scaffold00299-1 | hypothetical protein |
| supercontig_6.24-402 | Y34_scaffold00163-5 | P131_scaffold00299-5 | alpha/beta hydrolase fold protein |
| supercontig_6.24-403 | Y34_scaffold00163-6 | P131_scaffold00299-6 | HET domain protein |
| supercontig_6.24-427 | Y34_scaffold00163-28 | P131_scaffold00039-4 | histone deacetylase RpdA/Rpd3 |
| supercontig_6.24-441 | Y34_scaffold00282-5 | P131_scaffold01553-1 | hypothetical protein |
| supercontig_6.24-46 | Y34_scaffold00192-46 | P131_scaffold00455-48 | MSP domain containing protein |
| supercontig_6.24-473 | Y34_scaffold00197-5 | P131_scaffold00677-15 | hypothetical protein |
| supercontig_6.24-501 | Y34_scaffold00077-4 | P131_scaffold01565-2 | hypothetical protein |
| supercontig_6.24-55 | Y34_scaffold00192-37 | P131_scaffold00455-39 | disulfide isomerase |
| supercontig_6.24-71 | Y34_scaffold00192-22 | P131_scaffold00455-24 | APSES transcription factor Xbp1 |
| supercontig_6.24-74 | Y34_scaffold00192-19 | P131_scaffold00455-21 | hypothetical protein |
| supercontig_6.25-105 | Y34_scaffold01018-25 | P131_scaffold00278-8 | 37S ribosomal protein S12 |
| supercontig_6.25-111 | Y34_scaffold01018-19 | P131_scaffold00278-14 | hypothetical protein |
| supercontig_6.25-120 | Y34_scaffold01018-10 | P131_scaffold00096-10 | methyltransferase |
| supercontig_6.25-136 | Y34_scaffold00798-2 | P131_scaffold00104-3 | hypothetical protein |
| supercontig_6.25-150 | Y34_scaffold01075-31 | P131_scaffold00152-13 | hypothetical protein |
| supercontig_6.25-152 | Y34_scaffold01075-29 | P131_scaffold00152-11 | carboxypeptidase 2 |
| supercontig_6.25-160 | Y34_scaffold01075-21 | P131_scaffold00152-3 | HEAT repeat containing protein |
| supercontig_6.25-166 | Y34_scaffold01075-15 | P131_scaffold01342-7 | hypothetical protein |
| supercontig_6.25-169 | Y34_scaffold01075-12 | P131_scaffold01342-4 | polygalacturonase |
| supercontig_6.25-200 | Y34_scaffold00312-17 | P131_scaffold00515-17 | enoyl Coenzyme A hydratase domain containing 3 |
| supercontig_6.25-203 | Y34_scaffold00312-20 | P131_scaffold00515-20 | aromatic ring-opening dioxygenase family protein |
| supercontig_6.25-211 | Y34_scaffold00020-2 | P131_scaffold00515-28 | MFS transporter |
| supercontig_6.25-239 | Y34_scaffold01073-12 | P131_scaffold01517-14 | hypothetical protein |
| supercontig_6.25-24 | Y34_scaffold00706-10 | P131_scaffold00145-5 | hypothetical protein |
| supercontig_6.25-242 | Y34_scaffold01073-15 | P131_scaffold01517-11 | hypothetical protein |
| supercontig_6.25-245 | Y34_scaffold01073-18 | P131_scaffold01517-8 | hypothetical protein |
| supercontig_6.25-247 | Y34_scaffold01073-20 | P131_scaffold01517-6 | dihydroorotate dehydrogenase |
| supercontig_6.25-261 | Y34_scaffold01031-20 | P131_scaffold00206-2 | phospholipase D Active site motif protein |
| supercontig_6.25-262 | Y34_scaffold01031-19 | P131_scaffold00206-3 | aminopeptidase |
| supercontig_6.25-263 | Y34_scaffold01031-18 | P131_scaffold00206-4 | putative oxidoreductase |
| supercontig_6.25-264 | Y34_scaffold01031-17 | P131_scaffold00206-5 | mitochondrial ATPase (Afg1) |
| supercontig_6.25-29 | Y34_scaffold00706-14 | P131_scaffold00145-9 | hypothetical protein |
| supercontig_6.25-4 | Y34_scaffold00015-7 | P131_scaffold01548-3 | peptidase S41 family protein |
| supercontig_6.25-42 | Y34_scaffold00706-27 | P131_scaffold00280-5 | siderophore iron transporter mirB |
| supercontig_6.25-50 | Y34_scaffold00854-6 | P131_scaffold00383-6 | hypothetical protein |
| supercontig_6.25-99 | Y34_scaffold01018-31 | P131_scaffold00278-2 | hypothetical protein |
| supercontig_6.26-111 | Y34_scaffold00448-68 | P131_scaffold00332-6 | feruloyl esterase B precursor |
| supercontig_6.26-146 | Y34_scaffold00662-34 | P131_scaffold01288-2 | component of the RSC chromatin remodeling complex |
| supercontig_6.26-176 | Y34_scaffold00662-4 | P131_scaffold01337-25 | PH domain protein |
| supercontig_6.26-202 | Y34_scaffold00655-29 | P131_scaffold01337-51 | cysteine protease ATG4 |
| supercontig_6.26-203 | Y34_scaffold00655-28 | P131_scaffold01337-52 | C_2_H_2_ finger domain protein |
| supercontig_6.26-212 | Y34_scaffold00655-19 | P131_scaffold01337-61 | DUF1446 domain protein |
| supercontig_6.26-224 | Y34_scaffold00655-8 | P131_scaffold01337-73 | hypothetical protein 0.0 |
| supercontig_6.26-226 | Y34_scaffold00655-6 | P131_scaffold01337-75 | peptidase (PNG1) |
| supercontig_6.26-229 | Y34_scaffold00655-3 | P131_scaffold01337-78 | raffinose synthase Sip1 |
| supercontig_6.26-241 | Y34_scaffold00654-11 | P131_scaffold01320-11 | hypothetical protein |
| supercontig_6.26-248 | Y34_scaffold00654-18 | P131_scaffold01320-18 | related to Cutinase transcription factor 1 alpha |
| supercontig_6.26-262 | Y34_scaffold00654-31 | P131_scaffold00429-30 | glycerol transporter |
| supercontig_6.26-265 | Y34_scaffold00654-34 | P131_scaffold00429-27 | calcineurin-like phosphoesterase |
| supercontig_6.26-273 | Y34_scaffold00108-6 | P131_scaffold00429-19 | transcription initiation factor TFIId 127kD subunit |
| supercontig_6.26-293 | Y34_scaffold00161-5 | P131_scaffold01168-2 | hypothetical protein |
| supercontig_6.26-305 | Y34_scaffold00542-1 | P131_scaffold01168-14 | DNA repair exonuclease rad1 |
| supercontig_6.26-316 | Y34_scaffold00542-12 | P131_scaffold01168-25 | hypothetical protein |
| supercontig_6.26-329 | Y34_scaffold00542-24 | P131_scaffold01168-37 | estradiol 17-beta-dehydrogenase |
| supercontig_6.26-330 | Y34_scaffold00542-25 | P131_scaffold01168-38 | cholinephosphotransferase |
| supercontig_6.26-338 | Y34_scaffold00542-33 | P131_scaffold01168-46 | hypothetical protein |
| supercontig_6.26-346 | Y34_scaffold00542-41 | P131_scaffold01168-54 | NAP1-binding protein |
| supercontig_6.26-353 | Y34_scaffold00542-49 | P131_scaffold00378-38 | hypothetical protein |
| supercontig_6.26-354 | Y34_scaffold00542-50 | P131_scaffold00378-37 | high-affinity nicotinic acid transporter |
| supercontig_6.26-355 | Y34_scaffold00542-51 | P131_scaffold00378-36 | annexin ANXC4 |
| supercontig_6.26-42 | Y34_scaffold00437-2 | P131_scaffold00884-4 | hypothetical protein |
| supercontig_6.26-64 | Y34_scaffold00448-21 | P131_scaffold00503-21 | 2-hydroxyacid dehydrogenase |
| supercontig_6.26-75 | Y34_scaffold00448-32 | P131_scaffold00333-32 | hypothetical protein |
| supercontig_6.26-95 | Y34_scaffold00448-52 | P131_scaffold00333-12 | condensin component |
| supercontig_6.27-1007 | Y34_scaffold00590-55 | P131_scaffold00998-52 | GRAM domain-containing protein |
| supercontig_6.27-1009 | Y34_scaffold00590-57 | P131_scaffold00998-50 | cyclin domain-containing protein |
| supercontig_6.27-1015 | Y34_scaffold00590-63 | P131_scaffold00998-44 | hypothetical protein |
| supercontig_6.27-1016 | Y34_scaffold00590-64 | P131_scaffold00998-43 | transcription regulator BDF1 |
| supercontig_6.27-1026 | Y34_scaffold00590-74 | P131_scaffold00998-34 | small nuclear ribonucleoprotein E |
| supercontig_6.27-1028 | Y34_scaffold00590-76 | P131_scaffold00998-32 | oligopeptide transporter 4 |
| supercontig_6.27-103 | Y34_scaffold00744-15 | P131_scaffold01166-15 | beta-xylosidase |
| supercontig_6.27-1039 | Y34_scaffold00590-87 | P131_scaffold00998-22 | hypothetical protein |
| supercontig_6.27-1040 | Y34_scaffold00590-88 | P131_scaffold00998-21 | GMC oxidoreductase |
| supercontig_6.27-1051 | Y34_scaffold00590-99 | P131_scaffold00998-10 | recA family ATPase Rhp55 |
| supercontig_6.27-1054 | Y34_scaffold00590-102 | P131_scaffold00998-7 | hypothetical protein |
| supercontig_6.27-107 | Y34_scaffold00744-19 | P131_scaffold01166-19 | pentatricopeptide repeat containing protein |
| supercontig_6.27-1071 | Y34_scaffold00022-49 | P131_scaffold00301-12 | UAY_EMENI Positive regulator of purine utilization |
| supercontig_6.27-1079 | Y34_scaffold00022-41 | P131_scaffold00301-20 | origin recognition complex subunit 4 |
| supercontig_6.27-1082 | Y34_scaffold00022-38 | P131_scaffold00301-23 | regulator of G protein signalling Rgs1 |
| supercontig_6.27-1104 | Y34_scaffold00022-26 | P131_scaffold00219-11 | amino-acid acetyltransferase |
| supercontig_6.27-1108 | Y34_scaffold00022-22 | P131_scaffold00219-15 | no_hit |
| supercontig_6.27-1117 | Y34_scaffold00022-13 | P131_scaffold00219-23 | nitrogen assimilation transcription factor nit-4 |
| supercontig_6.27-1123 | Y34_scaffold00022-7 | P131_scaffold00219-29 | cytosolic phospholipase A2 |
| supercontig_6.27-113 | Y34_scaffold00744-25 | P131_scaffold01166-25 | glycosyltransferase family 39 |
| supercontig_6.27-1133 | Y34_scaffold00592-4 | P131_scaffold00469-14 | hypothetical protein |
| supercontig_6.27-1134 | Y34_scaffold00592-5 | P131_scaffold00469-13 | hypothetical protein |
| supercontig_6.27-1135 | Y34_scaffold00592-6 | P131_scaffold00469-12 | hypothetical protein |
| supercontig_6.27-1138 | Y34_scaffold00592-9 | P131_scaffold00469-9 | protein pyrABCN |
| supercontig_6.27-114 | Y34_scaffold00744-26 | P131_scaffold01166-26 | hypothetical protein |
| supercontig_6.27-1141 | Y34_scaffold00592-12 | P131_scaffold00469-7 | hypothetical protein |
| supercontig_6.27-1161 | Y34_scaffold00497-7 | P131_scaffold01457-14 | PHD finger and SET domain protein |
| supercontig_6.27-1164 | Y34_scaffold00497-10 | P131_scaffold01457-17 | nucleolar GTP-binding protein 2 |
| supercontig_6.27-1176 | Y34_scaffold00497-22 | P131_scaffold01457-29 | tyrosinase precursor |
| supercontig_6.27-1191 | Y34_scaffold01008-13 | P131_scaffold00005-5 | arsenite resistance protein Ars2 |
| supercontig_6.27-1216 | Y34_scaffold00162-12 | P131_scaffold01287-72 | hypothetical protein |
| supercontig_6.27-1228 | Y34_scaffold00162-24 | P131_scaffold01287-60 | rho guanyl nucleotide exchange factor |
| supercontig_6.27-1249 | Y34_scaffold00162-45 | P131_scaffold01287-39 | condensin complex component cnd2 |
| supercontig_6.27-1252 | Y34_scaffold00162-48 | P131_scaffold01287-36 | hypothetical protein |
| supercontig_6.27-1260 | Y34_scaffold00162-51 | P131_scaffold01287-32 | protein phosphatase methylesterase 1 |
| supercontig_6.27-1267 | Y34_scaffold00162-58 | P131_scaffold01287-25 | related to glyoxal oxidase precursor |
| supercontig_6.27-127 | Y34_scaffold00744-39 | P131_scaffold01358-6 | heat shock protein/chaperonin HSP78 |
| supercontig_6.27-1284 | Y34_scaffold00162-74 | P131_scaffold01287-8 | eukaryotic translation initiation factor 6 |
| supercontig_6.27-1285 | Y34_scaffold00162-75 | P131_scaffold01287-7 | hypothetical protein |
| supercontig_6.27-1288 | Y34_scaffold00162-78 | P131_scaffold01287-4 | hypothetical protein |
| supercontig_6.27-1295 | Y34_scaffold00147-16 | P131_scaffold00336-16 | related to nuclear pore protein |
| supercontig_6.27-1308 | Y34_scaffold00147-3 | P131_scaffold00336-3 | rho GDP dissociation inhibitor involved in the localization and regulation of Cdc42p |
| supercontig_6.27-133 | Y34_scaffold00744-45 | P131_scaffold01358-12 | hypothetical protein |
| supercontig_6.27-1339 | Y34_scaffold00433-17 | P131_scaffold00570-16 | hypothetical protein |
| supercontig_6.27-1343 | Y34_scaffold00433-21 | P131_scaffold00570-20 | acyltransferase Pth2 |
| supercontig_6.27-143 | Y34_scaffold00744-55 | P131_scaffold01358-22 | hypothetical protein |
| supercontig_6.27-152 | Y34_scaffold00744-64 | P131_scaffold01358-31 | hypothetical protein |
| supercontig_6.27-167 | Y34_scaffold00619-9 | P131_scaffold01358-46 | CGPB_FUSSO Cutinase gene palindrome-binding protein (PBP) |
| supercontig_6.27-170 | Y34_scaffold00619-12 | P131_scaffold01358-49 | ppx/GppA phosphatase family protein |
| supercontig_6.27-187 | Y34_scaffold00619-28 | P131_scaffold01358-66 | hypothetical protein |
| supercontig_6.27-192 | Y34_scaffold00619-32 | P131_scaffold01358-70 | hypothetical protein |
| supercontig_6.27-194 | Y34_scaffold00619-34 | P131_scaffold01358-72 | pyroglutamyl peptidase type I |
| supercontig_6.27-202 | Y34_scaffold00619-41 | P131_scaffold01358-79 | cellulose-binding family II |
| supercontig_6.27-205 | Y34_scaffold00619-44 | P131_scaffold01358-82 | hypothetical protein |
| supercontig_6.27-221 | Y34_scaffold00619-60 | P131_scaffold01358-98 | F-box domain protein |
| supercontig_6.27-222 | Y34_scaffold00619-61 | P131_scaffold01358-99 | hypothetical protein |
| supercontig_6.27-227 | Y34_scaffold00619-66 | P131_scaffold01358-104 | related to histone acetyltransferase |
| supercontig_6.27-255 | Y34_scaffold00749-10 | P131_scaffold00308-7 | hypothetical protein |
| supercontig_6.27-288 | Y34_scaffold00194-88 | P131_scaffold00268-6 | hypothetical protein |
| supercontig_6.27-299 | Y34_scaffold00194-77 | P131_scaffold00538-49 | hypothetical protein |
| supercontig_6.27-302 | Y34_scaffold00194-74 | P131_scaffold00538-46 | F-box domain protein |
| supercontig_6.27-311 | Y34_scaffold00194-65 | P131_scaffold00538-37 | monooxygenase |
| supercontig_6.27-335 | Y34_scaffold00194-41 | P131_scaffold00538-13 | PH domain protein |
| supercontig_6.27-340 | Y34_scaffold00194-36 | P131_scaffold00538-8 | ATP-dependent RNA helicase prp16 |
| supercontig_6.27-345 | Y34_scaffold00194-30 | P131_scaffold00538-2 | predicted protein |
| supercontig_6.27-348 | Y34_scaffold00194-27 | P131_scaffold00217-28 | hypothetical protein |
| supercontig_6.27-35 | Y34_scaffold00979-54 | P131_scaffold00888-32 | related to ribosomal protein YmL49 |
| supercontig_6.27-362 | Y34_scaffold00194-14 | P131_scaffold00217-14 | hypothetical protein |
| supercontig_6.27-363 | Y34_scaffold00194-13 | P131_scaffold00217-13 | nuclear pore complex protein An-Nup120 (Eurofung) |
| supercontig_6.27-38 | Y34_scaffold00979-51 | P131_scaffold00888-35 | hypothetical protein |
| supercontig_6.27-403 | Y34_scaffold00087-36 | P131_scaffold00411-4 | hypothetical protein |
| supercontig_6.27-404 | Y34_scaffold00087-35 | P131_scaffold00411-5 | mitochondrial import inner membrane translocase subunit tim-14 |
| supercontig_6.27-412 | Y34_scaffold00087-27 | P131_scaffold00194-2 | pre-mRNA-splicing factor CWC22 |
| supercontig_6.27-43 | Y34_scaffold00979-46 | P131_scaffold00888-40 | RNA binding protein |
| supercontig_6.27-433 | Y34_scaffold00087-7 | P131_scaffold00195-20 | hypothetical protein |
| supercontig_6.27-435 | Y34_scaffold00087-6 | P131_scaffold00195-22 | leucine Rich Repeat domain-containing protein |
| supercontig_6.27-436 | Y34_scaffold00087-5 | P131_scaffold00195-23 | small oligopeptide transporter |
| supercontig_6.27-451 | Y34_scaffold00228-57 | P131_scaffold00195-38 | transporter |
| supercontig_6.27-458 | Y34_scaffold00228-49 | P131_scaffold00195-45 | C-5 sterol desaturase |
| supercontig_6.27-466 | Y34_scaffold00228-41 | P131_scaffold00195-53 | RNA recognition domain-containing protein-containing protein |
| supercontig_6.27-467 | Y34_scaffold00228-40 | P131_scaffold00195-54 | condensin subunit Cnd3 |
| supercontig_6.27-468 | Y34_scaffold00228-39 | P131_scaffold00195-55 | pyruvate dehydrogenase protein x component |
| supercontig_6.27-469 | Y34_scaffold00228-38 | P131_scaffold00195-56 | magnesium transporter ALR1 |
| supercontig_6.27-472 | Y34_scaffold00228-35 | P131_scaffold00195-59 | flavin-containing amine oxidasedehydrogenase |
| supercontig_6.27-473 | Y34_scaffold00228-34 | P131_scaffold00195-60 | tyrosyl-DNA phosphodiesterase domain protein |
| supercontig_6.27-49 | Y34_scaffold00979-40 | P131_scaffold01075-5 | no_hit |
| supercontig_6.27-506 | Y34_scaffold00670-1 | P131_scaffold00399-2 | copper-sensing transcription factor |
| supercontig_6.27-507 | Y34_scaffold00240-72 | P131_scaffold01214-1 | hypothetical protein |
| supercontig_6.27-538 | Y34_scaffold00240-41 | P131_scaffold01214-32 | FYVE domain protein |
| supercontig_6.27-541 | Y34_scaffold00240-38 | P131_scaffold01214-35 | mitochondrial 54S ribosomal protein L51 |
| supercontig_6.27-545 | Y34_scaffold00240-34 | P131_scaffold01214-39 | serine/threonine-protein kinase tel-1 |
| supercontig_6.27-546 | Y34_scaffold00240-33 | P131_scaffold01214-40 | nuclear pore complex subunit Nup192 |
| supercontig_6.27-547 | Y34_scaffold00240-32 | P131_scaffold01214-41 | ubiquitin-protein ligase E3 Slx8 |
| supercontig_6.27-574 | Y34_scaffold00240-4 | P131_scaffold00625-3 | polygalacturonase |
| supercontig_6.27-581 | Y34_scaffold00642-2 | P131_scaffold01227-2 | hypothetical protein |
| supercontig_6.27-601 | Y34_scaffold00909-2 | P131_scaffold00612-1 | UDP-glucose |
| supercontig_6.27-612 | Y34_scaffold00473-1 | P131_scaffold01354-1 | hypothetical protein |
| supercontig_6.27-616 | Y34_scaffold00068-5 | P131_scaffold01267-4 | hypothetical protein |
| supercontig_6.27-638 | Y34_scaffold00921-2 | P131_scaffold00176-2 | hypothetical protein |
| supercontig_6.27-669 | Y34_scaffold00476-7 | P131_scaffold01028-4 | metaxin |
| supercontig_6.27-673 | Y34_scaffold00476-11 | P131_scaffold01028-8 | hypothetical protein |
| supercontig_6.27-674 | Y34_scaffold00476-12 | P131_scaffold01028-9 | hypothetical protein |
| supercontig_6.27-678 | Y34_scaffold00476-16 | P131_scaffold01028-13 | kinesin related protein 2 |
| supercontig_6.27-680 | Y34_scaffold00476-18 | P131_scaffold01028-15 | hypothetical protein |
| supercontig_6.27-681 | Y34_scaffold00476-19 | P131_scaffold01028-16 | beta-mannosidase |
| supercontig_6.27-695 | Y34_scaffold00476-34 | P131_scaffold01028-31 | hypothetical protein |
| supercontig_6.27-701 | Y34_scaffold00177-4 | P131_scaffold00253-3 | membrane bound cation transporter |
| supercontig_6.27-704 | Y34_scaffold00177-7 | P131_scaffold00253-6 | related to ser/thr protein kinase KIN4 |
| supercontig_6.27-710 | Y34_scaffold00177-12 | P131_scaffold01149-16 | hypothetical protein |
| supercontig_6.27-713 | Y34_scaffold00177-15 | P131_scaffold01149-14 | related to folylpolyglutamate synthetase |
| supercontig_6.27-727 | Y34_scaffold00177-29 | P131_scaffold00497-20 | hypothetical protein |
| supercontig_6.27-737 | Y34_scaffold00594-2 | P131_scaffold00497-10 | hypothetical protein |
| supercontig_6.27-74 | Y34_scaffold00979-15 | P131_scaffold01075-30 | hypothetical protein |
| supercontig_6.27-755 | Y34_scaffold00594-20 | P131_scaffold01065-6 | succinate-semialdehyde dehydrogenase |
| supercontig_6.27-766 | Y34_scaffold00594-31 | P131_scaffold00420-24 | hypothetical protein |
| supercontig_6.27-811 | Y34_scaffold00207-42 | P131_scaffold01061-3 | hypothetical protein |
| supercontig_6.27-819 | Y34_scaffold00207-50 | P131_scaffold01058-12 | G-patch domain protein |
| supercontig_6.27-85 | Y34_scaffold00979-4 | P131_scaffold01075-41 | putative glycosyl hydrolase |
| supercontig_6.27-850 | Y34_scaffold00103-6 | P131_scaffold01099-9 | major facilitator superfamily domain-containing protein 7-b |
| supercontig_6.27-853 | Y34_scaffold00103-9 | P131_scaffold01099-6 | no_hit |
| supercontig_6.27-854 | Y34_scaffold00103-10 | P131_scaffold01099-5 | GTPase binding protein Rid1 |
| supercontig_6.27-865 | Y34_scaffold00103-21 | P131_scaffold00178-7 | acetylserotonin methytransferase-like protein (ASMTL) |
| supercontig_6.27-87 | Y34_scaffold00979-2 | P131_scaffold01075-43 | hydrolase |
| supercontig_6.27-896 | Y34_scaffold00636-12 | P131_scaffold01639-12 | mitogen-activated protein kinase MAF1 |
| supercontig_6.27-898 | Y34_scaffold00636-10 | P131_scaffold01639-10 | hypothetical protein |
| supercontig_6.27-901 | Y34_scaffold00636-7 | P131_scaffold01639-7 | hypothetical protein |
| supercontig_6.27-906 | Y34_scaffold00636-2 | P131_scaffold01639-2 | mediator of RNA polymerase II transcription subunit 5 |
| supercontig_6.27-919 | Y34_scaffold00530-2 | P131_scaffold00131-13 | hypothetical protein 0.0 |
| supercontig_6.27-937 | Y34_scaffold00245-17 | P131_scaffold00507-6 | hypothetical protein |
| supercontig_6.27-962 | Y34_scaffold00590-10 | P131_scaffold00252-1 | folylpolyglutamate synthase |
| supercontig_6.27-965 | Y34_scaffold00590-13 | P131_scaffold00955-3 | hypothetical protein |
| supercontig_6.27-973 | Y34_scaffold00590-21 | P131_scaffold00955-11 | WD repeat-containing protein |
| supercontig_6.27-976 | Y34_scaffold00590-24 | P131_scaffold00955-14 | probable ubiquitin-protein ligase |
| supercontig_6.27-979 | Y34_scaffold00590-27 | P131_scaffold00955-17 | hypothetical protein |
| supercontig_6.27-989 | Y34_scaffold00590-37 | P131_scaffold00998-70 | hypothetical protein |
| supercontig_6.27-991 | Y34_scaffold00590-39 | P131_scaffold00998-68 | hypothetical protein |
| supercontig_6.27-995 | Y34_scaffold00590-43 | P131_scaffold00998-64 | hexokinase-1 |
| supercontig_6.27-997 | Y34_scaffold00590-45 | P131_scaffold00998-62 | pre-mRNA-splicing factor CEF1 |
| supercontig_6.28-10 | Y34_scaffold00257-7 | P131_scaffold00193-2 | tetracycline-efflux transporter |
| supercontig_6.28-196 | Y34_scaffold00070-2 | P131_scaffold01364-2 | C6 zinc finger domain-containing protein |
| supercontig_6.28-211 | Y34_scaffold00733-13 | P131_scaffold01225-2 | hypothetical protein |
| supercontig_6.28-237 | Y34_scaffold01165-9 | P131_scaffold00655-12 | hypothetical protein |
| supercontig_6.28-240 | Y34_scaffold01165-12 | P131_scaffold00655-15 | ankyrin repeat domain protein |
| supercontig_6.28-241 | Y34_scaffold01165-13 | P131_scaffold00655-16 | hypothetical protein |
| supercontig_6.28-248 | Y34_scaffold00303-5 | P131_scaffold00583-3 | vitamin H transporter |
| supercontig_6.28-254 | Y34_scaffold00692-50 | P131_scaffold01240-3 | aldehyde dehydrogenase 3F1 |
| supercontig_6.28-26 | Y34_scaffold00287-3 | P131_scaffold00984-30 | thioesterase domain protein |
| supercontig_6.28-274 | Y34_scaffold00692-38 | P131_scaffold00279-29 | endoglucanase-6B |
| supercontig_6.28-277 | Y34_scaffold00692-35 | P131_scaffold00279-26 | O-methyltransferase |
| supercontig_6.28-285 | Y34_scaffold00692-27 | P131_scaffold00279-18 | hypothetical protein |
| supercontig_6.28-288 | Y34_scaffold00692-25 | P131_scaffold00279-16 | no_hit |
| supercontig_6.28-292 | Y34_scaffold00692-21 | P131_scaffold00279-12 | hypothetical protein |
| supercontig_6.28-337 | Y34_scaffold00343-1 | P131_scaffold00015-1 | hypothetical protein |
| supercontig_6.28-390 | Y34_scaffold00193-12 | P131_scaffold01380-14 | DUF636 domain-containing protein |
| supercontig_6.28-395 | Y34_scaffold00193-16 | P131_scaffold01380-18 | hypothetical protein |
| supercontig_6.28-53 | Y34_scaffold00054-10 | P131_scaffold00984-5 | dipeptidyl aminopeptidase |
| supercontig_6.28-77 | Y34_scaffold00090-5 | P131_scaffold01278-5 | F-box domain protein |
| supercontig_6.28-79 | Y34_scaffold00090-3 | P131_scaffold01278-3 | hypothetical protein |
| supercontig_6.28-88 | Y34_scaffold00030-8 | P131_scaffold01612-3 | no_hit |
| supercontig_6.28-97 | Y34_scaffold00050-1 | P131_scaffold00759-5 | negative-acting regulatory protein |
| supercontig_6.28-99 | Y34_scaffold00050-3 | P131_scaffold00759-3 | 3-dehydroshikimate dehydratase |
| supercontig_6.29-108 | Y34_scaffold00094-47 | P131_scaffold01076-15 | glycosyl hydrolase |
| supercontig_6.29-11 | Y34_scaffold00492-51 | P131_scaffold01519-12 | related to nuclear pore protein |
| supercontig_6.29-110 | Y34_scaffold00094-49 | P131_scaffold01076-17 | pre-mRNA-processing factor 17 |
| supercontig_6.29-117 | Y34_scaffold00094-56 | P131_scaffold01076-24 | methionine aminopeptidase |
| supercontig_6.29-119 | Y34_scaffold00094-58 | P131_scaffold01076-26 | WD repeat protein |
| supercontig_6.29-121 | Y34_scaffold00094-60 | P131_scaffold01076-28 | thiamine-repressible mitochondrial transporter THI74 |
| supercontig_6.29-126 | Y34_scaffold00094-65 | P131_scaffold01076-33 | thioredoxin domain containing protein |
| supercontig_6.29-136 | Y34_scaffold00094-74 | P131_scaffold00276-8 | no_hit |
| supercontig_6.29-137 | Y34_scaffold00094-75 | P131_scaffold00276-7 | hypothetical protein |
| supercontig_6.29-149 | Y34_scaffold00094-87 | P131_scaffold01180-8 | transmembrane E3 ubiquitin-protein ligase |
| supercontig_6.29-164 | Y34_scaffold00638-3 | P131_scaffold01039-8 | hypothetical protein |
| supercontig_6.29-179 | Y34_scaffold00610-13 | P131_scaffold01039-23 | related to aromatic amino acid aminotransferase I |
| supercontig_6.29-182 | Y34_scaffold00610-16 | P131_scaffold01039-26 | related to ser/arg-related nuclear matrix protein |
| supercontig_6.29-187 | Y34_scaffold00610-21 | P131_scaffold01039-31 | hypothetical protein |
| supercontig_6.29-189 | Y34_scaffold00610-23 | P131_scaffold01039-33 | cutinase transcription factor 1 beta |
| supercontig_6.29-191 | Y34_scaffold00610-25 | P131_scaffold00323-1 | L-ascorbate peroxidase |
| supercontig_6.29-223 | Y34_scaffold00610-56 | P131_scaffold00323-32 | hypothetical protein |
| supercontig_6.29-23 | Y34_scaffold00492-39 | P131_scaffold00264-39 | hypothetical protein |
| supercontig_6.29-234 | Y34_scaffold00610-67 | P131_scaffold00323-43 | related to phosphatidylserine decarboxylase |
| supercontig_6.29-235 | Y34_scaffold00610-68 | P131_scaffold00323-44 | metalloproteinase |
| supercontig_6.29-247 | Y34_scaffold00707-73 | P131_scaffold00141-13 | epoxide hydrolase |
| supercontig_6.29-288 | Y34_scaffold00707-32 | P131_scaffold01192-30 | 3' exoribonuclease |
| supercontig_6.29-295 | Y34_scaffold00707-25 | P131_scaffold01192-37 | pre-mRNA-splicing factor SYF1 |
| supercontig_6.29-301 | Y34_scaffold00707-19 | P131_scaffold01192-43 | hypothetical protein |
| supercontig_6.29-32 | Y34_scaffold00492-30 | P131_scaffold00264-30 | pantetheine-phosphate adenylyltransferase family protein |
| supercontig_6.29-33 | Y34_scaffold00492-29 | P131_scaffold00264-29 | WD repeat-containing protein |
| supercontig_6.29-353 | Y34_scaffold00773-23 | P131_scaffold01179-28 | nucleolar GTP-binding protein |
| supercontig_6.29-363 | Y34_scaffold00773-13 | P131_scaffold00922-2 | gamma-tubulin complex component GCP6 |
| supercontig_6.29-379 | Y34_scaffold00618-32 | P131_scaffold00922-18 | SNF2 family DNA-dependent ATPase |
| supercontig_6.29-392 | Y34_scaffold00618-19 | P131_scaffold00922-31 | argininosuccinate synthase |
| supercontig_6.29-416 | Y34_scaffold00545-4 | P131_scaffold00461-3 | glutamate synthase precursor |
| supercontig_6.29-44 | Y34_scaffold00492-18 | P131_scaffold00264-18 | NADP-specific glutamate dehydrogenase |
| supercontig_6.29-442 | Y34_scaffold00703-20 | P131_scaffold01275-2 | D-3-phosphoglycerate dehydrogenase |
| supercontig_6.29-446 | Y34_scaffold00703-24 | P131_scaffold01275-6 | putative microbody (peroxisome) proliferation protein peroxin 23-like |
| supercontig_6.29-449 | Y34_scaffold00703-27 | P131_scaffold01275-9 | LPS glycosyltransferase |
| supercontig_6.29-493 | Y34_scaffold01180-5 | P131_scaffold00204-2 | cytochrome P450 |
| supercontig_6.29-494 | Y34_scaffold01180-6 | P131_scaffold00204-3 | hypothetical protein |
| supercontig_6.29-495 | Y34_scaffold01180-7 | P131_scaffold00204-4 | Pfs |
| supercontig_6.29-50 | Y34_scaffold00492-11 | P131_scaffold00264-11 | peptidyl-prolyl cis-trans isomerase B |
| supercontig_6.29-509 | Y34_scaffold00855-2 | P131_scaffold01154-1 | fungal specific transcription factor |
| supercontig_6.29-51 | Y34_scaffold00492-10 | P131_scaffold00264-10 | related to syntaxin family member TLG1 |
| supercontig_6.29-512 | Y34_scaffold00597-30 | P131_scaffold00421-11 | glutaminase GtaA |
| supercontig_6.29-517 | Y34_scaffold00597-25 | P131_scaffold00421-6 | C2H2 finger domain protein |
| supercontig_6.29-546 | Y34_scaffold00768-39 | P131_scaffold01066-26 | related to CELL DIVISION CYCLE 2-RELATED PROTEIN KINASE 7 |
| supercontig_6.29-573 | Y34_scaffold00768-11 | P131_scaffold01246-5 | C2H2 finger domain protein |
| supercontig_6.29-581 | Y34_scaffold00768-3 | P131_scaffold00535-4 | hypothetical protein |
| supercontig_6.29-596 | Y34_scaffold00461-33 | P131_scaffold00200-9 | actin polymerization protein Bzz1 |
| supercontig_6.29-599 | Y34_scaffold00461-30 | P131_scaffold00200-12 | CDC50 domain containing protein |
| supercontig_6.29-601 | Y34_scaffold00461-28 | P131_scaffold00200-14 | BCAS2 domain-containing protein |
| supercontig_6.29-604 | Y34_scaffold00461-25 | P131_scaffold00200-17 | C_2_H_2_ type zinc finger domain protein |
| supercontig_6.29-608 | Y34_scaffold00461-21 | P131_scaffold00200-21 | no_hit |
| supercontig_6.29-611 | Y34_scaffold00461-18 | P131_scaffold01551-7 | hypothetical protein |
| supercontig_6.29-623 | Y34_scaffold00461-6 | P131_scaffold01250-6 | isoamyl alcohol oxidase |
| supercontig_6.29-686 | Y34_scaffold01016-3 | P131_scaffold00063-2 | hypothetical protein |
| supercontig_6.29-692 | Y34_scaffold00503-3 | P131_scaffold00996-2 | aminopeptidase Y |
| supercontig_6.29-725 | Y34_scaffold01028-5 | P131_scaffold01619-5 | hypothetical protein |
| supercontig_6.29-733 | Y34_scaffold01028-13 | P131_scaffold01619-14 | hypothetical protein |
| supercontig_6.29-739 | Y34_scaffold00237-2 | P131_scaffold01608-2 | hypothetical protein |
| supercontig_6.29-743 | Y34_scaffold00269-3 | P131_scaffold00028-3 | no_hit |
| supercontig_6.29-744 | Y34_scaffold00269-2 | P131_scaffold00028-2 | putative chitin deacetylase |
| supercontig_6.29-78 | Y34_scaffold00094-17 | P131_scaffold00067-8 | ras GTPase-activating protein gap-1 |
| supercontig_6.29-816 | Y34_scaffold00766-32 | P131_scaffold00274-8 | INO80 chromatin remodeling complex Ies1 |
| supercontig_6.29-821 | Y34_scaffold00766-26 | P131_scaffold00274-3 | abhydrolase domain-containing protein |
| supercontig_6.29-857 | Y34_scaffold00555-5 | P131_scaffold00431-4 | purine transporter |
| supercontig_6.29-883 | Y34_scaffold00925-19 | P131_scaffold01570-6 | regulatory protein ral2 |
| supercontig_6.29-911 | Y34_scaffold00989-2 | P131_scaffold00856-2 | cytochrome P450 52A11 |
| supercontig_6.4-108 | Y34_scaffold00641-46 | P131_scaffold00254-21 | poly(A) RNA polymerase cid13 |
| supercontig_6.4-109 | Y34_scaffold00641-45 | P131_scaffold00254-22 | hypothetical protein |
| supercontig_6.4-124 | Y34_scaffold00641-30 | P131_scaffold00254-37 | GATA transcription factor |
| supercontig_6.4-126 | Y34_scaffold00641-28 | P131_scaffold00254-39 | SAP domain-containing protein |
| supercontig_6.4-133 | Y34_scaffold00641-21 | P131_scaffold00491-4 | hypothetical protein |
| supercontig_6.4-138 | Y34_scaffold00641-16 | P131_scaffold00288-1 | no_hit |
| supercontig_6.4-170 | Y34_scaffold00887-3 | P131_scaffold00509-3 | MFS transporter |
| supercontig_6.4-228 | Y34_scaffold00518-2 | P131_scaffold01228-1 | hypothetical protein |
| supercontig_6.4-238 | Y34_scaffold00927-9 | P131_scaffold00062-9 | hypothetical protein |
| supercontig_6.4-4 | Y34_scaffold00521-2 | P131_scaffold01100-1 | hypothetical protein |
| supercontig_6.4-48 | Y34_scaffold00584-11 | P131_scaffold00974-6 | C_2_H_2_ finger domain protein |
| supercontig_6.4-50 | Y34_scaffold00584-13 | P131_scaffold00974-4 | probable aflatoxin efflux pump AFLT |
| supercontig_6.4-73 | Y34_scaffold00735-7 | P131_scaffold00100-5 | hypothetical protein |
| supercontig_6.4-85 | Y34_scaffold00641-70 | P131_scaffold00973-6 | ketol-acid reductoisomerase |
| supercontig_6.4-91 | Y34_scaffold00641-64 | P131_scaffold00254-3 | hypothetical protein |
| supercontig_6.4-96 | Y34_scaffold00641-58 | P131_scaffold00254-9 | MFS transporter |
| supercontig_6.7-133 | Y34_scaffold01196-3 | P131_scaffold01690-1 | hypothetical protein |
| supercontig_6.7-30 | Y34_scaffold00685-10 | P131_scaffold00367-4 | oligopeptide transporter |
| supercontig_6.7-32 | Y34_scaffold00685-12 | P131_scaffold00367-6 | nucleoside diphosphate kinase |
| supercontig_6.7-33 | Y34_scaffold00685-13 | P131_scaffold00367-7 | related to glucosidase II |
| supercontig_6.7-41 | Y34_scaffold00685-21 | P131_scaffold00367-15 | hypothetical protein |
| supercontig_6.7-50 | Y34_scaffold00685-30 | P131_scaffold00367-24 | predicted GTPase |
| supercontig_6.7-68 | Y34_scaffold01090-8 | P131_scaffold01208-8 | hypothetical protein |
| supercontig_6.7-84 | Y34_scaffold00536-1 | P131_scaffold01002-1 | hypothetical protein |
| supercontig_6.8-112 | Y34_scaffold00489-6 | P131_scaffold00725-4 | hypothetical protein |
| supercontig_6.8-137 | Y34_scaffold00412-3 | P131_scaffold01008-13 | hypothetical protein |
| supercontig_6.8-157 | Y34_scaffold00076-2 | P131_scaffold00031-4 | GPI-anchored cell wall beta-1 |
| supercontig_6.8-163 | Y34_scaffold00205-2 | P131_scaffold00025-3 | hypothetical protein |
| supercontig_6.8-172 | Y34_scaffold00101-5 | P131_scaffold00182-12 | endopolygalacturonase |
| supercontig_6.8-182 | Y34_scaffold00133-2 | P131_scaffold00182-2 | cytochrome b5 reductase |
| supercontig_6.8-25 | Y34_scaffold00334-25 | P131_scaffold00344-18 | 12-oxophytodienoate reductase |
| supercontig_6.8-28 | Y34_scaffold00334-28 | P131_scaffold00344-21 | homogentisate oxidase family member |
| supercontig_6.8-3 | Y34_scaffold00334-3 | P131_scaffold00544-4 | glutamyl-tRNA(Gln) amidotransferase |
| supercontig_6.8-32 | Y34_scaffold00334-32 | P131_scaffold00344-25 | hypothetical protein |
| supercontig_6.8-59 | Y34_scaffold00652-3 | P131_scaffold00146-4 | hypothetical protein |
| supercontig_6.8-79 | Y34_scaffold00609-2 | P131_scaffold01586-2 | hypothetical protein |
| supercontig_6.8-95 | Y34_scaffold01047-2 | P131_scaffold01242-2 | beta-mannosidase |
| supercontig_6.9-11 | Y34_scaffold00535-13 | P131_scaffold01289-9 | CDF zinc ion transporter |
| supercontig_6.9-114 | Y34_scaffold00679-25 | P131_scaffold01689-26 | hypothetical protein |
| supercontig_6.9-124 | Y34_scaffold00679-15 | P131_scaffold01689-16 | hypothetical protein |
| supercontig_6.9-127 | Y34_scaffold00679-12 | P131_scaffold01689-13 | arsenate reductase (Arc2) |
| supercontig_6.9-135 | Y34_scaffold00679-4 | P131_scaffold01689-5 | hypothetical protein |
| supercontig_6.9-36 | Y34_scaffold00229-2 | P131_scaffold00327-2 | predicted protein |
| supercontig_6.9-41 | Y34_scaffold00932-2 | P131_scaffold00327-7 | hypothetical protein |
| supercontig_6.9-58 | Y34_scaffold00601-2 | P131_scaffold00156-11 | L-lactate dehydrogenase |
