# Supplementary material for: Comparative Analysis of the Genomes of Two Field Isolates of the Rice Blast Fungus Magnaporthe oryzae
Source: PLoS Genet. 2012 Aug 2;8(8):e1002869. doi: 10.1371/journal.pgen.1002869 (PMC3410873; doi:10.1371/journal.pgen.1002869)
Supplement: Table S13 — Genes of isolates P131 and Y34 mapped against chromosomal assembly of 70-15 and found to be disrupted by TE. (DOC) [file pgen.1002869.s021.doc]

**Table S13** Genes of isolates P131 and Y34 mapped against chromosomal assembly of 70-15 and found to be disrupted by TE.

| **Chromosome** | **Begin** | **End** | **TE** | **P131 gene** | **Y34 gene** | **PSORT** | **Annotation** |
| --- | --- | --- | --- | --- | --- | --- | --- |
| III | 1746319 | 1748230 | MGL | - | Y34_scaffold005212-1 | cysk | hypothetical protein |
| VI | 164185 | 166042 | Pot2/Pot4 | P131_scaffold001218-4 | - | cysk | heterokaryon incompatibility protein |
| IV | 1880969 | 1882829 | Pot3 | - | Y34_scaffold000156-3 | cysk | riboflavin biosynthesis protein |
| II | 5003465 | 5003646 | cluster8 | - | Y34_scaffold007687-1 | cyto | actin-like ATPase |
| II | 6403716 | 6409353 | Maggy | P131_scaffold002215-1 | - | cyto | hypothetical protein |
| II | 267386 | 273365 | MGL | - | Y34_scaffold000239-3 | cyto | Yippee zinc-binding protein |
| II | 1160778 | 1163532 | MGL | - | Y34_scaffold000467-1 | cyto | hypothetical protein |
| I | 713468 | 716156 | Occan | - | Y34_scaffold002543-1 | cyto | Pfs, NACHT and Ankyrin domain protein |
| III | 2200541 | 2203229 | Occan | - | Y34_scaffold002459-1 | cyto | hypothetical protein |
| I | 7432885 | 7434745 | Pot2/Pot4 | P131_scaffold007868-1 | Y34_scaffold000110-5 | cyto | antimicrobial peptides |
| II | 5004229 | 5005723 | Pot2/Pot4 | - | Y34_scaffold007687-1 | cyto | hypothetical protein |
| III | 6489285 | 6491129 | Pot2/Pot4 | P131_scaffold003771-1 | - | cyto | proline dipeptidase |
| VII | 980344 | 982204 | Pot3 | P131_scaffold000459-3 | - | cyto | hypothetical protein |
| VI | 4830339 | 4830794 | Pyret | P131_scaffold001594-2 | - | cyto | hypothetical protein |
| IV | 3240196 | 3240419 | RETRO6 | - | Y34_scaffold000326-1 | cyto | FAD linked oxidase |
| VII | 736718 | 736772 | cluster3 | - | Y34_scaffold000804-2 | cyto_nucl | hypothetical protein |
| III | 6197223 | 6199080 | Pot2/Pot4 | - | Y34_scaffold002874-1 | cyto_nucl | hypothetical protein |
| III | 1146046 | 1147904 | Pot2/Pot4 | - | Y34_scaffold010782-1 | cyto_nucl | hypothetical protein |
| IV | 1279906 | 1281763 | Pot2/Pot4 | P131_scaffold000689-3 | Y34_scaffold002530-1 | cyto_nucl | similar to phosphoesterase |
| VII | 93329 | 95189 | Pot2/Pot4 | - | Y34_scaffold000757-2 | cyto_nucl | hypothetical protein |
| I | 7622934 | 7623891 | Pyret | P131_scaffold001810-2 | Y34_scaffold004575-1 | cyto_nucl | hypothetical protein |
| VII | 1691046 | 1698662 | RETRO6 | P131_scaffold002657-1 | - | cyto_nucl | exopolygalacturonase |
| IV | 3382753 | 3382967 | RETRO7 | P131_scaffold000367-4 | - | cyto_nucl | hypothetical protein |
| V | 5043739 | 5046338 | cluster1 | P131_scaffold000279-3 | - | extr | hypothetical protein |
| II | 348520 | 348628 | cluster3 | P131_scaffold001954-1 | - | extr | hypothetical protein |
| III | 6022337 | 6022568 | cluster3 | P131_scaffold008458-1 | Y34_scaffold001839-1 | extr | hypothetical protein |
| II | 297927 | 298126 | cluster8 | - | Y34_scaffold000289-7 | extr | hypothetical protein |
| I | 2249983 | 2262998 | Maggy | - | Y34_scaffold001470-2 | extr | hypothetical protein |
| I | 5288669 | 5291032 | MGL | - | Y34_scaffold000124-2 | extr | histidine acid phosphatase |
| II | 870897 | 876876 | MGL | P131_scaffold002069-1 | - | extr | hypothetical protein |
| I | 7822296 | 7822501 | Mg-MINE | P131_scaffold000080-1 | - | extr | hypothetical protein |
| II | 4948734 | 4949207 | Mg-SINE | P131_scaffold000713-2 | - | extr | arylsulfatase, putative |
| III | 3583408 | 3583880 | Mg-SINE | P131_scaffold001580-1 | Y34_scaffold001142-2 | extr | hypothetical protein |
| III | 1087318 | 1087791 | Mg-SINE | P131_scaffold004648-1 | - | extr | hypothetical protein |
| VI | 3570064 | 3570536 | Mg-SINE | P131_scaffold002218-1 | Y34_scaffold000448-4 | extr | hypothetical protein |
| VI | 3588672 | 3589146 | Mg-SINE | P131_scaffold000010-12 | - | extr | polyketide synthase |
| VI | 993803 | 994276 | Mg-SINE | P131_scaffold002133-1 | - | extr | kelch repeat protein |
| VII | 4506987 | 4507460 | Mg-SINE | P131_scaffold000342-3 | Y34_scaffold000468-1 | extr | hypothetical protein |
| IV | 4227063 | 4229751 | Occan | - | Y34_scaffold000222-1 | extr | snake toxin like protein |
| VII | 384774 | 384826 | Occan | P131_scaffold002043-1 | - | extr | hypothetical protein |
| III | 1865430 | 1867287 | Pot2/Pot4 | P131_scaffold003734-1 | - | extr | hypothetical protein |
| VII | 227828 | 229788 | Pot2/Pot4 | P131_scaffold001190-1 | Y34_scaffold002158-1 | extr | AVR-Pita1 |
| VII | 1898695 | 1900556 | Pot2/Pot4 | - | Y34_scaffold002639-1 | extr | peptidase M35 |
| II | 5922946 | 5923094 | Pyret | P131_scaffold001615-2 | - | extr | hypothetical protein |
| VII | 1713205 | 1717159 | Pyret | P131_scaffold004591-1 | Y34_scaffold000853-1 | extr | hypothetical protein |
| I | 4332704 | 4340239 | RETRO5 | - | Y34_scaffold001375-2 | extr | tRNA dihydrouridine synthase Smm1 |
| I | 430809 | 430937 | RETRO5 | - | Y34_scaffold002395-1 | extr | hypothetical protein |
| V | 4884356 | 4884549 | RETRO6 | - | Y34_scaffold003013-1 | extr | fungal specific transcription factor |
| VII | 1522433 | 1525326 | RETRO6 | - | Y34_scaffold005055-1 | extr | putative transposase |
| VII | 2289964 | 2290459 | cluster1 | - | Y34_scaffold000306-1 | mito | DNase I-like |
| UNKNOWN | 51517 | 51643 | cluster7 | - | Y34_scaffold000087-7 | mito | hypothetical protein |
| I | 5804672 | 5804862 | cluster8 | - | Y34_scaffold003227-1 | mito | hypothetical protein |
| IV | 3846179 | 3846299 | cluster8 | - | Y34_scaffold003679-1 | mito | dihydrouridine synthase |
| VII | 170952 | 171176 | cluster8 | - | Y34_scaffold000325-1 | mito | hypothetical protein |
| III | 6200001 | 6200097 | cluster9 | P131_scaffold005753-1 | - | mito | hypothetical protein |
| II | 2951773 | 2955001 | Maggy | P131_scaffold005204-1 | Y34_scaffold000158-1 | mito | hypothetical protein |
| V | 5115726 | 5126384 | Maggy | - | Y34_scaffold003408-1 | mito | hypothetical protein |
| III | 33297 | 36624 | MGL | P131_scaffold008795-1 | Y34_scaffold001795-1 | mito | hypothetical protein |
| III | 6130024 | 6136003 | MGL | P131_scaffold001412-3 | Y34_scaffold003045-1 | mito | nuclear protein SET |
| IV | 245059 | 250165 | MGL | P131_scaffold001590-1 | - | mito | ARCA-like protein |
| V | 4874565 | 4880545 | MGL | - | Y34_scaffold007080-1 | mito | 3'-5' exonuclease |
| V | 143059 | 145359 | MGL | P131_scaffold001261-2 | - | mito | hypothetical protein |
| I | 252076 | 252550 | Mg-SINE | - | Y34_scaffold001907-1 | mito | zinc finger protein |
| I | 5369039 | 5369511 | Mg-SINE | P131_scaffold000018-3 | - | mito | hypothetical protein |
| I | 7486629 | 7487102 | Mg-SINE | P131_scaffold003061-1 | - | mito | hypothetical protein |
| II | 4718924 | 4719397 | Mg-SINE | P131_scaffold001104-2 | - | mito | hypothetical protein |
| III | 497893 | 498367 | Mg-SINE | P131_scaffold001995-2 | Y34_scaffold000461-2 | mito | F-box domain containing protein |
| III | 207422 | 207894 | Mg-SINE | - | Y34_scaffold000693-3 | mito | WSC domain-containing protein |
| IV | 482106 | 482579 | Mg-SINE | - | Y34_scaffold000488-4 | mito | hypothetical protein |
| V | 448483 | 448858 | Mg-SINE | - | Y34_scaffold000056-6 | mito | hypothetical protein |
| V | 720716 | 721189 | Mg-SINE | P131_scaffold003177-2 | - | mito | hypothetical protein |
| VI | 2967660 | 2968133 | Mg-SINE | - | Y34_scaffold000231-2 | mito | O-methyltransferase |
| VII | 4464983 | 4465478 | Mg-SINE | P131_scaffold000194-3 | Y34_scaffold001078-2 | mito | hypothetical protein |
| VII | 391134 | 391607 | Mg-SINE | P131_scaffold002099-2 | - | mito | hypothetical protein |
| II | 4540673 | 4540713 | Occan | P131_scaffold004651-1 | Y34_scaffold001580-2 | mito | hypothetical protein |
| VII | 2139952 | 2142641 | Occan | P131_scaffold000227-1 | - | mito | rhoGAP domain containing protein |
| I | 7426997 | 7428854 | Pot2/Pot4 | P131_scaffold006153-1 | Y34_scaffold000110-8 | mito | putative transposase |
| I | 429035 | 429974 | Pot2/Pot4 | - | Y34_scaffold002395-1 | mito | hypothetical protein |
| IV | 3659020 | 3660877 | Pot2/Pot4 | P131_scaffold001827-1 | Y34_scaffold001402-2 | mito | hypothetical protein |
| IV | 40353 | 40943 | Pot2/Pot4 | - | Y34_scaffold007348-1 | mito | 3'-5' exonuclease |
| VII | 1300134 | 1301995 | Pot2/Pot4 | - | Y34_scaffold003236-1 | mito | 2,3-dihydroxybenzoic acid decarboxylase |
| I | 7617328 | 7619188 | Pot3 | - | Y34_scaffold000151-1 | mito | hypothetical protein |
| III | 6123010 | 6124870 | Pot3 | - | Y34_scaffold003765-1 | mito | hypothetical protein |
| I | 2440990 | 2476573 | Pyret | P131_scaffold002814-2 | - | mito | hypothetical protein |
| V | 4689580 | 4698831 | Pyret | P131_scaffold002066-1 | - | mito | metalloproteases |
| VI | 3643204 | 3643985 | RETRO5 | P131_scaffold002962-1 | - | mito | hypothetical protein |
| VII | 253625 | 259748 | RETRO5 | P131_scaffold003488-1 | - | mito | hypothetical protein |
| IV | 382616 | 382808 | RETRO6 | P131_scaffold000127-6 | Y34_scaffold000020-1 | mito | hypothetical protein |
| IV | 1528634 | 1532070 | RETRO6 | - | Y34_scaffold000063-4 | mito | pol polyprotein |
| VI | 321049 | 321244 | RETRO6 | P131_scaffold000278-4 | - | mito | hypothetical protein |
| I | 8340859 | 8341443 | RETRO7 | - | Y34_scaffold002335-1 | mito | FAD dependent oxidoreductase |
| IV | 1194902 | 1195095 | cluster8 | P131_scaffold000759-2 | - | nucl | hypothetical protein |
| V | 1980973 | 1981513 | Maggy | P131_scaffold000175-2 | - | nucl | hypothetical protein |
| II | 1302883 | 1314288 | MGL | P131_scaffold001670-1 | Y34_scaffold000168-1 | nucl | concanavalin A-like lectins/glucanases |
| VII | 2436787 | 2442763 | MGL | P131_scaffold003347-1 | - | nucl | hypothetical protein |
| IV | 3380471 | 3381320 | Mg-MINE | P131_scaffold006900-1 | - | nucl | hypothetical protein |
| III | 3284937 | 3285370 | Mg-SINE | P131_scaffold000246-2 | Y34_scaffold000805-3 | nucl | Sec23/Sec24 trunk domain-containing protein |
| III | 40886 | 41359 | Mg-SINE | - | Y34_scaffold002231-2 | nucl | hypothetical protein |
| VII | 2373258 | 2373731 | Mg-SINE | - | Y34_scaffold000549-1 | nucl | JmjC transcription factor |
| IV | 3185557 | 3187411 | Pot2/Pot4 | - | Y34_scaffold000091-4 | nucl | zf-C2H2 zinc finger protein |
| VI | 4722455 | 4724312 | Pot2/Pot4 | P131_scaffold003267-1 | Y34_scaffold001775-1 | nucl | hypothetical protein |
| VII | 994677 | 996534 | Pot2/Pot4 | - | Y34_scaffold001685-2 | nucl | hypothetical protein |
| VII | 1209711 | 1212353 | Pot2/Pot4 | P131_scaffold002063-1 | - | nucl | hypothetical protein |
| VII | 406983 | 408844 | Pot2/Pot4 | P131_scaffold002111-1 | - | nucl | coiled-coil domain containing protein |
| III | 1900062 | 1900510 | Pyret | - | Y34_scaffold003535-2 | nucl | coiled-coil domain containing protein |
| II | 1661151 | 1661367 | RETRO6 | P131_scaffold001312-1 | - | nucl | putative transposase |
| I | 7517420 | 7523356 | RETRO7 | P131_scaffold006104-1 | Y34_scaffold006665-1 | nucl | hypothetical protein |
| II | 727182 | 733514 | RETRO7 | - | Y34_scaffold010488-1 | nucl | hypothetical protein |
| III | 187557 | 196542 | MGL | P131_scaffold003857-1 | - | plas | ABC transporter related protein |
| II | 1153017 | 1153490 | Mg-SINE | P131_scaffold000334-1 | Y34_scaffold000467-2 | plas | integral membrane protein |
| IV | 1246048 | 1246521 | Mg-SINE | P131_scaffold000941-1 | - | plas | coiled-coil domain containing protein |
| I | 7707549 | 7709409 | Pot2/Pot4 | P131_scaffold000281-2 | - | plas | cytochrome P450 |
| I | 7551072 | 7552931 | Pot2/Pot4 | P131_scaffold000557-1 | - | plas | vitamin H transporter |
| IV | 39584 | 40218 | Pot3 | - | Y34_scaffold007348-1 | plas | 3'-5' exonuclease |
| VI | 4162438 | 4168304 | RETRO6 | P131_scaffold001005-1 | Y34_scaffold001394-2 | plas | hypothetical protein |
| II | 2042771 | 2044628 | Pot2/Pot4 | - | Y34_scaffold000324-2 | plas | hypothetical protein |
