# Supplementary material for: Comparative Analysis of the Genomes of Two Field Isolates of the Rice Blast Fungus Magnaporthe oryzae
Source: PLoS Genet. 2012 Aug 2;8(8):e1002869. doi: 10.1371/journal.pgen.1002869 (PMC3410873; doi:10.1371/journal.pgen.1002869)
Supplement: Table S14 — Genes of isolates Y34 and 70-15 mapped against chromosomal assembly of P131 and found to be disrupted by TE. (DOC) [file pgen.1002869.s022.doc]

**Table S14** Genes of isolates Y34 and 70-15 mapped against chromosomal assembly of P131 and found to be disrupted by TE.

| **Scaffold** | **Begin** | **End** | **TE** | **Y34 gene** | **70-15 gene** | **PSORT** | **Annotation** |
| --- | --- | --- | --- | --- | --- | --- | --- |
| Scaffold000361 | 2692 | 2779 | cluster2 | Y34_Scaffold000532-1 | - | cyto | dynamin like coiled-coil domain containing protein |
| Scaffold000067 | 7154 | 7687 | MGL | Y34_Scaffold004577-1 | - | cyto | hypothetical protein |
| Scaffold000491 | 7836 | 8309 | Mg-SINE | - | MGG_02561 | cyto | hypothetical protein |
| Scaffold001426 | 5666 | 6139 | Mg-SINE | Y34_Scaffold000298-5 | - | cyto | hypothetical protein |
| Scaffold004118 | 1077 | 1548 | Pyret | Y34_Scaffold000289-2 | - | cyto | hypothetical protein |
| Scaffold000133 | 12098 | 12571 | Mg-SINE | Y34_Scaffold003411-2 | - | cyto_nucl | hypothetical protein |
| Scaffold000323 | 3554 | 4026 | Mg-SINE | Y34_Scaffold000994-1 | - | cyto_nucl | glycoside hydrolase |
| Scaffold005447 | 439 | 772 | Maggy | - | MGG_15410 | extr | hypothetical protein |
| Scaffold000743 | 3906 | 4517 | MGL | Y34_Scaffold000007-7 | - | extr | reverse transcriptase |
| Scaffold000342 | 7244 | 7717 | Mg-SINE | - | MGG_10317 | extr | hypothetical protein |
| Scaffold000254 | 5059 | 6916 | Pot2/Pot4 | Y34_Scaffold004041-1 | - | extr | hypothetical protein |
| Scaffold008642 | 309 | 609 | Pot2/Pot4 | - | MGG_13719 | extr | hypothetical protein |
| Scaffold000540 | 6 | 327 | Pot3 | Y34_Scaffold002816-2 | - | extr | 3-phytase A |
| Scaffold006294 | 431 | 624 | RETRO6 | - | MGG_14830 | extr | hypothetical protein |
| Scaffold000041 | 6779 | 6978 | cluster8 | Y34_Scaffold000289-7 | - | mito | hypothetical protein |
| Scaffold000300 | 3614 | 3838 | cluster8 | Y34_Scaffold000325-1 | - | mito | hypothetical protein |
| Scaffold000934 | 1368 | 1559 | cluster8 | Y34_Scaffold003227-1 | - | mito | hypothetical protein |
| Scaffold001027 | 2837 | 3022 | cluster8 | Y34_Scaffold003086-1 | - | mito | hypothetical protein |
| Scaffold000927 | 3078 | 3652 | MGL | Y34_Scaffold000284-5 | - | mito | reverse transcriptase |
| Scaffold000194 | 5088 | 5562 | Mg-SINE | Y34_Scaffold001078-2 | - | mito | hypothetical protein |
| Scaffold000303 | 4171 | 4747 | Mg-SINE | Y34_Scaffold000385-2 | - | mito | cytochrome P450 |
| Scaffold001583 | 2540 | 3013 | Mg-SINE | Y34_Scaffold005212-1 | MGG_05791 | mito | hypothetical protein |
| Scaffold002133 | 1462 | 1935 | Mg-SINE | Y34_Scaffold002231-2 | - | mito | hypothetical protein |
| Scaffold002218 | 3316 | 3977 | Mg-SINE | Y34_Scaffold000448-4 | - | mito | hypothetical protein |
| Scaffold000975 | 2428 | 3126 | Pot2/Pot4 | Y34_Scaffold000020-1 | - | mito | hypothetical protein |
| Scaffold001060 | 5007 | 5052 | Pot2/Pot4 | Y34_Scaffold000436-1 | - | mito | hypothetical protein |
| Scaffold008363 | 1 | 92 | cluster4 | - | MGG_14588 | nucl | ATPase |
| Scaffold000425 | 2869 | 3602 | Mg-MINE | - | MGG_14968 | nucl | hypothetical protein |
| Scaffold002002 | 1758 | 2231 | Mg-SINE | - | MGG_01945 | nucl | hypothetical protein |
| Scaffold003426 | 775 | 2113 | Pyret | Y34_Scaffold002627-1 | - | nucl | hypothetical protein |
| Scaffold000195 | 153 | 744 | RETRO7 | Y34_Scaffold001218-1 | - | nucl | gag protein |
| Scaffold008127 | 171 | 484 | RETRO7 | Y34_Scaffold002389-1 | - | pero | hypothetical protein |
| Scaffold000765 | 8217 | 8275 | cluster3 | - | MGG_15321 | plas | DUF300 domain protein |
| Scaffold000334 | 4377 | 4850 | Mg-SINE | Y34_Scaffold000467-2 | - | plas | pth11-like integral membrane protein |
| Scaffold004153 | 747 | 1288 | Pyret | Y34_Scaffold003535-2 | - | plas | hypothetical protein |
