# Supplementary material for: Comparative Analysis of the Genomes of Two Field Isolates of the Rice Blast Fungus Magnaporthe oryzae
Source: PLoS Genet. 2012 Aug 2;8(8):e1002869. doi: 10.1371/journal.pgen.1002869 (PMC3410873; doi:10.1371/journal.pgen.1002869)
Supplement: Table S15 — Genes of isolates P131 and 70-15 mapped against chromosomal assembly of Y34 and found to be disrupted by TE. (DOC) [file pgen.1002869.s023.doc]

**Table S15** Genes of isolates P131 and 70-15 mapped against chromosomal assembly of Y34 and found to be disrupted by TE.

| **Scaffold** | **Begin** | **End** | **TE** | **70-15 gene** | | **P131 gene** | **PSORT** | **Annotation** |
| --- | --- | --- | --- | --- | --- | --- | --- | --- |
| Scaffold001142 | 2386 | 2858 | Mg-SINE | MGG_02561 | | - | cyto | hypothetical protein |
| Scaffold000759 | 3863 | 4331 | Pot2/Pot4 | | - | P131_Scaffold003771-1 | cyto | proline dipeptidase |
| Scaffold000178 | 549 | 615 | MGL | MGG_07887 | | - | cyto_nucl | flavin-binding monooxygenase |
| Scaffold000056 | 20030 | 20588 | Mg-SINE | - | | P131_Scaffold000682-4 | cyto_nucl | hypothetical protein |
| Scaffold002530 | 1334 | 1807 | Mg-SINE | - | | P131_Scaffold000689-3 | cyto_nucl | similar to phosphoesterase |
| Scaffold000068 | 13052 | 14103 | Pyret | - | | P131_Scaffold000697-4 | cyto_nucl | hypothetical protein |
| Scaffold004575 | 460 | 1164 | Pyret | - | | P131_Scaffold001810-2 | cyto_nucl | hypothetical protein |
| Scaffold000853 | 4094 | 5756 | cluster1 | - | | P131_Scaffold004591-1 | extr | hypothetical protein |
| Scaffold005352 | 885 | 993 | cluster3 | - | | P131_Scaffold001954-1 | extr | hypothetical protein |
| Scaffold000818 | 4562 | 4763 | cluster5 | - | | P131_Scaffold002839-1 | extr | hypothetical protein |
| Scaffold001320 | 6363 | 6469 | Mg-MINE | MGG_14830 | | - | extr | hypothetical protein |
| Scaffold000385 | 10583 | 11055 | Mg-SINE | MGG_03821 | | - | extr | cytochrome P450 monooxygenase |
| Scaffold000448 | 7390 | 7860 | Mg-SINE | - | | P131_Scaffold002218-1 | extr | hypothetical protein |
| Scaffold000468 | 2556 | 3029 | Mg-SINE | MGG_10317 | | - | extr | hypothetical protein |
| Scaffold000789 | 3611 | 4084 | Mg-SINE | MGG_13719 | | P131_Scaffold004411-1 | extr | hypothetical protein |
| Scaffold001015 | 6306 | 6779 | Mg-SINE | - | | P131_Scaffold000010-12 | extr | polyketide synthase |
| Scaffold001207 | 1993 | 2466 | Mg-SINE | - | | P131_Scaffold000713-2 | extr | arylsulfatase |
| Scaffold002231 | 1106 | 1579 | Mg-SINE | MGG_09069 | | - | extr | hypothetical protein |
| Scaffold002866 | 412 | 1245 | Mg-SINE | MGG_02582 | | - | extr | kelch repeat protein |
| Scaffold000704 | 1091 | 1183 | cluster6 | - | | P131_Scaffold000847-2 | mito | hypothetical protein |
| Scaffold006262 | 1 | 399 | MGL | MGG_14477 | | - | mito | GMC oxidoreductase |
| Scaffold000097 | 1578 | 1905 | Mg-MINE | MGG_14625 | | - | mito | reverse transcriptase |
| Scaffold006284 | 1 | 79 | Mg-MINE | - | | P131_Scaffold002410-1 | mito | bZIP transcription factor |
| Scaffold001078 | 3722 | 4196 | Mg-SINE | - | | P131_Scaffold000194-3 | mito | hypothetical protein |
| Scaffold005212 | 325 | 798 | Mg-SINE | MGG_05791 | | - | mito | hypothetical protein |
| Scaffold004379 | 426 | 631 | Pot2/Pot4 | | - | P131_Scaffold000975-3 | mito | hypothetical protein |
| Scaffold000007 | 19926 | 21561 | Pyret | - | | P131_Scaffold004157-1 | mito | hypothetical protein |
| Scaffold000476 | 5182 | 5636 | Pyret | MGG_15314 | | - | mito | hypothetical protein |
| Scaffold000476 | 7013 | 7206 | cluster8 | - | | P131_Scaffold000759-2 | nucl | hypothetical protein |
| Scaffold000005 | 17997 | 18033 | Maggy | MGG_00345 | | - | nucl | serine/threonine-protein kinase ppk18 |
| Scaffold000054 | 18701 | 19632 | MGL | - | | P131_Scaffold000177-2 | nucl | reverse transcriptase |
| Scaffold001749 | 1346 | 1783 | Mg-MINE | MGG_08952 | | - | nucl | hypothetical protein |
| Scaffold000056 | 16590 | 17013 | Mg-SINE | - | | P131_Scaffold000682-5 | nucl | hypothetical protein |
| Scaffold000313 | 6585 | 7059 | Mg-SINE | MGG_15316 | | P131_Scaffold000941-1 | nucl | hypothetical protein |
| Scaffold000270 | 6090 | 6148 | cluster3 | MGG_15321 | | - | plas | hypothetical protein |
| Scaffold004239 | 804 | 837 | Mg-MINE | MGG_02204 | | - | plas | vitamin H transporter |
| Scaffold000376 | 6592 | 7066 | Mg-SINE | - | | P131_Scaffold003802-1 | plas | hypothetical protein |
| Scaffold003057 | 974 | 1504 | Pot2/Pot4 | | - | P131_Scaffold001716-1 | plas | hypothetical protein |
