# Supplementary material for: Comparative Analysis of the Genomes of Two Field Isolates of the Rice Blast Fungus Magnaporthe oryzae
Source: PLoS Genet. 2012 Aug 2;8(8):e1002869. doi: 10.1371/journal.pgen.1002869 (PMC3410873; doi:10.1371/journal.pgen.1002869)
Supplement: Table S16 — Transposable elements located within 1.0 kb of members of the duplicated gene families in 70-15. (DOC) [file pgen.1002869.s024.doc]

**Table S16** Transposable elements located within 1.0 kb of members of the duplicated gene families in 70-15.

| **TE** | **ORTHOMCL** | **Genes** | | | **Annotation** |
| --- | --- | --- | --- | --- | --- |
| cluster2 | ORTHOMCL140 | supercontig_6.10-282 | supercontig_6.10-152 |  | hypothetical protein |
| RETRO6_2 | ORTHOMCL11455 | supercontig_6.12-919 | supercontig_6.16-262 |  | hypothetical protein |
| MGL | ORTHOMCL8 | supercontig_6.13-6 | supercontig_6.13-1184 |  | amidase signature enzyme |
| Pot3 | ORTHOMCL186 | supercontig_6.14-98 | supercontig_6.6-23 |  | NUDIX hydrolase |
| Pot4 | ORTHOMCL199 | supercontig_6.15-171 | supercontig_6.15-174 |  | hypothetical protein |
| Pot2 | ORTHOMCL172 | supercontig_6.17-30 | supercontig_6.15-179 |  | hypothetical protein |
| Pyret_2 | ORTHOMCL115 | supercontig_6.17-58 | supercontig_6.7-96 |  | elongation factor 2 kinase |
| MGL | ORTHOMCL134 | supercontig_6.17-7 | supercontig_6.15-527 |  | hypothetical protein |
| RETRO6_1 | ORTHOMCL11448 | supercontig_6.18-1378 | supercontig_6.21-1302 |  | hypothetical protein |
| MGLR_3 | ORTHOMCL211 | supercontig_6.21-1334 | supercontig_6.21-1399 |  | hypothetical protein |
| MGLR_3 | ORTHOMCL11447 | supercontig_6.21-1410 | supercontig_6.2-15 |  | hypothetical protein |
| RETRO7_1 | ORTHOMCL11447 | supercontig_6.21-1410 | supercontig_6.2-15 |  | hypothetical protein |
| MAGGY | ORTHOMCL61 | supercontig_6.23-110 | supercontig_6.6-20 |  | hypothetical protein |
| RETRO6_2 | ORTHOMCL61 | supercontig_6.23-110 | supercontig_6.2-4 |  | hypothetical protein |
| RETRO7_2 | ORTHOMCL126 | supercontig_6.25-254 | supercontig_6.13-287 |  | hypothetical protein |
| Mg_SINE | ORTHOMCL214 | supercontig_6.28-158 | supercontig_6.12-57 |  | PWL2 |
| Pot2 | ORTHOMCL214 | supercontig_6.28-158 | supercontig_6.12-57 |  | PWL2 |
| RETRO7_1 | ORTHOMCL214 | supercontig_6.28-158 | supercontig_6.12-57 |  | PWL2 |
| Pot2 | ORTHOMCL5524 | supercontig_6.28-165 | supercontig_6.28-130 |  | hypothetical protein |
| Pyret_1 | ORTHOMCL5524 | supercontig_6.28-165 | supercontig_6.28-130 |  | hypothetical protein |
| RETRO5 | ORTHOMCL207 | supercontig_6.28-415 | supercontig_6.28-420 | supercontig_6.28-419 | hypothetical protein |
| cluster6 | ORTHOMCL11445 | supercontig_6.29-890 | supercontig_6.29-895 |  | hypothetical protein |
| cluster3 | ORTHOMCL204 | supercontig_6.30-14 | supercontig_6.29-852 |  | Acyl transferase |
| MAGGY | ORTHOMCL129 | supercontig_6.4-198 | supercontig_6.7-105 | supercontig_6.15-51 | hypothetical protein |
| Mg_SINE | ORTHOMCL116 | supercontig_6.7-94 | supercontig_6.17-56 |  | hypothetical protein |
| Pot4 | ORTHOMCL11446 | supercontig_6.8-155 | supercontig_6.21-1324 |  | hypothetical protein |
| Pot3 | ORTHOMCL149 | supercontig_6.8-210 | supercontig_6.11-45 |  | hypothetical protein |
| RETRO6_1 | ORTHOMCL149 | supercontig_6.8-210 | supercontig_6.11-45 |  | hypothetical protein |
| cluster8 | ORTHOMCL218 | supercontig_6.9-103 | supercontig_6.21-1321 |  | hypothetical protein |
| Pot2 | ORTHOMCL218 | supercontig_6.9-103 | supercontig_6.21-1321 |  | hypothetical protein |
|  |  |  |  |  |  |
