# Supplementary material for: Comparative Analysis of the Genomes of Two Field Isolates of the Rice Blast Fungus Magnaporthe oryzae
Source: PLoS Genet. 2012 Aug 2;8(8):e1002869. doi: 10.1371/journal.pgen.1002869 (PMC3410873; doi:10.1371/journal.pgen.1002869)
Supplement: Table S17 — PCR primers used in this study. (DOC) [file pgen.1002869.s025.doc]

**Table S17 PCR primers used in this study.**

| **Primer** | **Sequence (5'-->3')** |
| --- | --- |
| CFEM_Y34_scaffold00067-1f | ACTTGACCAGACTTGCCTTG |
| CFEM_Y34_scaffold00067-1r | TCGTGTCGATCCAGCTCCAC |
| CFEM_Y34_scaffold00105-2f | GATATTCTACGGACTCCGAG |
| CFEM_Y34_scaffold00105-2r | TCCACCGTCTGAGATATCAC |
| CFEM_Y34_scaffold00399-3f | CTTGGTGGTTCGGACGACAG |
| CFEM_Y34_scaffold00399-3r | CACTCGCATCTGCAAGCTC |
| CHEF_P131_scaffold00006-11f | CTCGAAACACGTCACACTG |
| CHEF_P131_scaffold00006-11r | TCTATCTCCTCCGTCTCAAG |
| CHEF_P131_scaffold00087-106f | GGACGACGCAGAGGAAGTTG |
| CHEF_P131_scaffold00087-106r | TCCGCAGAGGCTCAAGTGAC |
| CHEF_Y34_scaffold00824-1665f | TGTGAGCGATCTGAAGGTTG |
| CHEF_Y34_scaffold00824-1665r | CTGTACTTCGATACGAAGGAC |
| CHEF_Y34_scaffold00875-1778f | ATGATGTCGTAGGTGCAGTG |
| CHEF_Y34_scaffold00875-1778r | CTCGAATGATAACGTGGTTC |
| CHEF_Y34_scaffold01193-2376f | TAGGACTGGTCGCAATGGAC |
| CHEF_Y34_scaffold01193-2376r | AAGCACCATCTGGATCTCAC |
| DUP_P131_scaffold01428-1f | TCTAACCGATAACAGCAGAG |
| DUP_P131_scaffold01428-1r | CGAAGCTACGCTGAGATC |
| DUP_P131_scaffold01531-1f | TTGACTTCTTGCTTTGTGTC |
| DUP_P131_scaffold01531-1r | CAAGCTTGGAACCGTCCTAC |
| DUP_Y34_scaffold00708-1f | GACAAAAGGCTGCAACGTG |
| DUP_Y34_scaffold00708-1r | TCCGTGCACTCCTAAGAATC |
| DUP_Y34_scaffold00846-5f | CTAGAATGCCTCAGCATCTG |
| DUP_Y34_scaffold00846-5r | CGCTCAATGAAGGTCGAC |
| P131_scaffold00006-6f | CTCCCAATCACCAGAGCA |
| P131_scaffold00006-6r | GTGCAGAGTGAGTCGTCGA |
| P131_scaffold00033-1f | AGGTTTCTCGTTACCCTC |
| P131_scaffold00033-1r | CTACATAGTCCTGTCGCA |
| P131_scaffold00050-2f | CTCCATCGTCAAACTGTG |
| P131_scaffold00050-2r | ACGCAGAGTCTAGACTTG |
| P131_scaffold00069-1f | GAAACTCCAACCTGCTG |
| P131_scaffold00069-1r | GCAAGGTCAGGGCTGTC |
| P131_scaffold00087-3f | CAGACATCCACAGCCAAC |
| P131_scaffold00087-3r | CTGACTTTCCTTGACCAC |
| P131_scaffold00088-3f | GACCTGTCAGAAGGCTAC |
| P131_scaffold00088-3r | TAACTCCGATCGTGTCTC |
| P131_scaffold00208-3f | GTTGTCCTCGCTCACCTC |
| P131_scaffold00208-3r | ATGGACCATGGGATTCAC |
| P131_scaffold00259-2f | ACACATGCAAAGTCGCTG |
| P131_scaffold00259-2r | CTCAAGAACCTCCTCATAG |
| P131_scaffold00297-2f | CATGAAGATTCCGATGGTC |
| P131_scaffold00297-2r | CGTCCGTAGATGTGTCAG |
| P131_scaffold00355-2f | AGATCCTTGCCAATGTGAC |
| P131_scaffold00355-2r | AACTTGTTTGCGCTCGAC |
| P131_scaffold00381-1f | ACACGCTACACTTCCTTC |
| P131_scaffold00381-1r | CTCCTCGTTGACGACAAC |
| P131_scaffold00493-1f | TCATCCAGGTCGTAGTC |
| P131_scaffold00493-1r | GCCATAACGTTGTGAG |
| P131_scaffold00513-4f | AGACTGCAATCAAGGAGCT |
| P131_scaffold00513-4r | GGATCCTGTCATGCCAAC |
| P131_scaffold00581-4f | AACACAGGCGTCTGATAC |
| P131_scaffold00581-4r | ACTGCCATATTCTCCGAG |
| P131_scaffold00595-2f | TTAAGACGCTTCGAATCC |
| P131_scaffold00595-2r | CATGATGTCGACTTCTG |
| P131_scaffold00620-1f | CTCGGACCAGGACGAAG |
| P131_scaffold00620-1r | TACGACAGCATGCAATC |
| P131_scaffold00695-1f | TGCTTGCCACCTTTACC |
| P131_scaffold00695-1r | CTTCCATTGCTCGAATCT |
| P131_scaffold00951-7f | TCCTCGGAGATAGCTAAAC |
| P131_scaffold00951-7r | CGCTATTCAAGGTGTCTGG |
| P131_scaffold01110-1f | GAGAAGCGTTTTGACAG |
| P131_scaffold01110-1r | TCTTCCGCATATGGAAG |
| P131_scaffold01206-2f | GAACGTGAACAGTACGAC |
| P131_scaffold01206-2r | ATGGGAGCAAGCAACGAG |
| P131_scaffold01475-1f | CTCCAACTCCAAGTCCA |
| P131_scaffold01475-1r | TTACAAAGCCAGTGGGT |
| P131_scaffold01481-1f | TTCTGCGAGCATCAGT |
| P131_scaffold01481-1r | CGTAGAGATCGTCGTTG |
| P131_scaffold01538-1f | AAGTTCTTCGCTGCTCT |
| P131_scaffold01538-1r | TAGTTGTCGCACAGCTTG |
| P131_scaffold01538-2f | GATCACGCTCGACTCTT |
| P131_scaffold01538-2r | TCCCAGCCAAAGTAGAC |
| P131_scaffold01538-5f | TCCGTCGCAGCCATTTC |
| P131_scaffold01538-5r | AACGAGTGCAGTCCCAGT |
| P131_scaffold01540-2f | TGGTCCTGATGCTCTCGA |
| P131_scaffold01540-2r | CTCAAGCCTTCATTCTCCA |
| P131_scaffold01671-8f | ATGTCGGCAGGGAACAAC |
| P131_scaffold01671-8r | TCAGATCTTCTAGTAGTG |
| P131_scaffold01677-4f | AGGTTCAAGCGACTCTTC |
| P131_scaffold01677-4r | AGCCGACGTTGTACTCAC |
| P131_scaffold01773-2f | ATGCATACCCAGGTCACG |
| P131_scaffold01773-2r | GTCGTTTGTCGTACTCGTC |
| P131_scaffold01777-6f | TTCTGACGAAACGTGGAC |
| P131_scaffold01777-6r | TACTAGCAGTGCGAGGTC |
| P131_scaffold01777-7f | GTCAAGCTGGATTGCAG |
| P131_scaffold01777-7r | CTCCACATCATGCTCCTG |
| P131_scaffold01822-3f | GCTCCAGTCGATCCTTGTC |
| P131_scaffold01822-3r | GTCGTGATTGACCTCGTGT |
| supercontig_6.10-280f | ATCACCATGCTCCTTTC |
| supercontig_6.10-280r | GTTCCTTCCAGGGATTC |
| supercontig_6.12-66f | GAACTAAGGGAATCCGTC |
| supercontig_6.12-66r | ATGAGAAGCTCTGCCAAG |
| supercontig_6.12-67f | ACCGTGATTGTTGCTATC |
| supercontig_6.12-67r | ATTGTCTGTAGGGTTGTC |
| supercontig_6.12-991f | CCAGATTCCTGCTGTTC |
| supercontig_6.12-991r | CGTGGCAGCACTAAATC |
| supercontig_6.13-339f | GATGACTGCTCTCCAGAG |
| supercontig_6.13-339r | ATGGAACGTGATGACATG |
| supercontig_6.15-68f | GCTTGGACGACTACTTG |
| supercontig_6.15-68r | CAACACCCAGGCTAAAG |
| supercontig_6.24-462f | ACCTCCGTCATCCTCAC |
| supercontig_6.24-462r | TGACTCGGTCGTCGTAG |
| supercontig_6.24-491f | CAGCATGATGATGATCTG |
| supercontig_6.24-491r | ATGAGCAACATGAGCATG |
| supercontig_6.28-186f | ATGCAGCTCCACAACGT |
| supercontig_6.28-186r | ATCCACCTGTGCTTGAC |
| supercontig_6.6-13f | GACATGGATGCGTACTG |
| supercontig_6.6-13r | GTATGGGAACAGGTACAG |
| supercontig_6.6-15f | ATGCGTCTCTTCAAGCT |
| supercontig_6.6-15r | TTGTGCCATATCCTTCAG |
| Y34_scaffold00105-1f | TAACATCTGCGGCAGTCG |
| Y34_scaffold00105-1r | ACGGGCTGACATTGATCG |
| Y34_scaffold00105-2f | AGCAGGTGATCGCTCTG |
| Y34_scaffold00105-2f | TGACAGCAATCTTGGTC |
| Y34_scaffold00139-1f | CAGTCAACGGTGTGGTT |
| Y34_scaffold00139-1r | CAAAGGAACAGGCTGGT |
| Y34_scaffold00252-7f | GATATCAAGGAAGCATG |
| Y34_scaffold00252-7r | GTGTGCCAGTCCAGAAC |
| Y34_scaffold00562-1f | AGTATCCTTTCTCGCCATC |
| Y34_scaffold00562-1r | TGACGAACTGAAGTGTGC |
| Y34_scaffold00585-2f | ACTTCTTCTCTTGGCAAAG |
| Y34_scaffold00585-2r | ACGACCGTAAAGGCTTC |
| Y34_scaffold00585-5f | TTGCTCTTTGCCAGTG |
| Y34_scaffold00585-5r | CTGCCCATTTAGGGAT |
| Y34_scaffold00682-1f | CGGTCAACGCTTTCATT |
| Y34_scaffold00682-1r | AGTCGCCGAACAACAAG |
| Y34_scaffold00714-4f | ATACCTTGTCATGAACAG |
| Y34_scaffold00714-4r | TCAAACGTCAGCCAGATG |
| Y34_scaffold00723-1f | CATGCGCTCGTCATCAG |
| Y34_scaffold00723-1r | TACGACCTTGCCACCAC |
| Y34_scaffold00743-5f | CTGCGACCTTGTCCCTG |
| Y34_scaffold00743-5r | CTTCAACTGCTCGAGTG |
| Y34_scaffold00855-11f | TCATGCCTGGAGGCTATC |
| Y34_scaffold00855-11r | TGCGTTTATGTCCGAGTG |
| Y34_scaffold00875-1f | ATCTACCTGGCATTCCTC |
| Y34_scaffold00875-1r | CAATGTACGGCATCTGTC |
| Y34_scaffold00876-1f | ACTAGTGATCCTGAGCA |
| Y34_scaffold00876-1r | GTCAGACTGACGTTGAA |
| Y34_scaffold00877-6f | ATGCCGTACCAACGGAT |
| Y34_scaffold00877-6r | TCGAGTGAAGTATCGCCT |
| Y34_scaffold00879-1f | ATTAGGAAGGGCTCGGA |
| Y34_scaffold00879-1r | TTCTCGCTGTTCACGGT |
| Y34_scaffold00901-1f | ACCGCCAGTTCACAAAG |
| Y34_scaffold00901-1r | AAAGTAGTGCCGATGAC |
| Y34_scaffold00901-3f | TCTTCTCCAGCAGCCTG |
| Y34_scaffold00901-3r | CGTAGCCGTTGATGGTG |
| Y34_scaffold00901-6f | GCAACCATTCTGGCACT |
| Y34_scaffold00901-6r | GCAACCAAACGATTCTC |
| Y34_scaffold01105-1f | TTCTCTCTCGCCATCCT |
| Y34_scaffold01105-1r | TTGCGTAGTCCTTGAG |
| Y34_scaffold01152-4f | TGCAGGAGATCCCAGTC |
| Y34_scaffold01152-4r | AACCTTGGAGATCCCAC |
| Y34_scaffold01176-2f | CAAGGTCACGATTTAC |
| Y34_scaffold01176-2r | AGTGGCGAGAAGTTTG |
| Y34_scaffold01193-4f | TTTGGAAAGCTGCGATGG |
| Y34_scaffold01193-4r | GCCCTTGTATTCGAGTAG |
| P1 (hyg_up) | GACAGACGTCGCGGTGAGTT |
| P2 (hyg_down) | TCTGGACCGATGGCTGTGTAG |
| P131_scaffold00208-2probef | CTTCGACGAGGATTGGGAC |
| P131_scaffold00208-2prober | CGATATCGAGTCGTGAACAG |
| P131_scaffold00208-2KO_LBf | CATGGTACCTGTGCTCCAACGTCTG |
| P131_scaffold00208-2KO_LBr | CATAAGCTTCGCCGGGTGCAATTC |
| P131_scaffold00208-2KO_RBf | CATGAATTCTGGCACCGTGTACTC |
| P131_scaffold00208-2KO_RBr | CATACTAGTGGGTGCGTTTGATCG |
| P11 (P_PS208-2out1) | CCCGATTGGAACATCTGAAC |
| P12 (P_PS208-2out2) | TGAGGCCGCCTGGGTAATAG |
| Y34_scaffold00105-1KO_LBf | GTCACTAGTCTCCAAATATCACGC |
| Y34_scaffold00105-1KO_LBr | CTAGAATTCTGGCGTCATCCTC |
| Y34_scaffold00105-1KO_RBf | GCTAAGCTTCTAGTCTACAGGCCT |
| Y34_scaffold00105-1KO_RBr | CTAGGTACCATGCTATCGATTCTC |
| P13 (P_YS105-1out1) | GAGAGCACTTACGCAGGGTTG |
| P14 (P_YS105-1out2) | ATTCCGTTGTTTATCCTGCGT |
| Y34_scaffold00105-2KO_LBf | CCTGCTCCTATCGCTGACT |
| Y34_scaffold00105-2KO_LBr | TTGACCTCCACTAGCTCCAGCCAAGCCACAAGACACATTGCTCACAGA |
| Y34_scaffold00105-2KO_RBf | GAATAGAGTAGATGCCGACCGGGTCCTTGGAGTATTCCGTAGACA |
| Y34_scaffold00105-2KO_RBr | GTCCAGGCAGTTCTCATACAAT |
| P15 (P_YS105-2out1) | CCTGCTCCTATCGCTGACTCAG |
| P16 (P_YS105-2out2) | GTCCAGGCAGTTCTCATACAAT |
| Y34_scaffold01048-2KO_LBf | CATGAATTCGGGCCTCGGCTTCG |
| Y34_scaffold01048-2KO_LBr | CATGGATCCTCGCGTCCAGGGTTTG |
| Y34_scaffold01048-2KO_RBf | CATGGTACCTTAACCACTGCGCCATC |
| Y34_scaffold01048-2KO_RBr | CATAAGCTTGGTTCACGGCACAGAG |
| P_YS1048-2out | ACTTTAGGCCGCTGGCTTTG |
| P131_scaffold01777-6-7-8KO_LBf | CATACTAGTCCGTAAACGCGATCG |
| P131_scaffold01777-6-7-8KO_LBr | GTGGAATTCTGAATAAGTTGCTCG |
| P131_scaffold01777-6-7-8KO_RBf | GACGTCGACAAGGTGAGGTTTTC |
| P131_scaffold01777-6-7-8KO_RBr | GATGGTACCAGGTTTGACGGC |
| P_PS1777-6-7-8out | CGATCGAGCTTGAGGTGGAT |
| P131_scaffold01784-1-2-3KO_LBf | ATAGGATCCAGCGAGTGATGTTGT |
| P131_scaffold01784-1-2-3KO_LBr | GATGAATTCGGTGACTGTGCTTCT |
| P131_scaffold01784-1-2-3KO_RBf | GTCGTCGACTGAAGCTTTCTCTC |
| P131_scaffold01784-1-2-3KO_RBr | ACTGGGCCCACTGTATCGATATC |
| P_PS1784-1-2-3out | AGACCCTTGACATATTGCGC |
| P131_scaffold00297-2KO_LBf | CATGAATTCGGTGGTGGCGACGAATTTG |
| P131_scaffold00297-2KO_LBr | CATACTAGTGAGGTGCTGCTGGGTTG |
| P131_scaffold00297-2KO_RBf | CATCTCGAGCTGTCCCTGATGCATTG |
| P131_scaffold00297-2KO_RBr | CATAAGCTTTAGGGCCTGCATTGG |
| P_PS297-2out | ACCGCAAGAGGACGTGGAAG |
| P131_scaffold00493-1KO_LBf | CATGAATTCGCCCGGAAGTGGATAC |
| P131_scaffold00493-1KO_LBr | CATACTAGTCTGAGCCTGACCAGAG |
| P131_scaffold00493-1KO_RBf | CATGGTACCCACGAGTCTCGGTAAC |
| P131_scaffold00493-1KO_RBr | CATCTCGAGGGCACAGAGTGAAACG |
| P_PS493-1out | GGCCGTGTTGTTCAATTCCC |
| P131_scaffold00033-1KO_LBf | CAAGAATTCACCGTCCCTGGTTCTG |
| P131_scaffold00033-1KO_LBr | CAAACTAGTCTGCCATTGGAGGGTTG |
| P131_scaffold00033-1KO_RBf | CAACTCGAGGTGCCTGGTGCTTTCT |
| P131_scaffold00033-1KO_RBr | CAAAAGCTTCCCCGGAGGCTTGA |
| P_PS33-1out | AACCAGCGACTGCCATTG |
| P131_scaffold00951-7KO_LBf | CATGAATTCCAGATCAGCGCGAAG |
| P131_scaffold00951-7KO_LBr | CATGGATCCGCCGGTTATGTCCTACAC |
| P131_scaffold00951-7KO_RBf | CATGGTACCTGGGAGCCATTTCTCAC |
| P131_scaffold00951-7KO_RBr | CATAAGCTTCCGATACCGTCGGAAC |
| P_PS951-7out | CACGCCTCATCACTGCCTAC |
| Y34_scaffold01193-2KO_LBf | GCTGAAGGACGTTACGATCGAGGAG |
| Y34_scaffold01193-2KO_LBr | TTGACCTCCACTAGCTCCAGCCAAGCCGCTCTCCGATTCCTATGTTC |
| Y34_scaffold01193-2KO_RBf | ATAGAGTAGATGCCGACCGCGGGTTCAAGATAACTAATATTACAAC |
| Y34_scaffold01193-2KO_RBr | AGCCAACACGCTAATGATGAAGTTCTGC |
| P_YS1193-2out | ACCACCAAAGCCAATTCCTTCG |
| Y34_scaffold00875-1KO_LBf | TGGATGTCATGGCAAGATCTACGTGTC |
| Y34_scaffold00875-1KO_LBr | TTGACCTCCACTAGCTCCAGCCAAGCCTCATTGCTCAACCAGATTCG |
| Y34_scaffold00875-1KO_RBf | ATAGAGTAGATGCCGACCGCGGGTTCATACCACTGATAATTCCACTC |
| Y34_scaffold00875-1KO_RBr | GATTTCTGAGAGCAACTTCTTGACGG |
| P_YS875-1out | AAGTGTGAGCGTGTGCAACG |
| Y34_scaffold00875-3KO_LBf | CTCACACTTTCCGAACAACAACCATG |
| Y34_scaffold00875-3KO_LBr | TTGACCTCCACTAGCTCCAGCCAAGCCCAGAATTGTGTCTATTAACG |
| Y34_scaffold00875-3KO_RBf | ATAGAGTAGATGCCGACCGCGGGTTCAGAAATATCATTGACGAGAAC |
| Y34_scaffold00875-3KO_RBr | TTGAATTGCCTAAGTGGAAGGTCTG |
| P_Y875-3out | TACTGCGTGTGTGAATCAAC |
| Y34_scaffold00857-6KO_LBf | GCGAAGAATTTGGCGTTCGATCAC |
| Y34_scaffold00857-6KO_LBr | TTGACCTCCACTAGCTCCAGCCAAGCCGTAGTTGCGAGAGCAAG |
| Y34_scaffold00857-6KO_RBf | ATAGAGTAGATGCCGACCGCGGGTTCTCATTTGCTCCAGCACCTTG |
| Y34_scaffold00857-6KO_RBr | GCGATGCCTATCGAAGCTGAAAGTC |
| P_YS857-6out | GCTCCAAGACCTTGCATCAG |
| Y34_scaffold00855-11KO_LBf | ACCTGAGTATTAGTTCGGCCTCAC |
| Y34_scaffold00855-11KO_LBr | TTGACCTCCACTAGCTCCAGCCAAGCCGACTGGTAGGAGTACGCAATAG |
| Y34_scaffold00855-11KO_RBf | ATAGAGTAGATGCCGACCGCGGGTTCGTAGCGCAGCACATTATAAC |
| Y34_scaffold00855-11KO_RBr | GAAGATTGCTTGGATTGCGGATG |
| P_YS855-11out | GGCAGCCTTATCGGGACTTC |
| Y34_scaffold00005-1KO_LBf | CAACACAGGAATGCATGAAGTCGGA |
| Y34_scaffold00005-1KO_LBr | TTGACCTCCACTAGCTCCAGCCAAGCCTCACGGCCAGGGTAAGCATAG |
| Y34_scaffold00005-1KO_RBf | ATAGAGTAGATGCCGACCGCGGGTTCAGTGTACAGTTCAGTACGAG |
| Y34_scaffold00005-1KO_RBr | CATGATTATGCAGTCACCAACGAG |
| P_YS5-1out | AAGCGCAGTCCGATTCGCAG |
| NX341f | AGGCACTCTTCCGAGTCGT |
| NX341r | ATGCAGTGTCTGCACGCA |
| NX353f | TCTTCGTGACGATCCGTCTC |
| NX353r | ACTCAGGGTCATCACTACCAC |
| NX445f | TGGAGCTCTGATCGATTG |
| NX445r | AGCAGACTGCAAGACGTGA |
| NX346f | GTCGTAACATTGACGTTCGA |
| NX346r | CAACATGCAGCCAGGATC |
| NX355f | CGACAACACGGTAAGTGACA |
| NX355r | CACTATGACTTGACTCTCGA |
| NX1321f | TCGATGCCGTCATGGTCT |
| NX1321r | GGTGTGGATTGCCAGAGGT |
| NX77f | GTGGACTTTGTCGAGTGGAC |
| NX77r | GTTCCTGACCCAAGCGGAC |
| NX119f | TCTTGGTGAAGACTGCAGCA |
| NX119r | CAGGAGATCGGACATGGTTC |
| NX125f | ACTGCTCATCTGGTCCCTGA |
| NX125r | ATCTGGTAGACGCAGAGAAC |
| NX351f | CTGAACAGCAAGCGACAGAG |
| NX351r | ACCTTGAACCTTGGCATGCA |
| NX352f | TAGGCACTCTCCTTCGCAG |
| NX352r | TGGAACTGAGTCTTCCTGAC |
| NX131f | TCGTCTATCTACGTCGCATG |
| NX131r | CATGGAGCAGACAGACTCAC |
| NX252f | TCGGAGTCGACTCCATCATC |
| NX252r | TTGCCATCGCTCTGGATGTC |
| NX253f | GACTGACGCATCTAGCCTAC |
| NX253r | AGTAGAGTACCACGCTGCAG |
| NX255f | TGTGCCGTAGACGAGGTACT |
| NX255r | CTGGTCGTATACACGTGAG |
| NX310f | CACCGTGACCATCGAGTC |
| NX310r | GCGTGTCATGTCGGAACA |
| NX355f | CGACAACACGGTAAGTGACA |
| NX355r | CACTATGACTTGACTCTCGA |
| NX309f | TTGAGCTGCTCGTGGAGAC |
| NX309r | TGCGTGTCATGTCGGAACA |
| EP6-6f | ATACCGTGCCACCAGAGCA |
| EP6-6r | GAGACATCCGACAACGCAAC |
| EP3-2f | GCTACCCACATTGGAAGTGA |
| EP3-2r | GACATTCAGAGATGGCAACA |
| EP87-3f | TTGCCAGCTCGACTCAGAC |
| EP87-3r | GTGCAGGTATGTTCGCTAGT |
| EP208-3f | CCTGTTCACGACTCGATATC |
| EP208-3r | GAAATGTACCAAACGATGCA |
| EP513-4f | TGATACCGATCTTGTACGAAG |
| EP513-4r | GACTCCATTGGTGAATCGCA |
| EP620-1f | GAGACCAACGTTGCGAAGTC |
| EP620-1r | GCTCAGCATTGTGCTCAGCT |
| EP581-4f | GTCAGCTGCAGACGAAAGCT |
| EP581-4r | TCATGCCGATCTTGATCTGT |
| EP980-1f | GACTACATCTCGACCGATG |
| EP980-1r | ACCAATCCTCCAGAGTCGT |
| EP1323-1f | ATGCAGCTTGTCGTCTCTAG |
| EP1323-1r | GGATGGGCTTGTCGTCATC |
| EP1475-1f | GTTGGGAATCTCGGCTTC |
| EP1475-1r | GTTCGTCCAAGGTTGACGT |
| EY439-2f | CAAGGGTCAGGTAGAGCAAC |
| EY439-2r | CTGAAGGTGACTTGGTGCA |
| EY479-1f | GAACAGTCGAGCAGGCA |
| EY479-1r | TACCATCCCACTGGTCCA |
| EY824-1f | ATAGCAGAGCTACTGGTCAAC |
| EY824-1r | TGTATCGACTGGCTGGAAGT |
| EY824-3f | GATTGGCTCTGGTTCCACTC |
| EY824-3r | CACGTTAACCGCATGTTGTG |
| EY855-11f | CGACATCACTCAGAGCATC |
| EY855-11r | AGTTCGAATCGAGAGCTGGT |
| EY79-3f | TAGCTCACGGAATTGAAGTC |
| EY79-3r | TTCGACGCTGTCTAGGATC |
| EY79-4f | TATCCACGGCTCGTCGAC |
| EY79-4r | ACTGTCCAGGAGAACAGCA |
| EY104-5f | AAGCTGAAGCGTGAGGCAG |
| EY104-5r | GCCTTCTGTGCCACGAAC |
| EY316-2f | CTAGATCCCTTGCGTCGA |
| EY316-2r | CGTAATCCAGCAATCCAG |
| EY870-4f | CACTTCTTGTGCCTGGTGC |
| EY870-4r | GAGGGCTAGGAGATTGATGT |
| EY875-3f | CTCAGACGCGCATGGAGAC |
| EY875-3r | CTCAGATGTGCTGTCCAGCT |
| EY1009-2f | GACTGAAGGATTCGACAAGA |
| EY1009-2r | TCTCCTTGCTACCATCGATG |
| EY1193-3f | ATGATACCTTCCTGCACGTC |
| EY1193-3r | TGCCTTGTCTGTGATCAGCA |
| EY1093-4f | TGAACAACCTCTTCGACCT |
| EY1093-4r | TCTCGTCGTTGTCGACCA |
